# Herbal Medicine Hewei Jiangni Decoction Is Noninferior to Oral Omeprazole for the Treatment of Nonerosive Gastroesophageal Reflux Disease: A Randomized, Double-Blind, and Double-Dummy Controlled Trail

**DOI:** 10.1155/2022/9647003

**Published:** 2022-09-22

**Authors:** Feng Li, Yali Yuan, Di Wu, Junxiang Li, Haixiao Han, Xiaohong Li, Liming Hu, Runhua Chen, Yupu Yao, Yao Jiao, Huihui Chen, Tangyou Mao, Chune Xie

**Affiliations:** ^1^Beijing University of Chinese Medicine, Beijing, China; ^2^Shenzhen Hospital, Beijing University of Chinese Medicine, Shenzhen, China; ^3^Dongfang Hospital, Beijing University of Chinese Medicine, Beijing, China; ^4^Beijing Fengtai YouAnMen Community Service Center, Beijing, China

## Abstract

**Objectives:**

Conventional approaches for patients with nonerosive gastroesophageal reflux disease (NERD) were not satisfactory. This study aimed to evaluate the effectiveness and mechanisms of Chinese herbal medicine Hewei Jiangni Decoction (HWJND) as a novel and promising regimen for NERD.

**Methods:**

A total of 128 patients with NERD were randomly assigned to the Treatment group and Control group. The patients from the Treatment group were administered HWJND (81 g) plus dummy omeprazole (20 mg) daily for 8 weeks, and the others were given dummy HWJND granules (81 g) plus omeprazole (20 mg). The clinical efficacy was assessed using the gastroesophageal reflux disease questionnaire (GERD-Q) scale, patient reported outcomes (PRO) scale, and short form health survey 36 (SF-36) scale at week 4. Moreover, its pharmacological and molecular mechanisms were elucidated based on network pharmacology and molecular docking.

**Results:**

Due to case shedding and other reasons, 109 patients, including 56 in the Treatment group and 53 in the Control group completed this study. Our results showed that HWJND significantly improved heartburn, regurgitation, epigastric pain, nausea, and sleep disturbance, which led to a significant reduction of GERD-Q scores in NERD patients. In addition, PRO scores of NERD patients with HWJND administration were improved, and sufficient relief of physical role, body pain, general health, social function, and mental health on the SF-36 scale was also observed in patients after HWJND treatment. We further showed that the curative effect of HWJND was close to that of omeprazole, except for the better improvement of general health and social function. What's more, the main active ingredients of HWJND included quercetin, beta-sitosterol, naringenin, baicalein, and kaempferol were retrieved, and the protective effects of HWJND against NERD may be closely related to targets such as TNF, IL6, IL1B, MMP9, CXCL8, and EGFR, which were mainly enriched in IL-17 signaling pathway and TNF signaling pathway.

**Conclusion:**

Our findings demonstrate that HWJND is noninferior to oral omeprazole for the treatment of patients with NERD, plays a therapeutic role through multiple targets and diverse pathways, and holds promise for complementary and alternative therapy for the treatment of NERD. This trial is registered with http://www.chictr.org.cn, Chinese Clinical Trials Registry [ChiCTR2200055960].

## 1. Introduction

Gastroesophageal reflux disease (GERD) is a common condition in which the reflux of gastric and/or duodenum contents into the esophagus, with typical clinical symptoms including heartburn and acid reflux [1]. Other atypical symptoms such as pharyngeal discomfort, cough, asthma, belching, chest pain, abdominal distension, and abdominal pain can also occur [2]. Based on the esophagoscopy results, there are three major phenotypes of GERD: nonerosive gastroesophageal reflux disease (NERD), reflux esophagitis (RE), and Barrett's esophagus. The incidence and recurrence rate of GERD are on the rise in Western and Asian countries, and the global average incidence is 13.3% [3]. Refractory characteristics of NERD have seriously affected patient's quality of life and significant burden on health care systems.

Proton pump inhibitors (PPIs) are the preferred drug for the treatment of NERD, but 54.1% of patients do not respond to the standard dose of PPIs [4]. In addition, two-thirds of patients with NERD who respond to PPIs demonstrate symptomatic relapse over time after the withdrawal of PPIs [5]. At the same time, long-term use of PPIs is associated with various limitations, such as kidney disease, dementia, fracture, gastric polyps, and other diseases [6]. Therefore, there is an urgent need to develop new and effective drugs for treating NERD to replace or reduce the use of PPIs preparations. Complementary and alternative medicines, especially herbal medicines, have increasingly been used in the treatment of NERD patients in recent years [7] and have shown an indispensable role in inhibiting inflammatory response during NERD management. HWJND is a new effective Chinese medicine prescription developed by Professor Junxiang Li of Beijing University of Chinese Medicine, consisting of 9 g Scutellaria baicalensis Georgi. [Lamiaceae], 6 g Coptis chinensis Franch. [Ranunculaceae], 9 g Zingiber officinale Roscoe. [Zingiberaceae], 9 g Pinellia cordata N.E.Br. [Araceae], 9 g Fritillaria thunbergii Miq. [Liliaceae], 9 g *Taraxacum* F.H.Wigg. [Asteraceae], 9 g *Gentiana* crassa subsp Halda. [Gentianaceae], 9 g Citrus aurantium *L*. [Rutaceae], 9 g *Trichosanthes* kirilowii Maxim. [Cucurbitaceae], and 3 g *Glycyrrhiza glabra L*. [Fabaceae]. Our previous animal experiments also showed that HWJND had significant resistance to ovalbumin-induced and acid-exposed esophageal morphological changes in rats [8]. However, HWJND, as traditional Chinese medicine, is an empirical medicine, and to date, no clinical trial has reported the efficacy of HWJND. The objective of this study is to examine the efficacy and safety of oral administration of HWJND in the treatment of NERD through a double-blind and double-dummy controlled randomized trial in patients.

## 2. Methods

### 2.1. Study Design

**Figure 1:**
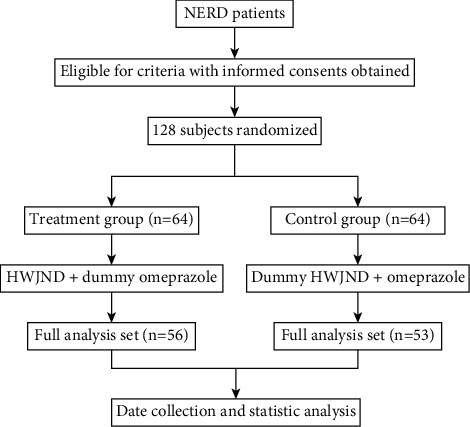
Outlines the study design.

This study was a randomized, double-blind, and double-dummy controlled trial. The study subjects were NERD patients who visited Dongfang Hospital, Beijing University of Chinese Medicine, from December 2018 to June 2021. Patients were randomly divided into the Treatment group and Control group in a 1 : 1 ratio. This study was approved by the IRB Ethics Committee of Dongfang Hospital, Beijing University of Chinese Medicine (JDF-IRB-2018036004) and obtained written informed consent of the patients. The specific flow chart is shown in Figure 1.

### 2.2. Participants

The diagnostic criteria for NERD were the consensus opinions of Chinese gastroesophageal reflux disease experts (2014 edition). Participants aged between 18 and 65 years who met the following diagnostic criteria were included typical reflux and heartburn related symptoms appearing at least 1 day a week; elimination of RE, Barrett's esophagus, and other upper gastrointestinal diseases by esophagogastroduodenoscopy (EGD) in the last 1 month; not receiving acid-inhibitory drugs or gastric mucosal protective agents within 2 weeks; voluntarily participated in this study, written informed consent.

The exclusion criteria were: patients with reflux esophagitis, reflux hypersensitivity, and Barrett's forgettable condition; patients with a history of upper gastrointestinal bleeding or surgery, esophageal stenosis, esophageal and gastric tumors, and other organic lesions; patients with cardiovascular and cerebrovascular diseases, liver, kidney, and hematopoietic system and other serious primary diseases; lactating and pregnant women; patients with a history of mental or neurological disorders or those who are difficult to complete; patients who were currently participating in other clinical trials.

### 2.3. Interventions

A total of 128 patients with NERD were randomly assigned to the Treatment group and Control group. The patients from the Treatment group were administered HWJND (81 g) plus dummy omeprazole (20 mg) daily for 8 weeks, and the others were given dummy HWJND granules (81 g) plus omeprazole (20 mg). HWJND and dummy HWJND are made by Beijing Kangrentang Pharmaceutical Co., LTD. The dummy HWJND granules were prepared by adding cyclodextrin (95%) and the original drug (5%) according to the preparation process of original therapeutic drugs and had the same taste, color, smell, and texture as HWJND granules except for pharmacological effect. Omeprazole and dummy omeprazole were both produced by Shandong New Time Pharmaceutical Co., Ltd., in China. Dummy omeprazole was made from cyclodextrin (80%), rice (15%), and bitters (5%) and had the same taste, color, smell, and texture as the omeprazole.

### 2.4. Randomization and Blinding

Professional clinical trial institutions other than our unit were employed to generate random sequences using statistical analysis system 9.4 (SAS Institute, Cary, NC, USA) for random grouping. Drug information with random numbers and corresponding sample numbers was sealed in an opaque envelope and kept by the clinical study coordinator. The researcher assigned a drug number according to the information of each drug number.

### 2.5. Outcomes

The GERD questionnaire (GERD-Q) scale, which included positive symptoms (heartburn, reflux), negative symptoms (upper abdominal pain, nausea), and positive effects (sleep disturbances, whether OTC medication was used), was used to evaluate the efficacy of patients [9]. The scoring method was used to evaluate each indicator, with higher scores for positive symptoms and positive effects and lower scores for negative symptoms. The Patient Reported Outcomes (PRO) scale, including evaluation in physiological, psychological, and social fields, can comprehensively and accurately evaluate the treatment effect from the perspective of patients and adapt to the clinical characteristics of NERD and the requirements of the modern medical model [10]. The Short Form Health Survey 36 (SF-36) scale was used to evaluate the quality of life, including eight dimensions of physical functioning, role physical, bodily pain, general health, vitality, social functioning, role emotional, and mental health [11]. The raw scores of each dimension were initially calculated and then converted into standard scores according to the formula, with scores ranging from 0 to 100. Standard score = (actual score − lowest possible score/general average possible score) × 100. The better the quality of life, the higher the score; the worse the quality of life, the lower the score.

### 2.6. Sample Size

Due to the lack of previous similar trials and pilot studies to consult, the sample size of this pilot study was set as 64 cases in each group, according to expert opinions.

### 2.7. Construction of a Database of HWJND Main Active Compounds

The active compounds of HWJND were collected from Traditional Chinese Medicine Systems Pharmacology (TCMSP, http://tcmspw.com/tcmsp.php), Traditional Chinese Medicine Integrated Database (TCMID, http://www.megabionet.org/tcmid/), and Chemistry database (http://www.organchem.csdb.cn). We screened the candidate active ingredients of herbs in TCMSP by setting the Oral bioavailability (OB) ≥30% and drug-likeness (DL) ≥0.18, which are the most commonly used screening indicators in network pharmacological analysis. PubChem database (https://pubchem.ncbi.nlm.nih.gov) was used to obtain the corresponding 2D structure of compounds, and we selected the compounds in SwissADME (http://www.swissadme.ch/index.php) based on the 2D structure.

### 2.8. Identified and Predicted Targets of HWJND

TCMSP was used to predict the corresponding protein targets of each active compound of HWJND. The corresponding gene names of the protein targets were further extracted using UniProt Database (https://www.uniprot.org/) with the limitation of ‘Reviewed' and ‘*Homo sapiens*'. Finally, the corresponding gene names were obtained.

### 2.9. Construction of Herbs-Compounds-Targets Network

Based on the aforementioned data, we constructed the Herbs-Compounds-Targets network through Cytoscape 3.7.2, a popular software for analyzing and visualizing the long lists of proteins or genes and integrating biomolecular interaction networks with high-throughput expression data and other molecular states into a unified conceptual framework [12]. In the network, the nodes, determined by the degree value, represent the connection between the compounds and targets. The larger the node, the closer the connection between the compound and the target, and the more important the compound is for the treatment of disease.

### 2.10. Predicting the Targets of NERD

Therapeutic Target Database (TTD, https://db.idrblab.org/ttd/), DisGeNET database (https://www.disgenet.org/), GeneCards database (https://www.genecards.org/), and Online Mendelian Inheritance in Man (OMIM, https://www.omim.org/) were used to retrieve the NERD-related targets through the keyword ‘Nonerosive reflux disease' and ‘Nonerosive reflux disease'. The four databases are combined to obtain disease-related targets after deduplication.

### 2.11. Construction of Protein-Protein Interactions' (PPI) Network

We got the intersection targets of HWJND predicted targets and NERD-related targets by Venny 2.1.0 (https://bioinfogp.cnb.csic.es/tools/venny/). The intersection targets were introduced into the STRING (https://string-db.org) for constructing the protein-protein interactions (PPI). Finally, we visualized the PPI network in Cytoscape 3.7.2 and determined the core targets by the Centiscape 2.2 plugin.

### 2.12. GO and KEGG Pathway Enrichment Analysis

GO and KEGG pathway enrichment of the core targets were analyzed by Metascape (http://metascape.org/gp/index.html), an effective and efficient tool that integrates multiple authoritative databases such as GO, KEGG, UniProt, and DrugBank. GO enrichment analysis, which mainly includes the biological process (BP), cellular composition (CC), and molecular function (MF), and KEGG enrichment analysis were obtained and visualized.

### 2.13. Verification through Molecular Docking

To improve the accuracy and further validate the binding ability between active compounds and key targets, we performed molecular docking with AutoDock vina. Based on the degree values, the three most important core targets from the PPI network were selected as reporter proteins and docked with the first three components of HWJND selected from the herb-compound-target network as molecular ligands. The sdf format of the molecular ligands was obtained in the Pubchem database, and the structures were calculated and exported in mol2 format using ChemBio 3D software. The 3D structures of the core targets were downloaded from the RCSB PDB database (https://www.pdbus.org/). The receptor proteins were dehydrated and their original ligands were removed in PyMOL software, and hydrogenation charge calculation, and construction of the docking grid box were performed with Autodock software. Then, the core targets were docked to the molecular ligands using Autodock Vina. Finally, the docking results were visualized using PyMol software.

### 2.14. Statistical Analysis

Statistical analysis was performed using IBM SPSS Statistics version 20.0. *P* ≤ 0.05 was considered statistically significant. The chi-square test or Student's *t*-test was used to compare the differences between the demographic characteristics of the patients and the clinical characteristics at baseline, with data expressed in absolute value or Mean ± standard deviation (SD). Paired *t*-test was used to compare GERD-Q scores, and the data was represented by Mean ± SD. Wilcoxon rank-sum test was used for differences in heartburn scores, reflux scores, epigastric pain scores, nausea scores, sleep disorder scores, PRO scores, and SF-36 scores, and medians (25th-75th percentiles) were used to express data. The differences in serum ALT, AST, BUN, and Scr levels before and after treatment were determined by Student's *t*-test, and the data was expressed by mean ± SD.

## 3. Results

### 3.1. Demographic Factors and Baseline Characteristics

From December 2018 to June 2021, a total of 128 patients participated in this study and were randomly divided into the Treatment group and Control group in a 1 : 1 ratio. 109 patients, including 56 (87.50%) in the Treatment group and 53 (82.81%) in the Control group, were eligible for inclusion in the study. 19 cases were shed during the study period. 8 patients in the Treatment group had abscission (the causes of abscission included: loss to follow up, *n* = 4; withdrawal of consent, *n* = 3; not satisfied with the therapeutic effect, *n* = 1). 11 cases in the Control group had abscission (the causes of abscission included: lost to follow up, *n* = 3; withdrawal of consent, *n* = 5; not satisfied with the therapeutic effect, *n* = 1; unintended pregnancy, *n* = 2). The patient's demographic and baseline clinical characteristics were shown in Table 1. There were no statistically significant differences in age, gender, eating habits, drinking tea, smoking and alcohol abuse, and other illnesses between the two groups. Similarly, there were no significant differences in weight, BMI, serum ALT, AST, RBC, WBC, PLT, BUN and Scr between Treatment group and Control group.

### 3.2. HWJND Improved the Clinical Symptoms of Patients with NERD

**Figure 2:**
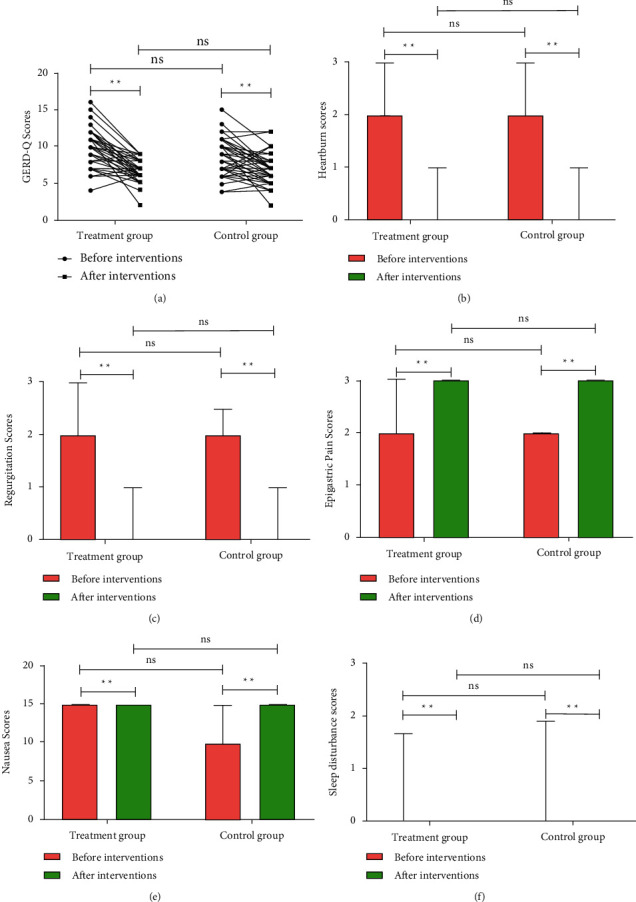
HWJND improved the clinical symptoms of patients with NERD. (a) GERD-Q Scores; (b) Heartburn Scores; (c) Regurgitation Scores; (d) Epigastric Pain Scores; (e) Nausea Scores; (f) Sleep disturbance scores. Treatment group, *n* = 56; Control group, *n* = 53.  ^*∗*^ ^*∗*^*P* < 0.01; n.s., not significant.

The GERD-Q scale, composed of positive symptoms (heartburn, reflux), negative symptoms (upper abdominal pain, nausea), and positive effects (sleep disturbances), was used to evaluate the efficacy of patients [9]. As shown in Figure 2, there were no differences in heartburn, regurgitation, epigastric pain, nausea, or sleep disturbance between Treatment and Control groups before interventions, suggesting the same baseline in GERD-Q scores between the two groups. After 8 weeks of interventions, administration of HWJND significantly improved above discomfort symptoms (*P* < 0.01), and there were no statistically significant differences between the two groups. These results strongly demonstrated a protective role of HWJND in the treatment of patients with NERD.

### 3.3. HWJND Increased the Physiological, Psychological, and Social Abilities of Patients with NERD

**Figure 3:**
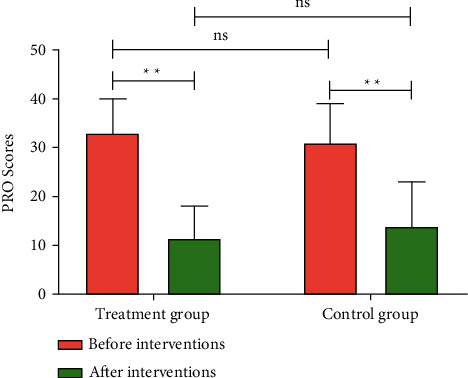
HWJND increased the physiological, psychological, and social abilities of patients with NERD. PRO Scores. Treatment group, *n* = 56; Control group, *n* = 53. ^*∗*^ ^*∗*^*P* < 0.01; n.s., not significant.

The PRO scale could comprehensively and accurately evaluate the treatment effects from the perspective of patients and adapt to the clinical characteristics of NERD and the requirements of the modern medical model.  Figure 3 shows that there was no difference in PRO scores between the Treatment group and Control group before treatment. After 8 weeks of interventions, PRO scores decreased significantly in both groups (*P* < 0.01), even though there was no statistical difference in PRO scores between the two groups, indicating that HWJND increased the physiological, psychological, and social abilities of patients with NERD.

### 3.4. HWJND Improved the Quality of Life of Patients with NERD

**Figure 4:**
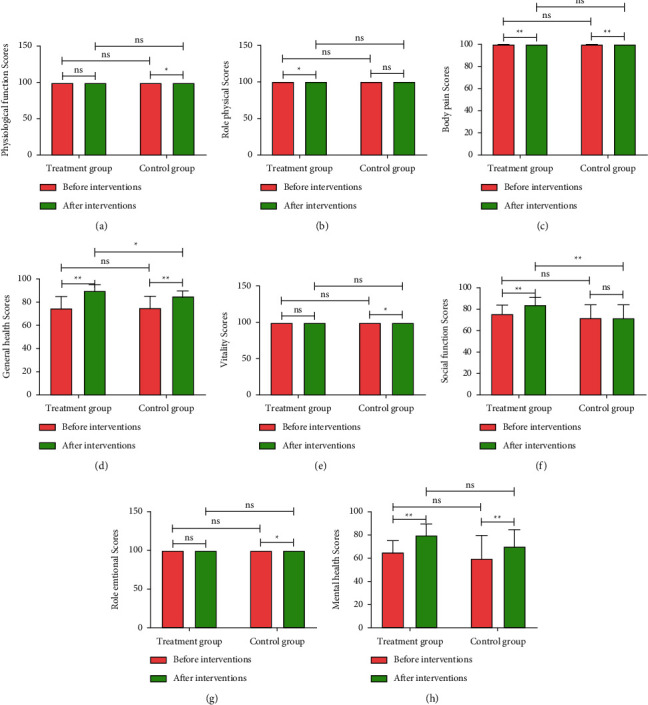
HWJND improved the quality of life of patients with NERD. (a) Physiological function scores;(b) Role physical scores;(c) Body pain scores;(d) General health scores;(e) Vitality scores;(f) Social function scores;(g) Role emotional scores;(h) Mental health scores. Treatment group, *n* = 56; Control group, *n* = 53.  ^*∗*^ ^*∗*^*P* < 0.01; ^*∗*^*P* < 0.05; n.s., not significant.

The SF-36 scale was used to evaluate the quality of life, including eight dimensions of physical functioning, role physical, bodily pain, general health, vitality, social functioning, role emotional, and mental health [11].  Figure 4 showed that there was no difference in the scores of each indicator between the Treatment group and the Control group before interventions. Our results showed that sufficient relief of role physical, body pain, general health, social function, and mental health on SF-36 scale was observed in patients after HWJND treatment (*P* < 0.05 or *P* < 0.01). Notably, the curative effect of HWJND was close to that of omeprazole, except for the better improvement of general health and social function (*P* < 0.05 or *P* < 0.01).

### 3.5. Safety Assessment

**Figure 5:**
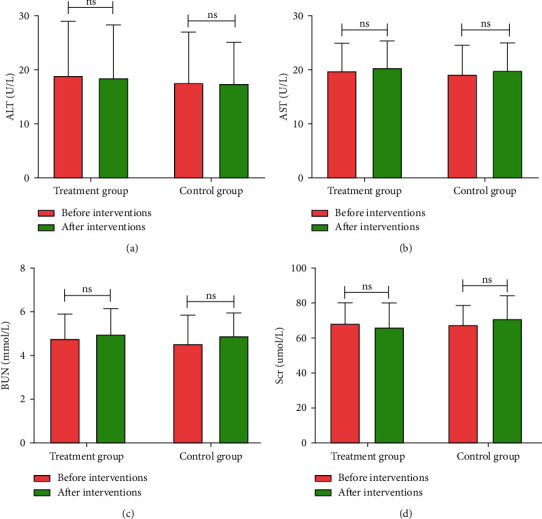
Safety assessment. (a) ALT levels; (b) AST levels; (c) BUN levels; (d) Scr levels. Treatment group, *n* = 56; Control group, *n* = 53. n.s., not significant.

Studies have shown that some Chinese herbal medicines have certain damage to liver and kidney function [13, 14]. Therefore, we tested serum ALT, AST, BUN, and Scr before and after enrollments. The results in Figure 5 showed that there was no statistically significant difference in serum ALT, AST, BUN, and Scr levels between 2 groups before and after interventions. Collectively, these results demonstrated that HWJND has no liver or kidney damage and hold promise to be a candidate drug for the treatment of NERD.

### 3.6. Pharmacological and Molecular Mechanisms of HWJND on NERD Based on Network Pharmacology

**Figure 6:**
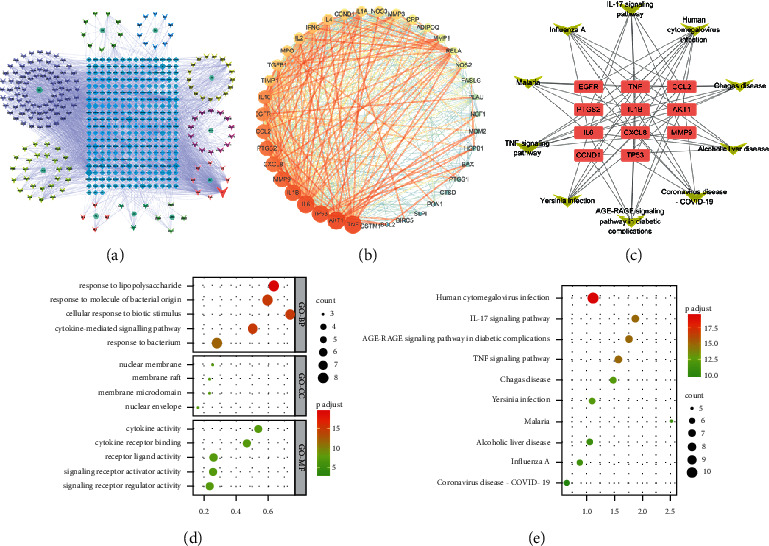
Network pharmacology. (a) Herbs-Compounds-Targets (H-C-T) network diagram; (b) PPI network diagram of targets for HWJND treating GERD; (c) Targets-Signal pathways network diagram; (d) GO enrichment analysis; (e) KEGG pathway enrichment analysis.

To further explore the pharmacological and molecular mechanisms of HWJND on NERD, network pharmacology was carried. A total of 180 active compounds and 283 targets of HWJND were retrieved after deleting the repetition. Detailed information is provided in Supplementary materials 1. Based on the degree value in the herbs-compounds-targets network (Figure 6(a)), the top 5 compounds were CPL (quercetin, degree = 421), XQM (beta-sitosterol, degree = 112), CS (aringenin, degree = 71), XQ1 (baicalein, degree = 68), and GC11 (kaempferol, degree = 58). We obtained 688 NERD-related targets after the four databases were merged and deduplicated. The detailed information is provided in Supplementary materials 2. A total of 44 common targets were obtained after 283 targets of HWJND were combined with 688 targets of GERD-related targets. The common targets were submitted to the STRING database to construct the PPI network, visualized via Cytoscape 3.7.2 (Figure 6(b)), and further filtered for 12 core targets by the Centiscape 2.2 plugin. In which TNF, IL6, IL1B, MMP9, CXCL8, and EGFR play an important role in the treatment of NERD with HWJND.

The results of GO and KEGG pathway enrichment analysis included 399 biological processes (BP), 4 cell components (CC), 11 molecular functions (MF) (*P*-value<0.01) (Figure 6(d) for the top five), and 83 KEGG pathways. The main pathway included IL-17 signaling pathway and TNF signaling pathway as well as others (*P*-value<0.01) (Figure 6(e) for the top ten). The network diagram of “Targets-Signal pathways” was constructed to show the relationship between the signal pathways and the targets (Figure 6(c)). The detailed information of GO and KEGG pathway enrichment analysis is provided in Supplementary materials 3. Altogether, these results showed that the protective effects of HWJND against NERD may be closely related to targets such as TNF, IL6, IL1B, MMP9, CXCL8 and EGFR, and IL-17 signaling pathway and TNF signaling pathway may play a fundamental role of in the treatment of NERD by HWJND.

### 3.7. Verification with Molecular Docking

**Figure 7:**
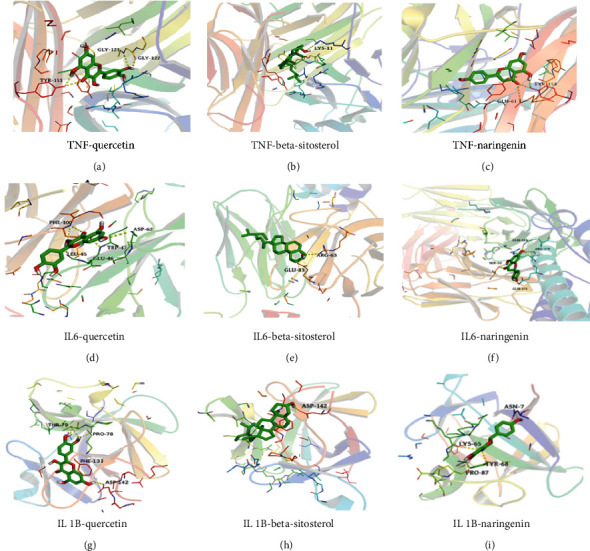
The protein-ligand of the docking simulation. Molecular docking of the three important targets (TNF, IL6, IL1B) in the PPI network and the three most important active components (quercetin, beta-sitosterol, and naringenin) in the H-C-T network were performed.

Molecular docking of the three important targets (TNF, IL6, IL1B) in the PPI network and the three most important active components (quercetin, beta-sitosterol, naringenin) in the H-C-T network were performed separately, and the lowest binding energies of all receptors and ligands were <−5.0 kcal/mol, indicating that the active molecule docked well with the proteins. The affinity energy of best mode IL6-quercetin, IL6-beta-sitosterol, and IL6-naringenin were −7.8 kcal/mol, −6.8 kcal/mol, and −7.3 kcal/mol. The affinity energy of best mode TNF-quercetin, TNF-beta-sitosterol, and TNF-naringenin were −10.4 kcal/mol, −11.3 kcal/mol, and −10.4 kcal/mol. The affinity energy of best mode IL1B-quercetin, IL1B-beta-sitosterol, and IL1B-naringenin was −7.0 kcal/mol, −6.5 kcal/mol, and −7.1 kcal/mol. The above molecular docking results were visualized using PyMol software (Figure 7). Detailed information of molecular docking is provided in Supplementary materials 4. Therefore, the above results provided sufficient evidence to demonstrate the role of these three targets (TNF, IL6, IL1B) in the treatment of NERD by HWJDN.

## 4. Discussion

In this study, we found that HWJND significantly alleviated the clinical symptoms of patients with NERD and improved their physical, psychological, and social abilities, and the therapeutic effect was noninferior to oral omeprazole. At the same time, HWJND improved the quality of life of patients with NERD to a certain extent, especially had a better improvement in general health and social function than that of omeprazole. In addition, safety examination also showed that HWJND did not cause drug-induced damage to the liver and kidneys of patients with NERD.

The pathogenesis of NERD is generally believed to be associated with impaired lower esophageal sphincter (LES) function and transient lower esophageal sphincter relaxations (TLESRs), abnormal esophageal mucosal barrier, defective esophageal peristalsis, and visceral hypersensitivity [15–18]. PPIs and histamine-2 receptor antagonists are usually used to improve regurgitation and heartburn, and gastroprokinetic drugs are used to improve the symptoms of abdominal distension and nausea [19]. However, PPIs is superior to histamine-2 receptor antagonist in the treatment of major symptoms such as heartburn and reflux, so it is used as the first-line treatment for NERD after the failure of lifestyle intervention [20, 21]. Therefore, PPIs remain the most widely used treatment for NERD. Unfortunately, there are still many patients whose clinical symptoms could not be significantly relieved after taking PPIs, and many mechanisms have been proposed to explain the failure of PPIs treatment for patients with NERD, including drug resistance of PPIs, gas or liquid reflux, primary esophageal motion disorders, etc. [22]. In addition, NERD is a chronic, relapsing disease whose symptoms affect the patient's mental and emotional well-being. In severe cases, anxiety and depression occur together, which eventually lead to a decline in the quality of life of patients [23, 24]. Therefore, there is an urgent need for the development of novel and safe therapeutic strategies for treating NERD.

In recent years, more and more scholars have realized that TCM has its unique advantages in the treatment of NERD. The results of a meta-analysis showed that TCM significantly improved the total clinical response rate and symptom relief and reduced the recurrence rate and adverse events in the treatment of NERD [7], which has also been further confirmed in our study and showed that HWJND significantly relieved heartburn and reflux of positive symptoms in patients with NERD and significantly improved negative symptoms and alleviated positive effects such as insomnia caused by positive symptoms, leading to a significant reduction of GERD-Q scores in NERD patients. Moreover, our results also showed that HWJND effectively reduced PRO scores of patients with NERD, indicating that HWJND improved patients' abilities in physical, psychological and social fields. Results from the SF-36 scale showed sufficient relief of role physical, body pain, general health, social function, and mental health in SF-36 scale after HWJND treatment.

The pharmacological and molecular mechanisms of HWJND in the treatment of NERD have been clearly elucidated based on network pharmacology and molecular docking. The main active ingredients of HWJND including quercetin, beta-sitosterol, naringenin, baicalein, and kaempferol can stimulate gastric mucus secretion, block H^+^ [25], regulate the inflammatory response of the esophagus [26–28], repair mucosal damage [29, 30], and thus play a therapeutic role in GERD. The core targets of HWJND for the treatment of GERD include TNF, IL6, IL1B, MMP9, CXCL8 EGFR, etc. Among them, TNF, IL6, and IL1B are inflammatory cytokines, which can not only damage the esophageal mucosal barrier function of the GERD mouse [30–32] but activate acid-sensitive receptors on afferent nerves and epithelial cells of the esophageal mucosa, stimulating neurogenic inflammation and pain, leading to DIS and further increase in epithelial barrier permeability [33]. In addition, through endoscopic biopsy, IL-1B and TNF-*α* may help distinguish AAE and NAE in NERD [34]. MMP9 expresses in esophageal tissue of GERD patients, and is most common in severe forms compared to the mild forms [35]. CXCL8 is also known as neutrophil activating factor (NAF) and interleukin 8 (IL-8) [36], which can aggravate the inflammatory response of the esophageal mucosa. EGFR plays an important role in epithelial repair and in the progression of GERD [37]. The molecular docking results showed that the docking conformation of the core target and the active ingredient of the drug was stable. The KEGG pathway enrichment analysis predicted that HWJND treated NERD through multiple pathways. IL-17 signaling pathway plays a highlighted role in GERD [38], which not only protect the mucosal barrier but also stimulate tissue regeneration and restore the barrier function when the tissue is damaged [39]. As mentioned above, TNF, as a major inflammation cytokine, is closely related to GERD. MAPK, NF-*κ*B signaling pathway, and other pathways can be activated when TNF is combined with TNFR1 [40]. Moreover, through the analysis of the KEGG signaling pathway, the results showed that the HWJND mainly influenced the immune-inflammatory pathways of GERD.

Taken together, these results demonstrated that HWJND was noninferior to oral omeprazole for the treatment of patients with NERD, played a therapeutic role through multiple targets and diverse pathways, and hold promise for complementary and alternative therapy for the treatment of NERD. However, there are still some deficiencies in our study. We did not conduct 24-hour pH monitoring in the recruited patients, which may have led to the inclusion of some patients with reflux hypersensitivity, resulting in lower efficacy than expected. Moreover, the protective mechanism of HWJND on NERD should be further explored.

## Figures and Tables

**Table 1:** Patient demographics and baseline clinical characteristics.

Characteristics	Treatment group (*n* = 56)	Control group (*n* = 53)	*P*-value	Testing method
Age (mean, SD)	48.05 ± 10.91	48.51 ± 10.31	0.823	Student ‘s *t*-test
Gender (%)			0.204	Chi-square test
Male	12 (21.43)	16 (30.19)		
Female	44 (78.57)	37 (69.81)		
Eating habits (%)			0.398	Chi-square test
Like to eat spicy greasy food	16 (28.57)	13 (24.53)		
A balanced diet	40 (71.43)	40 (75.47)		
Have the habit of drinking tea (%)			0.463	Chi-square test
Tea drinking habit	12 (21.43)	10 (18.87)		
No habit of drinking tea	44 (78.57)	43 (81.13)		
Have a history of smoking and alcohol abuse (%)			0.465	Chi-square test
Smoking and alcohol abuse	4 (7.14)	5 (9.43)		
No history of smoking or alcohol abuse	52 (92.86)	48 (90.57)		
Have a history of other diseases (%)			0.569	Chi-square test
History of other illnesses	7 (12.50)	7 (13.21)		
No history of other diseases	49 (87.50)	46 (86.79)		
Serum ALT (U/L) (Mean, SD)	18.89 ± 10.08	17.62 ± 9.43	0.505	Student ‘s *t*-test
Serum AST (U/L) (Mean, SD)	19.90 ± 5.08	19.08 ± 5.49	0.427	Student ‘s *t*-test
RBC (10^9^/L) (Mean, SD)	4.69 ± 0.45	4.69 ± 0.46	0.984	Student ‘s *t*-test
WBC (10^12^/L) (Mean, SD)	5.93 ± 1.34	5.96 ± 1.30	0.904	Student ‘s *t*-test
PLT (10^9^/L) (Mean, SD)	247.30 ± 54.45	261.41 ± 59.09	0.202	Student ‘s *t*-test
BUN (mmol/L) (Mean, SD)	4.78 ± 1.12	4.57 ± 1.29	0.362	Student ‘s *t*-test
Scr (umol/L) (Mean, SD)	69.14 ± 11.60	68.27 ± 11.02	0.694	Student ‘s *t*-test
Weight (kg) (Mean, SD)	63.18 ± 10.20	63.79 ± 9.73	0.749	Student ‘s *t*-test
BMI (kg/m^2^) (Mean, SD)	23.52 ± 3.19	23.83 ± 3.39	0.630	Student ‘s *t*-test

## Data Availability

The data used to support the findings of this study can be obtained from the corresponding author upon request.

## Supplementary Materials

Supplementary Materials (1) The active compounds and potential targets of HWJND. Supplementary materials. (2) The information of GERD-related targets. (3) The information on GO and KEGG pathway enrichment analysis. (4) The information of molecular docking.
