# Supplementary material for: Herbal Medicine Hewei Jiangni Decoction Is Noninferior to Oral Omeprazole for the Treatment of Nonerosive Gastroesophageal Reflux Disease: A Randomized, Double-Blind, and Double-Dummy Controlled Trail
Source: Evid Based Complement Alternat Med. 2022 Sep 22;2022:9647003. doi: 10.1155/2022/9647003 (PMC9522514; doi:10.1155/2022/9647003)
Supplement: Supplementary Materials — (1) The active compounds and potential targets of HWJND. Supplementary materials. (2) The information of GERD-related targets. (3) The information on GO and KEGG pathway enrichment analysis. (4) The information of molecular docking. [file 9647003.f1.zip › 9647003.f1/Supplementary materials 1 the active compounds and potential targets of HWJND.pdf]

Pinellia

Abbreviation	Molecule Name	Target Name	Genesymbol
BX1	24-Ethylcholest-4-en-3-one	Progesterone receptor	PGR
BX1	24-Ethylcholest-4-en-3-one	Mineralocorticoid receptor	NR3C2
BX2	Cavidine	Prostaglandin G/H synthase 1	PTGS1
BX2	Cavidine	Muscarinic acetylcholine receptor M3	CHRM3
BX2	Cavidine	Potassium voltage-gated channel subfamily H member 2	KCNH2
BX2	Cavidine	Muscarinic acetylcholine receptor M1	CHRM1
BX2	Cavidine	Beta-1 adrenergic receptor	ADRB1
BX2	Cavidine	Sodium channel protein type 5 subunit alpha	SCN5A
BX2	Cavidine	Coagulation factor X	F10
BX2	Cavidine	Muscarinic acetylcholine receptor M5	CHRM5
BX2	Cavidine	Prostaglandin G/H synthase 2	PTGS2
BX2	Cavidine	5-hydroxytryptamine 3 receptor	HTR3
BX2	Cavidine	Alpha-2C adrenergic receptor	ADRA2C
BX2	Cavidine	Muscarinic acetylcholine receptor M4	CHRM4
BX2	Cavidine	Retinoic acid receptor RXR-alpha	RXRA
BX2	Cavidine	Delta-type opioid receptor	OPRD1
BX2	Cavidine	5-hydroxytryptamine 2A receptor	HTR2A
BX2	Cavidine	5-hydroxytryptamine 2C receptor	HTR2C
BX2	Cavidine	Alpha-1B adrenergic receptor	ADRA1B
BX2	Cavidine	Beta-2 adrenergic receptor	ADRB2
BX2	Cavidine	Alpha-1D adrenergic receptor	ADRA1D
BX2	Cavidine	DNA topoisomerase 2-alpha	TOP2A
BX2	Cavidine	Mu-type opioid receptor	OPRM1
BX2	Cavidine	Heat shock protein HSP 90-alpha	HSP90AA1
BX2	Cavidine	D(1A) dopamine receptor	DRD1
BX2	Cavidine	Sodium-dependent serotonin transporter	SLC6A4
BX2	Cavidine	Coagulation factor VII	F7
BX2	Cavidine	cAMP and cAMP-inhibited cGMP 3',5'-cyclic phosphodiesterase 10A	PDE10A
XQ1	baicalein	Prostaglandin G/H synthase 1	PTGS1
XQ1	baicalein	Androgen receptor	AR
XQ1	baicalein	Prostaglandin G/H synthase 2	PTGS2
XQ1	baicalein	Heat shock protein HSP 90-alpha	HSP90AA1
XQ1	baicalein	cAMP-dependent protein kinase catalytic subunit alpha	PRKACA
XQ1	baicalein	Dipeptidyl peptidase 4	DPP4
XQ1	baicalein	Phosphatidylinositol-4,5-bisphosphate 3-kinase catalytic subunit gamma isoform	PIK3CG
XQ1	baicalein	cGMP-inhibited 3',5'-cyclic phosphodiesterase A	PDE3A
XQ1	baicalein	Trypsin-1	PRSS1
XQ1	baicalein	Nuclear receptor coactivator 2	NCOA2
XQ1	baicalein	Nuclear receptor coactivator 1	NCOA1
XQ1	baicalein	Transcription factor p65	RELA
XQ1	baicalein	RAC-alpha serine/threonine-protein kinase	AKT1
XQ1	baicalein	Apoptosis regulator Bcl-2	BCL2
XQ1	baicalein	Proto-oncogene c-Fos	FOS
XQ1	baicalein	Apoptosis regulator BAX	BAX
XQ1	baicalein	Matrix metalloproteinase-9	MMP9
XQ1	baicalein	Caspase-3	CASP3
XQ1	baicalein	Cellular tumor antigen p53	TP53

XQ1	baicalein	Hypoxia-inducible factor 1-alpha	HIF1A
XQ1	baicalein	Fos-related antigen 1	FOSL1
XQ1	baicalein	Fos-related antigen 2	FOSL2
XQ1	baicalein	Cell division control protein 2 homolog	#N/A
XQ1	baicalein	G2/mitotic-specific cyclin-B1	CCNB1
XQ1	baicalein	Myeloperoxidase	MPO
XQ1	baicalein	Aryl hydrocarbon receptor	AHR
XQ1	baicalein	Insulin-like growth factor II	IGF2
XQ1	baicalein	Cytochrome c	CYCS
XQ1	baicalein	Nuclear factor of activated T-cells, cytoplasmic 1	NFATC1
XQ1	baicalein	Tudor domain-containing protein 7	TDRD7
XQ1	baicalein	Egl nine homolog 1	EGLN1
XQ1	baicalein	NADPH oxidase 5	NOX5
XQ1	baicalein	Fatty acid-binding protein, epidermal	FABP5
XQ1	baicalein	Apolipoprotein D	APOD
BX3	Baicalin	Coagulation factor X	F10
BX3	Baicalin	Tyrosine-protein phosphatase non-receptor type 1	PTPN1
XQM	beta-sitosterol	Progesterone receptor	PGR
XQM	beta-sitosterol	Nuclear receptor coactivator 2	NCOA2
XQM	beta-sitosterol	Prostaglandin G/H synthase 1	PTGS1
XQM	beta-sitosterol	Prostaglandin G/H synthase 2	PTGS2
XQM	beta-sitosterol	Heat shock protein HSP 90-alpha	HSP90AA1
XQM	beta-sitosterol	Phosphatidylinositol-4,5-bisphosphate 3-kinase catalytic subunit gamma isoform	PIK3CG
XQM	beta-sitosterol	Potassium voltage-gated channel subfamily H member 2	KCNH2
XQM	beta-sitosterol	cAMP-dependent protein kinase catalytic subunit alpha	PRKACA
XQM	beta-sitosterol	D(1A) dopamine receptor	DRD1
XQM	beta-sitosterol	Muscarinic acetylcholine receptor M3	CHRM3
XQM	beta-sitosterol	Muscarinic acetylcholine receptor M1	CHRM1
XQM	beta-sitosterol	Sodium channel protein type 5 subunit alpha	SCN5A
XQM	beta-sitosterol	Gamma-aminobutyric-acid receptor subunit alpha-2	GABRA2
XQM	beta-sitosterol	Muscarinic acetylcholine receptor M4	CHRM4
XQM	beta-sitosterol	cGMP-inhibited 3',5'-cyclic phosphodiesterase A	PDE3A
XQM	beta-sitosterol	5-hydroxytryptamine 2A receptor	HTR2A
XQM	beta-sitosterol	Gamma-aminobutyric-acid receptor subunit alpha-5	GABRA5
XQM	beta-sitosterol	Alpha-1A adrenergic receptor	ADRA1A
XQM	beta-sitosterol	Gamma-aminobutyric-acid receptor subunit alpha-3	GABRA3
XQM	beta-sitosterol	Muscarinic acetylcholine receptor M2	CHRM2
XQM	beta-sitosterol	Alpha-1B adrenergic receptor	ADRA1B
XQM	beta-sitosterol	Beta-2 adrenergic receptor	ADRB2
XQM	beta-sitosterol	Neuronal acetylcholine receptor subunit alpha-2	CHRNA2
XQM	beta-sitosterol	Sodium-dependent serotonin transporter	SLC6A4
XQM	beta-sitosterol	Mu-type opioid receptor	OPRM1
XQM	beta-sitosterol	Gamma-aminobutyric-acid receptor subunit alpha-1	GABRA1
XQM	beta-sitosterol	Neuronal acetylcholine receptor subunit alpha-7	CHRNA7
XQM	beta-sitosterol	Apoptosis regulator Bcl-2	BCL2

XQM	beta-sitosterol	Apoptosis regulator BAX	BAX
XQM	beta-sitosterol	Caspase-9	CASP9
XQM	beta-sitosterol	Transcription factor AP-1	JUN
XQM	beta-sitosterol	Caspase-3	CASP3
XQM	beta-sitosterol	Caspase-8	CASP8
XQM	beta-sitosterol	Protein kinase C alpha type	PRKCA
XQM	beta-sitosterol	Transforming growth factor beta-1	TGFB1
XQM	beta-sitosterol	Serum paraoxonase/arylesterase 1	PON1
XQM	beta-sitosterol	Microtubule-associated protein 2	MAP2
XQ2	Stigmasterol	Progesterone receptor	PGR
XQ2	Stigmasterol	Mineralocorticoid receptor	NR3C2
XQ2	Stigmasterol	Nuclear receptor coactivator 2	NCOA2
XQ2	Stigmasterol	Retinoic acid receptor RXR-alpha	RXRA
XQ2	Stigmasterol	Nuclear receptor coactivator 1	NCOA1
XQ2	Stigmasterol	Prostaglandin G/H synthase 1	PTGS1
XQ2	Stigmasterol	Prostaglandin G/H synthase 2	PTGS2
XQ2	Stigmasterol	Alpha-2A adrenergic receptor	ADRA2A
XQ2	Stigmasterol	Sodium-dependent noradrenaline transporter	SLC6A2
XQ2	Stigmasterol	Sodium-dependent dopamine transporter	SLC6A3
XQ2	Stigmasterol	Beta-2 adrenergic receptor	ADRB2
XQ2	Stigmasterol	Urokinase-type plasminogen activator	PLAU
XQ2	Stigmasterol	Leukotriene A-4 hydrolase	LTA4H
XQ2	Stigmasterol	Amine oxidase [flavin-containing] B	MAOB
XQ2	Stigmasterol	Amine oxidase [flavin-containing] A	MAOA
XQ2	Stigmasterol	cAMP-dependent protein kinase catalytic subunit alpha	PRKACA
XQ2	Stigmasterol	Chymotrypsinogen B	CTRB1
XQ2	Stigmasterol	Muscarinic acetylcholine receptor M3	CHRM3
XQ2	Stigmasterol	Muscarinic acetylcholine receptor M1	CHRM1
XQ2	Stigmasterol	Beta-1 adrenergic receptor	ADRB1
XQ2	Stigmasterol	Sodium channel protein type 5 subunit alpha	SCN5A
XQ2	Stigmasterol	5-hydroxytryptamine 2A receptor	HTR2A
XQ2	Stigmasterol	Alpha-1A adrenergic receptor	ADRA1A
XQ2	Stigmasterol	Gamma-aminobutyric-acid receptor subunit alpha-3	GABRA3
XQ2	Stigmasterol	Muscarinic acetylcholine receptor M2	CHRM2
XQ2	Stigmasterol	Alpha-1B adrenergic receptor	ADRA1B
XQ2	Stigmasterol	Gamma-aminobutyric-acid receptor subunit alpha-1	GABRA1
XQ2	Stigmasterol	Neuronal acetylcholine receptor subunit alpha-7	CHRNA7
BX4	gondoic acid	Prostaglandin G/H synthase 1	PTGS1
BX4	gondoic acid	Nuclear receptor coactivator 2	NCOA2
BX5	coniferin	Muscarinic acetylcholine receptor M3	CHRM3
BX5	coniferin	Muscarinic acetylcholine receptor M1	CHRM1
BX5	coniferin	Estrogen receptor	ESR1
BX5	coniferin	Androgen receptor	AR
BX5	coniferin	Sodium channel protein type 5 subunit alpha	SCN5A
BX5	coniferin	Peroxisome proliferator-activated receptor gamma	PPARG
BX5	coniferin	Prostaglandin G/H synthase 2	PTGS2
BX5	coniferin	Carbonic anhydrase 2	CA2
BX5	coniferin	cGMP-inhibited 3',5'-cyclic phosphodiesterase A	PDE3A

BX5	coniferin	Alpha-1B adrenergic receptor	ADRA1B
BX5	coniferin	Beta-2 adrenergic receptor	ADRB2
BX5	coniferin	Alpha-1D adrenergic receptor	ADRA1D
BX5	coniferin	DNA topoisomerase 2-alpha	TOP2A
BX5	coniferin	Mu-type opioid receptor	OPRM1
BX5	coniferin	Beta-lactamase	DPEP1
BX5	coniferin	Neuronal acetylcholine receptor subunit alpha-7	CHRNA7
BX5	coniferin	Proto-oncogene serine/threonine-protein kinase Pim-1	PIM1
BX5	coniferin	Cyclin-A2	CCNA2
BX5	coniferin	Nuclear receptor coactivator 2	NCOA2
BX5	coniferin	Nuclear receptor coactivator 1	NCOA1
BX6	10,13-eicosadienoic	Prostaglandin G/H synthase 1	PTGS1
BX6	10,13-eicosadienoic	Nuclear receptor coactivator 2	NCOA2
BX7	(3S,6S)-3-(benzyl)-6-(4-hydroxybenzyl)piperazine-2,5-quinone	Androgen receptor	AR
BX7	(3S,6S)-3-(benzyl)-6-(4-hydroxybenzyl)piperazine-2,5-quinone	Prostaglandin G/H synthase 2	PTGS2
BX7	(3S,6S)-3-(benzyl)-6-(4-hydroxybenzyl)piperazine-2,5-quinone	Beta-2 adrenergic receptor	ADRB2
BX8	Cycloartenol	Mineralocorticoid receptor	NR3C2
BX9	beta-D-Ribofuranoside, xanthine-9	Purine nucleoside phosphorylase	PNP
BX9	beta-D-Ribofuranoside, xanthine-9	Prostaglandin G/H synthase 2	PTGS2

Glycyrrhizae

Abbreviation	Molecule Name	Target Name	Genesymbol
GC1	Inermine	Prostaglandin G/H synthase 1	PTGS1
GC1	Inermine	Muscarinic acetylcholine receptor M3	CHRM3
GC1	Inermine	Sodium channel protein type 5 subunit alpha	SCN5A
GC1	Inermine	Prostaglandin G/H synthase 2	PTGS2
GC1	Inermine	Retinoic acid receptor RXR-alpha	RXRA
GC1	Inermine	Alpha-1B adrenergic receptor	ADRA1B
GC1	Inermine	Alpha-1D adrenergic receptor	ADRA1D
GC1	Inermine	Phosphatidylinositol-4,5-bisphosphate 3-kinase catalytic subunit gamma isoform	PIK3CG
GC1	Inermine	cAMP-dependent protein kinase catalytic subunit alpha	PRKACA
GC1	Inermine	Trypsin-1	PRSS1
GC1	Inermine	Muscarinic acetylcholine receptor M1	CHRM1
GC1	Inermine	Beta-2 adrenergic receptor	ADRB2
GC1	Inermine	Mu-type opioid receptor	OPRM1
GC1	Inermine	Heat shock protein HSP 90-alpha	HSP90AA1
GC2	DFV	Prostaglandin G/H synthase 1	PTGS1
GC2	DFV	Estrogen receptor	ESR1
GC2	DFV	Prostaglandin G/H synthase 2	PTGS2
GC2	DFV	Retinoic acid receptor RXR-alpha	RXRA
GC2	DFV	Beta-2 adrenergic receptor	ADRB2
GC2	DFV	Heat shock protein HSP 90-alpha	HSP90AA1
GC2	DFV	Phosphatidylinositol-4,5-bisphosphate 3-kinase catalytic subunit gamma isoform	PIK3CG
GC2	DFV	cAMP-dependent protein kinase catalytic subunit alpha	PRKACA
GC2	DFV	Beta-lactamase	DPEP1
GC2	DFV	Amine oxidase [flavin-containing] B	MAOB
GC2	DFV	Sodium-dependent serotonin transporter	SLC6A4
GC2	DFV	cAMP-dependent protein kinase inhibitor alpha	PKIA
GC85	Mairin	Progesterone receptor	PGR
GC3	Glycyrol	Nitric oxide synthase, inducible	NOS2
GC3	Glycyrol	Estrogen receptor	ESR1
GC3	Glycyrol	Peroxisome proliferator-activated receptor gamma	PPARG
GC3	Glycyrol	Prostaglandin G/H synthase 2	PTGS2
GC3	Glycyrol	Vascular endothelial growth factor receptor 2	KDR
GC3	Glycyrol	Mitogen-activated protein kinase 14	MAPK14
GC3	Glycyrol	Glycogen synthase kinase-3 beta	GSK3B
GC3	Glycyrol	Serine/threonine-protein kinase Chk1	CHEK1
GC3	Glycyrol	Proto-oncogene serine/threonine-protein kinase Pim-1	PIM1
GC3	Glycyrol	Cyclin-A2	CCNA2
GC3	Glycyrol	Prothrombin	F2
GC4	Jaranol	Nitric oxide synthase, inducible	NOS2
GC4	Jaranol	Prostaglandin G/H synthase 1	PTGS1
GC4	Jaranol	Androgen receptor	AR
GC4	Jaranol	Sodium channel protein type 5 subunit alpha	SCN5A
GC4	Jaranol	Prostaglandin G/H synthase 2	PTGS2

GC4	Jaranol	Estrogen receptor beta	ESR2
GC4	Jaranol	Dipeptidyl peptidase 4	DPP4
GC4	Jaranol	Heat shock protein HSP 90-alpha	HSP90AA1
GC4	Jaranol	Serine/threonine-protein kinase Chk1	CHEK1
GC4	Jaranol	Trypsin-1	PRSS1
GC4	Jaranol	Nuclear receptor coactivator 2	NCOA2
GC5	Medicarpin	Nitric oxide synthase, inducible	NOS2
GC5	Medicarpin	Prostaglandin G/H synthase 1	PTGS1
GC5	Medicarpin	D(1A) dopamine receptor	DRD1
GC5	Medicarpin	Muscarinic acetylcholine receptor M3	CHRM3
GC5	Medicarpin	Muscarinic acetylcholine receptor M1	CHRM1
GC5	Medicarpin	Estrogen receptor	ESR1
GC5	Medicarpin	Sodium channel protein type 5 subunit alpha	SCN5A
GC5	Medicarpin	Muscarinic acetylcholine receptor M5	CHRM5
GC5	Medicarpin	Prostaglandin G/H synthase 2	PTGS2
GC5	Medicarpin	Muscarinic acetylcholine receptor M4	CHRM4
GC5	Medicarpin	Retinoic acid receptor RXR-alpha	RXRA
GC5	Medicarpin	5-hydroxytryptamine 2A receptor	HTR2A
GC5	Medicarpin	Alpha-1A adrenergic receptor	ADRA1A
GC5	Medicarpin	Muscarinic acetylcholine receptor M2	CHRM2
GC5	Medicarpin	Alpha-1B adrenergic receptor	ADRA1B
GC5	Medicarpin	Sodium-dependent dopamine transporter	SLC6A3
GC5	Medicarpin	Beta-2 adrenergic receptor	ADRB2
GC5	Medicarpin	Sodium-dependent serotonin transporter	SLC6A4
GC5	Medicarpin	Mu-type opioid receptor	OPRM1
GC5	Medicarpin	Estrogen receptor beta	ESR2
GC5	Medicarpin	Dipeptidyl peptidase 4	DPP4
GC5	Medicarpin	Mitogen-activated protein kinase 10	MAPK10
GC5	Medicarpin	Heat shock protein HSP 90-alpha	HSP90AA1
GC5	Medicarpin	Phosphatidylinositol-4,5-bisphosphate 3-kinase catalytic subunit gamma isoform	PIK3CG
GC5	Medicarpin	Neuronal acetylcholine receptor subunit alpha-7	CHRNA7
GC5	Medicarpin	cAMP-dependent protein kinase catalytic subunit alpha	PRKACA
GC5	Medicarpin	Trypsin-1	PRSS1
GC5	Medicarpin	Proto-oncogene serine/threonine-protein kinase Pim-1	PIM1
GC5	Medicarpin	Cyclin-A2	CCNA2
GC5	Medicarpin	Delta-type opioid receptor	OPRD1
GC5	Medicarpin	cGMP-inhibited 3',5'-cyclic phosphodiesterase A	PDE3A
GC5	Medicarpin	Alpha-1D adrenergic receptor	ADRA1D
GC6	isorhamnetin	Nitric oxide synthase, inducible	NOS2
GC6	isorhamnetin	Prostaglandin G/H synthase 1	PTGS1
GC6	isorhamnetin	Estrogen receptor	ESR1
GC6	isorhamnetin	Androgen receptor	AR
GC6	isorhamnetin	Peroxisome proliferator-activated receptor gamma	PPARG
GC6	isorhamnetin	Prostaglandin G/H synthase 2	PTGS2
GC6	isorhamnetin	Tyrosine-protein phosphatase non-receptor type 1	PTPN1
GC6	isorhamnetin	Estrogen receptor beta	ESR2
GC6	isorhamnetin	Dipeptidyl peptidase 4	DPP4

GC6	isorhamnetin	Mitogen-activated protein kinase 14	MAPK14
GC6	isorhamnetin	Glycogen synthase kinase-3 beta	GSK3B
GC6	isorhamnetin	Heat shock protein HSP 90-alpha	HSP90AA1
GC6	isorhamnetin	Phosphatidylinositol-4,5-bisphosphate 3-kinase catalytic subunit gamma isoform	PIK3CG
GC6	isorhamnetin	cAMP-dependent protein kinase catalytic subunit alpha	PRKACA
GC6	isorhamnetin	Trypsin-1	PRSS1
GC6	isorhamnetin	Proto-oncogene serine/threonine-protein kinase Pim-1	PIM1
GC6	isorhamnetin	Cyclin-A2	CCNA2
GC6	isorhamnetin	Nuclear receptor coactivator 2	NCOA2
GC6	isorhamnetin	Glycogen phosphorylase, muscle form	PYGM
GC6	isorhamnetin	Peroxisome proliferator-activated receptor delta	PPARD
GC6	isorhamnetin	Serine/threonine-protein kinase Chk1	CHEK1
GC6	isorhamnetin	Nuclear receptor coactivator 1	NCOA1
GC6	isorhamnetin	Coagulation factor VII	F7
GC6	isorhamnetin	Prothrombin	F2
GC6	isorhamnetin	Acetylcholinesterase	ACHE
GC6	isorhamnetin	Gamma-aminobutyric-acid receptor subunit alpha-1	GABRA1
GC6	isorhamnetin	Amine oxidase [flavin-containing] B	MAOB
GC6	isorhamnetin	Glutamate receptor 2	GRIA2
GC6	isorhamnetin	Transcription factor p65	RELA
GC6	isorhamnetin	Xanthine dehydrogenase/oxidase	XDH
GC6	isorhamnetin	Neutrophil cytosol factor 1	NCF1
GC6	isorhamnetin	Oxidized low-density lipoprotein receptor 1	OLR1
CJQ	sitosterol	Progesterone receptor	PGR
CJQ	sitosterol	Nuclear receptor coactivator 2	NCOA2
CJQ	sitosterol	Mineralocorticoid receptor	NR3C2
GC7	Lupiwighteone	Nitric oxide synthase, inducible	NOS2
GC7	Lupiwighteone	Prothrombin	F2
GC7	Lupiwighteone	Estrogen receptor	ESR1
GC7	Lupiwighteone	Androgen receptor	AR
GC7	Lupiwighteone	Sodium channel protein type 5 subunit alpha	SCN5A
GC7	Lupiwighteone	Peroxisome proliferator-activated receptor gamma	PPARG
GC7	Lupiwighteone	Coagulation factor X	F10
GC7	Lupiwighteone	Prostaglandin G/H synthase 2	PTGS2
GC7	Lupiwighteone	DNA topoisomerase 2-alpha	TOP2A
GC7	Lupiwighteone	Estrogen receptor beta	ESR2
GC7	Lupiwighteone	Dipeptidyl peptidase 4	DPP4
GC7	Lupiwighteone	Mitogen-activated protein kinase 14	MAPK14
GC7	Lupiwighteone	Glycogen synthase kinase-3 beta	GSK3B
GC7	Lupiwighteone	Heat shock protein HSP 90-alpha	HSP90AA1
GC7	Lupiwighteone	Serine/threonine-protein kinase Chk1	CHEK1
GC7	Lupiwighteone	Trypsin-1	PRSS1
GC7	Lupiwighteone	Proto-oncogene serine/threonine-protein kinase Pim-1	PIM1
GC7	Lupiwighteone	Cyclin-A2	CCNA2
GC7	Lupiwighteone	Nuclear receptor coactivator 2	NCOA2
GC8	7-Methoxy-2-methyl isoflavone	Nitric oxide synthase, inducible	NOS2

GC8	7-Methoxy-2-methyl isoflavone	Prostaglandin G/H synthase 1	PTGS1
GC8	7-Methoxy-2-methyl isoflavone	D(1A) dopamine receptor	DRD1
GC8	7-Methoxy-2-methyl isoflavone	Muscarinic acetylcholine receptor M3	CHRM3
GC8	7-Methoxy-2-methyl isoflavone	Prothrombin	F2
GC8	7-Methoxy-2-methyl isoflavone	Muscarinic acetylcholine receptor M1	CHRM1
GC8	7-Methoxy-2-methyl isoflavone	Estrogen receptor	ESR1
GC8	7-Methoxy-2-methyl isoflavone	Androgen receptor	AR
GC8	7-Methoxy-2-methyl isoflavone	Beta-1 adrenergic receptor	ADRB1
GC8	7-Methoxy-2-methyl isoflavone	Sodium channel protein type 5 subunit alpha	SCN5A
GC8	7-Methoxy-2-methyl isoflavone	Peroxisome proliferator-activated receptor gamma	PPARG
GC8	7-Methoxy-2-methyl isoflavone	Prostaglandin G/H synthase 2	PTGS2
GC8	7-Methoxy-2-methyl isoflavone	Retinoic acid receptor RXR-alpha	RXRA
GC8	7-Methoxy-2-methyl isoflavone	Acetylcholinesterase	ACHE
GC8	7-Methoxy-2-methyl isoflavone	cGMP-inhibited 3',5'-cyclic phosphodiesterase A	PDE3A
GC8	7-Methoxy-2-methyl isoflavone	Alpha-1B adrenergic receptor	ADRA1B
GC8	7-Methoxy-2-methyl isoflavone	Sodium-dependent dopamine transporter	SLC6A3
GC8	7-Methoxy-2-methyl isoflavone	Beta-2 adrenergic receptor	ADRB2
GC8	7-Methoxy-2-methyl isoflavone	Alpha-1D adrenergic receptor	ADRA1D
GC8	7-Methoxy-2-methyl isoflavone	Sodium-dependent serotonin transporter	SLC6A4
GC8	7-Methoxy-2-methyl isoflavone	Estrogen receptor beta	ESR2
GC8	7-Methoxy-2-methyl isoflavone	Gamma-aminobutyric-acid receptor subunit alpha-1	GABRA1
GC8	7-Methoxy-2-methyl isoflavone	Dipeptidyl peptidase 4	DPP4
GC8	7-Methoxy-2-methyl isoflavone	Mitogen-activated protein kinase 14	MAPK14
GC8	7-Methoxy-2-methyl isoflavone	Glycogen synthase kinase-3 beta	GSK3B
GC8	7-Methoxy-2-methyl isoflavone	Heat shock protein HSP 90-alpha	HSP90AA1
GC8	7-Methoxy-2-methyl isoflavone	Leukotriene A-4 hydrolase	LTA4H
GC8	7-Methoxy-2-methyl isoflavone	Amine oxidase [flavin-containing] B	MAOB

GC8	7-Methoxy-2-methyl isoflavone	Neuronal acetylcholine receptor subunit alpha-7	CHRNA7
GC8	7-Methoxy-2-methyl isoflavone	Serine/threonine-protein kinase Chk1	CHEK1
GC8	7-Methoxy-2-methyl isoflavone	cAMP-dependent protein kinase catalytic subunit alpha	PRKACA
GC8	7-Methoxy-2-methyl isoflavone	Trypsin-1	PRSS1
GC8	7-Methoxy-2-methyl isoflavone	Proto-oncogene serine/threonine-protein kinase Pim-1	PIM1
GC8	7-Methoxy-2-methyl isoflavone	Cyclin-A2	CCNA2
GC8	7-Methoxy-2-methyl isoflavone	Nuclear receptor coactivator 1	NCOA1
GC8	7-Methoxy-2-methyl isoflavone	cAMP-dependent protein kinase inhibitor alpha	PKIA
GC8	7-Methoxy-2-methyl isoflavone	Muscarinic acetylcholine receptor M5	CHRM5
GC8	7-Methoxy-2-methyl isoflavone	Mu-type opioid receptor	OPRM1
GC8	7-Methoxy-2-methyl isoflavone	Nuclear receptor coactivator 2	NCOA2
GC9	formononetin	Nitric oxide synthase, inducible	NOS2
GC9	formononetin	Prostaglandin G/H synthase 1	PTGS1
GC9	formononetin	Muscarinic acetylcholine receptor M1	CHRM1
GC9	formononetin	Estrogen receptor	ESR1
GC9	formononetin	Androgen receptor	AR
GC9	formononetin	Peroxisome proliferator-activated receptor gamma	PPARG
GC9	formononetin	Prostaglandin G/H synthase 2	PTGS2
GC9	formononetin	Retinoic acid receptor RXR-alpha	RXRA
GC9	formononetin	cGMP-inhibited 3',5'-cyclic phosphodiesterase A	PDE3A
GC9	formononetin	Alpha-1A adrenergic receptor	ADRA1A
GC9	formononetin	Sodium-dependent dopamine transporter	SLC6A3
GC9	formononetin	Beta-2 adrenergic receptor	ADRB2
GC9	formononetin	Sodium-dependent serotonin transporter	SLC6A4
GC9	formononetin	Estrogen receptor beta	ESR2
GC9	formononetin	Dipeptidyl peptidase 4	DPP4
GC9	formononetin	Mitogen-activated protein kinase 14	MAPK14
GC9	formononetin	Glycogen synthase kinase-3 beta	GSK3B
GC9	formononetin	Heat shock protein HSP 90-alpha	HSP90AA1
GC9	formononetin	Amine oxidase [flavin-containing] B	MAOB
GC9	formononetin	Serine/threonine-protein kinase Chk1	CHEK1
GC9	formononetin	cAMP-dependent protein kinase catalytic subunit alpha	PRKACA
GC9	formononetin	Trypsin-1	PRSS1
GC9	formononetin	Proto-oncogene serine/threonine-protein kinase Pim-1	PIM1
GC9	formononetin	Cyclin-A2	CCNA2
GC9	formononetin	cAMP-dependent protein kinase inhibitor alpha	PKIA
GC9	formononetin	Prothrombin	F2
GC9	formononetin	Acetylcholinesterase	ACHE
GC9	formononetin	Beta-lactamase	DPEP1

GC9	formononetin	Transcription factor AP-1	JUN
GC9	formononetin	Peroxisome proliferator-activated receptor gamma	PPARG
GC9	formononetin	Interleukin-4	IL4
GC9	formononetin	NAD-dependent deacetylase sirtuin-1	SIRT1
GC9	formononetin	ATP synthase subunit beta, mitochondrial	ATP5F1B
GC9	formononetin	NADH-ubiquinone oxidoreductase chain 6	MT-ND6
GC9	formononetin	3 beta-hydroxysteroid dehydrogenase/Delta 5-->4-isomerase type 2	HSD3B2
GC9	formononetin	3 beta-hydroxysteroid dehydrogenase/Delta 5-->4-isomerase type 1	HSD3B1
GC10	Calycosin	Nitric oxide synthase, inducible	NOS2
GC10	Calycosin	Prostaglandin G/H synthase 1	PTGS1
GC10	Calycosin	Estrogen receptor	ESR1
GC10	Calycosin	Androgen receptor	AR
GC10	Calycosin	Peroxisome proliferator-activated receptor gamma	PPARG
GC10	Calycosin	Prostaglandin G/H synthase 2	PTGS2
GC10	Calycosin	Retinoic acid receptor RXR-alpha	RXRA
GC10	Calycosin	cGMP-inhibited 3',5'-cyclic phosphodiesterase A	PDE3A
GC10	Calycosin	Estrogen receptor beta	ESR2
GC10	Calycosin	Dipeptidyl peptidase 4	DPP4
GC10	Calycosin	Mitogen-activated protein kinase 14	MAPK14
GC10	Calycosin	Glycogen synthase kinase-3 beta	GSK3B
GC10	Calycosin	Heat shock protein HSP 90-alpha	HSP90AA1
GC10	Calycosin	Serine/threonine-protein kinase Chk1	CHEK1
GC10	Calycosin	cAMP-dependent protein kinase catalytic subunit alpha	PRKACA
GC10	Calycosin	Trypsin-1	PRSS1
GC10	Calycosin	Proto-oncogene serine/threonine-protein kinase Pim-1	PIM1
GC10	Calycosin	Cyclin-A2	CCNA2
GC10	Calycosin	Nuclear receptor coactivator 2	NCOA2
GC10	Calycosin	Beta-2 adrenergic receptor	ADRB2
GC11	kaempferol	Nitric oxide synthase, inducible	NOS2
GC11	kaempferol	Prostaglandin G/H synthase 1	PTGS1
GC11	kaempferol	Androgen receptor	AR
GC11	kaempferol	Peroxisome proliferator-activated receptor gamma	PPARG
GC11	kaempferol	Prostaglandin G/H synthase 2	PTGS2
GC11	kaempferol	Heat shock protein HSP 90-alpha	HSP90AA1
GC11	kaempferol	Phosphatidylinositol-4,5-bisphosphate 3-kinase catalytic subunit gamma isoform	PIK3CG
GC11	kaempferol	cAMP-dependent protein kinase catalytic subunit alpha	PRKACA
GC11	kaempferol	Nuclear receptor coactivator 2	NCOA2
GC11	kaempferol	Dipeptidyl peptidase 4	DPP4
GC11	kaempferol	Trypsin-1	PRSS1
GC11	kaempferol	Progesterone receptor	PGR
GC11	kaempferol	Prothrombin	F2
GC11	kaempferol	Muscarinic acetylcholine receptor M1	CHRM1
GC11	kaempferol	Gamma-aminobutyric-acid receptor subunit alpha-2	GABRA2
GC11	kaempferol	Acetylcholinesterase	ACHE

GC11	kaempferol	Sodium-dependent noradrenaline transporter	SLC6A2
GC11	kaempferol	Muscarinic acetylcholine receptor M2	CHRM2
GC11	kaempferol	Alpha-1B adrenergic receptor	ADRA1B
GC11	kaempferol	Gamma-aminobutyric-acid receptor subunit alpha-1	GABRA1
GC11	kaempferol	DNA topoisomerase 2-alpha	TOP2A
GC11	kaempferol	Coagulation factor VII	F7
GC11	kaempferol	Transcription factor p65	RELA
GC11	kaempferol	Inhibitor of nuclear factor kappa-B kinase subunit beta	IKBKB
GC11	kaempferol	RAC-alpha serine/threonine-protein kinase	AKT1
GC11	kaempferol	Apoptosis regulator Bcl-2	BCL2
GC11	kaempferol	Apoptosis regulator BAX	BAX
GC11	kaempferol	Tumor necrosis factor	TNF
GC11	kaempferol	Transcription factor AP-1	JUN
GC11	kaempferol	Activator of 90 kDa heat shock protein ATPase homolog 1	AHSA1
GC11	kaempferol	Caspase-3	CASP3
GC11	kaempferol	Mitogen-activated protein kinase 8	MAPK8
GC11	kaempferol	Xanthine dehydrogenase/oxidase	XDH
GC11	kaempferol	Interstitial collagenase	MMP1
GC11	kaempferol	Signal transducer and activator of transcription 1-alpha/beta	STAT1
GC11	kaempferol	Peroxisome proliferator-activated receptor gamma	PPARG
GC11	kaempferol	Heme oxygenase 1	HMOX1
GC11	kaempferol	Cytochrome P450 3A4	CYP3A4
GC11	kaempferol	Cytochrome P450 1A1	CYP1A1
GC11	kaempferol	Intercellular adhesion molecule 1	ICAM1
GC11	kaempferol	E-selectin	SELE
GC11	kaempferol	Vascular cell adhesion protein 1	VCAM1
GC11	kaempferol	Nuclear receptor subfamily 1 group I member 2	NR1I2
GC11	kaempferol	Cytochrome P450 1B1	CYP1B1
GC11	kaempferol	Arachidonate 5-lipoxygenase	ALOX5
GC11	kaempferol	Hyaluronan synthase 2	HAS2
GC11	kaempferol	Aryl hydrocarbon receptor	AHR
GC11	kaempferol	26S proteasome non-ATPase regulatory subunit 3	PSMD3
GC11	kaempferol	Solute carrier family 2, facilitated glucose transporter member 4	SLC2A4
GC11	kaempferol	Nuclear receptor subfamily 1 group I member 3	NR1I3
GC11	kaempferol	Insulin receptor	INSR
GC11	kaempferol	Type I iodothyronine deiodinase	DIO1
GC11	kaempferol	Serine/threonine-protein phosphatase 2B catalytic subunit alpha isoform	PPP3CA
GC11	kaempferol	Glutathione S-transferase Mu 1	GSTM1
GC11	kaempferol	Glutathione S-transferase Mu 2	GSTM2
GC11	kaempferol	Aldo-keto reductase family 1 member C3	AKR1C3
GC11	kaempferol	Antileukoproteinase	SLPI
CS	naringenin	Prostaglandin G/H synthase 1	PTGS1
CS	naringenin	Estrogen receptor	ESR1
CS	naringenin	Prostaglandin G/H synthase 2	PTGS2

CS	naringenin	Heat shock protein HSP 90-alpha	HSP90AA1
CS	naringenin	Beta-lactamase	DPEP1
CS	naringenin	cAMP-dependent protein kinase catalytic subunit alpha	PRKACA
CS	naringenin	Phosphatidylinositol-4,5-bisphosphate 3-kinase catalytic subunit gamma isoform	PIK3CG
CS	naringenin	Transcription factor p65	RELA
CS	naringenin	RAC-alpha serine/threonine-protein kinase	AKT1
CS	naringenin	Apoptosis regulator Bcl-2	BCL2
CS	naringenin	Mitogen-activated protein kinase 3	MAPK3
CS	naringenin	Mitogen-activated protein kinase 1	MAPK1
CS	naringenin	Caspase-3	CASP3
CS	naringenin	Fatty acid synthase	FASN
CS	naringenin	Low-density lipoprotein receptor	LDLR
CS	naringenin	Bcl2 antagonist of cell death	BAD
CS	naringenin	Superoxide dismutase [Cu-Zn]	SOD1
CS	naringenin	Peroxisome proliferator-activated receptor gamma	PPARG
CS	naringenin	Microsomal triglyceride transfer protein large subunit	MTTP
CS	naringenin	Apolipoprotein B-100	APOB
CS	naringenin	Phospholipase B1, membrane-associated	PLB1
CS	naringenin	3-hydroxy-3-methylglutaryl-coenzyme A reductase	HMGCR
CS	naringenin	Cytochrome P450 19A1	CYP19A1
CS	naringenin	UDP-glucuronosyltransferase 1-1	UGT1A1
CS	naringenin	Peroxisome proliferator-activated receptor alpha	PPARA
CS	naringenin	Sterol regulatory element-binding protein 1	SREBF1
CS	naringenin	Glutathione reductase, mitochondrial	GSR
CS	naringenin	Multidrug resistance-associated protein 1	ABCC1
CS	naringenin	Adiponectin	ADIPOQ
CS	naringenin	Sterol O-acyltransferase 2	SOAT2
CS	naringenin	Aldo-keto reductase family 1 member C1	AKR1C1
CS	naringenin	Aspartate aminotransferase, cytoplasmic	GOT1
CS	naringenin	4-aminobutyrate aminotransferase, mitochondrial	ABAT
CS	naringenin	Liver carboxylesterase 1	CES1
CS	naringenin	Sterol O-acyltransferase 1	SOAT1
GC12	(2S)-2-[4-hydroxy-3-(3-methylbut-2-enyl)phenyl]-8,8-dimethyl-2,3-dihydropyrano[2,3-f]chromen-4-one	Nitric oxide synthase, inducible	NOS2
GC12	(2S)-2-[4-hydroxy-3-(3-methylbut-2-enyl)phenyl]-8,8-dimethyl-2,3-dihydropyrano[2,3-f]chromen-4-one	Potassium voltage-gated channel subfamily H member 2	KCNH2

GC12	(2S)-2-[4-hydroxy-3-(3-methylbut-2-enyl)phenyl]-8,8-dimethyl-2,3-dihydropyrano[2,3-f]chromen-4-one	Estrogen receptor	ESR1
GC12	(2S)-2-[4-hydroxy-3-(3-methylbut-2-enyl)phenyl]-8,8-dimethyl-2,3-dihydropyrano[2,3-f]chromen-4-one	Androgen receptor	AR
GC12	(2S)-2-[4-hydroxy-3-(3-methylbut-2-enyl)phenyl]-8,8-dimethyl-2,3-dihydropyrano[2,3-f]chromen-4-one	Peroxisome proliferator-activated receptor gamma	PPARG
GC12	(2S)-2-[4-hydroxy-3-(3-methylbut-2-enyl)phenyl]-8,8-dimethyl-2,3-dihydropyrano[2,3-f]chromen-4-one	Coagulation factor X	F10
GC12	(2S)-2-[4-hydroxy-3-(3-methylbut-2-enyl)phenyl]-8,8-dimethyl-2,3-dihydropyrano[2,3-f]chromen-4-one	Prostaglandin G/H synthase 2	PTGS2
GC12	(2S)-2-[4-hydroxy-3-(3-methylbut-2-enyl)phenyl]-8,8-dimethyl-2,3-dihydropyrano[2,3-f]chromen-4-one	Estrogen receptor beta	ESR2
GC12	(2S)-2-[4-hydroxy-3-(3-methylbut-2-enyl)phenyl]-8,8-dimethyl-2,3-dihydropyrano[2,3-f]chromen-4-one	Mitogen-activated protein kinase 14	MAPK14
GC12	(2S)-2-[4-hydroxy-3-(3-methylbut-2-enyl)phenyl]-8,8-dimethyl-2,3-dihydropyrano[2,3-f]chromen-4-one	Glycogen synthase kinase-3 beta	GSK3B
GC12	(2S)-2-[4-hydroxy-3-(3-methylbut-2-enyl)phenyl]-8,8-dimethyl-2,3-dihydropyrano[2,3-f]chromen-4-one	Proto-oncogene serine/threonine-protein kinase Pim-1	PIM1
GC13	euchrenone	Nitric oxide synthase, inducible	NOS2

GC13	euchrenone	Potassium voltage-gated channel subfamily H member 2	KCNH2
GC13	euchrenone	Estrogen receptor	ESR1
GC13	euchrenone	Sodium channel protein type 5 subunit alpha	SCN5A
GC13	euchrenone	Coagulation factor X	F10
GC13	euchrenone	Prostaglandin G/H synthase 2	PTGS2
GC13	euchrenone	Estrogen receptor beta	ESR2
GC13	euchrenone	Beta-secretase 1	BACE1
GC13	euchrenone	Proto-oncogene serine/threonine-protein kinase Pim-1	PIM1
GC14	glyasperin B	Nitric oxide synthase, inducible	NOS2
GC14	glyasperin B	Prothrombin	F2
GC14	glyasperin B	Estrogen receptor	ESR1
GC14	glyasperin B	Androgen receptor	AR
GC14	glyasperin B	Peroxisome proliferator-activated receptor gamma	PPARG
GC14	glyasperin B	Coagulation factor X	F10
GC14	glyasperin B	Prostaglandin G/H synthase 2	PTGS2
GC14	glyasperin B	Coagulation factor VII	F7
GC14	glyasperin B	Vascular endothelial growth factor receptor 2	KDR
GC14	glyasperin B	Acetylcholinesterase	ACHE
GC14	glyasperin B	DNA topoisomerase 2-alpha	TOP2A
GC14	glyasperin B	Estrogen receptor beta	ESR2
GC14	glyasperin B	Dipeptidyl peptidase 4	DPP4
GC14	glyasperin B	Glycogen synthase kinase-3 beta	GSK3B
GC14	glyasperin B	Heat shock protein HSP 90-alpha	HSP90AA1
GC14	glyasperin B	Trypsin-1	PRSS1
GC14	glyasperin B	Proto-oncogene serine/threonine-protein kinase Pim-1	PIM1
GC14	glyasperin B	Cyclin-A2	CCNA2
GC14	glyasperin B	Nuclear receptor coactivator 2	NCOA2
GC15	glyasperin F	Nitric oxide synthase, inducible	NOS2
GC15	glyasperin F	Prostaglandin G/H synthase 1	PTGS1
GC15	glyasperin F	Estrogen receptor	ESR1
GC15	glyasperin F	Androgen receptor	AR
GC15	glyasperin F	Sodium channel protein type 5 subunit alpha	SCN5A
GC15	glyasperin F	Peroxisome proliferator-activated receptor gamma	PPARG
GC15	glyasperin F	Coagulation factor X	F10
GC15	glyasperin F	Prostaglandin G/H synthase 2	PTGS2
GC15	glyasperin F	DNA topoisomerase 2-alpha	TOP2A
GC15	glyasperin F	Estrogen receptor beta	ESR2
GC15	glyasperin F	Mitogen-activated protein kinase 14	MAPK14
GC15	glyasperin F	Glycogen synthase kinase-3 beta	GSK3B
GC15	glyasperin F	Heat shock protein HSP 90-alpha	HSP90AA1
GC15	glyasperin F	Trypsin-1	PRSS1
GC15	glyasperin F	Proto-oncogene serine/threonine-protein kinase Pim-1	PIM1
GC15	glyasperin F	Cyclin-A2	CCNA2
GC16	Glyasperin C	Nitric oxide synthase, inducible	NOS2
GC16	Glyasperin C	Prothrombin	F2

GC16	Glyasperin C	Potassium voltage-gated channel subfamily H member 2	KCNH2
GC16	Glyasperin C	Estrogen receptor	ESR1
GC16	Glyasperin C	Androgen receptor	AR
GC16	Glyasperin C	Sodium channel protein type 5 subunit alpha	SCN5A
GC16	Glyasperin C	Peroxisome proliferator-activated receptor gamma	PPARG
GC16	Glyasperin C	Coagulation factor X	F10
GC16	Glyasperin C	Prostaglandin G/H synthase 2	PTGS2
GC16	Glyasperin C	Retinoic acid receptor RXR-alpha	RXRA
GC16	Glyasperin C	Acetylcholinesterase	ACHE
GC16	Glyasperin C	DNA topoisomerase 2-alpha	TOP2A
GC16	Glyasperin C	Estrogen receptor beta	ESR2
GC16	Glyasperin C	Dipeptidyl peptidase 4	DPP4
GC16	Glyasperin C	Mitogen-activated protein kinase 14	MAPK14
GC16	Glyasperin C	Glycogen synthase kinase-3 beta	GSK3B
GC16	Glyasperin C	Heat shock protein HSP 90-alpha	HSP90AA1
GC16	Glyasperin C	Serine/threonine-protein kinase Chk1	CHEK1
GC16	Glyasperin C	Trypsin-1	PRSS1
GC16	Glyasperin C	Proto-oncogene serine/threonine-protein kinase Pim-1	PIM1
GC16	Glyasperin C	Cyclin-A2	CCNA2
GC16	Glyasperin C	Nuclear receptor coactivator 2	NCOA2
GC17	Isotrifoliol	Nitric oxide synthase, inducible	NOS2
GC17	Isotrifoliol	Estrogen receptor	ESR1
GC17	Isotrifoliol	Androgen receptor	AR
GC17	Isotrifoliol	Prostaglandin G/H synthase 2	PTGS2
GC17	Isotrifoliol	Estrogen receptor beta	ESR2
GC17	Isotrifoliol	Mitogen-activated protein kinase 14	MAPK14
GC17	Isotrifoliol	Glycogen synthase kinase-3 beta	GSK3B
GC17	Isotrifoliol	Heat shock protein HSP 90-alpha	HSP90AA1
GC17	Isotrifoliol	Phosphatidylinositol-4,5-bisphosphate 3-kinase catalytic subunit gamma isoform	PIK3CG
GC17	Isotrifoliol	Serine/threonine-protein kinase Chk1	CHEK1
GC17	Isotrifoliol	cAMP-dependent protein kinase catalytic subunit alpha	PRKACA
GC17	Isotrifoliol	Proto-oncogene serine/threonine-protein kinase Pim-1	PIM1
GC17	Isotrifoliol	Cyclin-A2	CCNA2
GC18	(E)-1-(2,4-dihydroxyphenyl)-3-(2,2-dimethylchromen-6-yl)prop-2-en-1-one	Nitric oxide synthase, inducible	NOS2
GC18	(E)-1-(2,4-dihydroxyphenyl)-3-(2,2-dimethylchromen-6-yl)prop-2-en-1-one	Prostaglandin G/H synthase 1	PTGS1

GC18	(E)-1-(2,4-dihydroxyphenyl)-3-(2,2-dimethylchromen-6-yl)prop-2-en-1-one	Estrogen receptor	ESR1
GC18	(E)-1-(2,4-dihydroxyphenyl)-3-(2,2-dimethylchromen-6-yl)prop-2-en-1-one	Androgen receptor	AR
GC18	(E)-1-(2,4-dihydroxyphenyl)-3-(2,2-dimethylchromen-6-yl)prop-2-en-1-one	Sodium channel protein type 5 subunit alpha	SCN5A
GC18	(E)-1-(2,4-dihydroxyphenyl)-3-(2,2-dimethylchromen-6-yl)prop-2-en-1-one	Peroxisome proliferator-activated receptor gamma	PPARG
GC18	(E)-1-(2,4-dihydroxyphenyl)-3-(2,2-dimethylchromen-6-yl)prop-2-en-1-one	Coagulation factor X	F10
GC18	(E)-1-(2,4-dihydroxyphenyl)-3-(2,2-dimethylchromen-6-yl)prop-2-en-1-one	Prostaglandin G/H synthase 2	PTGS2
GC18	(E)-1-(2,4-dihydroxyphenyl)-3-(2,2-dimethylchromen-6-yl)prop-2-en-1-one	Carbonic anhydrase 2	CA2
GC18	(E)-1-(2,4-dihydroxyphenyl)-3-(2,2-dimethylchromen-6-yl)prop-2-en-1-one	Retinoic acid receptor RXR-alpha	RXRA
GC18	(E)-1-(2,4-dihydroxyphenyl)-3-(2,2-dimethylchromen-6-yl)prop-2-en-1-one	Alpha-1B adrenergic receptor	ADRA1B

GC18	(E)-1-(2,4-dihydroxyphenyl)-3-(2,2-dimethylchromen-6-yl)prop-2-en-1-one	Estrogen receptor beta	ESR2
GC18	(E)-1-(2,4-dihydroxyphenyl)-3-(2,2-dimethylchromen-6-yl)prop-2-en-1-one	Mitogen-activated protein kinase 14	MAPK14
GC18	(E)-1-(2,4-dihydroxyphenyl)-3-(2,2-dimethylchromen-6-yl)prop-2-en-1-one	Glycogen synthase kinase-3 beta	GSK3B
GC18	(E)-1-(2,4-dihydroxyphenyl)-3-(2,2-dimethylchromen-6-yl)prop-2-en-1-one	Serine/threonine-protein kinase Chk1	CHEK1
GC18	(E)-1-(2,4-dihydroxyphenyl)-3-(2,2-dimethylchromen-6-yl)prop-2-en-1-one	Proto-oncogene serine/threonine-protein kinase Pim-1	PIM1
GC18	(E)-1-(2,4-dihydroxyphenyl)-3-(2,2-dimethylchromen-6-yl)prop-2-en-1-one	Cyclin-A2	CCNA2
GC18	(E)-1-(2,4-dihydroxyphenyl)-3-(2,2-dimethylchromen-6-yl)prop-2-en-1-one	Nuclear receptor coactivator 2	NCOA2
GC19	kanzonols W	Nitric oxide synthase, inducible	NOS2
GC19	kanzonols W	Prostaglandin G/H synthase 1	PTGS1
GC19	kanzonols W	Estrogen receptor	ESR1
GC19	kanzonols W	Androgen receptor	AR
GC19	kanzonols W	Sodium channel protein type 5 subunit alpha	SCN5A
GC19	kanzonols W	Peroxisome proliferator-activated receptor gamma	PPARG
GC19	kanzonols W	Coagulation factor X	F10
GC19	kanzonols W	Prostaglandin G/H synthase 2	PTGS2
GC19	kanzonols W	Retinoic acid receptor RXR-alpha	RXRA
GC19	kanzonols W	DNA topoisomerase 2-alpha	TOP2A
GC19	kanzonols W	Estrogen receptor beta	ESR2

GC19	kanzonols W	Mitogen-activated protein kinase 14	MAPK14
GC19	kanzonols W	Glycogen synthase kinase-3 beta	GSK3B
GC19	kanzonols W	Serine/threonine-protein kinase Chk1	CHEK1
GC19	kanzonols W	Trypsin-1	PRSS1
GC19	kanzonols W	Proto-oncogene serine/threonine-protein kinase Pim-1	PIM1
GC19	kanzonols W	Cyclin-A2	CCNA2
GC19	kanzonols W	Nuclear receptor coactivator 2	NCOA2
GC19	kanzonols W	Nuclear receptor coactivator 1	NCOA1
GC20	(2S)-6-(2,4-dihydroxyphenyl)-2-(2-hydroxypropan-2-yl)-4-methoxy-2,3-dihydrofuro[3,2-g]chromen-7-one	Nitric oxide synthase, inducible	NOS2
GC20	(2S)-6-(2,4-dihydroxyphenyl)-2-(2-hydroxypropan-2-yl)-4-methoxy-2,3-dihydrofuro[3,2-g]chromen-7-one	Prothrombin	F2
GC20	(2S)-6-(2,4-dihydroxyphenyl)-2-(2-hydroxypropan-2-yl)-4-methoxy-2,3-dihydrofuro[3,2-g]chromen-7-one	Estrogen receptor	ESR1
GC20	(2S)-6-(2,4-dihydroxyphenyl)-2-(2-hydroxypropan-2-yl)-4-methoxy-2,3-dihydrofuro[3,2-g]chromen-7-one	Androgen receptor	AR
GC20	(2S)-6-(2,4-dihydroxyphenyl)-2-(2-hydroxypropan-2-yl)-4-methoxy-2,3-dihydrofuro[3,2-g]chromen-7-one	Peroxisome proliferator-activated receptor gamma	PPARG
GC20	(2S)-6-(2,4-dihydroxyphenyl)-2-(2-hydroxypropan-2-yl)-4-methoxy-2,3-dihydrofuro[3,2-g]chromen-7-one	Coagulation factor X	F10

GC20	(2S)-6-(2,4-dihydroxyphenyl)-2-(2-hydroxypropan-2-yl)-4-methoxy-2,3-dihydrofuro[3,2-g]chromen-7-one	Prostaglandin G/H synthase 2	PTGS2
GC20	(2S)-6-(2,4-dihydroxyphenyl)-2-(2-hydroxypropan-2-yl)-4-methoxy-2,3-dihydrofuro[3,2-g]chromen-7-one	Coagulation factor VII	F7
GC20	(2S)-6-(2,4-dihydroxyphenyl)-2-(2-hydroxypropan-2-yl)-4-methoxy-2,3-dihydrofuro[3,2-g]chromen-7-one	Vascular endothelial growth factor receptor 2	KDR
GC20	(2S)-6-(2,4-dihydroxyphenyl)-2-(2-hydroxypropan-2-yl)-4-methoxy-2,3-dihydrofuro[3,2-g]chromen-7-one	Acetylcholinesterase	ACHE
GC20	(2S)-6-(2,4-dihydroxyphenyl)-2-(2-hydroxypropan-2-yl)-4-methoxy-2,3-dihydrofuro[3,2-g]chromen-7-one	DNA topoisomerase 2-alpha	TOP2A
GC20	(2S)-6-(2,4-dihydroxyphenyl)-2-(2-hydroxypropan-2-yl)-4-methoxy-2,3-dihydrofuro[3,2-g]chromen-7-one	Estrogen receptor beta	ESR2
GC20	(2S)-6-(2,4-dihydroxyphenyl)-2-(2-hydroxypropan-2-yl)-4-methoxy-2,3-dihydrofuro[3,2-g]chromen-7-one	Dipeptidyl peptidase 4	DPP4

GC20	(2S)-6-(2,4-dihydroxyphenyl)-2-(2-hydroxypropan-2-yl)-4-methoxy-2,3-dihydrofuro[3,2-g]chromen-7-one	Mitogen-activated protein kinase 14	MAPK14
GC20	(2S)-6-(2,4-dihydroxyphenyl)-2-(2-hydroxypropan-2-yl)-4-methoxy-2,3-dihydrofuro[3,2-g]chromen-7-one	Glycogen synthase kinase-3 beta	GSK3B
GC20	(2S)-6-(2,4-dihydroxyphenyl)-2-(2-hydroxypropan-2-yl)-4-methoxy-2,3-dihydrofuro[3,2-g]chromen-7-one	Serine/threonine-protein kinase Chk1	CHEK1
GC20	(2S)-6-(2,4-dihydroxyphenyl)-2-(2-hydroxypropan-2-yl)-4-methoxy-2,3-dihydrofuro[3,2-g]chromen-7-one	Trypsin-1	PRSS1
GC20	(2S)-6-(2,4-dihydroxyphenyl)-2-(2-hydroxypropan-2-yl)-4-methoxy-2,3-dihydrofuro[3,2-g]chromen-7-one	Proto-oncogene serine/threonine-protein kinase Pim-1	PIM1
GC20	(2S)-6-(2,4-dihydroxyphenyl)-2-(2-hydroxypropan-2-yl)-4-methoxy-2,3-dihydrofuro[3,2-g]chromen-7-one	Cyclin-A2	CCNA2
GC21	Semilicoisoflavone B	Nitric oxide synthase, inducible	NOS2
GC21	Semilicoisoflavone B	Prothrombin	F2
GC21	Semilicoisoflavone B	Estrogen receptor	ESR1
GC21	Semilicoisoflavone B	Androgen receptor	AR
GC21	Semilicoisoflavone B	Sodium channel protein type 5 subunit alpha	SCN5A
GC21	Semilicoisoflavone B	Peroxisome proliferator-activated receptor gamma	PPARG

GC21	Semilicoisoflavone B	Coagulation factor X	F10
GC21	Semilicoisoflavone B	Prostaglandin G/H synthase 2	PTGS2
GC21	Semilicoisoflavone B	Coagulation factor VII	F7
GC21	Semilicoisoflavone B	Acetylcholinesterase	ACHE
GC21	Semilicoisoflavone B	DNA topoisomerase 2-alpha	TOP2A
GC21	Semilicoisoflavone B	Glycogen synthase kinase-3 beta	GSK3B
GC21	Semilicoisoflavone B	Heat shock protein HSP 90-alpha	HSP90AA1
GC21	Semilicoisoflavone B	Serine/threonine-protein kinase Chk1	CHEK1
GC21	Semilicoisoflavone B	Trypsin-1	PRSS1
GC22	Glepidotin A	Nitric oxide synthase, inducible	NOS2
GC22	Glepidotin A	Prostaglandin G/H synthase 1	PTGS1
GC22	Glepidotin A	Prothrombin	F2
GC22	Glepidotin A	Estrogen receptor	ESR1
GC22	Glepidotin A	Androgen receptor	AR
GC22	Glepidotin A	Sodium channel protein type 5 subunit alpha	SCN5A
GC22	Glepidotin A	Peroxisome proliferator-activated receptor gamma	PPARG
GC22	Glepidotin A	Coagulation factor X	F10
GC22	Glepidotin A	Prostaglandin G/H synthase 2	PTGS2
GC22	Glepidotin A	Coagulation factor VII	F7
GC22	Glepidotin A	Vascular endothelial growth factor receptor 2	KDR
GC22	Glepidotin A	Retinoic acid receptor RXR-alpha	RXRA
GC22	Glepidotin A	cGMP-inhibited 3',5'-cyclic phosphodiesterase A	PDE3A
GC22	Glepidotin A	DNA topoisomerase 2-alpha	TOP2A
GC22	Glepidotin A	Dipeptidyl peptidase 4	DPP4
GC22	Glepidotin A	Mitogen-activated protein kinase 14	MAPK14
GC22	Glepidotin A	Glycogen synthase kinase-3 beta	GSK3B
GC22	Glepidotin A	Heat shock protein HSP 90-alpha	HSP90AA1
GC22	Glepidotin A	Serine/threonine-protein kinase Chk1	CHEK1
GC22	Glepidotin A	Trypsin-1	PRSS1
GC22	Glepidotin A	Proto-oncogene serine/threonine-protein kinase Pim-1	PIM1
GC22	Glepidotin A	Cyclin-A2	CCNA2
GC23	Glepidotin B	Prostaglandin G/H synthase 1	PTGS1
GC23	Glepidotin B	Estrogen receptor	ESR1
GC23	Glepidotin B	Sodium channel protein type 5 subunit alpha	SCN5A
GC23	Glepidotin B	Coagulation factor X	F10
GC23	Glepidotin B	Prostaglandin G/H synthase 2	PTGS2
GC23	Glepidotin B	Coagulation factor VII	F7
GC23	Glepidotin B	Retinoic acid receptor RXR-alpha	RXRA
GC23	Glepidotin B	cGMP-inhibited 3',5'-cyclic phosphodiesterase A	PDE3A

GC23	Glepidotin B	Alpha-1B adrenergic receptor	ADRA1B
GC23	Glepidotin B	DNA topoisomerase 2-alpha	TOP2A
GC23	Glepidotin B	Heat shock protein HSP 90-alpha	HSP90AA1
GC23	Glepidotin B	Nuclear receptor coactivator 1	NCOA1
GC24	Phaseolinisoflavan	Nitric oxide synthase, inducible	NOS2
GC24	Phaseolinisoflavan	Muscarinic acetylcholine receptor M1	CHRM1
GC24	Phaseolinisoflavan	Estrogen receptor	ESR1
GC24	Phaseolinisoflavan	Androgen receptor	AR
GC24	Phaseolinisoflavan	Sodium channel protein type 5 subunit alpha	SCN5A
GC24	Phaseolinisoflavan	Peroxisome proliferator-activated receptor gamma	PPARG
GC24	Phaseolinisoflavan	Coagulation factor X	F10
GC24	Phaseolinisoflavan	Prostaglandin G/H synthase 2	PTGS2
GC24	Phaseolinisoflavan	Retinoic acid receptor RXR-alpha	RXRA
GC24	Phaseolinisoflavan	Acetylcholinesterase	ACHE
GC24	Phaseolinisoflavan	Alpha-1B adrenergic receptor	ADRA1B
GC24	Phaseolinisoflavan	Beta-2 adrenergic receptor	ADRB2
GC24	Phaseolinisoflavan	Estrogen receptor beta	ESR2
GC24	Phaseolinisoflavan	Mitogen-activated protein kinase 14	MAPK14
GC24	Phaseolinisoflavan	Glycogen synthase kinase-3 beta	GSK3B
GC24	Phaseolinisoflavan	Serine/threonine-protein kinase Chk1	CHEK1
GC24	Phaseolinisoflavan	Trypsin-1	PRSS1
GC24	Phaseolinisoflavan	Proto-oncogene serine/threonine-protein kinase Pim-1	PIM1
GC24	Phaseolinisoflavan	Cyclin-A2	CCNA2
GC24	Phaseolinisoflavan	Nuclear receptor coactivator 1	NCOA1
GC25	Glypallichalcone	Nitric oxide synthase, inducible	NOS2
GC25	Glypallichalcone	Prostaglandin G/H synthase 1	PTGS1
GC25	Glypallichalcone	Muscarinic acetylcholine receptor M1	CHRM1
GC25	Glypallichalcone	Estrogen receptor	ESR1
GC25	Glypallichalcone	Androgen receptor	AR
GC25	Glypallichalcone	Sodium channel protein type 5 subunit alpha	SCN5A
GC25	Glypallichalcone	Peroxisome proliferator-activated receptor gamma	PPARG
GC25	Glypallichalcone	Prostaglandin G/H synthase 2	PTGS2
GC25	Glypallichalcone	Carbonic anhydrase 2	CA2
GC25	Glypallichalcone	cGMP-inhibited 3',5'-cyclic phosphodiesterase A	PDE3A
GC25	Glypallichalcone	Alpha-1B adrenergic receptor	ADRA1B
GC25	Glypallichalcone	Sodium-dependent dopamine transporter	SLC6A3
GC25	Glypallichalcone	Beta-2 adrenergic receptor	ADRB2
GC25	Glypallichalcone	Sodium-dependent serotonin transporter	SLC6A4
GC25	Glypallichalcone	Estrogen receptor beta	ESR2
GC25	Glypallichalcone	Mitogen-activated protein kinase 14	MAPK14
GC25	Glypallichalcone	Glycogen synthase kinase-3 beta	GSK3B
GC25	Glypallichalcone	Heat shock protein HSP 90-alpha	HSP90AA1
GC25	Glypallichalcone	Leukotriene A-4 hydrolase	LTA4H
GC25	Glypallichalcone	Amine oxidase [flavin-containing] B	MAOB
GC25	Glypallichalcone	Serine/threonine-protein kinase Chk1	CHEK1
GC25	Glypallichalcone	cAMP-dependent protein kinase catalytic subunit alpha	PRKACA
GC25	Glypallichalcone	Cyclin-A2	CCNA2
GC25	Glypallichalcone	Nuclear receptor coactivator 1	NCOA1

GC25	Glypallichalcone	cAMP-dependent protein kinase inhibitor alpha	PKIA
GC26	8-(6-hydroxy-2-benzofuranyl)-2,2-dimethyl-5-chromenol	Nitric oxide synthase, inducible	NOS2
GC26	8-(6-hydroxy-2-benzofuranyl)-2,2-dimethyl-5-chromenol	Estrogen receptor	ESR1
GC26	8-(6-hydroxy-2-benzofuranyl)-2,2-dimethyl-5-chromenol	Prostaglandin G/H synthase 2	PTGS2
GC26	8-(6-hydroxy-2-benzofuranyl)-2,2-dimethyl-5-chromenol	Retinoic acid receptor RXR-alpha	RXRA
GC26	8-(6-hydroxy-2-benzofuranyl)-2,2-dimethyl-5-chromenol	Heat shock protein HSP 90-alpha	HSP90AA1
GC26	8-(6-hydroxy-2-benzofuranyl)-2,2-dimethyl-5-chromenol	Phosphatidylinositol-4,5-bisphosphate 3-kinase catalytic subunit gamma isoform	PIK3CG
GC27	Licochalcone B	Nitric oxide synthase, inducible	NOS2
GC27	Licochalcone B	Prostaglandin G/H synthase 1	PTGS1
GC27	Licochalcone B	Estrogen receptor	ESR1
GC27	Licochalcone B	Androgen receptor	AR
GC27	Licochalcone B	Peroxisome proliferator-activated receptor gamma	PPARG
GC27	Licochalcone B	Prostaglandin G/H synthase 2	PTGS2
GC27	Licochalcone B	Carbonic anhydrase 2	CA2
GC27	Licochalcone B	cGMP-inhibited 3',5'-cyclic phosphodiesterase A	PDE3A
GC27	Licochalcone B	Beta-2 adrenergic receptor	ADRB2
GC27	Licochalcone B	Estrogen receptor beta	ESR2
GC27	Licochalcone B	Mitogen-activated protein kinase 14	MAPK14
GC27	Licochalcone B	Glycogen synthase kinase-3 beta	GSK3B
GC27	Licochalcone B	Heat shock protein HSP 90-alpha	HSP90AA1
GC27	Licochalcone B	Serine/threonine-protein kinase Chk1	CHEK1
GC27	Licochalcone B	cAMP-dependent protein kinase catalytic subunit alpha	PRKACA
GC27	Licochalcone B	Proto-oncogene serine/threonine-protein kinase Pim-1	PIM1
GC27	Licochalcone B	Cyclin-A2	CCNA2
GC28	licochalcone G	Nitric oxide synthase, inducible	NOS2
GC28	licochalcone G	Estrogen receptor	ESR1
GC28	licochalcone G	Androgen receptor	AR
GC28	licochalcone G	Peroxisome proliferator-activated receptor gamma	PPARG
GC28	licochalcone G	Coagulation factor X	F10
GC28	licochalcone G	Prostaglandin G/H synthase 2	PTGS2

GC28	licochalcone G	Vascular endothelial growth factor receptor 2	KDR
GC28	licochalcone G	Estrogen receptor beta	ESR2
GC28	licochalcone G	Mitogen-activated protein kinase 14	MAPK14
GC28	licochalcone G	Glycogen synthase kinase-3 beta	GSK3B
GC28	licochalcone G	Heat shock protein HSP 90-alpha	HSP90AA1
GC28	licochalcone G	Proto-oncogene serine/threonine-protein kinase Pim-1	PIM1
GC28	licochalcone G	Cyclin-A2	CCNA2
GC28	licochalcone G	Nuclear receptor coactivator 2	NCOA2
GC29	3-(2,4-dihydroxyphenyl)-8-(1,1-dimethylprop-2-enyl)-7-hydroxy-5-methoxy-coumarin	Nitric oxide synthase, inducible	NOS2
GC29	3-(2,4-dihydroxyphenyl)-8-(1,1-dimethylprop-2-enyl)-7-hydroxy-5-methoxy-coumarin	Prothrombin	F2
GC29	3-(2,4-dihydroxyphenyl)-8-(1,1-dimethylprop-2-enyl)-7-hydroxy-5-methoxy-coumarin	Potassium voltage-gated channel subfamily H member 2	KCNH2
GC29	3-(2,4-dihydroxyphenyl)-8-(1,1-dimethylprop-2-enyl)-7-hydroxy-5-methoxy-coumarin	Estrogen receptor	ESR1
GC29	3-(2,4-dihydroxyphenyl)-8-(1,1-dimethylprop-2-enyl)-7-hydroxy-5-methoxy-coumarin	Androgen receptor	AR
GC29	3-(2,4-dihydroxyphenyl)-8-(1,1-dimethylprop-2-enyl)-7-hydroxy-5-methoxy-coumarin	Peroxisome proliferator-activated receptor gamma	PPARG
GC29	3-(2,4-dihydroxyphenyl)-8-(1,1-dimethylprop-2-enyl)-7-hydroxy-5-methoxy-coumarin	Coagulation factor X	F10

GC29	3-(2,4-dihydroxyphenyl)-8-(1,1-dimethylprop-2-enyl)-7-hydroxy-5-methoxy-coumarin	Prostaglandin G/H synthase 2	PTGS2
GC29	3-(2,4-dihydroxyphenyl)-8-(1,1-dimethylprop-2-enyl)-7-hydroxy-5-methoxy-coumarin	Coagulation factor VII	F7
GC29	3-(2,4-dihydroxyphenyl)-8-(1,1-dimethylprop-2-enyl)-7-hydroxy-5-methoxy-coumarin	Vascular endothelial growth factor receptor 2	KDR
GC29	3-(2,4-dihydroxyphenyl)-8-(1,1-dimethylprop-2-enyl)-7-hydroxy-5-methoxy-coumarin	DNA topoisomerase 2-alpha	TOP2A
GC29	3-(2,4-dihydroxyphenyl)-8-(1,1-dimethylprop-2-enyl)-7-hydroxy-5-methoxy-coumarin	Estrogen receptor beta	ESR2
GC29	3-(2,4-dihydroxyphenyl)-8-(1,1-dimethylprop-2-enyl)-7-hydroxy-5-methoxy-coumarin	Dipeptidyl peptidase 4	DPP4
GC29	3-(2,4-dihydroxyphenyl)-8-(1,1-dimethylprop-2-enyl)-7-hydroxy-5-methoxy-coumarin	Mitogen-activated protein kinase 14	MAPK14
GC29	3-(2,4-dihydroxyphenyl)-8-(1,1-dimethylprop-2-enyl)-7-hydroxy-5-methoxy-coumarin	Glycogen synthase kinase-3 beta	GSK3B
GC29	3-(2,4-dihydroxyphenyl)-8-(1,1-dimethylprop-2-enyl)-7-hydroxy-5-methoxy-coumarin	Heat shock protein HSP 90-alpha	HSP90AA1

GC29	3-(2,4-dihydroxyphenyl)-8-(1,1-dimethylprop-2-enyl)-7-hydroxy-5-methoxy-coumarin	Serine/threonine-protein kinase Chk1	CHEK1
GC29	3-(2,4-dihydroxyphenyl)-8-(1,1-dimethylprop-2-enyl)-7-hydroxy-5-methoxy-coumarin	Trypsin-1	PRSS1
GC29	3-(2,4-dihydroxyphenyl)-8-(1,1-dimethylprop-2-enyl)-7-hydroxy-5-methoxy-coumarin	Proto-oncogene serine/threonine-protein kinase Pim-1	PIM1
GC29	3-(2,4-dihydroxyphenyl)-8-(1,1-dimethylprop-2-enyl)-7-hydroxy-5-methoxy-coumarin	Nuclear receptor coactivator 2	NCOA2
GC29	3-(2,4-dihydroxyphenyl)-8-(1,1-dimethylprop-2-enyl)-7-hydroxy-5-methoxy-coumarin	Nuclear receptor coactivator 1	NCOA1
GC30	Licoricone	Nitric oxide synthase, inducible	NOS2
GC30	Licoricone	Prothrombin	F2
GC30	Licoricone	Potassium voltage-gated channel subfamily H member 2	KCNH2
GC30	Licoricone	Estrogen receptor	ESR1
GC30	Licoricone	Androgen receptor	AR
GC30	Licoricone	Peroxisome proliferator-activated receptor gamma	PPARG
GC30	Licoricone	Coagulation factor X	F10
GC30	Licoricone	Prostaglandin G/H synthase 2	PTGS2
GC30	Licoricone	Vascular endothelial growth factor receptor 2	KDR
GC30	Licoricone	DNA topoisomerase 2-alpha	TOP2A
GC30	Licoricone	Serine/threonine-protein kinase Chk1	CHEK1
GC30	Licoricone	Trypsin-1	PRSS1
GC30	Licoricone	Proto-oncogene serine/threonine-protein kinase Pim-1	PIM1
GC30	Licoricone	Nuclear receptor coactivator 2	NCOA2
GC31	Gancaonin A	Nitric oxide synthase, inducible	NOS2
GC31	Gancaonin A	Prothrombin	F2
GC31	Gancaonin A	Estrogen receptor	ESR1
GC31	Gancaonin A	Androgen receptor	AR
GC31	Gancaonin A	Sodium channel protein type 5 subunit alpha	SCN5A

GC31	Gancaonin A	Peroxisome proliferator-activated receptor gamma	PPARG
GC31	Gancaonin A	Coagulation factor X	F10
GC31	Gancaonin A	Prostaglandin G/H synthase 2	PTGS2
GC31	Gancaonin A	Acetylcholinesterase	ACHE
GC31	Gancaonin A	DNA topoisomerase 2-alpha	TOP2A
GC31	Gancaonin A	Estrogen receptor beta	ESR2
GC31	Gancaonin A	Dipeptidyl peptidase 4	DPP4
GC31	Gancaonin A	Glycogen synthase kinase-3 beta	GSK3B
GC31	Gancaonin A	Heat shock protein HSP 90-alpha	HSP90AA1
GC31	Gancaonin A	Serine/threonine-protein kinase Chk1	CHEK1
GC31	Gancaonin A	Trypsin-1	PRSS1
GC31	Gancaonin A	Proto-oncogene serine/threonine-protein kinase Pim-1	PIM1
GC31	Gancaonin A	Cyclin-A2	CCNA2
GC31	Gancaonin A	Nuclear receptor coactivator 2	NCOA2
GC32	Gancaonin B	Nitric oxide synthase, inducible	NOS2
GC32	Gancaonin B	Prothrombin	F2
GC32	Gancaonin B	Estrogen receptor	ESR1
GC32	Gancaonin B	Androgen receptor	AR
GC32	Gancaonin B	Peroxisome proliferator-activated receptor gamma	PPARG
GC32	Gancaonin B	Coagulation factor X	F10
GC32	Gancaonin B	Prostaglandin G/H synthase 2	PTGS2
GC32	Gancaonin B	Coagulation factor VII	F7
GC32	Gancaonin B	Vascular endothelial growth factor receptor 2	KDR
GC32	Gancaonin B	Alpha-1B adrenergic receptor	ADRA1B
GC32	Gancaonin B	Beta-2 adrenergic receptor	ADRB2
GC32	Gancaonin B	DNA topoisomerase 2-alpha	TOP2A
GC32	Gancaonin B	Estrogen receptor beta	ESR2
GC32	Gancaonin B	Dipeptidyl peptidase 4	DPP4
GC32	Gancaonin B	Glycogen synthase kinase-3 beta	GSK3B
GC32	Gancaonin B	Heat shock protein HSP 90-alpha	HSP90AA1
GC32	Gancaonin B	Serine/threonine-protein kinase Chk1	CHEK1
GC32	Gancaonin B	Trypsin-1	PRSS1
GC32	Gancaonin B	Proto-oncogene serine/threonine-protein kinase Pim-1	PIM1
GC32	Gancaonin B	Cyclin-A2	CCNA2
GC32	Gancaonin B	Nuclear receptor coactivator 2	NCOA2
GC33	3-(3,4-dihydroxyphenyl)-5,7-dihydroxy-8-(3-methylbut-2-enyl)chromone	Nitric oxide synthase, inducible	NOS2
GC33	3-(3,4-dihydroxyphenyl)-5,7-dihydroxy-8-(3-methylbut-2-enyl)chromone	Prothrombin	F2
GC33	3-(3,4-dihydroxyphenyl)-5,7-dihydroxy-8-(3-methylbut-2-enyl)chromone	Estrogen receptor	ESR1

GC33	3-(3,4-dihydroxyphenyl)-5,7-dihydroxy-8-(3-methylbut-2-enyl)chromone	Androgen receptor	AR
GC33	3-(3,4-dihydroxyphenyl)-5,7-dihydroxy-8-(3-methylbut-2-enyl)chromone	Peroxisome proliferator-activated receptor gamma	PPARG
GC33	3-(3,4-dihydroxyphenyl)-5,7-dihydroxy-8-(3-methylbut-2-enyl)chromone	Coagulation factor X	F10
GC33	3-(3,4-dihydroxyphenyl)-5,7-dihydroxy-8-(3-methylbut-2-enyl)chromone	Prostaglandin G/H synthase 2	PTGS2
GC33	3-(3,4-dihydroxyphenyl)-5,7-dihydroxy-8-(3-methylbut-2-enyl)chromone	Tyrosine-protein phosphatase non-receptor type 1	PTPN1
GC33	3-(3,4-dihydroxyphenyl)-5,7-dihydroxy-8-(3-methylbut-2-enyl)chromone	Mitogen-activated protein kinase 14	MAPK14
GC33	3-(3,4-dihydroxyphenyl)-5,7-dihydroxy-8-(3-methylbut-2-enyl)chromone	Glycogen synthase kinase-3 beta	GSK3B
GC33	3-(3,4-dihydroxyphenyl)-5,7-dihydroxy-8-(3-methylbut-2-enyl)chromone	Heat shock protein HSP 90-alpha	HSP90AA1
GC33	3-(3,4-dihydroxyphenyl)-5,7-dihydroxy-8-(3-methylbut-2-enyl)chromone	Serine/threonine-protein kinase Chk1	CHEK1
GC33	3-(3,4-dihydroxyphenyl)-5,7-dihydroxy-8-(3-methylbut-2-enyl)chromone	Trypsin-1	PRSS1
GC33	3-(3,4-dihydroxyphenyl)-5,7-dihydroxy-8-(3-methylbut-2-enyl)chromone	Proto-oncogene serine/threonine-protein kinase Pim-1	PIM1

GC33	3-(3,4-dihydroxyphenyl)-5,7-dihydroxy-8-(3-methylbut-2-enyl)chromone	Cyclin-A2	CCNA2
GC33	3-(3,4-dihydroxyphenyl)-5,7-dihydroxy-8-(3-methylbut-2-enyl)chromone	Nuclear receptor coactivator 2	NCOA2
GC34	5,7-dihydroxy-3-(4-methoxyphenyl)-8-(3-methylbut-2-enyl)chromone	Nitric oxide synthase, inducible	NOS2
GC34	5,7-dihydroxy-3-(4-methoxyphenyl)-8-(3-methylbut-2-enyl)chromone	Potassium voltage-gated channel subfamily H member 2	KCNH2
GC34	5,7-dihydroxy-3-(4-methoxyphenyl)-8-(3-methylbut-2-enyl)chromone	Estrogen receptor	ESR1
GC34	5,7-dihydroxy-3-(4-methoxyphenyl)-8-(3-methylbut-2-enyl)chromone	Androgen receptor	AR
GC34	5,7-dihydroxy-3-(4-methoxyphenyl)-8-(3-methylbut-2-enyl)chromone	Peroxisome proliferator-activated receptor gamma	PPARG
GC34	5,7-dihydroxy-3-(4-methoxyphenyl)-8-(3-methylbut-2-enyl)chromone	Coagulation factor X	F10
GC34	5,7-dihydroxy-3-(4-methoxyphenyl)-8-(3-methylbut-2-enyl)chromone	Prostaglandin G/H synthase 2	PTGS2
GC34	5,7-dihydroxy-3-(4-methoxyphenyl)-8-(3-methylbut-2-enyl)chromone	DNA topoisomerase 2-alpha	TOP2A
GC34	5,7-dihydroxy-3-(4-methoxyphenyl)-8-(3-methylbut-2-enyl)chromone	Estrogen receptor beta	ESR2

GC34	5,7-dihydroxy-3-(4-methoxyphenyl)-8-(3-methylbut-2-enyl)chromone	Dipeptidyl peptidase 4	DPP4
GC34	5,7-dihydroxy-3-(4-methoxyphenyl)-8-(3-methylbut-2-enyl)chromone	Mitogen-activated protein kinase 14	MAPK14
GC34	5,7-dihydroxy-3-(4-methoxyphenyl)-8-(3-methylbut-2-enyl)chromone	Glycogen synthase kinase-3 beta	GSK3B
GC34	5,7-dihydroxy-3-(4-methoxyphenyl)-8-(3-methylbut-2-enyl)chromone	Heat shock protein HSP 90-alpha	HSP90AA1
GC34	5,7-dihydroxy-3-(4-methoxyphenyl)-8-(3-methylbut-2-enyl)chromone	Serine/threonine-protein kinase Chk1	CHEK1
GC34	5,7-dihydroxy-3-(4-methoxyphenyl)-8-(3-methylbut-2-enyl)chromone	Trypsin-1	PRSS1
GC34	5,7-dihydroxy-3-(4-methoxyphenyl)-8-(3-methylbut-2-enyl)chromone	Proto-oncogene serine/threonine-protein kinase Pim-1	PIM1
GC34	5,7-dihydroxy-3-(4-methoxyphenyl)-8-(3-methylbut-2-enyl)chromone	Cyclin-A2	CCNA2
GC34	5,7-dihydroxy-3-(4-methoxyphenyl)-8-(3-methylbut-2-enyl)chromone	Nuclear receptor coactivator 2	NCOA2
GC35	2-(3,4-dihydroxyphenyl)-5,7-dihydroxy-6-(3-methylbut-2-enyl)chromone	Prothrombin	F2
GC35	2-(3,4-dihydroxyphenyl)-5,7-dihydroxy-6-(3-methylbut-2-enyl)chromone	Androgen receptor	AR

GC35	2-(3,4-dihydroxyphenyl)-5,7-dihydroxy-6-(3-methylbut-2-enyl)chromone	Sodium channel protein type 5 subunit alpha	SCN5A
GC35	2-(3,4-dihydroxyphenyl)-5,7-dihydroxy-6-(3-methylbut-2-enyl)chromone	Peroxisome proliferator-activated receptor gamma	PPARG
GC35	2-(3,4-dihydroxyphenyl)-5,7-dihydroxy-6-(3-methylbut-2-enyl)chromone	Coagulation factor X	F10
GC35	2-(3,4-dihydroxyphenyl)-5,7-dihydroxy-6-(3-methylbut-2-enyl)chromone	Prostaglandin G/H synthase 2	PTGS2
GC35	2-(3,4-dihydroxyphenyl)-5,7-dihydroxy-6-(3-methylbut-2-enyl)chromone	Coagulation factor VII	F7
GC35	2-(3,4-dihydroxyphenyl)-5,7-dihydroxy-6-(3-methylbut-2-enyl)chromone	Beta-2 adrenergic receptor	ADRB2
GC35	2-(3,4-dihydroxyphenyl)-5,7-dihydroxy-6-(3-methylbut-2-enyl)chromone	Dipeptidyl peptidase 4	DPP4
GC35	2-(3,4-dihydroxyphenyl)-5,7-dihydroxy-6-(3-methylbut-2-enyl)chromone	Heat shock protein HSP 90-alpha	HSP90AA1
GC35	2-(3,4-dihydroxyphenyl)-5,7-dihydroxy-6-(3-methylbut-2-enyl)chromone	Serine/threonine-protein kinase Chk1	CHEK1
GC35	2-(3,4-dihydroxyphenyl)-5,7-dihydroxy-6-(3-methylbut-2-enyl)chromone	Trypsin-1	PRSS1
GC35	2-(3,4-dihydroxyphenyl)-5,7-dihydroxy-6-(3-methylbut-2-enyl)chromone	Proto-oncogene serine/threonine-protein kinase Pim-1	PIM1

GC35	2-(3,4-dihydroxyphenyl)-5,7-dihydroxy-6-(3-methylbut-2-enyl)chromone	Cyclin-A2	CCNA2
GC36	Glycyrin	Nitric oxide synthase, inducible	NOS2
GC36	Glycyrin	Prothrombin	F2
GC36	Glycyrin	Potassium voltage-gated channel subfamily H member 2	KCNH2
GC36	Glycyrin	Estrogen receptor	ESR1
GC36	Glycyrin	Androgen receptor	AR
GC36	Glycyrin	Peroxisome proliferator-activated receptor gamma	PPARG
GC36	Glycyrin	Coagulation factor X	F10
GC36	Glycyrin	Prostaglandin G/H synthase 2	PTGS2
GC36	Glycyrin	Vascular endothelial growth factor receptor 2	KDR
GC36	Glycyrin	DNA topoisomerase 2-alpha	TOP2A
GC36	Glycyrin	Estrogen receptor beta	ESR2
GC36	Glycyrin	Dipeptidyl peptidase 4	DPP4
GC36	Glycyrin	Serine/threonine-protein kinase Chk1	CHEK1
GC36	Glycyrin	Trypsin-1	PRSS1
GC36	Glycyrin	Proto-oncogene serine/threonine-protein kinase Pim-1	PIM1
GC36	Glycyrin	Nuclear receptor coactivator 2	NCOA2
GC37	Licocoumarone	Estrogen receptor	ESR1
GC37	Licocoumarone	Androgen receptor	AR
GC37	Licocoumarone	Estrogen receptor beta	ESR2
GC37	Licocoumarone	Glycogen synthase kinase-3 beta	GSK3B
GC37	Licocoumarone	Heat shock protein HSP 90-alpha	HSP90AA1
GC37	Licocoumarone	Cyclin-A2	CCNA2
GC38	Licoisoflavone	Nitric oxide synthase, inducible	NOS2
GC38	Licoisoflavone	Prothrombin	F2
GC38	Licoisoflavone	Estrogen receptor	ESR1
GC38	Licoisoflavone	Androgen receptor	AR
GC38	Licoisoflavone	Peroxisome proliferator-activated receptor gamma	PPARG
GC38	Licoisoflavone	Coagulation factor X	F10
GC38	Licoisoflavone	Prostaglandin G/H synthase 2	PTGS2
GC38	Licoisoflavone	Vascular endothelial growth factor receptor 2	KDR
GC38	Licoisoflavone	DNA topoisomerase 2-alpha	TOP2A
GC38	Licoisoflavone	Dipeptidyl peptidase 4	DPP4
GC38	Licoisoflavone	Mitogen-activated protein kinase 14	MAPK14
GC38	Licoisoflavone	Heat shock protein HSP 90-alpha	HSP90AA1
GC38	Licoisoflavone	Serine/threonine-protein kinase Chk1	CHEK1
GC38	Licoisoflavone	Trypsin-1	PRSS1
GC38	Licoisoflavone	Proto-oncogene serine/threonine-protein kinase Pim-1	PIM1
GC38	Licoisoflavone	Cyclin-A2	CCNA2
GC38	Licoisoflavone	Nuclear receptor coactivator 2	NCOA2
GC39	Licoisoflavone B	Nitric oxide synthase, inducible	NOS2
GC39	Licoisoflavone B	Prothrombin	F2
GC39	Licoisoflavone B	Estrogen receptor	ESR1
GC39	Licoisoflavone B	Androgen receptor	AR

GC39	Licoisoflavone B	Peroxisome proliferator-activated receptor gamma	PPARG
GC39	Licoisoflavone B	Coagulation factor X	F10
GC39	Licoisoflavone B	Prostaglandin G/H synthase 2	PTGS2
GC39	Licoisoflavone B	Acetylcholinesterase	ACHE
GC39	Licoisoflavone B	DNA topoisomerase 2-alpha	TOP2A
GC39	Licoisoflavone B	Estrogen receptor beta	ESR2
GC39	Licoisoflavone B	Glycogen synthase kinase-3 beta	GSK3B
GC39	Licoisoflavone B	Serine/threonine-protein kinase Chk1	CHEK1
GC39	Licoisoflavone B	Trypsin-1	PRSS1
GC39	Licoisoflavone B	Proto-oncogene serine/threonine-protein kinase Pim-1	PIM1
GC39	Licoisoflavone B	Cyclin-A2	CCNA2
GC40	licoisoflavanone	Nitric oxide synthase, inducible	NOS2
GC40	licoisoflavanone	Prostaglandin G/H synthase 1	PTGS1
GC40	licoisoflavanone	Estrogen receptor	ESR1
GC40	licoisoflavanone	Androgen receptor	AR
GC40	licoisoflavanone	Sodium channel protein type 5 subunit alpha	SCN5A
GC40	licoisoflavanone	Peroxisome proliferator-activated receptor gamma	PPARG
GC40	licoisoflavanone	Coagulation factor X	F10
GC40	licoisoflavanone	Prostaglandin G/H synthase 2	PTGS2
GC40	licoisoflavanone	Coagulation factor VII	F7
GC40	licoisoflavanone	Acetylcholinesterase	ACHE
GC40	licoisoflavanone	DNA topoisomerase 2-alpha	TOP2A
GC40	licoisoflavanone	Estrogen receptor beta	ESR2
GC40	licoisoflavanone	Glycogen synthase kinase-3 beta	GSK3B
GC40	licoisoflavanone	Heat shock protein HSP 90-alpha	HSP90AA1
GC40	licoisoflavanone	Trypsin-1	PRSS1
GC40	licoisoflavanone	Proto-oncogene serine/threonine-protein kinase Pim-1	PIM1
GC40	licoisoflavanone	Cyclin-A2	CCNA2
GC40	licoisoflavanone	Nuclear receptor coactivator 1	NCOA1
GC41	shinpterocarpin	Nitric oxide synthase, inducible	NOS2
GC41	shinpterocarpin	Prostaglandin G/H synthase 1	PTGS1
GC41	shinpterocarpin	Muscarinic acetylcholine receptor M3	CHRM3
GC41	shinpterocarpin	Potassium voltage-gated channel subfamily H member 2	KCNH2
GC41	shinpterocarpin	Muscarinic acetylcholine receptor M1	CHRM1
GC41	shinpterocarpin	Estrogen receptor	ESR1
GC41	shinpterocarpin	Androgen receptor	AR
GC41	shinpterocarpin	Sodium channel protein type 5 subunit alpha	SCN5A
GC41	shinpterocarpin	Peroxisome proliferator-activated receptor gamma	PPARG
GC41	shinpterocarpin	Prostaglandin G/H synthase 2	PTGS2
GC41	shinpterocarpin	Retinoic acid receptor RXR-alpha	RXRA
GC41	shinpterocarpin	Delta-type opioid receptor	OPRD1
GC41	shinpterocarpin	Alpha-1B adrenergic receptor	ADRA1B
GC41	shinpterocarpin	Beta-2 adrenergic receptor	ADRB2
GC41	shinpterocarpin	Alpha-1D adrenergic receptor	ADRA1D
GC41	shinpterocarpin	Mu-type opioid receptor	OPRM1
GC41	shinpterocarpin	Estrogen receptor beta	ESR2
GC41	shinpterocarpin	Mitogen-activated protein kinase 14	MAPK14

GC41	shinpterocarpin	Glycogen synthase kinase-3 beta	GSK3B
GC41	shinpterocarpin	Phosphatidylinositol-4,5-bisphosphate 3-kinase catalytic subunit gamma isoform	PIK3CG
GC41	shinpterocarpin	Neuronal acetylcholine receptor subunit alpha-7	CHRNA7
GC41	shinpterocarpin	cAMP-dependent protein kinase catalytic subunit alpha	PRKACA
GC41	shinpterocarpin	Trypsin-1	PRSS1
GC41	shinpterocarpin	Proto-oncogene serine/threonine-protein kinase Pim-1	PIM1
GC41	shinpterocarpin	Cyclin-A2	CCNA2
GC41	shinpterocarpin	Nuclear receptor coactivator 1	NCOA1
GC42	(E)-3-[3,4-dihydroxy-5-(3-methylbut-2-enyl)phenyl]-1-(2,4-dihydroxyphenyl)prop-2-en-1-one	Estrogen receptor	ESR1
GC42	(E)-3-[3,4-dihydroxy-5-(3-methylbut-2-enyl)phenyl]-1-(2,4-dihydroxyphenyl)prop-2-en-1-one	Androgen receptor	AR
GC42	(E)-3-[3,4-dihydroxy-5-(3-methylbut-2-enyl)phenyl]-1-(2,4-dihydroxyphenyl)prop-2-en-1-one	Peroxisome proliferator-activated receptor gamma	PPARG
GC42	(E)-3-[3,4-dihydroxy-5-(3-methylbut-2-enyl)phenyl]-1-(2,4-dihydroxyphenyl)prop-2-en-1-one	Prostaglandin G/H synthase 2	PTGS2
GC42	(E)-3-[3,4-dihydroxy-5-(3-methylbut-2-enyl)phenyl]-1-(2,4-dihydroxyphenyl)prop-2-en-1-one	Mitogen-activated protein kinase 14	MAPK14
GC42	(E)-3-[3,4-dihydroxy-5-(3-methylbut-2-enyl)phenyl]-1-(2,4-dihydroxyphenyl)prop-2-en-1-one	Glycogen synthase kinase-3 beta	GSK3B

GC42	(E)-3-[3,4-dihydroxy-5-(3-methylbut-2-enyl)phenyl]-1-(2,4-dihydroxyphenyl)prop-2-en-1-one	Heat shock protein HSP 90-alpha	HSP90AA1
GC42	(E)-3-[3,4-dihydroxy-5-(3-methylbut-2-enyl)phenyl]-1-(2,4-dihydroxyphenyl)prop-2-en-1-one	Proto-oncogene serine/threonine-protein kinase Pim-1	PIM1
GC42	(E)-3-[3,4-dihydroxy-5-(3-methylbut-2-enyl)phenyl]-1-(2,4-dihydroxyphenyl)prop-2-en-1-one	Cyclin-A2	CCNA2
GC42	(E)-3-[3,4-dihydroxy-5-(3-methylbut-2-enyl)phenyl]-1-(2,4-dihydroxyphenyl)prop-2-en-1-one	Nuclear receptor coactivator 2	NCOA2
GC43	liquiritin	Coagulation factor X	F10
GC43	liquiritin	Coagulation factor VII	F7
GC43	liquiritin	Prostaglandin G/H synthase 2	PTGS2
GC43	liquiritin	Vascular endothelial growth factor receptor 2	KDR
GC43	liquiritin	Superoxide dismutase [Cu-Zn]	SOD1
GC44	licopyranocoumarin	Nitric oxide synthase, inducible	NOS2
GC44	licopyranocoumarin	Prothrombin	F2
GC44	licopyranocoumarin	Estrogen receptor	ESR1
GC44	licopyranocoumarin	Androgen receptor	AR
GC44	licopyranocoumarin	Peroxisome proliferator-activated receptor gamma	PPARG
GC44	licopyranocoumarin	Coagulation factor X	F10
GC44	licopyranocoumarin	Prostaglandin G/H synthase 2	PTGS2
GC44	licopyranocoumarin	Coagulation factor VII	F7
GC44	licopyranocoumarin	Vascular endothelial growth factor receptor 2	KDR
GC44	licopyranocoumarin	Acetylcholinesterase	ACHE
GC44	licopyranocoumarin	DNA topoisomerase 2-alpha	TOP2A
GC44	licopyranocoumarin	Trypsin-1	PRSS1
GC44	licopyranocoumarin	Proto-oncogene serine/threonine-protein kinase Pim-1	PIM1
GC44	licopyranocoumarin	Cyclin-A2	CCNA2
GC45	Glyzaglabrin	Nitric oxide synthase, inducible	NOS2
GC45	Glyzaglabrin	Prostaglandin G/H synthase 1	PTGS1
GC45	Glyzaglabrin	Estrogen receptor	ESR1
GC45	Glyzaglabrin	Androgen receptor	AR

GC45	Glyzaglabrin	Peroxisome proliferator-activated receptor gamma	PPARG
GC45	Glyzaglabrin	Prostaglandin G/H synthase 2	PTGS2
GC45	Glyzaglabrin	Estrogen receptor beta	ESR2
GC45	Glyzaglabrin	Dipeptidyl peptidase 4	DPP4
GC45	Glyzaglabrin	Mitogen-activated protein kinase 14	MAPK14
GC45	Glyzaglabrin	Glycogen synthase kinase-3 beta	GSK3B
GC45	Glyzaglabrin	Heat shock protein HSP 90-alpha	HSP90AA1
GC45	Glyzaglabrin	Phosphatidylinositol-4,5-bisphosphate 3-kinase catalytic subunit gamma isoform	PIK3CG
GC45	Glyzaglabrin	Serine/threonine-protein kinase Chk1	CHEK1
GC45	Glyzaglabrin	cAMP-dependent protein kinase catalytic subunit alpha	PRKACA
GC45	Glyzaglabrin	Trypsin-1	PRSS1
GC45	Glyzaglabrin	Proto-oncogene serine/threonine-protein kinase Pim-1	PIM1
GC45	Glyzaglabrin	Cyclin-A2	CCNA2
GC46	Glabridin	Nitric oxide synthase, inducible	NOS2
GC46	Glabridin	Muscarinic acetylcholine receptor M1	CHRM1
GC46	Glabridin	Estrogen receptor	ESR1
GC46	Glabridin	Androgen receptor	AR
GC46	Glabridin	Sodium channel protein type 5 subunit alpha	SCN5A
GC46	Glabridin	Peroxisome proliferator-activated receptor gamma	PPARG
GC46	Glabridin	Prostaglandin G/H synthase 2	PTGS2
GC46	Glabridin	Retinoic acid receptor RXR-alpha	RXRA
GC46	Glabridin	Acetylcholinesterase	ACHE
GC46	Glabridin	Alpha-1B adrenergic receptor	ADRA1B
GC46	Glabridin	Beta-2 adrenergic receptor	ADRB2
GC46	Glabridin	Estrogen receptor beta	ESR2
GC46	Glabridin	Mitogen-activated protein kinase 14	MAPK14
GC46	Glabridin	Glycogen synthase kinase-3 beta	GSK3B
GC46	Glabridin	Serine/threonine-protein kinase Chk1	CHEK1
GC46	Glabridin	cAMP-dependent protein kinase catalytic subunit alpha	PRKACA
GC46	Glabridin	Trypsin-1	PRSS1
GC46	Glabridin	Proto-oncogene serine/threonine-protein kinase Pim-1	PIM1
GC46	Glabridin	Cyclin-A2	CCNA2
GC46	Glabridin	Nuclear receptor coactivator 2	NCOA2
GC46	Glabridin	Nuclear receptor coactivator 1	NCOA1
GC47	Glabranin	Nitric oxide synthase, inducible	NOS2
GC47	Glabranin	Prostaglandin G/H synthase 1	PTGS1
GC47	Glabranin	Estrogen receptor	ESR1
GC47	Glabranin	Sodium channel protein type 5 subunit alpha	SCN5A
GC47	Glabranin	Coagulation factor X	F10
GC47	Glabranin	Prostaglandin G/H synthase 2	PTGS2
GC47	Glabranin	cGMP-inhibited 3',5'-cyclic phosphodiesterase A	PDE3A
GC47	Glabranin	Heat shock protein HSP 90-alpha	HSP90AA1
GC47	Glabranin	cAMP-dependent protein kinase catalytic subunit alpha	PRKACA
GC48	Glabrene	Nitric oxide synthase, inducible	NOS2

GC48	Glabrene	Prostaglandin G/H synthase 1	PTGS1
GC48	Glabrene	Estrogen receptor	ESR1
GC48	Glabrene	Androgen receptor	AR
GC48	Glabrene	Sodium channel protein type 5 subunit alpha	SCN5A
GC48	Glabrene	Peroxisome proliferator-activated receptor gamma	PPARG
GC48	Glabrene	Coagulation factor X	F10
GC48	Glabrene	Prostaglandin G/H synthase 2	PTGS2
GC48	Glabrene	Retinoic acid receptor RXR-alpha	RXRA
GC48	Glabrene	Beta-2 adrenergic receptor	ADRB2
GC48	Glabrene	Estrogen receptor beta	ESR2
GC48	Glabrene	Mitogen-activated protein kinase 14	MAPK14
GC48	Glabrene	Glycogen synthase kinase-3 beta	GSK3B
GC48	Glabrene	Heat shock protein HSP 90-alpha	HSP90AA1
GC48	Glabrene	Trypsin-1	PRSS1
GC48	Glabrene	Proto-oncogene serine/threonine-protein kinase Pim-1	PIM1
GC48	Glabrene	Nuclear receptor coactivator 2	NCOA2
GC49	Glabrone	Nitric oxide synthase, inducible	NOS2
GC49	Glabrone	Prostaglandin G/H synthase 1	PTGS1
GC49	Glabrone	Prothrombin	F2
GC49	Glabrone	Estrogen receptor	ESR1
GC49	Glabrone	Androgen receptor	AR
GC49	Glabrone	Sodium channel protein type 5 subunit alpha	SCN5A
GC49	Glabrone	Peroxisome proliferator-activated receptor gamma	PPARG
GC49	Glabrone	Coagulation factor X	F10
GC49	Glabrone	Prostaglandin G/H synthase 2	PTGS2
GC49	Glabrone	Retinoic acid receptor RXR-alpha	RXRA
GC49	Glabrone	Acetylcholinesterase	ACHE
GC49	Glabrone	Estrogen receptor beta	ESR2
GC49	Glabrone	Dipeptidyl peptidase 4	DPP4
GC49	Glabrone	Mitogen-activated protein kinase 14	MAPK14
GC49	Glabrone	Glycogen synthase kinase-3 beta	GSK3B
GC49	Glabrone	Serine/threonine-protein kinase Chk1	CHEK1
GC49	Glabrone	Trypsin-1	PRSS1
GC49	Glabrone	Proto-oncogene serine/threonine-protein kinase Pim-1	PIM1
GC49	Glabrone	Cyclin-A2	CCNA2
GC50	1,3-dihydroxy-9-methoxy-6-benzofurano[3,2-c]chromenone	Estrogen receptor	ESR1
GC50	1,3-dihydroxy-9-methoxy-6-benzofurano[3,2-c]chromenone	Peroxisome proliferator-activated receptor gamma	PPARG
GC50	1,3-dihydroxy-9-methoxy-6-benzofurano[3,2-c]chromenone	Estrogen receptor beta	ESR2

GC50	1,3-dihydroxy-9-methoxy-6-benzofurano[3,2-c]chromenone	Mitogen-activated protein kinase 14	MAPK14
GC50	1,3-dihydroxy-9-methoxy-6-benzofurano[3,2-c]chromenone	Glycogen synthase kinase-3 beta	GSK3B
GC50	1,3-dihydroxy-9-methoxy-6-benzofurano[3,2-c]chromenone	Heat shock protein HSP 90-alpha	HSP90AA1
GC50	1,3-dihydroxy-9-methoxy-6-benzofurano[3,2-c]chromenone	Serine/threonine-protein kinase Chk1	CHEK1
GC50	1,3-dihydroxy-9-methoxy-6-benzofurano[3,2-c]chromenone	cAMP-dependent protein kinase catalytic subunit alpha	PRKACA
GC50	1,3-dihydroxy-9-methoxy-6-benzofurano[3,2-c]chromenone	Cyclin-A2	CCNA2
GC51	1,3-dihydroxy-8,9-dimethoxy-6-benzofurano[3,2-c]chromenone	Estrogen receptor	ESR1
GC51	1,3-dihydroxy-8,9-dimethoxy-6-benzofurano[3,2-c]chromenone	Androgen receptor	AR
GC51	1,3-dihydroxy-8,9-dimethoxy-6-benzofurano[3,2-c]chromenone	Peroxisome proliferator-activated receptor gamma	PPARG
GC51	1,3-dihydroxy-8,9-dimethoxy-6-benzofurano[3,2-c]chromenone	Mitogen-activated protein kinase 14	MAPK14
GC51	1,3-dihydroxy-8,9-dimethoxy-6-benzofurano[3,2-c]chromenone	Glycogen synthase kinase-3 beta	GSK3B
GC51	1,3-dihydroxy-8,9-dimethoxy-6-benzofurano[3,2-c]chromenone	Heat shock protein HSP 90-alpha	HSP90AA1
GC51	1,3-dihydroxy-8,9-dimethoxy-6-benzofurano[3,2-c]chromenone	Serine/threonine-protein kinase Chk1	CHEK1

GC51	1,3-dihydroxy-8,9-dimethoxy-6-benzofurano[3,2-c]chromenone	cAMP-dependent protein kinase catalytic subunit alpha	PRKACA
GC52	Eurycarpin A	Nitric oxide synthase, inducible	NOS2
GC52	Eurycarpin A	Prothrombin	F2
GC52	Eurycarpin A	Estrogen receptor	ESR1
GC52	Eurycarpin A	Androgen receptor	AR
GC52	Eurycarpin A	Sodium channel protein type 5 subunit alpha	SCN5A
GC52	Eurycarpin A	Peroxisome proliferator-activated receptor gamma	PPARG
GC52	Eurycarpin A	Coagulation factor X	F10
GC52	Eurycarpin A	Prostaglandin G/H synthase 2	PTGS2
GC52	Eurycarpin A	Estrogen receptor beta	ESR2
GC52	Eurycarpin A	Dipeptidyl peptidase 4	DPP4
GC52	Eurycarpin A	Mitogen-activated protein kinase 14	MAPK14
GC52	Eurycarpin A	Glycogen synthase kinase-3 beta	GSK3B
GC52	Eurycarpin A	Heat shock protein HSP 90-alpha	HSP90AA1
GC52	Eurycarpin A	Serine/threonine-protein kinase Chk1	CHEK1
GC52	Eurycarpin A	Trypsin-1	PRSS1
GC52	Eurycarpin A	Proto-oncogene serine/threonine-protein kinase Pim-1	PIM1
GC52	Eurycarpin A	Cyclin-A2	CCNA2
GC53	(-)-Medicocarpin	Prostaglandin G/H synthase 2	PTGS2
GC53	(-)-Medicocarpin	Acetylcholinesterase	ACHE
GC54	Sigmoidin-B	Estrogen receptor	ESR1
GC54	Sigmoidin-B	Coagulation factor X	F10
GC54	Sigmoidin-B	Prostaglandin G/H synthase 2	PTGS2
GC54	Sigmoidin-B	Vascular endothelial growth factor receptor 2	KDR
GC54	Sigmoidin-B	Heat shock protein HSP 90-alpha	HSP90AA1
GC55	(2R)-7-hydroxy-2-(4-hydroxyphenyl)chroman-4-one	Prostaglandin G/H synthase 1	PTGS1
GC55	(2R)-7-hydroxy-2-(4-hydroxyphenyl)chroman-4-one	Estrogen receptor	ESR1
GC55	(2R)-7-hydroxy-2-(4-hydroxyphenyl)chroman-4-one	Prostaglandin G/H synthase 2	PTGS2
GC55	(2R)-7-hydroxy-2-(4-hydroxyphenyl)chroman-4-one	Retinoic acid receptor RXR-alpha	RXRA
GC55	(2R)-7-hydroxy-2-(4-hydroxyphenyl)chroman-4-one	cGMP-inhibited 3',5'-cyclic phosphodiesterase A	PDE3A

GC55	(2R)-7-hydroxy-2-(4-hydroxyphenyl)chroman-4-one	Beta-2 adrenergic receptor	ADRB2
GC55	(2R)-7-hydroxy-2-(4-hydroxyphenyl)chroman-4-one	Heat shock protein HSP 90-alpha	HSP90AA1
GC55	(2R)-7-hydroxy-2-(4-hydroxyphenyl)chroman-4-one	Phosphatidylinositol-4,5-bisphosphate 3-kinase catalytic subunit gamma isoform	PIK3CG
GC55	(2R)-7-hydroxy-2-(4-hydroxyphenyl)chroman-4-one	Beta-lactamase	DPEP1
GC55	(2R)-7-hydroxy-2-(4-hydroxyphenyl)chroman-4-one	Amine oxidase [flavin-containing] B	MAOB
GC55	(2R)-7-hydroxy-2-(4-hydroxyphenyl)chroman-4-one	cAMP-dependent protein kinase catalytic subunit alpha	PRKACA
GC55	(2R)-7-hydroxy-2-(4-hydroxyphenyl)chroman-4-one	cAMP-dependent protein kinase inhibitor alpha	PKIA
GC55	(2R)-7-hydroxy-2-(4-hydroxyphenyl)chroman-4-one	Gamma-aminobutyric-acid receptor subunit alpha-1	GABRA1
GC55	(2R)-7-hydroxy-2-(4-hydroxyphenyl)chroman-4-one	Sodium-dependent serotonin transporter	SLC6A4
GC56	(2S)-7-hydroxy-2-(4-hydroxyphenyl)-8-(3-methylbut-2-enyl)chroman-4-one	Nitric oxide synthase, inducible	NOS2
GC56	(2S)-7-hydroxy-2-(4-hydroxyphenyl)-8-(3-methylbut-2-enyl)chroman-4-one	Prostaglandin G/H synthase 1	PTGS1
GC56	(2S)-7-hydroxy-2-(4-hydroxyphenyl)-8-(3-methylbut-2-enyl)chroman-4-one	Estrogen receptor	ESR1

GC56	(2S)-7-hydroxy-2-(4-hydroxyphenyl)-8-(3-methylbut-2-enyl)chroman-4-one	Sodium channel protein type 5 subunit alpha	SCN5A
GC56	(2S)-7-hydroxy-2-(4-hydroxyphenyl)-8-(3-methylbut-2-enyl)chroman-4-one	Coagulation factor X	F10
GC56	(2S)-7-hydroxy-2-(4-hydroxyphenyl)-8-(3-methylbut-2-enyl)chroman-4-one	Prostaglandin G/H synthase 2	PTGS2
GC56	(2S)-7-hydroxy-2-(4-hydroxyphenyl)-8-(3-methylbut-2-enyl)chroman-4-one	cGMP-inhibited 3',5'-cyclic phosphodiesterase A	PDE3A
GC56	(2S)-7-hydroxy-2-(4-hydroxyphenyl)-8-(3-methylbut-2-enyl)chroman-4-one	Alpha-1B adrenergic receptor	ADRA1B
GC56	(2S)-7-hydroxy-2-(4-hydroxyphenyl)-8-(3-methylbut-2-enyl)chroman-4-one	Beta-2 adrenergic receptor	ADRB2
GC56	(2S)-7-hydroxy-2-(4-hydroxyphenyl)-8-(3-methylbut-2-enyl)chroman-4-one	Estrogen receptor beta	ESR2
GC56	(2S)-7-hydroxy-2-(4-hydroxyphenyl)-8-(3-methylbut-2-enyl)chroman-4-one	Heat shock protein HSP 90-alpha	HSP90AA1
GC57	Isoglycyrol	Nitric oxide synthase, inducible	NOS2
GC57	Isoglycyrol	Estrogen receptor	ESR1
GC57	Isoglycyrol	Androgen receptor	AR
GC57	Isoglycyrol	Prostaglandin G/H synthase 2	PTGS2
GC57	Isoglycyrol	Dipeptidyl peptidase 4	DPP4
GC57	Isoglycyrol	Glycogen synthase kinase-3 beta	GSK3B
GC57	Isoglycyrol	Proto-oncogene serine/threonine-protein kinase Pim-1	PIM1
GC58	Isolicoflavonol	Nitric oxide synthase, inducible	NOS2
GC58	Isolicoflavonol	Prothrombin	F2
GC58	Isolicoflavonol	Estrogen receptor	ESR1
GC58	Isolicoflavonol	Androgen receptor	AR
GC58	Isolicoflavonol	Peroxisome proliferator-activated receptor gamma	PPARG
GC58	Isolicoflavonol	Coagulation factor X	F10

GC58	Isolicoflavonol	Prostaglandin G/H synthase 2	PTGS2
GC58	Isolicoflavonol	Glycogen synthase kinase-3 beta	GSK3B
GC58	Isolicoflavonol	Heat shock protein HSP 90-alpha	HSP90AA1
GC58	Isolicoflavonol	Trypsin-1	PRSS1
GC58	Isolicoflavonol	Proto-oncogene serine/threonine-protein kinase Pim-1	PIM1
GC58	Isolicoflavonol	Cyclin-A2	CCNA2
GC58	Isolicoflavonol	Nuclear receptor coactivator 2	NCOA2
GC59	HMO	Nitric oxide synthase, inducible	NOS2
GC59	HMO	Prostaglandin G/H synthase 1	PTGS1
GC59	HMO	Muscarinic acetylcholine receptor M1	CHRM1
GC59	HMO	Estrogen receptor	ESR1
GC59	HMO	Androgen receptor	AR
GC59	HMO	Sodium channel protein type 5 subunit alpha	SCN5A
GC59	HMO	Peroxisome proliferator-activated receptor gamma	PPARG
GC59	HMO	Prostaglandin G/H synthase 2	PTGS2
GC59	HMO	Retinoic acid receptor RXR-alpha	RXRA
GC59	HMO	cGMP-inhibited 3',5'-cyclic phosphodiesterase A	PDE3A
GC59	HMO	Sodium-dependent dopamine transporter	SLC6A3
GC59	HMO	Beta-2 adrenergic receptor	ADRB2
GC59	HMO	Sodium-dependent serotonin transporter	SLC6A4
GC59	HMO	Estrogen receptor beta	ESR2
GC59	HMO	Dipeptidyl peptidase 4	DPP4
GC59	HMO	Mitogen-activated protein kinase 14	MAPK14
GC59	HMO	Glycogen synthase kinase-3 beta	GSK3B
GC59	HMO	Amine oxidase [flavin-containing] B	MAOB
GC59	HMO	Serine/threonine-protein kinase Chk1	CHEK1
GC59	HMO	cAMP-dependent protein kinase catalytic subunit alpha	PRKACA
GC59	HMO	Trypsin-1	PRSS1
GC59	HMO	Proto-oncogene serine/threonine-protein kinase Pim-1	PIM1
GC59	HMO	Cyclin-A2	CCNA2
GC59	HMO	cAMP-dependent protein kinase inhibitor alpha	PKIA
GC60	1-Methoxyphaseollidin	Nitric oxide synthase, inducible	NOS2
GC60	1-Methoxyphaseollidin	Prostaglandin G/H synthase 1	PTGS1
GC60	1-Methoxyphaseollidin	Prothrombin	F2
GC60	1-Methoxyphaseollidin	Potassium voltage-gated channel subfamily H member 2	KCNH2
GC60	1-Methoxyphaseollidin	Estrogen receptor	ESR1

GC60	1-Methoxyphaseollidin	Androgen receptor	AR
GC60	1-Methoxyphaseollidin	Sodium channel protein type 5 subunit alpha	SCN5A
GC60	1-Methoxyphaseollidin	Peroxisome proliferator-activated receptor gamma	PPARG
GC60	1-Methoxyphaseollidin	Coagulation factor X	F10
GC60	1-Methoxyphaseollidin	Prostaglandin G/H synthase 2	PTGS2
GC60	1-Methoxyphaseollidin	Vascular endothelial growth factor receptor 2	KDR
GC60	1-Methoxyphaseollidin	Retinoic acid receptor RXR-alpha	RXRA
GC60	1-Methoxyphaseollidin	Alpha-1B adrenergic receptor	ADRA1B
GC60	1-Methoxyphaseollidin	Beta-2 adrenergic receptor	ADRB2
GC60	1-Methoxyphaseollidin	Alpha-1D adrenergic receptor	ADRA1D
GC60	1-Methoxyphaseollidin	DNA topoisomerase 2-alpha	TOP2A
GC60	1-Methoxyphaseollidin	Estrogen receptor beta	ESR2
GC60	1-Methoxyphaseollidin	Mitogen-activated protein kinase 14	MAPK14
GC60	1-Methoxyphaseollidin	Glycogen synthase kinase-3 beta	GSK3B
GC60	1-Methoxyphaseollidin	Heat shock protein HSP 90-alpha	HSP90AA1
GC60	1-Methoxyphaseollidin	Phosphatidylinositol-4,5-bisphosphate 3-kinase catalytic subunit gamma isoform	PIK3CG
GC60	1-Methoxyphaseollidin	Trypsin-1	PRSS1
GC60	1-Methoxyphaseollidin	Proto-oncogene serine/threonine-protein kinase Pim-1	PIM1

GC60	1-Methoxyphaseollidin	Cyclin-A2	CCNA2
GC60	1-Methoxyphaseollidin	Nuclear receptor coactivator 2	NCOA2
GC60	1-Methoxyphaseollidin	Nuclear receptor coactivator 1	NCOA1
GC61	Quercetin der.	Nitric oxide synthase, inducible	NOS2
GC61	Quercetin der.	Prostaglandin G/H synthase 1	PTGS1
GC61	Quercetin der.	Estrogen receptor	ESR1
GC61	Quercetin der.	Androgen receptor	AR
GC61	Quercetin der.	Sodium channel protein type 5 subunit alpha	SCN5A
GC61	Quercetin der.	Peroxisome proliferator-activated receptor gamma	PPARG
GC61	Quercetin der.	Prostaglandin G/H synthase 2	PTGS2
GC61	Quercetin der.	Tyrosine-protein phosphatase non-receptor type 1	PTPN1
GC61	Quercetin der.	Estrogen receptor beta	ESR2
GC61	Quercetin der.	Dipeptidyl peptidase 4	DPP4
GC61	Quercetin der.	Mitogen-activated protein kinase 14	MAPK14
GC61	Quercetin der.	Glycogen synthase kinase-3 beta	GSK3B
GC61	Quercetin der.	Heat shock protein HSP 90-alpha	HSP90AA1
GC61	Quercetin der.	Trypsin-1	PRSS1
GC61	Quercetin der.	Nuclear receptor coactivator 2	NCOA2
GC62	3'-Hydroxy-4'-O-Methylglabridin	Nitric oxide synthase, inducible	NOS2
GC62	3'-Hydroxy-4'-O-Methylglabridin	Prostaglandin G/H synthase 1	PTGS1
GC62	3'-Hydroxy-4'-O-Methylglabridin	Potassium voltage-gated channel subfamily H member 2	KCNH2
GC62	3'-Hydroxy-4'-O-Methylglabridin	Estrogen receptor	ESR1
GC62	3'-Hydroxy-4'-O-Methylglabridin	Androgen receptor	AR
GC62	3'-Hydroxy-4'-O-Methylglabridin	Sodium channel protein type 5 subunit alpha	SCN5A
GC62	3'-Hydroxy-4'-O-Methylglabridin	Peroxisome proliferator-activated receptor gamma	PPARG
GC62	3'-Hydroxy-4'-O-Methylglabridin	Coagulation factor X	F10
GC62	3'-Hydroxy-4'-O-Methylglabridin	Prostaglandin G/H synthase 2	PTGS2
GC62	3'-Hydroxy-4'-O-Methylglabridin	Coagulation factor VII	F7
GC62	3'-Hydroxy-4'-O-Methylglabridin	Vascular endothelial growth factor receptor 2	KDR
GC62	3'-Hydroxy-4'-O-Methylglabridin	Alpha-1B adrenergic receptor	ADRA1B
GC62	3'-Hydroxy-4'-O-Methylglabridin	Beta-2 adrenergic receptor	ADRB2
GC62	3'-Hydroxy-4'-O-Methylglabridin	DNA topoisomerase 2-alpha	TOP2A

GC62	3'-Hydroxy-4'-O-Methylglabridin	Estrogen receptor beta	ESR2
GC62	3'-Hydroxy-4'-O-Methylglabridin	Mitogen-activated protein kinase 14	MAPK14
GC62	3'-Hydroxy-4'-O-Methylglabridin	Glycogen synthase kinase-3 beta	GSK3B
GC62	3'-Hydroxy-4'-O-Methylglabridin	Heat shock protein HSP 90-alpha	HSP90AA1
GC62	3'-Hydroxy-4'-O-Methylglabridin	Serine/threonine-protein kinase Chk1	CHEK1
GC62	3'-Hydroxy-4'-O-Methylglabridin	cAMP-dependent protein kinase catalytic subunit alpha	PRKACA
GC62	3'-Hydroxy-4'-O-Methylglabridin	Trypsin-1	PRSS1
GC62	3'-Hydroxy-4'-O-Methylglabridin	Proto-oncogene serine/threonine-protein kinase Pim-1	PIM1
GC62	3'-Hydroxy-4'-O-Methylglabridin	Cyclin-A2	CCNA2
GC62	3'-Hydroxy-4'-O-Methylglabridin	Nuclear receptor coactivator 2	NCOA2
GC62	3'-Hydroxy-4'-O-Methylglabridin	Nuclear receptor coactivator 1	NCOA1
GC62	3'-Hydroxy-4'-O-Methylglabridin	Calcium-activated potassium channel subunit alpha 1	KCNMA1
GC63	licochalcone a	Nitric oxide synthase, inducible	NOS2
GC63	licochalcone a	Prostaglandin G/H synthase 1	PTGS1
GC63	licochalcone a	Muscarinic acetylcholine receptor M1	CHRM1
GC63	licochalcone a	Estrogen receptor	ESR1
GC63	licochalcone a	Androgen receptor	AR
GC63	licochalcone a	Sodium channel protein type 5 subunit alpha	SCN5A
GC63	licochalcone a	Peroxisome proliferator-activated receptor gamma	PPARG
GC63	licochalcone a	Coagulation factor X	F10
GC63	licochalcone a	Prostaglandin G/H synthase 2	PTGS2
GC63	licochalcone a	Carbonic anhydrase 2	CA2
GC63	licochalcone a	Alpha-1B adrenergic receptor	ADRA1B
GC63	licochalcone a	Sodium-dependent dopamine transporter	SLC6A3
GC63	licochalcone a	Estrogen receptor beta	ESR2
GC63	licochalcone a	Mitogen-activated protein kinase 14	MAPK14
GC63	licochalcone a	Glycogen synthase kinase-3 beta	GSK3B
GC63	licochalcone a	Heat shock protein HSP 90-alpha	HSP90AA1
GC63	licochalcone a	Serine/threonine-protein kinase Chk1	CHEK1
GC63	licochalcone a	Proto-oncogene serine/threonine-protein kinase Pim-1	PIM1
GC63	licochalcone a	Cyclin-A2	CCNA2
GC63	licochalcone a	Beta-2 adrenergic receptor	ADRB2
GC63	licochalcone a	Nuclear receptor coactivator 2	NCOA2
GC63	licochalcone a	Transcription factor p65	RELA
GC63	licochalcone a	Signal transducer and activator of transcription 3	STAT3
GC63	licochalcone a	G1/S-specific cyclin-D1	CCND1
GC63	licochalcone a	Apoptosis regulator Bcl-2	BCL2
GC63	licochalcone a	Eukaryotic translation initiation factor 6	EIF6
GC63	licochalcone a	Mitogen-activated protein kinase 1	MAPK1

GC63	licochalcone a	Retinoblastoma-associated protein	RB1
GC63	licochalcone a	Cell division protein kinase 4	CDK4
GC63	licochalcone a	Fos-related antigen 2	FOSL2
GC64	3'-Methoxyglabridin	Nitric oxide synthase, inducible	NOS2
GC64	3'-Methoxyglabridin	Prostaglandin G/H synthase 1	PTGS1
GC64	3'-Methoxyglabridin	Potassium voltage-gated channel subfamily H member 2	KCNH2
GC64	3'-Methoxyglabridin	Estrogen receptor	ESR1
GC64	3'-Methoxyglabridin	Androgen receptor	AR
GC64	3'-Methoxyglabridin	Sodium channel protein type 5 subunit alpha	SCN5A
GC64	3'-Methoxyglabridin	Peroxisome proliferator-activated receptor gamma	PPARG
GC64	3'-Methoxyglabridin	Coagulation factor X	F10
GC64	3'-Methoxyglabridin	Prostaglandin G/H synthase 2	PTGS2
GC64	3'-Methoxyglabridin	Coagulation factor VII	F7
GC64	3'-Methoxyglabridin	Retinoic acid receptor RXR-alpha	RXRA
GC64	3'-Methoxyglabridin	Acetylcholinesterase	ACHE
GC64	3'-Methoxyglabridin	Alpha-1B adrenergic receptor	ADRA1B
GC64	3'-Methoxyglabridin	Beta-2 adrenergic receptor	ADRB2
GC64	3'-Methoxyglabridin	DNA topoisomerase 2-alpha	TOP2A
GC64	3'-Methoxyglabridin	Estrogen receptor beta	ESR2
GC64	3'-Methoxyglabridin	Mitogen-activated protein kinase 14	MAPK14
GC64	3'-Methoxyglabridin	Glycogen synthase kinase-3 beta	GSK3B
GC64	3'-Methoxyglabridin	Heat shock protein HSP 90-alpha	HSP90AA1
GC64	3'-Methoxyglabridin	Serine/threonine-protein kinase Chk1	CHEK1
GC64	3'-Methoxyglabridin	Trypsin-1	PRSS1
GC64	3'-Methoxyglabridin	Proto-oncogene serine/threonine-protein kinase Pim-1	PIM1
GC64	3'-Methoxyglabridin	Cyclin-A2	CCNA2
GC64	3'-Methoxyglabridin	Nuclear receptor coactivator 2	NCOA2
GC64	3'-Methoxyglabridin	Nuclear receptor coactivator 1	NCOA1
GC64	3'-Methoxyglabridin	Calcium-activated potassium channel subunit alpha 1	KCNMA1

GC65	2-[(3R)-8,8-dimethyl-3,4-dihydro-2H-pyrano[6,5-f]chromen-3-yl]-5-methoxyphenol	Nitric oxide synthase, inducible	NOS2
GC65	2-[(3R)-8,8-dimethyl-3,4-dihydro-2H-pyrano[6,5-f]chromen-3-yl]-5-methoxyphenol	Prostaglandin G/H synthase 1	PTGS1
GC65	2-[(3R)-8,8-dimethyl-3,4-dihydro-2H-pyrano[6,5-f]chromen-3-yl]-5-methoxyphenol	Muscarinic acetylcholine receptor M3	CHRM3
GC65	2-[(3R)-8,8-dimethyl-3,4-dihydro-2H-pyrano[6,5-f]chromen-3-yl]-5-methoxyphenol	Potassium voltage-gated channel subfamily H member 2	KCNH2
GC65	2-[(3R)-8,8-dimethyl-3,4-dihydro-2H-pyrano[6,5-f]chromen-3-yl]-5-methoxyphenol	Muscarinic acetylcholine receptor M1	CHRM1
GC65	2-[(3R)-8,8-dimethyl-3,4-dihydro-2H-pyrano[6,5-f]chromen-3-yl]-5-methoxyphenol	Estrogen receptor	ESR1
GC65	2-[(3R)-8,8-dimethyl-3,4-dihydro-2H-pyrano[6,5-f]chromen-3-yl]-5-methoxyphenol	Androgen receptor	AR
GC65	2-[(3R)-8,8-dimethyl-3,4-dihydro-2H-pyrano[6,5-f]chromen-3-yl]-5-methoxyphenol	Sodium channel protein type 5 subunit alpha	SCN5A
GC65	2-[(3R)-8,8-dimethyl-3,4-dihydro-2H-pyrano[6,5-f]chromen-3-yl]-5-methoxyphenol	Peroxisome proliferator-activated receptor gamma	PPARG

GC65	2-[(3R)-8,8-dimethyl-3,4-dihydro-2H-pyrano[6,5-f]chromen-3-yl]-5-methoxyphenol	Coagulation factor X	F10
GC65	2-[(3R)-8,8-dimethyl-3,4-dihydro-2H-pyrano[6,5-f]chromen-3-yl]-5-methoxyphenol	Prostaglandin G/H synthase 2	PTGS2
GC65	2-[(3R)-8,8-dimethyl-3,4-dihydro-2H-pyrano[6,5-f]chromen-3-yl]-5-methoxyphenol	Retinoic acid receptor RXR-alpha	RXRA
GC65	2-[(3R)-8,8-dimethyl-3,4-dihydro-2H-pyrano[6,5-f]chromen-3-yl]-5-methoxyphenol	Acetylcholinesterase	ACHE
GC65	2-[(3R)-8,8-dimethyl-3,4-dihydro-2H-pyrano[6,5-f]chromen-3-yl]-5-methoxyphenol	Alpha-1B adrenergic receptor	ADRA1B
GC65	2-[(3R)-8,8-dimethyl-3,4-dihydro-2H-pyrano[6,5-f]chromen-3-yl]-5-methoxyphenol	Sodium-dependent dopamine transporter	SLC6A3
GC65	2-[(3R)-8,8-dimethyl-3,4-dihydro-2H-pyrano[6,5-f]chromen-3-yl]-5-methoxyphenol	Beta-2 adrenergic receptor	ADRB2
GC65	2-[(3R)-8,8-dimethyl-3,4-dihydro-2H-pyrano[6,5-f]chromen-3-yl]-5-methoxyphenol	Estrogen receptor beta	ESR2
GC65	2-[(3R)-8,8-dimethyl-3,4-dihydro-2H-pyrano[6,5-f]chromen-3-yl]-5-methoxyphenol	Mitogen-activated protein kinase 14	MAPK14

GC65	2-[(3R)-8,8-dimethyl-3,4-dihydro-2H-pyrano[6,5-f]chromen-3-yl]-5-methoxyphenol	Glycogen synthase kinase-3 beta	GSK3B
GC65	2-[(3R)-8,8-dimethyl-3,4-dihydro-2H-pyrano[6,5-f]chromen-3-yl]-5-methoxyphenol	Serine/threonine-protein kinase Chk1	CHEK1
GC65	2-[(3R)-8,8-dimethyl-3,4-dihydro-2H-pyrano[6,5-f]chromen-3-yl]-5-methoxyphenol	cAMP-dependent protein kinase catalytic subunit alpha	PRKACA
GC65	2-[(3R)-8,8-dimethyl-3,4-dihydro-2H-pyrano[6,5-f]chromen-3-yl]-5-methoxyphenol	Trypsin-1	PRSS1
GC65	2-[(3R)-8,8-dimethyl-3,4-dihydro-2H-pyrano[6,5-f]chromen-3-yl]-5-methoxyphenol	Proto-oncogene serine/threonine-protein kinase Pim-1	PIM1
GC65	2-[(3R)-8,8-dimethyl-3,4-dihydro-2H-pyrano[6,5-f]chromen-3-yl]-5-methoxyphenol	Cyclin-A2	CCNA2
GC65	2-[(3R)-8,8-dimethyl-3,4-dihydro-2H-pyrano[6,5-f]chromen-3-yl]-5-methoxyphenol	Nuclear receptor coactivator 2	NCOA2
GC65	2-[(3R)-8,8-dimethyl-3,4-dihydro-2H-pyrano[6,5-f]chromen-3-yl]-5-methoxyphenol	Nuclear receptor coactivator 1	NCOA1
GC65	2-[(3R)-8,8-dimethyl-3,4-dihydro-2H-pyrano[6,5-f]chromen-3-yl]-5-methoxyphenol	Calcium-activated potassium channel subunit alpha 1	KCNMA1
GC66	Inflacoumarin A	Prothrombin	F2

GC66	Inflacoumarin A	Estrogen receptor	ESR1
GC66	Inflacoumarin A	Androgen receptor	AR
GC66	Inflacoumarin A	Peroxisome proliferator-activated receptor gamma	PPARG
GC66	Inflacoumarin A	Coagulation factor X	F10
GC66	Inflacoumarin A	Prostaglandin G/H synthase 2	PTGS2
GC66	Inflacoumarin A	Beta-2 adrenergic receptor	ADRB2
GC66	Inflacoumarin A	Dipeptidyl peptidase 4	DPP4
GC66	Inflacoumarin A	Heat shock protein HSP 90-alpha	HSP90AA1
GC66	Inflacoumarin A	Trypsin-1	PRSS1
GC66	Inflacoumarin A	Proto-oncogene serine/threonine-protein kinase Pim-1	PIM1
GC66	Inflacoumarin A	Nuclear receptor coactivator 2	NCOA2
GC66	Inflacoumarin A	Prostaglandin G/H synthase 1	PTGS1
GC66	Inflacoumarin A	Sodium channel protein type 5 subunit alpha	SCN5A
GC67	icos-5-enoic acid	Nuclear receptor coactivator 2	NCOA2
GC68	Kanzonol F	Estrogen receptor	ESR1
GC68	Kanzonol F	Androgen receptor	AR
GC68	Kanzonol F	Coagulation factor X	F10
GC68	Kanzonol F	Prostaglandin G/H synthase 2	PTGS2
GC68	Kanzonol F	Estrogen receptor beta	ESR2
GC68	Kanzonol F	Proto-oncogene serine/threonine-protein kinase Pim-1	PIM1
GC68	Kanzonol F	Nuclear receptor coactivator 2	NCOA2
GC69	6-prenylated eriodictyol	Nitric oxide synthase, inducible	NOS2
GC69	6-prenylated eriodictyol	Estrogen receptor	ESR1
GC69	6-prenylated eriodictyol	Sodium channel protein type 5 subunit alpha	SCN5A
GC69	6-prenylated eriodictyol	Coagulation factor X	F10
GC69	6-prenylated eriodictyol	Prostaglandin G/H synthase 2	PTGS2
GC69	6-prenylated eriodictyol	Coagulation factor VII	F7
GC69	6-prenylated eriodictyol	Heat shock protein HSP 90-alpha	HSP90AA1
GC70	7,2',4'-trihydroxy – 5-methoxy-3 – arylcoumarin	Nitric oxide synthase, inducible	NOS2
GC70	7,2',4'-trihydroxy – 5-methoxy-3 – arylcoumarin	Prostaglandin G/H synthase 1	PTGS1
GC70	7,2',4'-trihydroxy – 5-methoxy-3 – arylcoumarin	Estrogen receptor	ESR1
GC70	7,2',4'-trihydroxy – 5-methoxy-3 – arylcoumarin	Androgen receptor	AR
GC70	7,2',4'-trihydroxy – 5-methoxy-3 – arylcoumarin	Peroxisome proliferator-activated receptor gamma	PPARG

GC70	7,2',4'-trihydroxy－5-methoxy-3－arylcoumarin	Prostaglandin G/H synthase 2	PTGS2
GC70	7,2',4'-trihydroxy－5-methoxy-3－arylcoumarin	Estrogen receptor beta	ESR2
GC70	7,2',4'-trihydroxy－5-methoxy-3－arylcoumarin	Dipeptidyl peptidase 4	DPP4
GC70	7,2',4'-trihydroxy－5-methoxy-3－arylcoumarin	Mitogen-activated protein kinase 14	MAPK14
GC70	7,2',4'-trihydroxy－5-methoxy-3－arylcoumarin	Glycogen synthase kinase-3 beta	GSK3B
GC70	7,2',4'-trihydroxy－5-methoxy-3－arylcoumarin	Heat shock protein HSP 90-alpha	HSP90AA1
GC70	7,2',4'-trihydroxy－5-methoxy-3－arylcoumarin	Serine/threonine-protein kinase Chk1	CHEK1
GC70	7,2',4'-trihydroxy－5-methoxy-3－arylcoumarin	cAMP-dependent protein kinase catalytic subunit alpha	PRKACA
GC70	7,2',4'-trihydroxy－5-methoxy-3－arylcoumarin	Proto-oncogene serine/threonine-protein kinase Pim-1	PIM1
GC71	7-Acetoxy-2-methylisoflavone	Nitric oxide synthase, inducible	NOS2
GC71	7-Acetoxy-2-methylisoflavone	Prostaglandin G/H synthase 1	PTGS1
GC71	7-Acetoxy-2-methylisoflavone	Prothrombin	F2
GC71	7-Acetoxy-2-methylisoflavone	Estrogen receptor	ESR1
GC71	7-Acetoxy-2-methylisoflavone	Androgen receptor	AR
GC71	7-Acetoxy-2-methylisoflavone	Sodium channel protein type 5 subunit alpha	SCN5A
GC71	7-Acetoxy-2-methylisoflavone	Peroxisome proliferator-activated receptor gamma	PPARG
GC71	7-Acetoxy-2-methylisoflavone	Prostaglandin G/H synthase 2	PTGS2
GC71	7-Acetoxy-2-methylisoflavone	Retinoic acid receptor RXR-alpha	RXRA
GC71	7-Acetoxy-2-methylisoflavone	Acetylcholinesterase	ACHE
GC71	7-Acetoxy-2-methylisoflavone	cGMP-inhibited 3',5'-cyclic phosphodiesterase A	PDE3A
GC71	7-Acetoxy-2-methylisoflavone	Alpha-1B adrenergic receptor	ADRA1B
GC71	7-Acetoxy-2-methylisoflavone	Beta-2 adrenergic receptor	ADRB2
GC71	7-Acetoxy-2-methylisoflavone	Alpha-1D adrenergic receptor	ADRA1D

GC71	7-Acetoxy-2-methylisoflavone	Gamma-aminobutyric-acid receptor subunit alpha-1	GABRA1
GC71	7-Acetoxy-2-methylisoflavone	Dipeptidyl peptidase 4	DPP4
GC71	7-Acetoxy-2-methylisoflavone	Mitogen-activated protein kinase 14	MAPK14
GC71	7-Acetoxy-2-methylisoflavone	Glycogen synthase kinase-3 beta	GSK3B
GC71	7-Acetoxy-2-methylisoflavone	Heat shock protein HSP 90-alpha	HSP90AA1
GC71	7-Acetoxy-2-methylisoflavone	Serine/threonine-protein kinase Chk1	CHEK1
GC71	7-Acetoxy-2-methylisoflavone	Trypsin-1	PRSS1
GC71	7-Acetoxy-2-methylisoflavone	Nuclear receptor coactivator 2	NCOA2
GC72	8-prenylated eriodictyol	Estrogen receptor	ESR1
GC72	8-prenylated eriodictyol	Sodium channel protein type 5 subunit alpha	SCN5A
GC72	8-prenylated eriodictyol	Coagulation factor X	F10
GC72	8-prenylated eriodictyol	Prostaglandin G/H synthase 2	PTGS2
GC72	8-prenylated eriodictyol	Coagulation factor VII	F7
GC72	8-prenylated eriodictyol	Heat shock protein HSP 90-alpha	HSP90AA1
GC72	8-prenylated eriodictyol	Nuclear receptor coactivator 1	NCOA1
GC73	gadelaidic acid	Nuclear receptor coactivator 2	NCOA2
GC74	Vestitol	Nitric oxide synthase, inducible	NOS2
GC74	Vestitol	Prostaglandin G/H synthase 1	PTGS1
GC74	Vestitol	Muscarinic acetylcholine receptor M1	CHRM1
GC74	Vestitol	Estrogen receptor	ESR1
GC74	Vestitol	Androgen receptor	AR
GC74	Vestitol	Sodium channel protein type 5 subunit alpha	SCN5A
GC74	Vestitol	Peroxisome proliferator-activated receptor gamma	PPARG
GC74	Vestitol	Prostaglandin G/H synthase 2	PTGS2
GC74	Vestitol	Muscarinic acetylcholine receptor M4	CHRM4
GC74	Vestitol	Retinoic acid receptor RXR-alpha	RXRA
GC74	Vestitol	cGMP-inhibited 3',5'-cyclic phosphodiesterase A	PDE3A
GC74	Vestitol	5-hydroxytryptamine 2A receptor	HTR2A
GC74	Vestitol	Alpha-1A adrenergic receptor	ADRA1A
GC74	Vestitol	Alpha-1B adrenergic receptor	ADRA1B
GC74	Vestitol	Sodium-dependent dopamine transporter	SLC6A3
GC74	Vestitol	Beta-2 adrenergic receptor	ADRB2
GC74	Vestitol	Sodium-dependent serotonin transporter	SLC6A4
GC74	Vestitol	Estrogen receptor beta	ESR2
GC74	Vestitol	Dipeptidyl peptidase 4	DPP4
GC74	Vestitol	Mitogen-activated protein kinase 14	MAPK14
GC74	Vestitol	Glycogen synthase kinase-3 beta	GSK3B

GC74	Vestitol	Heat shock protein HSP 90-alpha	HSP90AA1
GC74	Vestitol	Serine/threonine-protein kinase Chk1	CHEK1
GC74	Vestitol	cAMP-dependent protein kinase catalytic subunit alpha	PRKACA
GC74	Vestitol	Trypsin-1	PRSS1
GC74	Vestitol	Proto-oncogene serine/threonine-protein kinase Pim-1	PIM1
GC74	Vestitol	Cyclin-A2	CCNA2
GC74	Vestitol	cAMP-dependent protein kinase inhibitor alpha	PKIA
GC75	Gancaonin G	Nitric oxide synthase, inducible	NOS2
GC75	Gancaonin G	Prothrombin	F2
GC75	Gancaonin G	Estrogen receptor	ESR1
GC75	Gancaonin G	Androgen receptor	AR
GC75	Gancaonin G	Peroxisome proliferator-activated receptor gamma	PPARG
GC75	Gancaonin G	Coagulation factor X	F10
GC75	Gancaonin G	Prostaglandin G/H synthase 2	PTGS2
GC75	Gancaonin G	DNA topoisomerase 2-alpha	TOP2A
GC75	Gancaonin G	Estrogen receptor beta	ESR2
GC75	Gancaonin G	Dipeptidyl peptidase 4	DPP4
GC75	Gancaonin G	Mitogen-activated protein kinase 14	MAPK14
GC75	Gancaonin G	Glycogen synthase kinase-3 beta	GSK3B
GC75	Gancaonin G	Heat shock protein HSP 90-alpha	HSP90AA1
GC75	Gancaonin G	Serine/threonine-protein kinase Chk1	CHEK1
GC75	Gancaonin G	Trypsin-1	PRSS1
GC75	Gancaonin G	Proto-oncogene serine/threonine-protein kinase Pim-1	PIM1
GC75	Gancaonin G	Cyclin-A2	CCNA2
GC75	Gancaonin G	Nuclear receptor coactivator 2	NCOA2
GC76	Gancaonin H	Estrogen receptor	ESR1
GC76	Gancaonin H	Androgen receptor	AR
GC76	Gancaonin H	Coagulation factor X	F10
GC76	Gancaonin H	Prostaglandin G/H synthase 2	PTGS2
GC76	Gancaonin H	Vascular endothelial growth factor receptor 2	KDR
GC76	Gancaonin H	DNA topoisomerase 2-alpha	TOP2A
GC76	Gancaonin H	Heat shock protein HSP 90-alpha	HSP90AA1
GC76	Gancaonin H	Trypsin-1	PRSS1
GC76	Gancaonin H	Proto-oncogene serine/threonine-protein kinase Pim-1	PIM1
GC76	Gancaonin H	Cyclin-A2	CCNA2
GC76	Gancaonin H	Nuclear receptor coactivator 2	NCOA2
GC77	Licoagrocarpin	Nitric oxide synthase, inducible	NOS2
GC77	Licoagrocarpin	Prostaglandin G/H synthase 1	PTGS1
GC77	Licoagrocarpin	Muscarinic acetylcholine receptor M3	CHRM3
GC77	Licoagrocarpin	Prothrombin	F2
GC77	Licoagrocarpin	Potassium voltage-gated channel subfamily H member 2	KCNH2
GC77	Licoagrocarpin	Muscarinic acetylcholine receptor M1	CHRM1
GC77	Licoagrocarpin	Estrogen receptor	ESR1
GC77	Licoagrocarpin	Androgen receptor	AR
GC77	Licoagrocarpin	Sodium channel protein type 5 subunit alpha	SCN5A

GC77	Licoagrocarpin	Peroxisome proliferator-activated receptor gamma	PPARG
GC77	Licoagrocarpin	Coagulation factor X	F10
GC77	Licoagrocarpin	Muscarinic acetylcholine receptor M5	CHRM5
GC77	Licoagrocarpin	Prostaglandin G/H synthase 2	PTGS2
GC77	Licoagrocarpin	Retinoic acid receptor RXR-alpha	RXRA
GC77	Licoagrocarpin	Acetylcholinesterase	ACHE
GC77	Licoagrocarpin	Alpha-1B adrenergic receptor	ADRA1B
GC77	Licoagrocarpin	Beta-2 adrenergic receptor	ADRB2
GC77	Licoagrocarpin	Estrogen receptor beta	ESR2
GC77	Licoagrocarpin	Mitogen-activated protein kinase 14	MAPK14
GC77	Licoagrocarpin	Glycogen synthase kinase-3 beta	GSK3B
GC77	Licoagrocarpin	Heat shock protein HSP 90-alpha	HSP90AA1
GC77	Licoagrocarpin	Trypsin-1	PRSS1
GC77	Licoagrocarpin	Proto-oncogene serine/threonine-protein kinase Pim-1	PIM1
GC77	Licoagrocarpin	Cyclin-A2	CCNA2
GC77	Licoagrocarpin	Nuclear receptor coactivator 2	NCOA2
GC78	Glyasperins M	Nitric oxide synthase, inducible	NOS2
GC78	Glyasperins M	Prostaglandin G/H synthase 1	PTGS1
GC78	Glyasperins M	Potassium voltage-gated channel subfamily H member 2	KCNH2
GC78	Glyasperins M	Estrogen receptor	ESR1
GC78	Glyasperins M	Androgen receptor	AR
GC78	Glyasperins M	Sodium channel protein type 5 subunit alpha	SCN5A
GC78	Glyasperins M	Peroxisome proliferator-activated receptor gamma	PPARG
GC78	Glyasperins M	Coagulation factor X	F10
GC78	Glyasperins M	Prostaglandin G/H synthase 2	PTGS2
GC78	Glyasperins M	Coagulation factor VII	F7
GC78	Glyasperins M	Vascular endothelial growth factor receptor 2	KDR
GC78	Glyasperins M	Acetylcholinesterase	ACHE
GC78	Glyasperins M	DNA topoisomerase 2-alpha	TOP2A
GC78	Glyasperins M	Estrogen receptor beta	ESR2
GC78	Glyasperins M	Peroxisome proliferator-activated receptor delta	PPARD
GC78	Glyasperins M	Glycogen synthase kinase-3 beta	GSK3B
GC78	Glyasperins M	Heat shock protein HSP 90-alpha	HSP90AA1
GC78	Glyasperins M	cAMP-dependent protein kinase catalytic subunit alpha	PRKACA
GC78	Glyasperins M	Trypsin-1	PRSS1
GC78	Glyasperins M	Proto-oncogene serine/threonine-protein kinase Pim-1	PIM1
GC78	Glyasperins M	Cyclin-A2	CCNA2
GC78	Glyasperins M	Nuclear receptor coactivator 2	NCOA2
GC78	Glyasperins M	Nuclear receptor coactivator 1	NCOA1
GC78	Glyasperins M	Calcium-activated potassium channel subunit alpha 1	KCNMA1
GC79	Glycyrrhiza flavonol A	Nitric oxide synthase, inducible	NOS2
GC79	Glycyrrhiza flavonol A	Estrogen receptor	ESR1

GC79	Glycyrrhiza flavonol A	Androgen receptor	AR
GC79	Glycyrrhiza flavonol A	Coagulation factor X	F10
GC79	Glycyrrhiza flavonol A	Prostaglandin G/H synthase 2	PTGS2
GC79	Glycyrrhiza flavonol A	Coagulation factor VII	F7
GC79	Glycyrrhiza flavonol A	Acetylcholinesterase	ACHE
GC79	Glycyrrhiza flavonol A	DNA topoisomerase 2-alpha	TOP2A
GC79	Glycyrrhiza flavonol A	Estrogen receptor beta	ESR2
GC79	Glycyrrhiza flavonol A	Dipeptidyl peptidase 4	DPP4
GC79	Glycyrrhiza flavonol A	Glycogen synthase kinase-3 beta	GSK3B
GC79	Glycyrrhiza flavonol A	Heat shock protein HSP 90-alpha	HSP90AA1
GC79	Glycyrrhiza flavonol A	Trypsin-1	PRSS1
GC79	Glycyrrhiza flavonol A	Proto-oncogene serine/threonine-protein kinase Pim-1	PIM1
GC79	Glycyrrhiza flavonol A	Cyclin-A2	CCNA2
GC80	Licoagroisoflavone	Nitric oxide synthase, inducible	NOS2
GC80	Licoagroisoflavone	Prothrombin	F2
GC80	Licoagroisoflavone	Estrogen receptor	ESR1
GC80	Licoagroisoflavone	Androgen receptor	AR
GC80	Licoagroisoflavone	Sodium channel protein type 5 subunit alpha	SCN5A
GC80	Licoagroisoflavone	Peroxisome proliferator-activated receptor gamma	PPARG
GC80	Licoagroisoflavone	Coagulation factor X	F10
GC80	Licoagroisoflavone	Prostaglandin G/H synthase 2	PTGS2
GC80	Licoagroisoflavone	Estrogen receptor beta	ESR2
GC80	Licoagroisoflavone	Dipeptidyl peptidase 4	DPP4
GC80	Licoagroisoflavone	Mitogen-activated protein kinase 14	MAPK14
GC80	Licoagroisoflavone	Glycogen synthase kinase-3 beta	GSK3B
GC80	Licoagroisoflavone	Serine/threonine-protein kinase Chk1	CHEK1
GC80	Licoagroisoflavone	Trypsin-1	PRSS1
GC80	Licoagroisoflavone	Proto-oncogene serine/threonine-protein kinase Pim-1	PIM1
GC80	Licoagroisoflavone	Cyclin-A2	CCNA2
GC81	Odoratin	Nitric oxide synthase, inducible	NOS2
GC81	Odoratin	Prostaglandin G/H synthase 1	PTGS1
GC81	Odoratin	Estrogen receptor	ESR1
GC81	Odoratin	Androgen receptor	AR
GC81	Odoratin	Sodium channel protein type 5 subunit alpha	SCN5A
GC81	Odoratin	Peroxisome proliferator-activated receptor gamma	PPARG
GC81	Odoratin	Prostaglandin G/H synthase 2	PTGS2
GC81	Odoratin	Retinoic acid receptor RXR-alpha	RXRA

GC81	Odoratin	Estrogen receptor beta	ESR2
GC81	Odoratin	Dipeptidyl peptidase 4	DPP4
GC81	Odoratin	Mitogen-activated protein kinase 14	MAPK14
GC81	Odoratin	Glycogen synthase kinase-3 beta	GSK3B
GC81	Odoratin	Heat shock protein HSP 90-alpha	HSP90AA1
GC81	Odoratin	Serine/threonine-protein kinase Chk1	CHEK1
GC81	Odoratin	Trypsin-1	PRSS1
GC81	Odoratin	Proto-oncogene serine/threonine-protein kinase Pim-1	PIM1
GC81	Odoratin	Cyclin-A2	CCNA2
GC81	Odoratin	Nuclear receptor coactivator 2	NCOA2
GC82	Phaseol	Prothrombin	F2
GC82	Phaseol	Estrogen receptor	ESR1
GC82	Phaseol	Androgen receptor	AR
GC82	Phaseol	Peroxisome proliferator-activated receptor gamma	PPARG
GC82	Phaseol	Prostaglandin G/H synthase 2	PTGS2
GC82	Phaseol	Vascular endothelial growth factor receptor 2	KDR
GC82	Phaseol	Mitogen-activated protein kinase 14	MAPK14
GC82	Phaseol	Glycogen synthase kinase-3 beta	GSK3B
GC82	Phaseol	Heat shock protein HSP 90-alpha	HSP90AA1
GC82	Phaseol	Serine/threonine-protein kinase Chk1	CHEK1
GC82	Phaseol	cAMP-dependent protein kinase catalytic subunit alpha	PRKACA
GC82	Phaseol	Proto-oncogene serine/threonine-protein kinase Pim-1	PIM1
GC82	Phaseol	Cyclin-A2	CCNA2
GC83	Xambioona	Nitric oxide synthase, inducible	NOS2
GC83	Xambioona	Estrogen receptor	ESR1
GC83	Xambioona	Coagulation factor X	F10
GC83	Xambioona	Prostaglandin G/H synthase 2	PTGS2
GC83	Xambioona	Estrogen receptor beta	ESR2
GC83	Xambioona	Proto-oncogene serine/threonine-protein kinase Pim-1	PIM1
GC83	Xambioona	Nuclear receptor coactivator 2	NCOA2
GC84	dehydroglyasperins C	Nitric oxide synthase, inducible	NOS2
GC84	dehydroglyasperins C	Estrogen receptor	ESR1
GC84	dehydroglyasperins C	Androgen receptor	AR
GC84	dehydroglyasperins C	Sodium channel protein type 5 subunit alpha	SCN5A
GC84	dehydroglyasperins C	Peroxisome proliferator-activated receptor gamma	PPARG
GC84	dehydroglyasperins C	Coagulation factor X	F10
GC84	dehydroglyasperins C	Prostaglandin G/H synthase 2	PTGS2
GC84	dehydroglyasperins C	Beta-2 adrenergic receptor	ADRB2
GC84	dehydroglyasperins C	Estrogen receptor beta	ESR2

GC84	dehydroglyasperins C	Mitogen-activated protein kinase 14	MAPK14
GC84	dehydroglyasperins C	Heat shock protein HSP 90-alpha	HSP90AA1
GC84	dehydroglyasperins C	Serine/threonine-protein kinase Chk1	CHEK1
GC84	dehydroglyasperins C	Trypsin-1	PRSS1
GC84	dehydroglyasperins C	Proto-oncogene serine/threonine-protein kinase Pim-1	PIM1
GC84	dehydroglyasperins C	Cyclin-A2	CCNA2
GC84	dehydroglyasperins C	Nuclear receptor coactivator 2	NCOA2
CPL	quercetin	Prostaglandin G/H synthase 1	PTGS1
CPL	quercetin	Androgen receptor	AR
CPL	quercetin	Peroxisome proliferator-activated receptor gamma	PPARG
CPL	quercetin	Prostaglandin G/H synthase 2	PTGS2
CPL	quercetin	Heat shock protein HSP 90-alpha	HSP90AA1
CPL	quercetin	Phosphatidylinositol-4,5-bisphosphate 3-kinase catalytic subunit gamma isoform	PIK3CG
CPL	quercetin	Nuclear receptor coactivator 2	NCOA2
CPL	quercetin	Dipeptidyl peptidase 4	DPP4
CPL	quercetin	Trypsin-1	PRSS1
CPL	quercetin	DNA topoisomerase 2-alpha	TOP2A
CPL	quercetin	Prothrombin	F2
CPL	quercetin	Potassium voltage-gated channel subfamily H member 2	KCNH2
CPL	quercetin	Sodium channel protein type 5 subunit alpha	SCN5A
CPL	quercetin	Coagulation factor X	F10
CPL	quercetin	Beta-2 adrenergic receptor	ADRB2
CPL	quercetin	Stromelysin-1	MMP3
CPL	quercetin	cAMP-dependent protein kinase catalytic subunit alpha	PRKACA
CPL	quercetin	Coagulation factor VII	F7
CPL	quercetin	Retinoic acid receptor RXR-alpha	RXRA
CPL	quercetin	Acetylcholinesterase	ACHE
CPL	quercetin	Gamma-aminobutyric-acid receptor subunit alpha-1	GABRA1
CPL	quercetin	Amine oxidase [flavin-containing] B	MAOB
CPL	quercetin	Transcription factor p65	RELA
CPL	quercetin	Epidermal growth factor receptor	EGFR
CPL	quercetin	RAC-alpha serine/threonine-protein kinase	AKT1
CPL	quercetin	G1/S-specific cyclin-D1	CCND1
CPL	quercetin	Apoptosis regulator Bcl-2	BCL2
CPL	quercetin	Bcl-2-like protein 1	BCL2L1
CPL	quercetin	Proto-oncogene c-Fos	FOS
CPL	quercetin	Cyclin-dependent kinase inhibitor 1	CDKN1A
CPL	quercetin	Eukaryotic translation initiation factor 6	EIF6
CPL	quercetin	Apoptosis regulator BAX	BAX
CPL	quercetin	Caspase-9	CASP9
CPL	quercetin	Urokinase-type plasminogen activator	PLAU
CPL	quercetin	72 kDa type IV collagenase	MMP2

CPL	quercetin	Matrix metalloproteinase-9	MMP9
CPL	quercetin	Mitogen-activated protein kinase 1	MAPK1
CPL	quercetin	Interleukin-10	IL10
CPL	quercetin	Retinoblastoma-associated protein	RB1
CPL	quercetin	Tumor necrosis factor	TNF
CPL	quercetin	Transcription factor AP-1	JUN
CPL	quercetin	Interleukin-6	IL6
CPL	quercetin	Activator of 90 kDa heat shock protein ATPase homolog 1	AHSA1
CPL	quercetin	Caspase-3	CASP3
CPL	quercetin	Cellular tumor antigen p53	TP53
CPL	quercetin	ETS domain-containing protein Elk-1	ELK1
CPL	quercetin	NF-kappa-B inhibitor alpha	NFKBIA
CPL	quercetin	Ornithine decarboxylase	ODC1
CPL	quercetin	Xanthine dehydrogenase/oxidase	XDH
CPL	quercetin	Caspase-8	CASP8
CPL	quercetin	DNA topoisomerase 1	TOP1
CPL	quercetin	RAF proto-oncogene serine/threonine-protein kinase	RAF1
CPL	quercetin	Superoxide dismutase [Cu-Zn]	SOD1
CPL	quercetin	Protein kinase C alpha type	PRKCA
CPL	quercetin	Interstitial collagenase	MMP1
CPL	quercetin	Hypoxia-inducible factor 1-alpha	HIF1A
CPL	quercetin	Signal transducer and activator of transcription 1-alpha/beta	STAT1
CPL	quercetin	Protein CBFA2T1	RUNX1T1
CPL	quercetin	78 kDa glucose-regulated protein	HSPA5
CPL	quercetin	Receptor tyrosine-protein kinase erbB-2	ERBB2
CPL	quercetin	Peroxisome proliferator-activated receptor gamma	PPARG
CPL	quercetin	Acetyl-CoA carboxylase 1	ACACA
CPL	quercetin	Heme oxygenase 1	HMOX1
CPL	quercetin	Cytochrome P450 3A4	CYP3A4
CPL	quercetin	Caveolin-1	CAV1
CPL	quercetin	Myc proto-oncogene protein	MYC
CPL	quercetin	Tissue factor	F3
CPL	quercetin	Gap junction alpha-1 protein	GJA1
CPL	quercetin	Cytochrome P450 1A1	CYP1A1
CPL	quercetin	Intercellular adhesion molecule 1	ICAM1
CPL	quercetin	Interleukin-1 beta	IL1B
CPL	quercetin	Small inducible cytokine A2	CCL2
CPL	quercetin	E-selectin	SELE
CPL	quercetin	Vascular cell adhesion protein 1	VCAM1
CPL	quercetin	Prostaglandin E2 receptor, EP3 subtype	PTGER3
CPL	quercetin	Interleukin-8	CXCL8
CPL	quercetin	Protein kinase C beta type	PRKCB
CPL	quercetin	Baculoviral IAP repeat-containing protein 5	BIRC5
CPL	quercetin	Dual oxidase 2	DUOX2
CPL	quercetin	Nitric oxide synthase, endothelial	NOS3
CPL	quercetin	Heat shock protein beta-1	HSPB1
CPL	quercetin	Transforming growth factor beta-1	TGFB1
CPL	quercetin	Maltase-glucoamylase, intestinal	MGAM
CPL	quercetin	Interleukin-2	IL2
CPL	quercetin	Nuclear receptor subfamily 1 group I member 2	NR1I2

CPL	quercetin	Cytochrome P450 1B1	CYP1B1
CPL	quercetin	G2/mitotic-specific cyclin-B1	CCNB1
CPL	quercetin	Tissue-type plasminogen activator	PLAT
CPL	quercetin	Thrombomodulin	THBD
CPL	quercetin	Plasminogen activator inhibitor 1	SERPINE1
CPL	quercetin	Interferon gamma	IFNG
CPL	quercetin	Arachidonate 5-lipoxygenase	ALOX5
CPL	quercetin	Phosphatidylinositol-3,4,5-trisphosphate 3-phosphatase and dual-specificity protein phosphatase PTEN	PTEN
CPL	quercetin	Interleukin-1 alpha	IL1A
CPL	quercetin	Myeloperoxidase	MPO
CPL	quercetin	DNA topoisomerase 2-alpha	TOP2A
CPL	quercetin	Neutrophil cytosol factor 1	NCF1
CPL	quercetin	ATP-binding cassette sub-family G member 2	ABCA2
CPL	quercetin	Hyaluronan synthase 2	HAS2
CPL	quercetin	Nuclear factor erythroid 2-related factor 2	NFE2L2
CPL	quercetin	NAD(P)H dehydrogenase [quinone] 1	NQO1
CPL	quercetin	Poly [ADP-ribose] polymerase 1	PARP1
CPL	quercetin	Aryl hydrocarbon receptor	AHR
CPL	quercetin	26S proteasome non-ATPase regulatory subunit 3	PSMD3
CPL	quercetin	Solute carrier family 2, facilitated glucose transporter member 4	SLC2A4
CPL	quercetin	Collagen alpha-1(III) chain	COL3A1
CPL	quercetin	C-X-C motif chemokine 11	CXCL11
CPL	quercetin	C-X-C motif chemokine 2	CXCL2
CPL	quercetin	DDB1- and CUL4-associated factor 5	DCAF5
CPL	quercetin	Nuclear receptor subfamily 1 group I member 3	NR1I3
CPL	quercetin	Serine/threonine-protein kinase Chk2	CHEK2
CPL	quercetin	Insulin receptor	INSR
CPL	quercetin	Claudin-4	CLDN4
CPL	quercetin	Peroxisome proliferator-activated receptor alpha	PPARA
CPL	quercetin	Peroxisome proliferator-activated receptor delta	PPARD
CPL	quercetin	Heat shock factor protein 1	HSF1
CPL	quercetin	C-reactive protein	CRP
CPL	quercetin	C-X-C motif chemokine 10	CXCL10
CPL	quercetin	Inhibitor of nuclear factor kappa-B kinase subunit alpha	CHUK
CPL	quercetin	Osteopontin	SPP1
CPL	quercetin	Runt-related transcription factor 2	RUNX2
CPL	quercetin	Ras association domain-containing protein 1	RASSF1
CPL	quercetin	Transcription factor E2F1	E2F1
CPL	quercetin	Transcription factor E2F2	E2F2
CPL	quercetin	Prostatic acid phosphatase	ACP3
CPL	quercetin	Cathepsin D	CTSD
CPL	quercetin	Insulin-like growth factor-binding protein 3	IGFBP3
CPL	quercetin	Insulin-like growth factor II	IGF2
CPL	quercetin	CD40 ligand	CD40LG
CPL	quercetin	Interferon regulatory factor 1	IRF1
CPL	quercetin	Receptor tyrosine-protein kinase erbB-3	ERBB3

CPL	quercetin	Serum paraoxonase/arylesterase 1	PON1
CPL	quercetin	Type I iodothyronine deiodinase	DIO1
CPL	quercetin	Procollagen C-endopeptidase enhancer 1	PCOLCE
CPL	quercetin	Puromycin-sensitive aminopeptidase	NPEPPS
CPL	quercetin	Hexokinase-2	HK2
CPL	quercetin	Homeobox protein Nkx-3.1	NKX3-1
CPL	quercetin	Ras GTPase-activating protein 1	RASA1
CPL	quercetin	Glutathione S-transferase Mu 1	GSTM1
CPL	quercetin	Glutathione S-transferase Mu 2	GSTM2

Zingiberis

Abbreviation	Molecule Name	Target Name	Genesymbol
CJQ	sitosterol	Progesterone receptor	PGR
CJQ	sitosterol	Nuclear receptor coactivator 2	NCOA2
CJQ	sitosterol	Mineralocorticoid receptor	NR3C2

Trichosanthis Fructus

Abbreviation	Molecule Name	Target Name	Genesymbol
GL1	Mandenol	Prostaglandin G/H synthase 1	PTGS1
GL1	Mandenol	Prostaglandin G/H synthase 2	PTGS2
GL1	Mandenol	Nuclear receptor coactivator 2	NCOA2
GL2	Diosmetin	Nitric oxide synthase, inducible	NOS2
GL2	Diosmetin	Prostaglandin G/H synthase 1	PTGS1
GL2	Diosmetin	Prostaglandin G/H synthase 2	PTGS2
GL2	Diosmetin	Dipeptidyl peptidase 4	DPP4
GL2	Diosmetin	Heat shock protein HSP 90-alpha	HSP90AA1
GL2	Diosmetin	cAMP-dependent protein kinase catalytic subunit alpha	PRKACA
GL2	Diosmetin	Trypsin-1	PRSS1
GL2	Diosmetin	Nuclear receptor coactivator 2	NCOA2
GL2	Diosmetin	Nuclear receptor coactivator 1	NCOA1
GL3	Spinasterol	Progesterone receptor	PGR
GL3	Spinasterol	Mineralocorticoid receptor	NR3C2
GL3	Spinasterol	Nuclear receptor coactivator 2	NCOA2
GL4	Hydroxygenkwanin	Nitric oxide synthase, inducible	NOS2
GL4	Hydroxygenkwanin	Prostaglandin G/H synthase 1	PTGS1
GL4	Hydroxygenkwanin	Prostaglandin G/H synthase 2	PTGS2
GL4	Hydroxygenkwanin	Dipeptidyl peptidase 4	DPP4
GL4	Hydroxygenkwanin	Heat shock protein HSP 90-alpha	HSP90AA1
GL4	Hydroxygenkwanin	cAMP-dependent protein kinase catalytic subunit alpha	PRKACA
GL4	Hydroxygenkwanin	Trypsin-1	PRSS1
GL4	Hydroxygenkwanin	Nuclear receptor coactivator 2	NCOA2
GL4	Hydroxygenkwanin	Phosphatidylinositol-4,5-bisphosphate 3-kinase catalytic subunit gamma isoform	PIK3CG
GL5	Schottenol	Progesterone receptor	PGR
GL5	Schottenol	Nuclear receptor coactivator 2	NCOA2
GL6	10α-cucurbita-5,24-diene-3β-ol	Progesterone receptor	PGR
GL6	10α-cucurbita-5,24-diene-3β-ol	Mineralocorticoid receptor	NR3C2
GL7	5-dehydrokarounidiol	Glucocorticoid receptor	NR3C1
GL8	7-oxo-dihydrokaro-unidiol	Mineralocorticoid receptor	NR3C2
GL9	Linolenic acid ethyl ester	Prostaglandin G/H synthase 1	PTGS1
GL9	Linolenic acid ethyl ester	Prostaglandin G/H synthase 2	PTGS2
GL10	vitamin-e	Coagulation factor X	F10

Coptidis

Abbreviation	Molecule Name	Target Name	Genesymbol
HL1	berberine	Nitric oxide synthase, inducible	NOS2
HL1	berberine	Prostaglandin G/H synthase 1	PTGS1
HL1	berberine	Potassium voltage-gated channel subfamily H member 2	KCNH2
HL1	berberine	Estrogen receptor	ESR1
HL1	berberine	Androgen receptor	AR
HL1	berberine	Sodium channel protein type 5 subunit alpha	SCN5A
HL1	berberine	Coagulation factor X	F10
HL1	berberine	Prostaglandin G/H synthase 2	PTGS2
HL1	berberine	Nitric-oxide synthase, endothelial	NOS3
HL1	berberine	Retinoic acid receptor RXR-alpha	RXRA
HL1	berberine	Beta-2 adrenergic receptor	ADRB2
HL1	berberine	Heat shock protein HSP 90-alpha	HSP90AA1
HL1	berberine	cAMP-dependent protein kinase catalytic subunit alpha	PRKACA
HL1	berberine	Trypsin-1	PRSS1
HL1	berberine	Nuclear receptor coactivator 2	NCOA2
HL1	berberine	cAMP and cAMP-inhibited cGMP 3',5'-cyclic phosphodiesterase 10A	PDE10A
HL2	Magnograndiolide	Gamma-aminobutyric-acid receptor subunit alpha-2	GABRA2
HL2	Magnograndiolide	Gamma-aminobutyric-acid receptor subunit alpha-1	GABRA1
HL2	Magnograndiolide	Glutamate receptor 2	GRIA2
HL2	Magnograndiolide	Gamma-aminobutyric-acid receptor subunit alpha-6	GABRA6
HL3	palmatine	Nitric oxide synthase, inducible	NOS2
HL3	palmatine	Prostaglandin G/H synthase 1	PTGS1
HL3	palmatine	Potassium voltage-gated channel subfamily H member 2	KCNH2
HL3	palmatine	Estrogen receptor	ESR1
HL3	palmatine	Androgen receptor	AR
HL3	palmatine	Sodium channel protein type 5 subunit alpha	SCN5A
HL3	palmatine	Prostaglandin G/H synthase 2	PTGS2
HL3	palmatine	Nitric-oxide synthase, endothelial	NOS3
HL3	palmatine	Retinoic acid receptor RXR-alpha	RXRA
HL3	palmatine	Beta-2 adrenergic receptor	ADRB2
HL3	palmatine	Estrogen receptor beta	ESR2
HL3	palmatine	Heat shock protein HSP 90-alpha	HSP90AA1
HL3	palmatine	Trypsin-1	PRSS1
HL3	palmatine	Proto-oncogene serine/threonine-protein kinase Pim-1	PIM1
HL3	palmatine	Nuclear receptor coactivator 2	NCOA2
HL3	palmatine	cAMP-dependent protein kinase catalytic subunit alpha	PRKACA
HL3	palmatine	Coagulation factor VII	F7
CPL	quercetin	Prostaglandin G/H synthase 1	PTGS1
CPL	quercetin	Androgen receptor	AR
CPL	quercetin	Peroxisome proliferator-activated receptor gamma	PPARG
CPL	quercetin	Prostaglandin G/H synthase 2	PTGS2

CPL	quercetin	Heat shock protein HSP 90-alpha	HSP90AA1
CPL	quercetin	Phosphatidylinositol-4,5-bisphosphate 3-kinase catalytic subunit gamma isoform	PIK3CG
CPL	quercetin	Nuclear receptor coactivator 2	NCOA2
CPL	quercetin	Dipeptidyl peptidase 4	DPP4
CPL	quercetin	Trypsin-1	PRSS1
CPL	quercetin	DNA topoisomerase 2-alpha	TOP2A
CPL	quercetin	Prothrombin	F2
CPL	quercetin	Potassium voltage-gated channel subfamily H member 2	KCNH2
CPL	quercetin	Sodium channel protein type 5 subunit alpha	SCN5A
CPL	quercetin	Coagulation factor X	F10
CPL	quercetin	Beta-2 adrenergic receptor	ADRB2
CPL	quercetin	Stromelysin-1	MMP3
CPL	quercetin	cAMP-dependent protein kinase catalytic subunit alpha	PRKACA
CPL	quercetin	Coagulation factor VII	F7
CPL	quercetin	Nitric-oxide synthase, endothelial	NOS3
CPL	quercetin	Retinoic acid receptor RXR-alpha	RXRA
CPL	quercetin	Acetylcholinesterase	ACHE
CPL	quercetin	Gamma-aminobutyric-acid receptor subunit alpha-1	GABRA1
CPL	quercetin	Amine oxidase [flavin-containing] B	MAOB
CPL	quercetin	Transcription factor p65	RELA
CPL	quercetin	Epidermal growth factor receptor	EGFR
CPL	quercetin	RAC-alpha serine/threonine-protein kinase	AKT1
CPL	quercetin	G1/S-specific cyclin-D1	CCND1
CPL	quercetin	Apoptosis regulator Bcl-2	BCL2
CPL	quercetin	Bcl-2-like protein 1	BCL2L1
CPL	quercetin	Proto-oncogene c-Fos	FOS
CPL	quercetin	Cyclin-dependent kinase inhibitor 1	CDKN1A
CPL	quercetin	Eukaryotic translation initiation factor 6	EIF6
CPL	quercetin	Apoptosis regulator BAX	BAX
CPL	quercetin	Caspase-9	CASP9
CPL	quercetin	Urokinase-type plasminogen activator	PLAU
CPL	quercetin	72 kDa type IV collagenase	MMP2
CPL	quercetin	Matrix metalloproteinase-9	MMP9
CPL	quercetin	Mitogen-activated protein kinase 1	MAPK1
CPL	quercetin	Interleukin-10	IL10
CPL	quercetin	Retinoblastoma-associated protein	RB1
CPL	quercetin	Tumor necrosis factor	TNF
CPL	quercetin	Transcription factor AP-1	JUN
CPL	quercetin	Interleukin-6	IL6
CPL	quercetin	Activator of 90 kDa heat shock protein ATPase homolog 1	AHSA1
CPL	quercetin	Caspase-3	CASP3
CPL	quercetin	Cellular tumor antigen p53	TP53
CPL	quercetin	ETS domain-containing protein Elk-1	ELK1
CPL	quercetin	NF-kappa-B inhibitor alpha	NFKBIA
CPL	quercetin	Ornithine decarboxylase	ODC1
CPL	quercetin	Xanthine dehydrogenase/oxidase	XDH
CPL	quercetin	Caspase-8	CASP8
CPL	quercetin	DNA topoisomerase 1	TOP1
CPL	quercetin	RAF proto-oncogene serine/threonine-protein kinase	RAF1

CPL	quercetin	Superoxide dismutase [Cu-Zn]	SOD1
CPL	quercetin	Protein kinase C alpha type	PRKCA
CPL	quercetin	Interstitial collagenase	MMP1
CPL	quercetin	Hypoxia-inducible factor 1-alpha	HIF1A
CPL	quercetin	Signal transducer and activator of transcription 1-alpha/beta	STAT1
CPL	quercetin	Protein CBFA2T1	RUNX1T1
CPL	quercetin	78 kDa glucose-regulated protein	HSPA5
CPL	quercetin	Receptor tyrosine-protein kinase erbB-2	ERBB2
CPL	quercetin	Peroxisome proliferator-activated receptor gamma	PPARG
CPL	quercetin	Acetyl-CoA carboxylase 1	ACACA
CPL	quercetin	Heme oxygenase 1	HMOX1
CPL	quercetin	Cytochrome P450 3A4	CYP3A4
CPL	quercetin	Caveolin-1	CAV1
CPL	quercetin	Myc proto-oncogene protein	MYC
CPL	quercetin	Tissue factor	F3
CPL	quercetin	Gap junction alpha-1 protein	GJA1
CPL	quercetin	Cytochrome P450 1A1	CYP1A1
CPL	quercetin	Intercellular adhesion molecule 1	ICAM1
CPL	quercetin	Interleukin-1 beta	IL1B
CPL	quercetin	Small inducible cytokine A2	CCL2
CPL	quercetin	E-selectin	SELE
CPL	quercetin	Vascular cell adhesion protein 1	VCAM1
CPL	quercetin	Prostaglandin E2 receptor, EP3 subtype	PTGER3
CPL	quercetin	Interleukin-8	CXCL8
CPL	quercetin	Protein kinase C beta type	PRKCB
CPL	quercetin	Baculoviral IAP repeat-containing protein 5	BIRC5
CPL	quercetin	Dual oxidase 2	DUOX2
CPL	quercetin	Nitric oxide synthase, endothelial	NOS3
CPL	quercetin	Heat shock protein beta-1	HSPB1
CPL	quercetin	Transforming growth factor beta-1	TGFB1
CPL	quercetin	Maltase-glucoamylase, intestinal	MGAM
CPL	quercetin	Interleukin-2	IL2
CPL	quercetin	Nuclear receptor subfamily 1 group I member 2	NR1I2
CPL	quercetin	Cytochrome P450 1B1	CYP1B1
CPL	quercetin	G2/mitotic-specific cyclin-B1	CCNB1
CPL	quercetin	Tissue-type plasminogen activator	PLAT
CPL	quercetin	Thrombomodulin	THBD
CPL	quercetin	Plasminogen activator inhibitor 1	SERPINE1
CPL	quercetin	Interferon gamma	IFNG
CPL	quercetin	Arachidonate 5-lipoxygenase	ALOX5
CPL	quercetin	Phosphatidylinositol-3,4,5-trisphosphate 3-phosphatase and dual-specificity protein phosphatase PTEN	PTEN
CPL	quercetin	Interleukin-1 alpha	IL1A
CPL	quercetin	Myeloperoxidase	MPO
CPL	quercetin	DNA topoisomerase 2-alpha	TOP2A
CPL	quercetin	Neutrophil cytosol factor 1	NCF1
CPL	quercetin	ATP-binding cassette sub-family G member 2	ABCA2
CPL	quercetin	Hyaluronan synthase 2	HAS2
CPL	quercetin	Nuclear factor erythroid 2-related factor 2	NFE2L2
CPL	quercetin	Poly [ADP-ribose] polymerase 1	PARP1
CPL	quercetin	Aryl hydrocarbon receptor	AHR

CPL	quercetin	26S proteasome non-ATPase regulatory subunit 3	PSMD3
CPL	quercetin	Solute carrier family 2, facilitated glucose transporter member 4	SLC2A4
CPL	quercetin	Collagen alpha-1(III) chain	COL3A1
CPL	quercetin	C-X-C motif chemokine 11	CXCL11
CPL	quercetin	C-X-C motif chemokine 2	CXCL2
CPL	quercetin	DDB1- and CUL4-associated factor 5	DCAF5
CPL	quercetin	Nuclear receptor subfamily 1 group I member 3	NR1I3
CPL	quercetin	Serine/threonine-protein kinase Chk2	CHEK2
CPL	quercetin	Insulin receptor	INSR
CPL	quercetin	Claudin-4	CLDN4
CPL	quercetin	Peroxisome proliferator-activated receptor alpha	PPARA
CPL	quercetin	Peroxisome proliferator-activated receptor delta	PPARD
CPL	quercetin	Heat shock factor protein 1	HSF1
CPL	quercetin	C-reactive protein	CRP
CPL	quercetin	C-X-C motif chemokine 10	CXCL10
CPL	quercetin	Inhibitor of nuclear factor kappa-B kinase subunit alpha	CHUK
CPL	quercetin	Osteopontin	SPP1
CPL	quercetin	Runt-related transcription factor 2	RUNX2
CPL	quercetin	Ras association domain-containing protein 1	RASSF1
CPL	quercetin	Transcription factor E2F1	E2F1
CPL	quercetin	Transcription factor E2F2	E2F2
CPL	quercetin	Prostatic acid phosphatase	ACP3
CPL	quercetin	Cathepsin D	CTSD
CPL	quercetin	Insulin-like growth factor-binding protein 3	IGFBP3
CPL	quercetin	Insulin-like growth factor II	IGF2
CPL	quercetin	CD40 ligand	CD40LG
CPL	quercetin	Interferon regulatory factor 1	IRF1
CPL	quercetin	Receptor tyrosine-protein kinase erbB-3	ERBB3
CPL	quercetin	Serum paraoxonase/arylesterase 1	PON1
CPL	quercetin	Type I iodothyronine deiodinase	DIO1
CPL	quercetin	Procollagen C-endopeptidase enhancer 1	PCOLCE
CPL	quercetin	Puromycin-sensitive aminopeptidase	NPEPPS
CPL	quercetin	Hexokinase-2	HK2
CPL	quercetin	Homeobox protein Nkx-3.1	NKX3-1
CPL	quercetin	Ras GTPase-activating protein 1	RASA1
CPL	quercetin	Glutathione S-transferase Mu 1	GSTM1
CPL	quercetin	Glutathione S-transferase Mu 2	GSTM2
LQ	coptisine	Nitric oxide synthase, inducible	NOS2
LQ	coptisine	Prostaglandin G/H synthase 1	PTGS1
LQ	coptisine	Potassium voltage-gated channel subfamily H member 2	KCNH2
LQ	coptisine	Estrogen receptor	ESR1
LQ	coptisine	Androgen receptor	AR
LQ	coptisine	Sodium channel protein type 5 subunit alpha	SCN5A
LQ	coptisine	Prostaglandin G/H synthase 2	PTGS2
LQ	coptisine	Nitric-oxide synthase, endothelial	NOS3
LQ	coptisine	Trypsin-1	PRSS1
HL4	Worenine	Nitric oxide synthase, inducible	NOS2
HL4	Worenine	Prostaglandin G/H synthase 1	PTGS1

HL4	Worenine	Estrogen receptor	ESR1
HL4	Worenine	Androgen receptor	AR
HL4	Worenine	Prostaglandin G/H synthase 2	PTGS2
HL4	Worenine	Serine/threonine-protein kinase Chk1	CHEK1
HL4	Worenine	Proto-oncogene serine/threonine-protein kinase Pim-1	PIM1

Scutellaria

Abbreviation	Molecule Name	Target Name	Genesymbol
HQ1	acacetin	Nitric oxide synthase, inducible	NOS2
HQ1	acacetin	Prostaglandin G/H synthase 1	PTGS1
HQ1	acacetin	Androgen receptor	AR
HQ1	acacetin	Prostaglandin G/H synthase 2	PTGS2
HQ1	acacetin	Dipeptidyl peptidase 4	DPP4
HQ1	acacetin	Heat shock protein HSP 90-alpha	HSP90AA1
HQ1	acacetin	cAMP-dependent protein kinase catalytic subunit alpha	PRKACA
HQ1	acacetin	Trypsin-1	PRSS1
HQ1	acacetin	Nuclear receptor coactivator 2	NCOA2
HQ1	acacetin	Nuclear receptor coactivator 1	NCOA1
HQ1	acacetin	Phosphatidylinositol-4,5-bisphosphate 3-kinase catalytic subunit gamma isoform	PIK3CG
HQ1	acacetin	Serine/threonine-protein kinase Chk1	CHEK1
HQ1	acacetin	Beta-2 adrenergic receptor	ADRB2
HQ1	acacetin	cGMP-inhibited 3',5'-cyclic phosphodiesterase A	PDE3A
HQ1	acacetin	Transcription factor p65	RELA
HQ1	acacetin	Apoptosis regulator Bcl-2	BCL2
HQ1	acacetin	Cyclin-dependent kinase inhibitor 1	CDKN1A
HQ1	acacetin	Apoptosis regulator BAX	BAX
HQ1	acacetin	Caspase-3	CASP3
HQ1	acacetin	Cellular tumor antigen p53	TP53
HQ1	acacetin	Caspase-8	CASP8
HQ1	acacetin	Fatty acid synthase	FASN
HQ1	acacetin	Tumor necrosis factor ligand superfamily member 6	FASLG
HQ1	acacetin	Cytochrome P450 19A1	CYP19A1
HQ2	wogonin	Nitric oxide synthase, inducible	NOS2
HQ2	wogonin	Prostaglandin G/H synthase 1	PTGS1
HQ2	wogonin	Estrogen receptor	ESR1
HQ2	wogonin	Androgen receptor	AR
HQ2	wogonin	Sodium channel protein type 5 subunit alpha	SCN5A
HQ2	wogonin	Peroxisome proliferator-activated receptor gamma	PPARG
HQ2	wogonin	Prostaglandin G/H synthase 2	PTGS2
HQ2	wogonin	Retinoic acid receptor RXR-alpha	RXRA
HQ2	wogonin	cGMP-inhibited 3',5'-cyclic phosphodiesterase A	PDE3A
HQ2	wogonin	Dipeptidyl peptidase 4	DPP4
HQ2	wogonin	Mitogen-activated protein kinase 14	MAPK14
HQ2	wogonin	Glycogen synthase kinase-3 beta	GSK3B
HQ2	wogonin	Heat shock protein HSP 90-alpha	HSP90AA1
HQ2	wogonin	Phosphatidylinositol-4,5-bisphosphate 3-kinase catalytic subunit gamma isoform	PIK3CG
HQ2	wogonin	Serine/threonine-protein kinase Chk1	CHEK1
HQ2	wogonin	cAMP-dependent protein kinase catalytic subunit alpha	PRKACA
HQ2	wogonin	Trypsin-1	PRSS1
HQ2	wogonin	Beta-2 adrenergic receptor	ADRB2
HQ2	wogonin	Gamma-aminobutyric-acid receptor subunit alpha-1	GABRA1
HQ2	wogonin	Transcription factor p65	RELA
HQ2	wogonin	RAC-alpha serine/threonine-protein kinase	AKT1

HQ2	wogonin	G1/S-specific cyclin-D1	CCND1
HQ2	wogonin	Apoptosis regulator Bcl-2	BCL2
HQ2	wogonin	Cyclin-dependent kinase inhibitor 1	CDKN1A
HQ2	wogonin	Eukaryotic translation initiation factor 6	EIF6
HQ2	wogonin	Apoptosis regulator BAX	BAX
HQ2	wogonin	Caspase-9	CASP9
HQ2	wogonin	Vascular endothelial growth factor receptor 2	KDR
HQ2	wogonin	Tumor necrosis factor	TNF
HQ2	wogonin	Transcription factor AP-1	JUN
HQ2	wogonin	Interleukin-6	IL6
HQ2	wogonin	Activator of 90 kDa heat shock protein ATPase homolog 1	AHSA1
HQ2	wogonin	Caspase-3	CASP3
HQ2	wogonin	Cellular tumor antigen p53	TP53
HQ2	wogonin	Bcl-2-binding component 3	BBC3
HQ2	wogonin	Telomerase protein component 1	TEP1
HQ2	wogonin	Interstitial collagenase	MMP1
HQ2	wogonin	Small inducible cytokine A2	CCL2
HQ2	wogonin	Protein kinase C delta type	PRKCD
HQ2	wogonin	Prostaglandin E2 receptor, EP3 subtype	PTGER3
HQ2	wogonin	Fibronectin	FN1
HQ2	wogonin	Interleukin-8	CXCL8
HQ2	wogonin	Induced myeloid leukemia cell differentiation protein Mcl-1	MCL1
HQ3	(2R)-7-hydroxy-5-methoxy-2-phenylchroman-4-one	Prostaglandin G/H synthase 1	PTGS1
HQ3	(2R)-7-hydroxy-5-methoxy-2-phenylchroman-4-one	D(1A) dopamine receptor	DRD1
HQ3	(2R)-7-hydroxy-5-methoxy-2-phenylchroman-4-one	Muscarinic acetylcholine receptor M3	CHRM3
HQ3	(2R)-7-hydroxy-5-methoxy-2-phenylchroman-4-one	Muscarinic acetylcholine receptor M1	CHRM1
HQ3	(2R)-7-hydroxy-5-methoxy-2-phenylchroman-4-one	Estrogen receptor	ESR1
HQ3	(2R)-7-hydroxy-5-methoxy-2-phenylchroman-4-one	Sodium channel protein type 5 subunit alpha	SCN5A
HQ3	(2R)-7-hydroxy-5-methoxy-2-phenylchroman-4-one	Prostaglandin G/H synthase 2	PTGS2

HQ3	(2R)-7-hydroxy-5-methoxy-2-phenylchroman-4-one	Retinoic acid receptor RXR-alpha	RXRA
HQ3	(2R)-7-hydroxy-5-methoxy-2-phenylchroman-4-one	cGMP-inhibited 3',5'-cyclic phosphodiesterase A	PDE3A
HQ3	(2R)-7-hydroxy-5-methoxy-2-phenylchroman-4-one	Alpha-1A adrenergic receptor	ADRA1A
HQ3	(2R)-7-hydroxy-5-methoxy-2-phenylchroman-4-one	Alpha-1B adrenergic receptor	ADRA1B
HQ3	(2R)-7-hydroxy-5-methoxy-2-phenylchroman-4-one	Sodium-dependent dopamine transporter	SLC6A3
HQ3	(2R)-7-hydroxy-5-methoxy-2-phenylchroman-4-one	Beta-2 adrenergic receptor	ADRB2
HQ3	(2R)-7-hydroxy-5-methoxy-2-phenylchroman-4-one	Sodium-dependent serotonin transporter	SLC6A4
HQ3	(2R)-7-hydroxy-5-methoxy-2-phenylchroman-4-one	Gamma-aminobutyric-acid receptor subunit alpha-1	GABRA1
HQ3	(2R)-7-hydroxy-5-methoxy-2-phenylchroman-4-one	Heat shock protein HSP 90-alpha	HSP90AA1
HQ3	(2R)-7-hydroxy-5-methoxy-2-phenylchroman-4-one	Phosphatidylinositol-4,5-bisphosphate 3-kinase catalytic subunit gamma isoform	PIK3CG
HQ3	(2R)-7-hydroxy-5-methoxy-2-phenylchroman-4-one	cAMP-dependent protein kinase catalytic subunit alpha	PRKACA
HQ3	(2R)-7-hydroxy-5-methoxy-2-phenylchroman-4-one	cAMP-dependent protein kinase inhibitor alpha	PKIA
HQ3	(2R)-7-hydroxy-5-methoxy-2-phenylchroman-4-one	Neuronal acetylcholine receptor subunit alpha-7	CHRNA7

HQ3	(2R)-7-hydroxy-5-methoxy-2-phenylchroman-4-one	Amine oxidase [flavin-containing] B	MAOB
XQ1	baicalein	Prostaglandin G/H synthase 1	PTGS1
XQ1	baicalein	Androgen receptor	AR
XQ1	baicalein	Prostaglandin G/H synthase 2	PTGS2
XQ1	baicalein	Heat shock protein HSP 90-alpha	HSP90AA1
XQ1	baicalein	cAMP-dependent protein kinase catalytic subunit alpha	PRKACA
XQ1	baicalein	Dipeptidyl peptidase 4	DPP4
XQ1	baicalein	Phosphatidylinositol-4,5-bisphosphate 3-kinase catalytic subunit gamma isoform	PIK3CG
XQ1	baicalein	cGMP-inhibited 3',5'-cyclic phosphodiesterase A	PDE3A
XQ1	baicalein	Trypsin-1	PRSS1
XQ1	baicalein	Nuclear receptor coactivator 2	NCOA2
XQ1	baicalein	Nuclear receptor coactivator 1	NCOA1
XQ1	baicalein	Transcription factor p65	RELA
XQ1	baicalein	RAC-alpha serine/threonine-protein kinase	AKT1
XQ1	baicalein	Apoptosis regulator Bcl-2	BCL2
XQ1	baicalein	Proto-oncogene c-Fos	FOS
XQ1	baicalein	Apoptosis regulator BAX	BAX
XQ1	baicalein	Matrix metalloproteinase-9	MMP9
XQ1	baicalein	Caspase-3	CASP3
XQ1	baicalein	Cellular tumor antigen p53	TP53
XQ1	baicalein	Hypoxia-inducible factor 1-alpha	HIF1A
XQ1	baicalein	Fos-related antigen 1	FOSL1
XQ1	baicalein	Fos-related antigen 2	FOSL2
XQ1	baicalein	Cell division control protein 2 homolog	cdk-1
XQ1	baicalein	G2/mitotic-specific cyclin-B1	CCNB1
XQ1	baicalein	Myeloperoxidase	MPO
XQ1	baicalein	Aryl hydrocarbon receptor	AHR
XQ1	baicalein	Insulin-like growth factor II	IGF2
XQ1	baicalein	Cytochrome c	CYCS
XQ1	baicalein	Nuclear factor of activated T-cells, cytoplasmic 1	NFATC1
XQ1	baicalein	Tudor domain-containing protein 7	TDRD7
XQ1	baicalein	Egl nine homolog 1	EGLN1
XQ1	baicalein	NADPH oxidase 5	NOX5
XQ1	baicalein	Fatty acid-binding protein, epidermal	FABP5
XQ1	baicalein	Apolipoprotein D	APOD
HQ4	5,7,2,5-tetrahydroxy-8,6-dimethoxyflavone	Nitric oxide synthase, inducible	NOS2
HQ4	5,7,2,5-tetrahydroxy-8,6-dimethoxyflavone	Prothrombin	F2
HQ4	5,7,2,5-tetrahydroxy-8,6-dimethoxyflavone	Androgen receptor	AR

HQ4	5,7,2,5-tetrahydroxy-8,6-dimethoxyflavone	Coagulation factor X	F10
HQ4	5,7,2,5-tetrahydroxy-8,6-dimethoxyflavone	Prostaglandin G/H synthase 2	PTGS2
HQ4	5,7,2,5-tetrahydroxy-8,6-dimethoxyflavone	Tyrosine-protein phosphatase non-receptor type 1	PTPN1
HQ4	5,7,2,5-tetrahydroxy-8,6-dimethoxyflavone	DNA topoisomerase 2-alpha	TOP2A
HQ4	5,7,2,5-tetrahydroxy-8,6-dimethoxyflavone	Dipeptidyl peptidase 4	DPP4
HQ4	5,7,2,5-tetrahydroxy-8,6-dimethoxyflavone	Glycogen phosphorylase, muscle form	PYGM
HQ4	5,7,2,5-tetrahydroxy-8,6-dimethoxyflavone	Heat shock protein HSP 90-alpha	HSP90AA1
HQ4	5,7,2,5-tetrahydroxy-8,6-dimethoxyflavone	Trypsin-1	PRSS1
HQ4	5,7,2,5-tetrahydroxy-8,6-dimethoxyflavone	Nuclear receptor coactivator 2	NCOA2
HQ4	5,7,2,5-tetrahydroxy-8,6-dimethoxyflavone	Calcium-activated potassium channel subunit alpha 1	KCNMA1
HQ5	Carthamidin	Prostaglandin G/H synthase 1	PTGS1
HQ5	Carthamidin	Prostaglandin G/H synthase 2	PTGS2
HQ5	Carthamidin	Heat shock protein HSP 90-alpha	HSP90AA1
HQ5	Carthamidin	cAMP-dependent protein kinase catalytic subunit alpha	PRKACA

HQ6	Dihydrobaicalin_qt	Prostaglandin G/H synthase 1	PTGS1
HQ6	Dihydrobaicalin_qt	Prostaglandin G/H synthase 2	PTGS2
HQ6	Dihydrobaicalin_qt	Heat shock protein HSP 90-alpha	HSP90AA1
HQ6	Dihydrobaicalin_qt	cAMP-dependent protein kinase catalytic subunit alpha	PRKACA
QS	Eriodyctiol (flavanone)	Prostaglandin G/H synthase 1	PTGS1
QS	Eriodyctiol (flavanone)	Prostaglandin G/H synthase 2	PTGS2
QS	Eriodyctiol (flavanone)	Heat shock protein HSP 90-alpha	HSP90AA1
QS	Eriodyctiol (flavanone)	cAMP-dependent protein kinase catalytic subunit alpha	PRKACA
QS	Eriodyctiol (flavanone)	Nuclear receptor coactivator 2	NCOA2
QS	Eriodyctiol (flavanone)	Phosphatidylinositol-4,5-bisphosphate 3-kinase catalytic subunit gamma isoform	PIK3CG
QS	Eriodyctiol (flavanone)	Glycogen phosphorylase, muscle form	PYGM
HQ8	Salvigenin	Nitric oxide synthase, inducible	NOS2
HQ8	Salvigenin	Prostaglandin G/H synthase 1	PTGS1
HQ8	Salvigenin	Prothrombin	F2
HQ8	Salvigenin	Sodium channel protein type 5 subunit alpha	SCN5A
HQ8	Salvigenin	Coagulation factor X	F10
HQ8	Salvigenin	Prostaglandin G/H synthase 2	PTGS2
HQ8	Salvigenin	Nitric-oxide synthase, endothelial	NOS3
HQ8	Salvigenin	Retinoic acid receptor RXR-alpha	RXRA
HQ8	Salvigenin	Acetylcholinesterase	ACHE
HQ8	Salvigenin	Alpha-1B adrenergic receptor	ADRA1B
HQ8	Salvigenin	Beta-2 adrenergic receptor	ADRB2
HQ8	Salvigenin	Dipeptidyl peptidase 4	DPP4
HQ8	Salvigenin	Heat shock protein HSP 90-alpha	HSP90AA1
HQ8	Salvigenin	Trypsin-1	PRSS1
HQ8	Salvigenin	Nuclear receptor coactivator 2	NCOA2
HQ8	Salvigenin	Coagulation factor VII	F7
HQ9	5,2',6'-Trihydroxy-7,8-dimethoxyflavone	Nitric oxide synthase, inducible	NOS2
HQ9	5,2',6'-Trihydroxy-7,8-dimethoxyflavone	Prostaglandin G/H synthase 1	PTGS1
HQ9	5,2',6'-Trihydroxy-7,8-dimethoxyflavone	Androgen receptor	AR
HQ9	5,2',6'-Trihydroxy-7,8-dimethoxyflavone	Sodium channel protein type 5 subunit alpha	SCN5A

HQ9	5,2',6'-Trihydroxy-7,8-dimethoxyflavone	Coagulation factor X	F10
HQ9	5,2',6'-Trihydroxy-7,8-dimethoxyflavone	Prostaglandin G/H synthase 2	PTGS2
HQ9	5,2',6'-Trihydroxy-7,8-dimethoxyflavone	DNA topoisomerase 2-alpha	TOP2A
HQ9	5,2',6'-Trihydroxy-7,8-dimethoxyflavone	Estrogen receptor beta	ESR2
HQ9	5,2',6'-Trihydroxy-7,8-dimethoxyflavone	Dipeptidyl peptidase 4	DPP4
HQ9	5,2',6'-Trihydroxy-7,8-dimethoxyflavone	Heat shock protein HSP 90-alpha	HSP90AA1
HQ9	5,2',6'-Trihydroxy-7,8-dimethoxyflavone	Phosphatidylinositol-4,5-bisphosphate 3-kinase catalytic subunit gamma isoform	PIK3CG
HQ9	5,2',6'-Trihydroxy-7,8-dimethoxyflavone	Serine/threonine-protein kinase Chk1	CHEK1
HQ9	5,2',6'-Trihydroxy-7,8-dimethoxyflavone	Trypsin-1	PRSS1
HQ9	5,2',6'-Trihydroxy-7,8-dimethoxyflavone	Nuclear receptor coactivator 2	NCOA2
HQ9	5,2',6'-Trihydroxy-7,8-dimethoxyflavone	Calcium-activated potassium channel subunit alpha 1	KCNMA1
HQ10	5,7,2',6'-Tetrahydroxyflavone	Prostaglandin G/H synthase 1	PTGS1
HQ10	5,7,2',6'-Tetrahydroxyflavone	Androgen receptor	AR
HQ10	5,7,2',6'-Tetrahydroxyflavone	Prostaglandin G/H synthase 2	PTGS2

HQ10	5,7,2',6'-Tetrahydroxyflavone	Dipeptidyl peptidase 4	DPP4
HQ10	5,7,2',6'-Tetrahydroxyflavone	Heat shock protein HSP 90-alpha	HSP90AA1
HQ10	5,7,2',6'-Tetrahydroxyflavone	cAMP-dependent protein kinase catalytic subunit alpha	PRKACA
HQ11	Skullcapflavone II	Nitric oxide synthase, inducible	NOS2
HQ11	Skullcapflavone II	Prostaglandin G/H synthase 1	PTGS1
HQ11	Skullcapflavone II	Prothrombin	F2
HQ11	Skullcapflavone II	Potassium voltage-gated channel subfamily H member 2	KCNH2
HQ11	Skullcapflavone II	Androgen receptor	AR
HQ11	Skullcapflavone II	Sodium channel protein type 5 subunit alpha	SCN5A
HQ11	Skullcapflavone II	Coagulation factor X	F10
HQ11	Skullcapflavone II	Prostaglandin G/H synthase 2	PTGS2
HQ11	Skullcapflavone II	Nitric-oxide synthase, endothelial	NOS3
HQ11	Skullcapflavone II	Coagulation factor VII	F7
HQ11	Skullcapflavone II	Vascular endothelial growth factor receptor 2	KDR
HQ11	Skullcapflavone II	Voltage-dependent calcium channel subunit alpha-2/delta-1	CACNA2D1
HQ11	Skullcapflavone II	DNA topoisomerase 2-alpha	TOP2A
HQ11	Skullcapflavone II	Dipeptidyl peptidase 4	DPP4
HQ11	Skullcapflavone II	Heat shock protein HSP 90-alpha	HSP90AA1
HQ11	Skullcapflavone II	Trypsin-1	PRSS1
HQ11	Skullcapflavone II	Nuclear receptor coactivator 2	NCOA2
HQ11	Skullcapflavone II	Nuclear receptor coactivator 1	NCOA1
HQ11	Skullcapflavone II	Calcium-activated potassium channel subunit alpha 1	KCNMA1
HQ12	oroxylin a	Nitric oxide synthase, inducible	NOS2
HQ12	oroxylin a	Prostaglandin G/H synthase 1	PTGS1
HQ12	oroxylin a	Androgen receptor	AR
HQ12	oroxylin a	Sodium channel protein type 5 subunit alpha	SCN5A
HQ12	oroxylin a	Prostaglandin G/H synthase 2	PTGS2
HQ12	oroxylin a	Retinoic acid receptor RXR-alpha	RXRA
HQ12	oroxylin a	cGMP-inhibited 3',5'-cyclic phosphodiesterase A	PDE3A
HQ12	oroxylin a	Alpha-1B adrenergic receptor	ADRA1B

HQ12	oroxylin a	Beta-2 adrenergic receptor	ADRB2
HQ12	oroxylin a	Dipeptidyl peptidase 4	DPP4
HQ12	oroxylin a	Heat shock protein HSP 90-alpha	HSP90AA1
HQ12	oroxylin a	Phosphatidylinositol-4,5-bisphosphate 3-kinase catalytic subunit gamma isoform	PIK3CG
HQ12	oroxylin a	cAMP-dependent protein kinase catalytic subunit alpha	PRKACA
HQ12	oroxylin a	Trypsin-1	PRSS1
HQ12	oroxylin a	Nuclear receptor coactivator 1	NCOA1
HQ12	oroxylin a	Nuclear receptor coactivator 2	NCOA2
HQ12	oroxylin a	cAMP-dependent protein kinase inhibitor alpha	PKIA
HQ12	oroxylin a	Apoptosis regulator Bcl-2	BCL2
HQ12	oroxylin a	Interleukin-6	IL6
HQ12	oroxylin a	Caspase-3	CASP3
HQ12	oroxylin a	Cell division control protein 2 homolog	cdk-1
HQ12	oroxylin a	G2/mitotic-specific cyclin-B1	CCNB1
HQ12	oroxylin a	Cytochrome P450 2C9	CYP2C9
HQ13	Panicolin	Nitric oxide synthase, inducible	NOS2
HQ13	Panicolin	Prostaglandin G/H synthase 1	PTGS1
HQ13	Panicolin	Androgen receptor	AR
HQ13	Panicolin	Sodium channel protein type 5 subunit alpha	SCN5A
HQ13	Panicolin	Prostaglandin G/H synthase 2	PTGS2
HQ13	Panicolin	Estrogen receptor beta	ESR2
HQ13	Panicolin	Dipeptidyl peptidase 4	DPP4
HQ13	Panicolin	Heat shock protein HSP 90-alpha	HSP90AA1
HQ13	Panicolin	Serine/threonine-protein kinase Chk1	CHEK1
HQ13	Panicolin	Trypsin-1	PRSS1
HQ13	Panicolin	Phosphatidylinositol-4,5-bisphosphate 3-kinase catalytic subunit gamma isoform	PIK3CG
HQ13	Panicolin	Nuclear receptor coactivator 1	NCOA1
HQ14	5,7,4'-Trihydroxy-8-methoxyflavone	Nitric oxide synthase, inducible	NOS2
HQ14	5,7,4'-Trihydroxy-8-methoxyflavone	Prostaglandin G/H synthase 1	PTGS1
HQ14	5,7,4'-Trihydroxy-8-methoxyflavone	Estrogen receptor	ESR1
HQ14	5,7,4'-Trihydroxy-8-methoxyflavone	Androgen receptor	AR
HQ14	5,7,4'-Trihydroxy-8-methoxyflavone	Peroxisome proliferator-activated receptor gamma	PPARG
HQ14	5,7,4'-Trihydroxy-8-methoxyflavone	Prostaglandin G/H synthase 2	PTGS2
HQ14	5,7,4'-Trihydroxy-8-methoxyflavone	Dipeptidyl peptidase 4	DPP4
HQ14	5,7,4'-Trihydroxy-8-methoxyflavone	Glycogen phosphorylase, muscle form	PYGM

HQ14	5,7,4'-Trihydroxy-8-methoxyflavone	Mitogen-activated protein kinase 14	MAPK14
HQ14	5,7,4'-Trihydroxy-8-methoxyflavone	Glycogen synthase kinase-3 beta	GSK3B
HQ14	5,7,4'-Trihydroxy-8-methoxyflavone	Heat shock protein HSP 90-alpha	HSP90AA1
HQ14	5,7,4'-Trihydroxy-8-methoxyflavone	Phosphatidylinositol-4,5-bisphosphate 3-kinase catalytic subunit gamma isoform	PIK3CG
HQ14	5,7,4'-Trihydroxy-8-methoxyflavone	Serine/threonine-protein kinase Chk1	CHEK1
HQ14	5,7,4'-Trihydroxy-8-methoxyflavone	Trypsin-1	PRSS1
HQ14	5,7,4'-Trihydroxy-8-methoxyflavone	Nuclear receptor coactivator 2	NCOA2
HQ14	5,7,4'-Trihydroxy-8-methoxyflavone	cAMP-dependent protein kinase catalytic subunit alpha	PRKACA
HQ15	NEOBAICALEIN	Nitric oxide synthase, inducible	NOS2
HQ15	NEOBAICALEIN	Prothrombin	F2
HQ15	NEOBAICALEIN	Potassium voltage-gated channel subfamily H member 2	KCNH2
HQ15	NEOBAICALEIN	Estrogen receptor	ESR1
HQ15	NEOBAICALEIN	Androgen receptor	AR
HQ15	NEOBAICALEIN	Sodium channel protein type 5 subunit alpha	SCN5A
HQ15	NEOBAICALEIN	Peroxisome proliferator-activated receptor gamma	PPARG
HQ15	NEOBAICALEIN	Coagulation factor X	F10
HQ15	NEOBAICALEIN	Prostaglandin G/H synthase 2	PTGS2
HQ15	NEOBAICALEIN	Coagulation factor VII	F7
HQ15	NEOBAICALEIN	Tyrosine-protein phosphatase non-receptor type 1	PTPN1
HQ15	NEOBAICALEIN	DNA topoisomerase 2-alpha	TOP2A
HQ15	NEOBAICALEIN	Estrogen receptor beta	ESR2
HQ15	NEOBAICALEIN	Dipeptidyl peptidase 4	DPP4
HQ15	NEOBAICALEIN	Glycogen phosphorylase, muscle form	PYGM
HQ15	NEOBAICALEIN	Glycogen synthase kinase-3 beta	GSK3B
HQ15	NEOBAICALEIN	Heat shock protein HSP 90-alpha	HSP90AA1
HQ15	NEOBAICALEIN	Serine/threonine-protein kinase Chk1	CHEK1
HQ15	NEOBAICALEIN	Trypsin-1	PRSS1
HQ15	NEOBAICALEIN	Nuclear receptor coactivator 2	NCOA2
HQ15	NEOBAICALEIN	Calcium-activated potassium channel subunit alpha 1	KCNMA1
HQ16	DIHYDROOROXYLIN	Prostaglandin G/H synthase 1	PTGS1
HQ16	DIHYDROOROXYLIN	Sodium channel protein type 5 subunit alpha	SCN5A
HQ16	DIHYDROOROXYLIN	Prostaglandin G/H synthase 2	PTGS2

HQ16	DIHYDROOROXYLIN	Retinoic acid receptor RXR-alpha	RXRA
HQ16	DIHYDROOROXYLIN	cGMP-inhibited 3',5'-cyclic phosphodiesterase A	PDE3A
HQ16	DIHYDROOROXYLIN	Alpha-1B adrenergic receptor	ADRA1B
HQ16	DIHYDROOROXYLIN	Beta-2 adrenergic receptor	ADRB2
HQ16	DIHYDROOROXYLIN	Heat shock protein HSP 90-alpha	HSP90AA1
HQ16	DIHYDROOROXYLIN	cAMP-dependent protein kinase catalytic subunit alpha	PRKACA
HQ16	DIHYDROOROXYLIN	Nuclear receptor coactivator 1	NCOA1
XQM	beta-sitosterol	Progesterone receptor	PGR
XQM	beta-sitosterol	Nuclear receptor coactivator 2	NCOA2
XQM	beta-sitosterol	Prostaglandin G/H synthase 1	PTGS1
XQM	beta-sitosterol	Prostaglandin G/H synthase 2	PTGS2
XQM	beta-sitosterol	Heat shock protein HSP 90-alpha	HSP90AA1
XQM	beta-sitosterol	Phosphatidylinositol-4,5-bisphosphate 3-kinase catalytic subunit gamma isoform	PIK3CG
XQM	beta-sitosterol	Potassium voltage-gated channel subfamily H member 2	KCNH2
XQM	beta-sitosterol	cAMP-dependent protein kinase catalytic subunit alpha	PRKACA
XQM	beta-sitosterol	D(1A) dopamine receptor	DRD1
XQM	beta-sitosterol	Muscarinic acetylcholine receptor M3	CHRM3
XQM	beta-sitosterol	Muscarinic acetylcholine receptor M1	CHRM1
XQM	beta-sitosterol	Sodium channel protein type 5 subunit alpha	SCN5A
XQM	beta-sitosterol	Gamma-aminobutyric-acid receptor subunit alpha-2	GABRA2
XQM	beta-sitosterol	Muscarinic acetylcholine receptor M4	CHRM4
XQM	beta-sitosterol	cGMP-inhibited 3',5'-cyclic phosphodiesterase A	PDE3A
XQM	beta-sitosterol	5-hydroxytryptamine 2A receptor	HTR2A
XQM	beta-sitosterol	Gamma-aminobutyric-acid receptor subunit alpha-5	GABRA5
XQM	beta-sitosterol	Alpha-1A adrenergic receptor	ADRA1A
XQM	beta-sitosterol	Gamma-aminobutyric-acid receptor subunit alpha-3	GABRA3
XQM	beta-sitosterol	Muscarinic acetylcholine receptor M2	CHRM2
XQM	beta-sitosterol	Alpha-1B adrenergic receptor	ADRA1B
XQM	beta-sitosterol	Beta-2 adrenergic receptor	ADRB2
XQM	beta-sitosterol	Neuronal acetylcholine receptor subunit alpha-2	CHRNA2
XQM	beta-sitosterol	Sodium-dependent serotonin transporter	SLC6A4
XQM	beta-sitosterol	Mu-type opioid receptor	OPRM1
XQM	beta-sitosterol	Gamma-aminobutyric-acid receptor subunit alpha-1	GABRA1
XQM	beta-sitosterol	Neuronal acetylcholine receptor subunit alpha-7	CHRNA7
XQM	beta-sitosterol	Apoptosis regulator Bcl-2	BCL2
XQM	beta-sitosterol	Apoptosis regulator BAX	BAX
XQM	beta-sitosterol	Caspase-9	CASP9
XQM	beta-sitosterol	Transcription factor AP-1	JUN
XQM	beta-sitosterol	Caspase-3	CASP3
XQM	beta-sitosterol	Caspase-8	CASP8
XQM	beta-sitosterol	Protein kinase C alpha type	PRKCA

XQM	beta-sitosterol	Transforming growth factor beta-1	TGFB1
XQM	beta-sitosterol	Serum paraoxonase/arylesterase 1	PON1
XQM	beta-sitosterol	Microtubule-associated protein 2	MAP2
CJQ	sitosterol	Progesterone receptor	PGR
CJQ	sitosterol	Nuclear receptor coactivator 2	NCOA2
CJQ	sitosterol	Mineralocorticoid receptor	NR3C2
HQ17	Norwogonin	Nitric oxide synthase, inducible	NOS2
HQ17	Norwogonin	Prostaglandin G/H synthase 1	PTGS1
HQ17	Norwogonin	Androgen receptor	AR
HQ17	Norwogonin	Peroxisome proliferator-activated receptor gamma	PPARG
HQ17	Norwogonin	Prostaglandin G/H synthase 2	PTGS2
HQ17	Norwogonin	cGMP-inhibited 3',5'-cyclic phosphodiesterase A	PDE3A
HQ17	Norwogonin	Dipeptidyl peptidase 4	DPP4
HQ17	Norwogonin	Heat shock protein HSP 90-alpha	HSP90AA1
HQ17	Norwogonin	Phosphatidylinositol-4,5-bisphosphate 3-kinase catalytic subunit gamma isoform	PIK3CG
HQ17	Norwogonin	Serine/threonine-protein kinase Chk1	CHEK1
HQ17	Norwogonin	cAMP-dependent protein kinase catalytic subunit alpha	PRKACA
HQ18	5,2'-Dihydroxy-6,7,8-trimethoxyflavone	Nitric oxide synthase, inducible	NOS2
HQ18	5,2'-Dihydroxy-6,7,8-trimethoxyflavone	Prostaglandin G/H synthase 1	PTGS1
HQ18	5,2'-Dihydroxy-6,7,8-trimethoxyflavone	Prothrombin	F2
HQ18	5,2'-Dihydroxy-6,7,8-trimethoxyflavone	Potassium voltage-gated channel subfamily H member 2	KCNH2
HQ18	5,2'-Dihydroxy-6,7,8-trimethoxyflavone	Androgen receptor	AR
HQ18	5,2'-Dihydroxy-6,7,8-trimethoxyflavone	Sodium channel protein type 5 subunit alpha	SCN5A
HQ18	5,2'-Dihydroxy-6,7,8-trimethoxyflavone	Coagulation factor X	F10
HQ18	5,2'-Dihydroxy-6,7,8-trimethoxyflavone	Prostaglandin G/H synthase 2	PTGS2

HQ18	5,2'-Dihydroxy-6,7,8-trimethoxyflavone	Nitric-oxide synthase, endothelial	NOS3
HQ18	5,2'-Dihydroxy-6,7,8-trimethoxyflavone	Coagulation factor VII	F7
HQ18	5,2'-Dihydroxy-6,7,8-trimethoxyflavone	DNA topoisomerase 2-alpha	TOP2A
HQ18	5,2'-Dihydroxy-6,7,8-trimethoxyflavone	Estrogen receptor beta	ESR2
HQ18	5,2'-Dihydroxy-6,7,8-trimethoxyflavone	Dipeptidyl peptidase 4	DPP4
HQ18	5,2'-Dihydroxy-6,7,8-trimethoxyflavone	Peroxisome proliferator-activated receptor delta	PPARD
HQ18	5,2'-Dihydroxy-6,7,8-trimethoxyflavone	Heat shock protein HSP 90-alpha	HSP90AA1
HQ18	5,2'-Dihydroxy-6,7,8-trimethoxyflavone	Trypsin-1	PRSS1
HQ18	5,2'-Dihydroxy-6,7,8-trimethoxyflavone	Nuclear receptor coactivator 2	NCOA2
HQ18	5,2'-Dihydroxy-6,7,8-trimethoxyflavone	Vascular endothelial growth factor receptor 2	KDR
HQ18	5,2'-Dihydroxy-6,7,8-trimethoxyflavone	Nuclear receptor coactivator 1	NCOA1
HQ18	5,2'-Dihydroxy-6,7,8-trimethoxyflavone	Calcium-activated potassium channel subunit alpha 1	KCNMA1
HQ19	ent-Epicatechin	Prostaglandin G/H synthase 1	PTGS1
HQ19	ent-Epicatechin	Estrogen receptor	ESR1
HQ19	ent-Epicatechin	Prostaglandin G/H synthase 2	PTGS2
HQ19	ent-Epicatechin	Heat shock protein HSP 90-alpha	HSP90AA1
HQ19	ent-Epicatechin	Beta-lactamase	DPEP1
HQ19	ent-Epicatechin	cAMP-dependent protein kinase catalytic subunit alpha	PRKACA

XQ2	Stigmasterol	Progesterone receptor	PGR
XQ2	Stigmasterol	Mineralocorticoid receptor	NR3C2
XQ2	Stigmasterol	Nuclear receptor coactivator 2	NCOA2
XQ2	Stigmasterol	Retinoic acid receptor RXR-alpha	RXRA
XQ2	Stigmasterol	Nuclear receptor coactivator 1	NCOA1
XQ2	Stigmasterol	Prostaglandin G/H synthase 1	PTGS1
XQ2	Stigmasterol	Prostaglandin G/H synthase 2	PTGS2
XQ2	Stigmasterol	Alpha-2A adrenergic receptor	ADRA2A
XQ2	Stigmasterol	Sodium-dependent noradrenaline transporter	SLC6A2
XQ2	Stigmasterol	Sodium-dependent dopamine transporter	SLC6A3
XQ2	Stigmasterol	Beta-2 adrenergic receptor	ADRB2
XQ2	Stigmasterol	Urokinase-type plasminogen activator	PLAU
XQ2	Stigmasterol	Leukotriene A-4 hydrolase	LTA4H
XQ2	Stigmasterol	Amine oxidase [flavin-containing] B	MAOB
XQ2	Stigmasterol	Amine oxidase [flavin-containing] A	MAOA
XQ2	Stigmasterol	cAMP-dependent protein kinase catalytic subunit alpha	PRKACA
XQ2	Stigmasterol	Chymotrypsinogen B	CTRB1
XQ2	Stigmasterol	Muscarinic acetylcholine receptor M3	CHRM3
XQ2	Stigmasterol	Muscarinic acetylcholine receptor M1	CHRM1
XQ2	Stigmasterol	Beta-1 adrenergic receptor	ADRB1
XQ2	Stigmasterol	Sodium channel protein type 5 subunit alpha	SCN5A
XQ2	Stigmasterol	5-hydroxytryptamine 2A receptor	HTR2A
XQ2	Stigmasterol	Alpha-1A adrenergic receptor	ADRA1A
XQ2	Stigmasterol	Gamma-aminobutyric-acid receptor subunit alpha-3	GABRA3
XQ2	Stigmasterol	Muscarinic acetylcholine receptor M2	CHRM2
XQ2	Stigmasterol	Alpha-1B adrenergic receptor	ADRA1B
XQ2	Stigmasterol	Gamma-aminobutyric-acid receptor subunit alpha-1	GABRA1
XQ2	Stigmasterol	Neuronal acetylcholine receptor subunit alpha-7	CHRNA7
LQ	coptisine	Nitric oxide synthase, inducible	NOS2
LQ	coptisine	Prostaglandin G/H synthase 1	PTGS1
LQ	coptisine	Potassium voltage-gated channel subfamily H member 2	KCNH2
LQ	coptisine	Estrogen receptor	ESR1
LQ	coptisine	Androgen receptor	AR
LQ	coptisine	Sodium channel protein type 5 subunit alpha	SCN5A
LQ	coptisine	Prostaglandin G/H synthase 2	PTGS2
LQ	coptisine	Nitric-oxide synthase, endothelial	NOS3
LQ	coptisine	Trypsin-1	PRSS1
HQ20	bis[(2S)-2-ethylhexyl] benzene-1,2-dicarboxylate	Sodium channel protein type 5 subunit alpha	SCN5A
HQ21	Diop	Sodium channel protein type 5 subunit alpha	SCN5A
HQ21	Diop	Beta-2 adrenergic receptor	ADRB2
HQ21	Diop	Muscarinic acetylcholine receptor M3	CHRM3
HQ22	epiberberine	Nitric oxide synthase, inducible	NOS2
HQ22	epiberberine	Potassium voltage-gated channel subfamily H member 2	KCNH2
HQ22	epiberberine	Estrogen receptor	ESR1
HQ22	epiberberine	Androgen receptor	AR
HQ22	epiberberine	Prostaglandin G/H synthase 2	PTGS2
HQ22	epiberberine	Nitric-oxide synthase, endothelial	NOS3

HQ22	epiberberine	Retinoic acid receptor RXR-alpha	RXRA
HQ22	epiberberine	cAMP-dependent protein kinase catalytic subunit alpha	PRKACA
HQ22	epiberberine	Trypsin-1	PRSS1
HQ22	epiberberine	Nuclear receptor coactivator 2	NCOA2
HQ22	epiberberine	cAMP and cAMP-inhibited cGMP 3',5'-cyclic phosphodiesterase 10A	PDE10A
HQ23	Moslosooflavone	Nitric oxide synthase, inducible	NOS2
HQ23	Moslosooflavone	Prostaglandin G/H synthase 1	PTGS1
HQ23	Moslosooflavone	Prothrombin	F2
HQ23	Moslosooflavone	Androgen receptor	AR
HQ23	Moslosooflavone	Sodium channel protein type 5 subunit alpha	SCN5A
HQ23	Moslosooflavone	Peroxisome proliferator-activated receptor gamma	PPARG
HQ23	Moslosooflavone	Prostaglandin G/H synthase 2	PTGS2
HQ23	Moslosooflavone	Retinoic acid receptor RXR-alpha	RXRA
HQ23	Moslosooflavone	Estrogen receptor beta	ESR2
HQ23	Moslosooflavone	Gamma-aminobutyric-acid receptor subunit alpha-1	GABRA1
HQ23	Moslosooflavone	Dipeptidyl peptidase 4	DPP4
HQ23	Moslosooflavone	Mitogen-activated protein kinase 14	MAPK14
HQ23	Moslosooflavone	Glycogen synthase kinase-3 beta	GSK3B
HQ23	Moslosooflavone	Heat shock protein HSP 90-alpha	HSP90AA1
HQ23	Moslosooflavone	Phosphatidylinositol-4,5-bisphosphate 3-kinase catalytic subunit gamma isoform	PIK3CG
HQ23	Moslosooflavone	Serine/threonine-protein kinase Chk1	CHEK1
HQ23	Moslosooflavone	cAMP-dependent protein kinase catalytic subunit alpha	PRKACA
HQ23	Moslosooflavone	Trypsin-1	PRSS1
HQ23	Moslosooflavone	Nuclear receptor coactivator 1	NCOA1
HQ23	Moslosooflavone	Calcium-activated potassium channel subunit alpha 1	KCNMA1
HQ23	Moslosooflavone	Alpha-1B adrenergic receptor	ADRA1B
HQ23	Moslosooflavone	Beta-2 adrenergic receptor	ADRB2
HQ23	Moslosooflavone	Neuronal acetylcholine receptor subunit alpha-7	CHRNA7

HQ24	11,13-Eicosadienoic acid, methyl ester	Nuclear receptor coactivator 2	NCOA2
HQ25	5,7,4'-trihydroxy-6-methoxyflavanone	Prostaglandin G/H synthase 1	PTGS1
HQ25	5,7,4'-trihydroxy-6-methoxyflavanone	Prostaglandin G/H synthase 2	PTGS2
HQ25	5,7,4'-trihydroxy-6-methoxyflavanone	Carbonic anhydrase 2	CA2
HQ25	5,7,4'-trihydroxy-6-methoxyflavanone	Heat shock protein HSP 90-alpha	HSP90AA1
HQ25	5,7,4'-trihydroxy-6-methoxyflavanone	cAMP-dependent protein kinase catalytic subunit alpha	PRKACA
HQ26	5,7,4'-trihydroxy-8-methoxyflavanone	Prostaglandin G/H synthase 1	PTGS1
HQ26	5,7,4'-trihydroxy-8-methoxyflavanone	Prostaglandin G/H synthase 2	PTGS2
HQ26	5,7,4'-trihydroxy-8-methoxyflavanone	Carbonic anhydrase 2	CA2
HQ26	5,7,4'-trihydroxy-8-methoxyflavanone	Heat shock protein HSP 90-alpha	HSP90AA1
HQ26	5,7,4'-trihydroxy-8-methoxyflavanone	Phosphatidylinositol-4,5-bisphosphate 3-kinase catalytic subunit gamma isoform	PIK3CG
HQ26	5,7,4'-trihydroxy-8-methoxyflavanone	cAMP-dependent protein kinase catalytic subunit alpha	PRKACA
HQ7	rivularin	Nitric oxide synthase, inducible	NOS2
HQ7	rivularin	Prostaglandin G/H synthase 1	PTGS1
HQ7	rivularin	Prothrombin	F2
HQ7	rivularin	Potassium voltage-gated channel subfamily H member 2	KCNH2
HQ7	rivularin	Androgen receptor	AR
HQ7	rivularin	Sodium channel protein type 5 subunit alpha	SCN5A

HQ7	rivularin	Coagulation factor X	F10
HQ7	rivularin	Prostaglandin G/H synthase 2	PTGS2
HQ7	rivularin	Nitric-oxide synthase, endothelial	NOS3
HQ7	rivularin	Carbonic anhydrase 2	CA2
HQ7	rivularin	Coagulation factor VII	F7
HQ7	rivularin	Vascular endothelial growth factor receptor 2	KDR
HQ7	rivularin	Retinoic acid receptor RXR-alpha	RXRA
HQ7	rivularin	DNA topoisomerase 2-alpha	TOP2A
HQ7	rivularin	Estrogen receptor beta	ESR2
HQ7	rivularin	Dipeptidyl peptidase 4	DPP4
HQ7	rivularin	Heat shock protein HSP 90-alpha	HSP90AA1
HQ7	rivularin	Trypsin-1	PRSS1
HQ7	rivularin	Nuclear receptor coactivator 2	NCOA2
HQ7	rivularin	Nuclear receptor coactivator 1	NCOA1
HQ7	rivularin	Calcium-activated potassium channel subunit alpha 1	KCNMA1

Gentianae

Abbreviation	Molecule Name	Target Name	Genesym bol
LDC1	gentianidine	Prostaglandin G/H synthase 1	PTGS1
LDC1	gentianidine	Prothrombin	F2
LDC1	gentianidine	Muscarinic acetylcholine receptor M1	CHRM1
LDC1	gentianidine	Beta-1 adrenergic receptor	ADRB1
LDC1	gentianidine	Prostaglandin G/H synthase 2	PTGS2
LDC1	gentianidine	Nitric-oxide synthase, endothelial	NOS3
LDC1	gentianidine	Alpha-2A adrenergic receptor	ADRA2A
LDC1	gentianidine	Alpha-2C adrenergic receptor	ADRA2C
LDC1	gentianidine	Acetylcholinesterase	ACHE
LDC1	gentianidine	Alpha-1A adrenergic receptor	ADRA1A
LDC1	gentianidine	Muscarinic acetylcholine receptor M2	CHRM2
LDC1	gentianidine	Alpha-1B adrenergic receptor	ADRA1B
LDC1	gentianidine	Sodium-dependent dopamine transporter	SLC6A3
LDC1	gentianidine	Beta-2 adrenergic receptor	ADRB2
LDC1	gentianidine	Dipeptidyl peptidase 4	DPP4
LDC1	gentianidine	Beta-lactamase	DPEP1
LDC1	gentianidine	Amine oxidase [flavin-containing] B	MAOB
LDC1	gentianidine	Trypsin-1	PRSS1
LDC2	gentianine	Prostaglandin G/H synthase 1	PTGS1
LDC2	gentianine	Muscarinic acetylcholine receptor M3	CHRM3
LDC2	gentianine	Muscarinic acetylcholine receptor M1	CHRM1
LDC2	gentianine	Prostaglandin G/H synthase 2	PTGS2
LDC2	gentianine	Alpha-1B adrenergic receptor	ADRA1B
LDC2	gentianine	Sodium-dependent dopamine transporter	SLC6A3
LDC2	gentianine	Beta-2 adrenergic receptor	ADRB2
LDC2	gentianine	Gamma-aminobutyric-acid receptor subunit alpha-1	GABRA1
LDC2	gentianine	Amine oxidase [flavin-containing] B	MAOB
LDC2	gentianine	Trypsin-1	PRSS1
LDC2	gentianine	Beta-1 adrenergic receptor	ADRB1
LDC2	gentianine	Ribosyldihydronicotinamide dehydrogenase [quinone]	NQO2
LDC2	gentianine	Alpha-1A adrenergic receptor	ADRA1A
LDC2	gentianine	Sodium channel protein type 5 subunit alpha	SCN5A
LDC2	gentianine	Alpha-2A adrenergic receptor	ADRA2A
LDC2	gentianine	Leukotriene A-4 hydrolase	LTA4H
LDC2	gentianine	Neuronal acetylcholine receptor subunit alpha-7	CHRNA7
LDC3	gentianal	Ribosyldihydronicotinamide dehydrogenase [quinone]	NQO2
LDC3	gentianal	Gamma-aminobutyric-acid receptor subunit alpha-2	GABRA2
LDC3	gentianal	Gamma-aminobutyric-acid receptor subunit alpha-1	GABRA1
LDC3	gentianal	Trypsin-1	PRSS1
LDC3	gentianal	Glutamate receptor 2	GRIA2

Taraxaci herba

Abbreviation	Molecule Name	Target Name	Genesymbol
PGY1	2-furfural	Lysozyme	LYZ
PGY2	4-hydroxy-4-methyl-2-pentanone	Prostaglandin G/H synthase 1	PTGS1
PGY2	4-hydroxy-4-methyl-2-pentanone	4-aminobutyrate aminotransferase, mitochondrial	ABAT
PGY2	4-hydroxy-4-methyl-2-pentanone	Gamma-aminobutyric-acid receptor subunit alpha-2	GABRA2
PGY2	4-hydroxy-4-methyl-2-pentanone	Glycine receptor subunit alpha-1	GLRA1
PGY2	4-hydroxy-4-methyl-2-pentanone	Sodium-dependent noradrenaline transporter	SLC6A2
PGY2	4-hydroxy-4-methyl-2-pentanone	Gamma-aminobutyric-acid receptor subunit alpha-3	GABRA3
PGY2	4-hydroxy-4-methyl-2-pentanone	Aspartate aminotransferase, cytoplasmic	GOT1
PGY2	4-hydroxy-4-methyl-2-pentanone	Branched-chain-amino-acid aminotransferase, mitochondrial	BCAT2
PGY2	4-hydroxy-4-methyl-2-pentanone	Gamma-aminobutyric-acid receptor subunit alpha-1	GABRA1
PGY2	4-hydroxy-4-methyl-2-pentanone	Cathepsin D	CTSD
PGY2	4-hydroxy-4-methyl-2-pentanone	Cholinesterase	BCHE
PGY2	4-hydroxy-4-methyl-2-pentanone	Phosphotriesterase	PTER
PGY2	4-hydroxy-4-methyl-2-pentanone	Ornithine carbamoyltransferase, mitochondrial	OTC
PGY2	4-hydroxy-4-methyl-2-pentanone	Aspartate aminotransferase, mitochondrial	GOT2
PGY2	4-hydroxy-4-methyl-2-pentanone	Gamma-aminobutyric-acid receptor subunit alpha-6	GABRA6
PGY2	4-hydroxy-4-methyl-2-pentanone	Glycine amidinotransferase, mitochondrial	GATM
PGY2	4-hydroxy-4-methyl-2-pentanone	Triosephosphate isomerase	TPI1

PGY2	4-hydroxy-4-methyl-2-pentanone	NAD-dependent malic enzyme, mitochondrial	ME2
PGY2	4-hydroxy-4-methyl-2-pentanone	Trypsin-3	PRSS3
PGY2	4-hydroxy-4-methyl-2-pentanone	Gamma-aminobutyric-acid receptor subunit alpha-5	GABRA5
PGY2	4-hydroxy-4-methyl-2-pentanone	Tyrosine-protein phosphatase non-receptor type 1	PTPN1
PGY2	4-hydroxy-4-methyl-2-pentanone	Choline dehydrogenase, mitochondrial	CHDH
PGY3	benzenecarboxylic acid	Amine oxidase [flavin-containing] B	MAOB
PGY3	benzenecarboxylic acid	Lysozyme	LYZ
PGY3	benzenecarboxylic acid	Group IIE secretory phospholipase A2	PLA2G2E
PGY3	benzenecarboxylic acid	Trypsin-3	PRSS3
PGY3	benzenecarboxylic acid	D-amino-acid oxidase	DAO
PGY3	benzenecarboxylic acid	Muscarinic acetylcholine receptor M3	CHRM3
PGY3	benzenecarboxylic acid	Prostaglandin G/H synthase 2	PTGS2
PGY3	benzenecarboxylic acid	Gamma-aminobutyric-acid receptor subunit alpha-2	GABRA2
PGY3	benzenecarboxylic acid	Retinoic acid receptor RXR-alpha	RXRA
PGY3	benzenecarboxylic acid	Gamma-aminobutyric-acid receptor subunit alpha-1	GABRA1
PGY3	benzenecarboxylic acid	Nuclear receptor coactivator 2	NCOA2
PGY4	caffeic acid	Prostaglandin G/H synthase 1	PTGS1
PGY4	caffeic acid	Beta-1 adrenergic receptor	ADRB1
PGY4	caffeic acid	Prostaglandin G/H synthase 2	PTGS2
PGY4	caffeic acid	Alpha-2A adrenergic receptor	ADRA2A
PGY4	caffeic acid	Alpha-2C adrenergic receptor	ADRA2C
PGY4	caffeic acid	Beta-2 adrenergic receptor	ADRB2
PGY4	caffeic acid	Amine oxidase [flavin-containing] B	MAOB
PGY4	caffeic acid	Amine oxidase [flavin-containing] A	MAOA
PGY4	caffeic acid	Chymotrypsinogen B	CTRB1
PGY5	esculetin	Apoptosis regulator Bcl-2	BCL2
PGY5	esculetin	Cyclin-dependent kinase inhibitor 1	CDKN1A
PGY5	esculetin	Retinoblastoma-associated protein	RB1
PGY5	esculetin	Cell division protein kinase 4	CDK4
PGY5	esculetin	Caspase-3	CASP3
PGY5	esculetin	Interstitial collagenase	MMP1
PGY5	esculetin	Stromelysin-1	MMP3
PGY5	esculetin	Transcription factor E2F1	E2F1
PGY6	ethyl caffeate	Prostaglandin G/H synthase 1	PTGS1

PGY6	ethyl caffeate	Prostaglandin G/H synthase 2	PTGS2
PGY6	ethyl caffeate	Beta-2 adrenergic receptor	ADRB2
PGY6	ethyl caffeate	Amine oxidase [flavin-containing] B	MAOB
PGY7	methyl caffeate	Prostaglandin G/H synthase 1	PTGS1
PGY7	methyl caffeate	Prostaglandin G/H synthase 2	PTGS2
PGY7	methyl caffeate	Sodium-dependent dopamine transporter	SLC6A3
PGY7	methyl caffeate	Beta-2 adrenergic receptor	ADRB2
PGY7	methyl caffeate	Leukotriene A-4 hydrolase	LTA4H
PGY7	methyl caffeate	Amine oxidase [flavin-containing] B	MAOB
PGY7	methyl caffeate	Amine oxidase [flavin-containing] A	MAOA
PGY8	myristic acid	Prostaglandin G/H synthase 1	PTGS1
PGY8	myristic acid	Prostaglandin G/H synthase 2	PTGS2
PGY8	myristic acid	Cholinesterase	BCHE
PGY8	myristic acid	Phospholipase A2	PLA2G1B
PGY8	myristic acid	Nuclear receptor coactivator 2	NCOA2
PGY8	myristic acid	Nuclear receptor coactivator 1	NCOA1
PGY8	myristic acid	Phosphatidylcholine-sterol acyltransferase	LCAT
PGY14	palmitic acid	Cathepsin D	CTSD
PGY14	palmitic acid	Prostaglandin G/H synthase 1	PTGS1
PGY14	palmitic acid	Prostaglandin G/H synthase 2	PTGS2
PGY14	palmitic acid	Rhodopsin	RHO
PGY14	palmitic acid	Nuclear receptor coactivator 2	NCOA2
PGY14	palmitic acid	Apoptosis regulator Bcl-2	BCL2
PGY14	palmitic acid	Interleukin-10	IL10
PGY14	palmitic acid	Tumor necrosis factor	TNF
PGY14	palmitic acid	Phosphatidylinositol-3,4,5-trisphosphate 3-phosphatase and dual-specificity protein phosphatase PTEN	PTEN
PGY14	palmitic acid	Solute carrier family 22 member 5	SLC22A5
PGY14	palmitic acid	Choline-phosphate cytidylyltransferase A	PCYT1A
PGY9	phenylacetic acid	Sodium-dependent dopamine transporter	SLC6A3
PGY9	phenylacetic acid	Amine oxidase [flavin-containing] B	MAOB
PGY9	phenylacetic acid	Amine oxidase [flavin-containing] A	MAOA
PGY9	phenylacetic acid	Lysozyme	LYZ
PGY9	phenylacetic acid	Trypsin-3	PRSS3
PGY10	protocatechuic aldehyde	Lysozyme	LYZ
PGY10	protocatechuic aldehyde	Muscarinic acetylcholine receptor M3	CHRM3
PGY10	protocatechuic aldehyde	Muscarinic acetylcholine receptor M1	CHRM1
PGY10	protocatechuic aldehyde	Gamma-aminobutyric-acid receptor subunit alpha-2	GABRA2
PGY10	protocatechuic aldehyde	Gamma-aminobutyric-acid receptor subunit alpha-3	GABRA3
PGY10	protocatechuic aldehyde	Muscarinic acetylcholine receptor M2	CHRM2
PGY10	protocatechuic aldehyde	Gamma-aminobutyric-acid receptor subunit alpha-1	GABRA1
PGY10	protocatechuic aldehyde	Neuronal acetylcholine receptor subunit alpha-7	CHRNA7
PGY10	protocatechuic aldehyde	Gamma-aminobutyric-acid receptor subunit alpha-6	GABRA6
PGY10	protocatechuic aldehyde	Ornithine decarboxylase	ODC1

CPL	quercetin	Prostaglandin G/H synthase 1	PTGS1
CPL	quercetin	Androgen receptor	AR
CPL	quercetin	Peroxisome proliferator-activated receptor gamma	PPARG
CPL	quercetin	Prostaglandin G/H synthase 2	PTGS2
CPL	quercetin	Heat shock protein HSP 90-alpha	HSP90AA1
CPL	quercetin	Phosphatidylinositol-4,5-bisphosphate 3-kinase catalytic subunit gamma isoform	PIK3CG
CPL	quercetin	Nuclear receptor coactivator 2	NCOA2
CPL	quercetin	Dipeptidyl peptidase 4	DPP4
CPL	quercetin	Trypsin-1	PRSS1
CPL	quercetin	DNA topoisomerase 2-alpha	TOP2A
CPL	quercetin	Prothrombin	F2
CPL	quercetin	Potassium voltage-gated channel subfamily H member 2	KCNH2
CPL	quercetin	Sodium channel protein type 5 subunit alpha	SCN5A
CPL	quercetin	Coagulation factor X	F10
CPL	quercetin	Beta-2 adrenergic receptor	ADRB2
CPL	quercetin	Stromelysin-1	MMP3
CPL	quercetin	cAMP-dependent protein kinase catalytic subunit alpha	PRKACA
CPL	quercetin	Coagulation factor VII	F7
CPL	quercetin	Retinoic acid receptor RXR-alpha	RXRA
CPL	quercetin	Acetylcholinesterase	ACHE
CPL	quercetin	Gamma-aminobutyric-acid receptor subunit alpha-1	GABRA1
CPL	quercetin	Amine oxidase [flavin-containing] B	MAOB
CPL	quercetin	Transcription factor p65	RELA
CPL	quercetin	Epidermal growth factor receptor	EGFR
CPL	quercetin	RAC-alpha serine/threonine-protein kinase	AKT1
CPL	quercetin	G1/S-specific cyclin-D1	CCND1
CPL	quercetin	Apoptosis regulator Bcl-2	BCL2
CPL	quercetin	Bcl-2-like protein 1	BCL2L1
CPL	quercetin	Proto-oncogene c-Fos	FOS
CPL	quercetin	Cyclin-dependent kinase inhibitor 1	CDKN1A
CPL	quercetin	Eukaryotic translation initiation factor 6	EIF6
CPL	quercetin	Apoptosis regulator BAX	BAX
CPL	quercetin	Caspase-9	CASP9
CPL	quercetin	Urokinase-type plasminogen activator	PLAU
CPL	quercetin	72 kDa type IV collagenase	MMP2
CPL	quercetin	Matrix metalloproteinase-9	MMP9
CPL	quercetin	Mitogen-activated protein kinase 1	MAPK1
CPL	quercetin	Interleukin-10	IL10
CPL	quercetin	Retinoblastoma-associated protein	RB1
CPL	quercetin	Tumor necrosis factor	TNF
CPL	quercetin	Transcription factor AP-1	JUN
CPL	quercetin	Interleukin-6	IL6
CPL	quercetin	Activator of 90 kDa heat shock protein ATPase homolog 1	AHSA1
CPL	quercetin	Caspase-3	CASP3
CPL	quercetin	Cellular tumor antigen p53	TP53
CPL	quercetin	ETS domain-containing protein Elk-1	ELK1
CPL	quercetin	NF-kappa-B inhibitor alpha	NFKBIA

CPL	quercetin	Ornithine decarboxylase	ODC1
CPL	quercetin	Xanthine dehydrogenase/oxidase	XDH
CPL	quercetin	Caspase-8	CASP8
CPL	quercetin	DNA topoisomerase 1	TOP1
CPL	quercetin	RAF proto-oncogene serine/threonine-protein kinase	RAF1
CPL	quercetin	Superoxide dismutase [Cu-Zn]	SOD1
CPL	quercetin	Protein kinase C alpha type	PRKCA
CPL	quercetin	Interstitial collagenase	MMP1
CPL	quercetin	Hypoxia-inducible factor 1-alpha	HIF1A
CPL	quercetin	Signal transducer and activator of transcription 1-alpha/beta	STAT1
CPL	quercetin	Protein CBFA2T1	RUNX1T1
CPL	quercetin	78 kDa glucose-regulated protein	HSPA5
CPL	quercetin	Receptor tyrosine-protein kinase erbB-2	ERBB2
CPL	quercetin	Peroxisome proliferator-activated receptor gamma	PPARG
CPL	quercetin	Acetyl-CoA carboxylase 1	ACACA
CPL	quercetin	Heme oxygenase 1	HMOX1
CPL	quercetin	Cytochrome P450 3A4	CYP3A4
CPL	quercetin	Caveolin-1	CAV1
CPL	quercetin	Myc proto-oncogene protein	MYC
CPL	quercetin	Tissue factor	F3
CPL	quercetin	Gap junction alpha-1 protein	GJA1
CPL	quercetin	Cytochrome P450 1A1	CYP1A1
CPL	quercetin	Intercellular adhesion molecule 1	ICAM1
CPL	quercetin	Interleukin-1 beta	IL1B
CPL	quercetin	Small inducible cytokine A2	CCL2
CPL	quercetin	E-selectin	SELE
CPL	quercetin	Vascular cell adhesion protein 1	VCAM1
CPL	quercetin	Prostaglandin E2 receptor, EP3 subtype	PTGER3
CPL	quercetin	Interleukin-8	CXCL8
CPL	quercetin	Protein kinase C beta type	PRKCB
CPL	quercetin	Baculoviral IAP repeat-containing protein 5	BIRC5
CPL	quercetin	Dual oxidase 2	DUOX2
CPL	quercetin	Nitric oxide synthase, endothelial	NOS3
CPL	quercetin	Heat shock protein beta-1	HSPB1
CPL	quercetin	Transforming growth factor beta-1	TGFB1
CPL	quercetin	Maltase-glucoamylase, intestinal	MGAM
CPL	quercetin	Interleukin-2	IL2
CPL	quercetin	Nuclear receptor subfamily 1 group I member 2	NR1I2
CPL	quercetin	Cytochrome P450 1B1	CYP1B1
CPL	quercetin	G2/mitotic-specific cyclin-B1	CCNB1
CPL	quercetin	Tissue-type plasminogen activator	PLAT
CPL	quercetin	Thrombomodulin	THBD
CPL	quercetin	Plasminogen activator inhibitor 1	SERPINE1
CPL	quercetin	Interferon gamma	IFNG
CPL	quercetin	Arachidonate 5-lipoxygenase	ALOX5
CPL	quercetin	Phosphatidylinositol-3,4,5-trisphosphate 3-phosphatase and dual-specificity protein phosphatase PTEN	PTEN
CPL	quercetin	Interleukin-1 alpha	IL1A
CPL	quercetin	Myeloperoxidase	MPO

CPL	quercetin	DNA topoisomerase 2-alpha	TOP2A
CPL	quercetin	Neutrophil cytosol factor 1	NCF1
CPL	quercetin	ATP-binding cassette sub-family G member 2	ABCA2
CPL	quercetin	Hyaluronan synthase 2	HAS2
CPL	quercetin	Nuclear factor erythroid 2-related factor 2	NFE2L2
CPL	quercetin	NAD(P)H dehydrogenase [quinone] 1	NQO1
CPL	quercetin	Poly [ADP-ribose] polymerase 1	PARP1
CPL	quercetin	Aryl hydrocarbon receptor	AHR
CPL	quercetin	26S proteasome non-ATPase regulatory subunit 3	PSMD3
CPL	quercetin	Solute carrier family 2, facilitated glucose transporter member 4	SLC2A4
CPL	quercetin	Collagen alpha-1(III) chain	COL3A1
CPL	quercetin	C-X-C motif chemokine 11	CXCL11
CPL	quercetin	C-X-C motif chemokine 2	CXCL2
CPL	quercetin	DDB1- and CUL4-associated factor 5	DCAF5
CPL	quercetin	Nuclear receptor subfamily 1 group I member 3	NR1I3
CPL	quercetin	Serine/threonine-protein kinase Chk2	CHEK2
CPL	quercetin	Insulin receptor	INSR
CPL	quercetin	Claudin-4	CLDN4
CPL	quercetin	Peroxisome proliferator-activated receptor alpha	PPARA
CPL	quercetin	Peroxisome proliferator-activated receptor delta	PPARD
CPL	quercetin	Heat shock factor protein 1	HSF1
CPL	quercetin	C-reactive protein	CRP
CPL	quercetin	C-X-C motif chemokine 10	CXCL10
CPL	quercetin	Inhibitor of nuclear factor kappa-B kinase subunit alpha	CHUK
CPL	quercetin	Osteopontin	SPP1
CPL	quercetin	Runt-related transcription factor 2	RUNX2
CPL	quercetin	Ras association domain-containing protein 1	RASSF1
CPL	quercetin	Transcription factor E2F1	E2F1
CPL	quercetin	Transcription factor E2F2	E2F2
CPL	quercetin	Prostatic acid phosphatase	ACP3
CPL	quercetin	Cathepsin D	CTSD
CPL	quercetin	Insulin-like growth factor-binding protein 3	IGFBP3
CPL	quercetin	Insulin-like growth factor II	IGF2
CPL	quercetin	CD40 ligand	CD40LG
CPL	quercetin	Interferon regulatory factor 1	IRF1
CPL	quercetin	Receptor tyrosine-protein kinase erbB-3	ERBB3
CPL	quercetin	Serum paraoxonase/arylesterase 1	PON1
CPL	quercetin	Type I iodothyronine deiodinase	DIO1
CPL	quercetin	Procollagen C-endopeptidase enhancer 1	PCOLCE
CPL	quercetin	Puromycin-sensitive aminopeptidase	NPEPPS
CPL	quercetin	Hexokinase-2	HK2
CPL	quercetin	Homeobox protein Nkx-3.1	NKX3-1
CPL	quercetin	Ras GTPase-activating protein 1	RASA1
CPL	quercetin	Glutathione S-transferase Mu 1	GSTM1
CPL	quercetin	Glutathione S-transferase Mu 2	GSTM2
PGY11	scopoletin	Prostaglandin G/H synthase 1	PTGS1

PGY11	scopoletin	Prothrombin	F2
PGY11	scopoletin	Ribosyldihydronicotinamide dehydrogenase [quinone]	NQO2
PGY11	scopoletin	Prostaglandin G/H synthase 2	PTGS2
PGY11	scopoletin	Carbonic anhydrase 1	CA1
PGY11	scopoletin	Beta-2 adrenergic receptor	ADRB2
PGY11	scopoletin	Leukotriene A-4 hydrolase	LTA4H
PGY11	scopoletin	Amine oxidase [flavin-containing] B	MAOB
PGY11	scopoletin	Glutamate receptor 2	GRIA2
PGY11	scopoletin	cAMP-dependent protein kinase catalytic subunit alpha	PRKACA
PGY11	scopoletin	Sodium-dependent noradrenaline transporter	SLC6A2
PGY12	vitamin b2	Prostaglandin G/H synthase 2	PTGS2
PGY12	vitamin b2	DNA topoisomerase 2-alpha	TOP2A
PGY13	vitamin c	Prostaglandin G/H synthase 1	PTGS1
PGY13	vitamin c	Prostaglandin G/H synthase 2	PTGS2
PGY13	vitamin c	Gamma-aminobutyric-acid receptor subunit alpha-1	GABRA1
PGY13	vitamin c	Lactase-phlorizin hydrolase	LCT
PGY13	vitamin c	Glutamate receptor 2	GRIA2

Fritillariae Thunbergii Bulbus

Abbreviation	Molecule Name	Target Name	Genesymbol
ZBM1	pelargonidin	Nitric oxide synthase, inducible	NOS2
ZBM1	pelargonidin	Prostaglandin G/H synthase 1	PTGS1
ZBM1	pelargonidin	Androgen receptor	AR
ZBM1	pelargonidin	Peroxisome proliferator-activated receptor gamma	PPARG
ZBM1	pelargonidin	Prostaglandin G/H synthase 2	PTGS2
ZBM1	pelargonidin	Carbonic anhydrase 2	CA2
ZBM1	pelargonidin	Heat shock protein HSP 90-alpha	HSP90AA1
ZBM1	pelargonidin	cAMP-dependent protein kinase catalytic subunit alpha	PRKACA
ZBM1	pelargonidin	Nuclear receptor coactivator 2	NCOA2
ZBM1	pelargonidin	Retinoic acid receptor RXR-alpha	RXRA
ZBM1	pelargonidin	Acetylcholinesterase	ACHE
ZBM1	pelargonidin	Progesterone receptor	PGR
ZBM1	pelargonidin	Mineralocorticoid receptor	NR3C2
ZBM1	pelargonidin	Glucocorticoid receptor	NR3C1
ZBM1	pelargonidin	Nuclear receptor coactivator 1	NCOA1
XQM	beta-sitosterol	Progesterone receptor	PGR
XQM	beta-sitosterol	Nuclear receptor coactivator 2	NCOA2
XQM	beta-sitosterol	Prostaglandin G/H synthase 1	PTGS1
XQM	beta-sitosterol	Prostaglandin G/H synthase 2	PTGS2
XQM	beta-sitosterol	Heat shock protein HSP 90-alpha	HSP90AA1
XQM	beta-sitosterol	Phosphatidylinositol-4,5-bisphosphate 3-kinase catalytic subunit gamma isoform	PIK3CG
XQM	beta-sitosterol	Potassium voltage-gated channel subfamily H member 2	KCNH2
XQM	beta-sitosterol	cAMP-dependent protein kinase catalytic subunit alpha	PRKACA
XQM	beta-sitosterol	D(1A) dopamine receptor	DRD1
XQM	beta-sitosterol	Muscarinic acetylcholine receptor M3	CHRM3
XQM	beta-sitosterol	Muscarinic acetylcholine receptor M1	CHRM1
XQM	beta-sitosterol	Sodium channel protein type 5 subunit alpha	SCN5A
XQM	beta-sitosterol	Gamma-aminobutyric-acid receptor subunit alpha-2	GABRA2
XQM	beta-sitosterol	Muscarinic acetylcholine receptor M4	CHRM4
XQM	beta-sitosterol	cGMP-inhibited 3',5'-cyclic phosphodiesterase A	PDE3A
XQM	beta-sitosterol	5-hydroxytryptamine 2A receptor	HTR2A
XQM	beta-sitosterol	Gamma-aminobutyric-acid receptor subunit alpha-5	GABRA5
XQM	beta-sitosterol	Alpha-1A adrenergic receptor	ADRA1A
XQM	beta-sitosterol	Gamma-aminobutyric-acid receptor subunit alpha-3	GABRA3
XQM	beta-sitosterol	Muscarinic acetylcholine receptor M2	CHRM2
XQM	beta-sitosterol	Alpha-1B adrenergic receptor	ADRA1B
XQM	beta-sitosterol	Beta-2 adrenergic receptor	ADRB2
XQM	beta-sitosterol	Neuronal acetylcholine receptor subunit alpha-2	CHRNA2
XQM	beta-sitosterol	Sodium-dependent serotonin transporter	SLC6A4
XQM	beta-sitosterol	Mu-type opioid receptor	OPRM1
XQM	beta-sitosterol	Gamma-aminobutyric-acid receptor subunit alpha-1	GABRA1
XQM	beta-sitosterol	Neuronal acetylcholine receptor subunit alpha-7	CHRNA7
XQM	beta-sitosterol	Apoptosis regulator Bcl-2	BCL2

XQM	beta-sitosterol	Apoptosis regulator BAX	BAX
XQM	beta-sitosterol	Caspase-9	CASP9
XQM	beta-sitosterol	Transcription factor AP-1	JUN
XQM	beta-sitosterol	Caspase-3	CASP3
XQM	beta-sitosterol	Caspase-8	CASP8
XQM	beta-sitosterol	Protein kinase C alpha type	PRKCA
XQM	beta-sitosterol	Transforming growth factor beta-1	TGFB1
XQM	beta-sitosterol	Serum paraoxonase/arylesterase 1	PON1
XQM	beta-sitosterol	Microtubule-associated protein 2	MAP2
ZBM2	Peimisine	Mineralocorticoid receptor	NR3C2
ZBM2	Peimisine	Glucocorticoid receptor	NR3C1
ZBM3	Zhebeiresinol	Prostaglandin G/H synthase 1	PTGS1
ZBM3	Zhebeiresinol	Sodium channel protein type 5 subunit alpha	SCN5A
ZBM3	Zhebeiresinol	Prostaglandin G/H synthase 2	PTGS2
ZBM3	Zhebeiresinol	Retinoic acid receptor RXR-alpha	RXRA
ZBM3	Zhebeiresinol	cGMP-inhibited 3',5'-cyclic phosphodiesterase A	PDE3A
ZBM3	Zhebeiresinol	Beta-2 adrenergic receptor	ADRB2
ZBM3	Zhebeiresinol	Gamma-aminobutyric-acid receptor subunit alpha-1	GABRA1
ZBM3	Zhebeiresinol	Heat shock protein HSP 90-alpha	HSP90AA1
ZBM4	6-Methoxyl-2-acetyl-3-methyl-1,4-naphthoquinone-8-O-beta-D-glucopyranoside	Estrogen receptor	ESR1
ZBM4	6-Methoxyl-2-acetyl-3-methyl-1,4-naphthoquinone-8-O-beta-D-glucopyranoside	Coagulation factor X	F10
ZBM4	6-Methoxyl-2-acetyl-3-methyl-1,4-naphthoquinone-8-O-beta-D-glucopyranoside	Prostaglandin G/H synthase 2	PTGS2
ZBM4	6-Methoxyl-2-acetyl-3-methyl-1,4-naphthoquinone-8-O-beta-D-glucopyranoside	Carbonic anhydrase 2	CA2
ZBM4	6-Methoxyl-2-acetyl-3-methyl-1,4-naphthoquinone-8-O-beta-D-glucopyranoside	DNA topoisomerase 2-alpha	TOP2A
ZBM4	6-Methoxyl-2-acetyl-3-methyl-1,4-naphthoquinone-8-O-beta-D-glucopyranoside	Heat shock protein HSP 90-alpha	HSP90AA1
ZBM4	6-Methoxyl-2-acetyl-3-methyl-1,4-naphthoquinone-8-O-beta-D-glucopyranoside	Nuclear receptor coactivator 2	NCOA2

Aurantii Fructus Immaturus

Abbreviation	Molecule Name	Target Name	Genesymbol
ZS1	Isosinensetin	Nitric oxide synthase, inducible	NOS2
ZS1	Isosinensetin	Prostaglandin G/H synthase 1	PTGS1
ZS1	Isosinensetin	Prothrombin	F2
ZS1	Isosinensetin	Potassium voltage-gated channel subfamily H member 2	KCNH2
ZS1	Isosinensetin	Estrogen receptor	ESR1
ZS1	Isosinensetin	Androgen receptor	AR
ZS1	Isosinensetin	Sodium channel protein type 5 subunit alpha	SCN5A
ZS1	Isosinensetin	ATP-binding cassette transporter sub-family C member 8	ABCC8
ZS1	Isosinensetin	Peroxisome proliferator-activated receptor gamma	PPARG
ZS1	Isosinensetin	Coagulation factor X	F10
ZS1	Isosinensetin	Prostaglandin G/H synthase 2	PTGS2
ZS1	Isosinensetin	Nitric-oxide synthase, endothelial	NOS3
ZS1	Isosinensetin	Carbonic anhydrase 2	CA2
ZS1	Isosinensetin	Coagulation factor VII	F7
ZS1	Isosinensetin	Acetylcholinesterase	ACHE
ZS1	Isosinensetin	Tyrosine-protein phosphatase non-receptor type 1	PTPN1
ZS1	Isosinensetin	Beta-2 adrenergic receptor	ADRB2
ZS1	Isosinensetin	DNA topoisomerase 2-alpha	TOP2A
ZS1	Isosinensetin	Estrogen receptor beta	ESR2
ZS1	Isosinensetin	Dipeptidyl peptidase 4	DPP4
ZS1	Isosinensetin	Heat shock protein HSP 90-alpha	HSP90AA1
ZS1	Isosinensetin	Serine/threonine-protein kinase Chk1	CHEK1
ZS1	Isosinensetin	Trypsin-1	PRSS1
ZS1	Isosinensetin	Nuclear receptor coactivator 2	NCOA2
ZS1	Isosinensetin	Nuclear receptor coactivator 1	NCOA1
ZS1	Isosinensetin	Calcium-activated potassium channel subunit alpha 1	KCNMA1
ZS2	5,7,4'-Trimethylapigenin	Nitric oxide synthase, inducible	NOS2
ZS2	5,7,4'-Trimethylapigenin	Prostaglandin G/H synthase 1	PTGS1
ZS2	5,7,4'-Trimethylapigenin	Sodium channel protein type 5 subunit alpha	SCN5A
ZS2	5,7,4'-Trimethylapigenin	Coagulation factor X	F10
ZS2	5,7,4'-Trimethylapigenin	Prostaglandin G/H synthase 2	PTGS2
ZS2	5,7,4'-Trimethylapigenin	Nitric-oxide synthase, endothelial	NOS3
ZS2	5,7,4'-Trimethylapigenin	Carbonic anhydrase 2	CA2
ZS2	5,7,4'-Trimethylapigenin	Alpha-2C adrenergic receptor	ADRA2C
ZS2	5,7,4'-Trimethylapigenin	Alpha-1B adrenergic receptor	ADRA1B
ZS2	5,7,4'-Trimethylapigenin	Beta-2 adrenergic receptor	ADRB2
ZS2	5,7,4'-Trimethylapigenin	Estrogen receptor beta	ESR2
ZS2	5,7,4'-Trimethylapigenin	Dipeptidyl peptidase 4	DPP4
ZS2	5,7,4'-Trimethylapigenin	Heat shock protein HSP 90-alpha	HSP90AA1
ZS2	5,7,4'-Trimethylapigenin	Trypsin-1	PRSS1
ZS2	5,7,4'-Trimethylapigenin	Nuclear receptor coactivator 2	NCOA2
ZS3	isosakuranetin-7-rutinoside	DNA topoisomerase 2-alpha	TOP2A
ZS4	Prangenin	Prothrombin	F2
ZS4	Prangenin	Prostaglandin G/H synthase 2	PTGS2
ZS4	Prangenin	Dipeptidyl peptidase 4	DPP4

ZS5	poncimarin	Prothrombin	F2
ZS5	poncimarin	Estrogen receptor	ESR1
ZS5	poncimarin	Androgen receptor	AR
ZS5	poncimarin	Prostaglandin G/H synthase 2	PTGS2
ZS6	isoponcimarin	Prothrombin	F2
ZS6	isoponcimarin	Prostaglandin G/H synthase 2	PTGS2
ZS6	isoponcimarin	cGMP-inhibited 3',5'-cyclic phosphodiesterase A	PDE3A
ZS6	isoponcimarin	Beta-2 adrenergic receptor	ADRB2
ZS7	6-Methoxy aurapten	Muscarinic acetylcholine receptor M3	CHRM3
ZS7	6-Methoxy aurapten	Muscarinic acetylcholine receptor M1	CHRM1
ZS7	6-Methoxy aurapten	Coagulation factor X	F10
ZS7	6-Methoxy aurapten	Prostaglandin G/H synthase 2	PTGS2
ZS7	6-Methoxy aurapten	Carbonic anhydrase 2	CA2
ZS7	6-Methoxy aurapten	Retinoic acid receptor RXR-alpha	RXRA
ZS7	6-Methoxy aurapten	cGMP-inhibited 3',5'-cyclic phosphodiesterase A	PDE3A
ZS7	6-Methoxy aurapten	Alpha-1B adrenergic receptor	ADRA1B
ZS7	6-Methoxy aurapten	Beta-2 adrenergic receptor	ADRB2
ZS7	6-Methoxy aurapten	Nuclear receptor coactivator 2	NCOA2
ZS8	citrusin B	Prothrombin	F2
ZS9	neohesperidin_qt	Prostaglandin G/H synthase 1	PTGS1
ZS9	neohesperidin_qt	Sodium channel protein type 5 subunit alpha	SCN5A
ZS9	neohesperidin_qt	Prostaglandin G/H synthase 2	PTGS2
ZS9	neohesperidin_qt	Heat shock protein HSP 90-alpha	HSP90AA1
ZS9	neohesperidin_qt	Phosphatidylinositol-4,5-bisphosphate 3-kinase catalytic subunit gamma isoform	PIK3CG
ZS9	neohesperidin_qt	cAMP-dependent protein kinase catalytic subunit alpha	PRKACA
ZS10	Sinensetin	Nitric oxide synthase, inducible	NOS2
ZS10	Sinensetin	Prothrombin	F2
ZS10	Sinensetin	Potassium voltage-gated channel subfamily H member 2	KCNH2
ZS10	Sinensetin	Androgen receptor	AR
ZS10	Sinensetin	Sodium channel protein type 5 subunit alpha	SCN5A
ZS10	Sinensetin	Coagulation factor X	F10
ZS10	Sinensetin	Prostaglandin G/H synthase 2	PTGS2
ZS10	Sinensetin	Coagulation factor VII	F7
ZS10	Sinensetin	Acetylcholinesterase	ACHE
ZS10	Sinensetin	Beta-2 adrenergic receptor	ADRB2
ZS10	Sinensetin	DNA topoisomerase 2-alpha	TOP2A
ZS10	Sinensetin	Estrogen receptor beta	ESR2
ZS10	Sinensetin	Dipeptidyl peptidase 4	DPP4
ZS10	Sinensetin	Heat shock protein HSP 90-alpha	HSP90AA1
ZS10	Sinensetin	Serine/threonine-protein kinase Chk1	CHEK1
ZS10	Sinensetin	Trypsin-1	PRSS1
ZS10	Sinensetin	Nuclear receptor coactivator 2	NCOA2
ZS10	Sinensetin	Nuclear receptor coactivator 1	NCOA1
ZS10	Sinensetin	Prostaglandin G/H synthase 1	PTGS1
ZS10	Sinensetin	Alpha-1B adrenergic receptor	ADRA1B
ZS11	Ammidin	Prothrombin	F2
ZS11	Ammidin	Muscarinic acetylcholine receptor M1	CHRM1

ZS11	Ammidin	Prostaglandin G/H synthase 2	PTGS2
ZS11	Ammidin	Gamma-aminobutyric-acid receptor subunit alpha-1	GABRA1
ZS11	Ammidin	Dipeptidyl peptidase 4	DPP4
ZS11	Ammidin	Phosphatidylinositol-4,5-bisphosphate 3-kinase catalytic subunit gamma isoform	PIK3CG
ZS11	Ammidin	cAMP-dependent protein kinase catalytic subunit alpha	PRKACA
ZS11	Ammidin	Amine oxidase [flavin-containing] B	MAOB
QS	Eriodyctiol (flavanone)	Prostaglandin G/H synthase 1	PTGS1
QS	Eriodyctiol (flavanone)	Prostaglandin G/H synthase 2	PTGS2
QS	Eriodyctiol (flavanone)	Heat shock protein HSP 90-alpha	HSP90AA1
QS	Eriodyctiol (flavanone)	cAMP-dependent protein kinase catalytic subunit alpha	PRKACA
QS	Eriodyctiol (flavanone)	Nuclear receptor coactivator 2	NCOA2
QS	Eriodyctiol (flavanone)	Phosphatidylinositol-4,5-bisphosphate 3-kinase catalytic subunit gamma isoform	PIK3CG
QS	Eriodyctiol (flavanone)	Glycogen phosphorylase, muscle form	PYGM
CS	naringenin	Prostaglandin G/H synthase 1	PTGS1
CS	naringenin	Estrogen receptor	ESR1
CS	naringenin	Prostaglandin G/H synthase 2	PTGS2
CS	naringenin	Heat shock protein HSP 90-alpha	HSP90AA1
CS	naringenin	Beta-lactamase	DPEP1
CS	naringenin	cAMP-dependent protein kinase catalytic subunit alpha	PRKACA
CS	naringenin	Phosphatidylinositol-4,5-bisphosphate 3-kinase catalytic subunit gamma isoform	PIK3CG
CS	naringenin	Transcription factor p65	RELA
CS	naringenin	RAC-alpha serine/threonine-protein kinase	AKT1
CS	naringenin	Apoptosis regulator Bcl-2	BCL2
CS	naringenin	Mitogen-activated protein kinase 3	MAPK3
CS	naringenin	Mitogen-activated protein kinase 1	MAPK1
CS	naringenin	Caspase-3	CASP3
CS	naringenin	Fatty acid synthase	FASN
CS	naringenin	Low-density lipoprotein receptor	LDLR
CS	naringenin	Bcl2 antagonist of cell death	BAD
CS	naringenin	Superoxide dismutase [Cu-Zn]	SOD1
CS	naringenin	Peroxisome proliferator-activated receptor gamma	PPARG
CS	naringenin	Microsomal triglyceride transfer protein large subunit	MTTP
CS	naringenin	Apolipoprotein B-100	APOB
CS	naringenin	Phospholipase B1, membrane-associated	PLB1
CS	naringenin	3-hydroxy-3-methylglutaryl-coenzyme A reductase	HMGCR
CS	naringenin	Cytochrome P450 19A1	CYP19A1
CS	naringenin	UDP-glucuronosyltransferase 1-1	UGT1A1
CS	naringenin	Peroxisome proliferator-activated receptor alpha	PPARA

CS	naringenin	Sterol regulatory element-binding protein 1	SREBF1
CS	naringenin	Glutathione reductase, mitochondrial	GSR
CS	naringenin	Multidrug resistance-associated protein 1	ABCC1
CS	naringenin	Adiponectin	ADIPOQ
CS	naringenin	Sterol O-acyltransferase 2	SOAT2
CS	naringenin	Aldo-keto reductase family 1 member C1	AKR1C1
CS	naringenin	Aspartate aminotransferase, cytoplasmic	GOT1
CS	naringenin	4-aminobutyrate aminotransferase, mitochondrial	ABAT
CS	naringenin	Liver carboxylesterase 1	CES1
CS	naringenin	Sterol O-acyltransferase 1	SOAT1
ZS12	5,7-dihydroxy-2-(3-hydroxy-4-methoxyphenyl)chroman-4-one	Prostaglandin G/H synthase 1	PTGS1
ZS12	5,7-dihydroxy-2-(3-hydroxy-4-methoxyphenyl)chroman-4-one	Prostaglandin G/H synthase 2	PTGS2
ZS12	5,7-dihydroxy-2-(3-hydroxy-4-methoxyphenyl)chroman-4-one	Heat shock protein HSP 90-alpha	HSP90AA1
ZS12	5,7-dihydroxy-2-(3-hydroxy-4-methoxyphenyl)chroman-4-one	cAMP-dependent protein kinase catalytic subunit alpha	PRKACA
ZS12	5,7-dihydroxy-2-(3-hydroxy-4-methoxyphenyl)chroman-4-one	Sodium channel protein type 5 subunit alpha	SCN5A
ZS12	5,7-dihydroxy-2-(3-hydroxy-4-methoxyphenyl)chroman-4-one	Phosphatidylinositol-4,5-bisphosphate 3-kinase catalytic subunit gamma isoform	PIK3CG
ZS12	5,7-dihydroxy-2-(3-hydroxy-4-methoxyphenyl)chroman-4-one	Nuclear receptor coactivator 2	NCOA2
ZS12	5,7-dihydroxy-2-(3-hydroxy-4-methoxyphenyl)chroman-4-one	Nuclear receptor coactivator 1	NCOA1
ZS12	5,7-dihydroxy-2-(3-hydroxy-4-methoxyphenyl)chroman-4-one	DNA topoisomerase 2-alpha	TOP2A
ZS13	nobiletin	Nitric oxide synthase, inducible	NOS2
ZS13	nobiletin	Prostaglandin G/H synthase 1	PTGS1
ZS13	nobiletin	Prothrombin	F2
ZS13	nobiletin	Potassium voltage-gated channel subfamily H member 2	KCNH2
ZS13	nobiletin	Estrogen receptor	ESR1
ZS13	nobiletin	Androgen receptor	AR
ZS13	nobiletin	Peroxisome proliferator-activated receptor gamma	PPARG
ZS13	nobiletin	Coagulation factor X	F10
ZS13	nobiletin	Prostaglandin G/H synthase 2	PTGS2
ZS13	nobiletin	Coagulation factor VII	F7
ZS13	nobiletin	Tyrosine-protein phosphatase non-receptor type 1	PTPN1
ZS13	nobiletin	DNA topoisomerase 2-alpha	TOP2A
ZS13	nobiletin	Estrogen receptor beta	ESR2
ZS13	nobiletin	Dipeptidyl peptidase 4	DPP4
ZS13	nobiletin	Heat shock protein HSP 90-alpha	HSP90AA1
ZS13	nobiletin	Serine/threonine-protein kinase Chk1	CHEK1
ZS13	nobiletin	Trypsin-1	PRSS1
ZS13	nobiletin	Nuclear receptor coactivator 2	NCOA2

ZS13	nobiletin	Calcium-activated potassium channel subunit alpha 1	KCNMA1
ZS13	nobiletin	Glycogen synthase kinase-3 beta	GSK3B
ZS13	nobiletin	Sodium channel protein type 5 subunit alpha	SCN5A
ZS13	nobiletin	Apoptosis regulator Bcl-2	BCL2
ZS13	nobiletin	Apoptosis regulator BAX	BAX
ZS13	nobiletin	Caspase-9	CASP9
ZS13	nobiletin	Matrix metalloproteinase-9	MMP9
ZS13	nobiletin	Transcription factor AP-1	JUN
ZS13	nobiletin	Cellular tumor antigen p53	TP53
ZS13	nobiletin	Mitogen-activated protein kinase 8	MAPK8
ZS13	nobiletin	Metalloproteinase inhibitor 1	TIMP1
ZS13	nobiletin	Peroxisome proliferator-activated receptor gamma	PPARG
ZS13	nobiletin	Cyclic AMP-responsive element-binding protein 1	CREB1
ZS13	nobiletin	Cytosolic phospholipase A2	PLA2G4A
ZS13	nobiletin	Scavenger receptor cysteine-rich type 1 protein M130	CD163
ZS13	nobiletin	Ephrin type-B receptor 2	EPHB2
ZS14	didymin	Prostaglandin G/H synthase 1	PTGS1
ZS14	didymin	Estrogen receptor	ESR1
ZS14	didymin	Sodium channel protein type 5 subunit alpha	SCN5A
ZS14	didymin	Prostaglandin G/H synthase 2	PTGS2
ZS14	didymin	Retinoic acid receptor RXR-alpha	RXRA
ZS14	didymin	Beta-2 adrenergic receptor	ADRB2
ZS14	didymin	Heat shock protein HSP 90-alpha	HSP90AA1
ZS14	didymin	Phosphatidylinositol-4,5-bisphosphate 3-kinase catalytic subunit gamma isoform	PIK3CG
ZS14	didymin	Beta-lactamase	DPEP1
ZS14	didymin	cAMP-dependent protein kinase catalytic subunit alpha	PRKACA
ZS14	didymin	Nuclear receptor coactivator 2	NCOA2
ZS14	didymin	Nuclear receptor coactivator 1	NCOA1
ZS15	luteolin	Prostaglandin G/H synthase 1	PTGS1
ZS15	luteolin	Androgen receptor	AR
ZS15	luteolin	Prostaglandin G/H synthase 2	PTGS2
ZS15	luteolin	Heat shock protein HSP 90-alpha	HSP90AA1
ZS15	luteolin	Trypsin-1	PRSS1
ZS15	luteolin	Nuclear receptor coactivator 2	NCOA2
ZS15	luteolin	cAMP-dependent protein kinase catalytic subunit alpha	PRKACA
ZS15	luteolin	Dipeptidyl peptidase 4	DPP4
ZS15	luteolin	Phosphatidylinositol-4,5-bisphosphate 3-kinase catalytic subunit gamma isoform	PIK3CG
ZS15	luteolin	Transcription factor p65	RELA
ZS15	luteolin	Epidermal growth factor receptor	EGFR
ZS15	luteolin	RAC-alpha serine/threonine-protein kinase	AKT1
ZS15	luteolin	G1/S-specific cyclin-D1	CCND1
ZS15	luteolin	Bcl-2-like protein 1	BCL2L1

ZS15	luteolin	Cyclin-dependent kinase inhibitor 1	CDKN1A
ZS15	luteolin	Caspase-9	CASP9
ZS15	luteolin	72 kDa type IV collagenase	MMP2
ZS15	luteolin	Matrix metalloproteinase-9	MMP9
ZS15	luteolin	Mitogen-activated protein kinase 1	MAPK1
ZS15	luteolin	Interleukin-10	IL10
ZS15	luteolin	Retinoblastoma-associated protein	RB1
ZS15	luteolin	Cell division protein kinase 4	CDK4
ZS15	luteolin	Tumor necrosis factor	TNF
ZS15	luteolin	Transcription factor AP-1	JUN
ZS15	luteolin	Interleukin-6	IL6
ZS15	luteolin	Caspase-3	CASP3
ZS15	luteolin	Cellular tumor antigen p53	TP53
ZS15	luteolin	NF-kappa-B inhibitor alpha	NFKBIA
ZS15	luteolin	Xanthine dehydrogenase/oxidase	XDH
ZS15	luteolin	DNA topoisomerase 1	TOP1
ZS15	luteolin	E3 ubiquitin-protein ligase Mdm2	MDM2
ZS15	luteolin	Amyloid beta A4 protein	APP
ZS15	luteolin	Interstitial collagenase	MMP1
ZS15	luteolin	Proliferating cell nuclear antigen	PCNA
ZS15	luteolin	Receptor tyrosine-protein kinase erbB-2	ERBB2
ZS15	luteolin	Peroxisome proliferator-activated receptor gamma	PPARG
ZS15	luteolin	Heme oxygenase 1	HMOX1
ZS15	luteolin	Caspase-7	CASP7
ZS15	luteolin	Intercellular adhesion molecule 1	ICAM1
ZS15	luteolin	Induced myeloid leukemia cell differentiation protein Mcl-1	MCL1
ZS15	luteolin	Baculoviral IAP repeat-containing protein 5	BIRC5
ZS15	luteolin	Interleukin-2	IL2
ZS15	luteolin	G2/mitotic-specific cyclin-B1	CCNB1
ZS15	luteolin	Tyrosinase	TYR
ZS15	luteolin	Interferon gamma	IFNG
ZS15	luteolin	Interleukin-4	IL4
ZS15	luteolin	DNA topoisomerase 2-alpha	TOP2A
ZS15	luteolin	Baculoviral IAP repeat-containing protein 4	XIAP
ZS15	luteolin	Solute carrier family 2, facilitated glucose transporter member 4	SLC2A4
ZS15	luteolin	Insulin receptor	INSR
ZS15	luteolin	CD40 ligand	CD40LG
ZS15	luteolin	Prostaglandin E synthase	PTGES
ZS15	luteolin	Kinetochore protein Nuf2	NUF2
ZS15	luteolin	Adenylate cyclase type 2	ADCY2
ZS15	luteolin	Hepatocyte growth factor receptor	MET
ZS16	Tetramethoxyluteolin	Nitric oxide synthase, inducible	NOS2
ZS16	Tetramethoxyluteolin	Prostaglandin G/H synthase 1	PTGS1
ZS16	Tetramethoxyluteolin	Prothrombin	F2
ZS16	Tetramethoxyluteolin	Androgen receptor	AR
ZS16	Tetramethoxyluteolin	Sodium channel protein type 5 subunit alpha	SCN5A
ZS16	Tetramethoxyluteolin	Peroxisome proliferator-activated receptor gamma	PPARG

ZS16	Tetramethoxyluteolin	Coagulation factor X	F10
ZS16	Tetramethoxyluteolin	Prostaglandin G/H synthase 2	PTGS2
ZS16	Tetramethoxyluteolin	Nitric-oxide synthase, endothelial	NOS3
ZS16	Tetramethoxyluteolin	Coagulation factor VII	F7
ZS16	Tetramethoxyluteolin	Retinoic acid receptor RXR-alpha	RXRA
ZS16	Tetramethoxyluteolin	Alpha-1B adrenergic receptor	ADRA1B
ZS16	Tetramethoxyluteolin	Beta-2 adrenergic receptor	ADRB2
ZS16	Tetramethoxyluteolin	Estrogen receptor beta	ESR2
ZS16	Tetramethoxyluteolin	Dipeptidyl peptidase 4	DPP4
ZS16	Tetramethoxyluteolin	Beta-secretase 1	BACE1
ZS16	Tetramethoxyluteolin	Mitogen-activated protein kinase 14	MAPK14
ZS16	Tetramethoxyluteolin	Glycogen synthase kinase-3 beta	GSK3B
ZS16	Tetramethoxyluteolin	Heat shock protein HSP 90-alpha	HSP90AA1
ZS16	Tetramethoxyluteolin	Serine/threonine-protein kinase Chk1	CHEK1
ZS16	Tetramethoxyluteolin	cAMP-dependent protein kinase catalytic subunit alpha	PRKACA
ZS16	Tetramethoxyluteolin	Trypsin-1	PRSS1
ZS16	Tetramethoxyluteolin	Nuclear receptor coactivator 2	NCOA2
ZS16	Tetramethoxyluteolin	Nuclear receptor coactivator 1	NCOA1
ZS16	Tetramethoxyluteolin	Potassium voltage-gated channel subfamily H member 2	KCNH2
ZS16	Tetramethoxyluteolin	Acetylcholinesterase	ACHE
ZS16	Tetramethoxyluteolin	Alpha-1D adrenergic receptor	ADRA1D
ZS16	Tetramethoxyluteolin	DNA topoisomerase 2-alpha	TOP2A
ZS16	Tetramethoxyluteolin	Nuclear receptor subfamily 1 group I member 2	NR1I2
ZS16	Tetramethoxyluteolin	Calcium-activated potassium channel subunit alpha 1	KCNMA1
ZS17	4-[(2S,3R)-5-[(E)-3-hydroxyprop-1-enyl]-7-methoxy-3-methylol-2,3-dihydrobenzofuran-2-yl]-2-methoxy-phenol	Prothrombin	F2
ZS17	4-[(2S,3R)-5-[(E)-3-hydroxyprop-1-enyl]-7-methoxy-3-methylol-2,3-dihydrobenzofuran-2-yl]-2-methoxy-phenol	Potassium voltage-gated channel subfamily H member 2	KCNH2
ZS17	4-[(2S,3R)-5-[(E)-3-hydroxyprop-1-enyl]-7-methoxy-3-methylol-2,3-dihydrobenzofuran-2-yl]-2-methoxy-phenol	Estrogen receptor	ESR1
ZS17	4-[(2S,3R)-5-[(E)-3-hydroxyprop-1-enyl]-7-methoxy-3-methylol-2,3-dihydrobenzofuran-2-yl]-2-methoxy-phenol	Coagulation factor X	F10
ZS17	4-[(2S,3R)-5-[(E)-3-hydroxyprop-1-enyl]-7-methoxy-3-methylol-2,3-dihydrobenzofuran-2-yl]-2-methoxy-phenol	Prostaglandin G/H synthase 2	PTGS2

ZS17	4-[(2S,3R)-5-[(E)-3-hydroxyprop-1-enyl]-7-methoxy-3-methylol-2,3-dihydrobenzofuran-2-yl]-2-methoxy-phenol	Dipeptidyl peptidase 4	DPP4
ZS17	4-[(2S,3R)-5-[(E)-3-hydroxyprop-1-enyl]-7-methoxy-3-methylol-2,3-dihydrobenzofuran-2-yl]-2-methoxy-phenol	Heat shock protein HSP 90-alpha	HSP90AA1
ZS17	4-[(2S,3R)-5-[(E)-3-hydroxyprop-1-enyl]-7-methoxy-3-methylol-2,3-dihydrobenzofuran-2-yl]-2-methoxy-phenol	Trypsin-1	PRSS1
ZS17	4-[(2S,3R)-5-[(E)-3-hydroxyprop-1-enyl]-7-methoxy-3-methylol-2,3-dihydrobenzofuran-2-yl]-2-methoxy-phenol	Proto-oncogene serine/threonine-protein kinase Pim-1	PIM1
ZS17	4-[(2S,3R)-5-[(E)-3-hydroxyprop-1-enyl]-7-methoxy-3-methylol-2,3-dihydrobenzofuran-2-yl]-2-methoxy-phenol	Acetylcholinesterase	ACHE
