# Supplementary material for: Herbal Medicine Hewei Jiangni Decoction Is Noninferior to Oral Omeprazole for the Treatment of Nonerosive Gastroesophageal Reflux Disease: A Randomized, Double-Blind, and Double-Dummy Controlled Trail
Source: Evid Based Complement Alternat Med. 2022 Sep 22;2022:9647003. doi: 10.1155/2022/9647003 (PMC9522514; doi:10.1155/2022/9647003)
Supplement: Supplementary Materials — (1) The active compounds and potential targets of HWJND. Supplementary materials. (2) The information of GERD-related targets. (3) The information on GO and KEGG pathway enrichment analysis. (4) The information of molecular docking. [file 9647003.f1.zip › 9647003.f1/Supplementary materials 2 the information of NERD-related targets.pdf]

GeneCards	OMIM	DisGeNET	TTD
IL6	AAOPD	IL1B	mGluR5
TP53	ABC1	CXCL8	HTR4
TNF	ABCR	OCLN	Proton pump
TGFB1	AD4	CA1	GABBR
NOD2	ADH3	TRPV1	H2R
IL10	ADMD	TAC1	HTR3A
CHAT	AILJK	NPDC1	MLNR
HLA-DRB1	AIS1	ATP4A	ATP4A
IL1B	AIS5	ATP12A	CCKBR
MPO	AITD3	SH2D1A	
CXCL8	APG16L	GSTK1	
CRP	ARC41	EIF6	
GER	ASD2	IL1RN	
GATA3	BCATE2	APCS	
EGFR	BCPM	CYP2C19	
MECP2	C1orf106	SLCO6A1	
CCL2	CCDD	CALCA	
MMP1	CD10		
MUC1	CELIAC1		
IL4	CGD1		
IL1RN	CHDS7		
GMPPA	CLP46		
FAS	CMRD		
TERT	CMT1B		
ASIC4-AS1	CMT1G		
ALDH18A1	CMT2A		
MIR146A	CMT2B		
JUP	CMT2DD		
IL23R	CMT2E		
PTGS2	CMT4A		
TNFRSF11B	CMT4K		
CCND1	CMTD1F		
FOXP3	CSIF		
IL2	D10S105E		
IRF5	DDX16		
ADIPOQ	DJC6		
EGF	DZIP2		
ELANE	E1B		
IL5	EIF4G		
CYP2C19	FARSLB		
IL1A	FRPHE		
IL13	FSGS9		
CCL11	GARS		
IL18	GBD4		
IL17A	GDE		
HP	GLE1L		
MSR1	GPR13		
ABCC8	GS		
COL5A1	GSD15		
GAST	GSD4		
MMP3	HARS		
GHRL	HD		
GNB3	HDFNRH		

LEP	HEDJ
ASCC1	HLA-H
SALL1	HLN2
HRH2	HMSNL
NFKB1	HOX4D
HTR2A	HSCR3
NEXMIF	HSP27
CXCR1	IBD14
CCK	IBD17
MDM2	IBMPFD1
S100A8	IBMPFD2
RPS6KA3	IDDM12
CTHRC1	IFI41
NPY	IFNB2
PTGS1	IGSF4B
CXCL12	IL1F7
MLN	JP1
GCG	KIAA0214
ATP4A	KIAA0274
CALCA	KIAA0517
LTA	KIAA0642
CCR3	KIAA0720
PTH	KIAA1441
MKI67	KIAA1630
CDX2	KIAA1985
CCL26	KROX20
VIP	LMN1
BAX	LQT9
NTS	LRG47
ATP12A	MDM
RETN	MEN2A
SLC22A4	MTDPS6
PYY	MYMY
SLC22A5	NACP
IL4R	NBPHOX
CALB2	NIID
ZNF469	NISBD2
DKK1	OMI
BCL2	OPG
DSG1	P62
COL11A2	PAHX
F2RL1	PARK2
SLC9A1	PARK22
TAC1	PARK5
RELA	PARK6
HGF	PARK7
DSG2	PCLD2
CBS	PDE11A1
TRPV1	PDNP1
COMP	PGAMM
GABBR1	PGY1
TGFA	PGY3
PGC	PKHD1
GPX7	PON

ACTL6A	PP2CM
BIRC5	PPH1
CXCR2	PPNAD3
FGF7	PROML1
FAM120AOS	PUM
PGA3	RHNA
CD59	RP47
PADI4	RTD
MUC3A	SAP1
HLA-DQB1	SAX3
IFNG	SCA17
AKT1	SEC61
TNFRSF1A	SIASD
MMP9	SLEB2
CST3	SPA2
CTSD	SRK
MTOR	TACE
FASLG	TAL
NOS2	TAN1
GSTP1	TIL3
SST	TNX
GSTM1	URBWD
TIMP1	URK
GAPDH	VAMAS3
GSTT1	VAMAS4
SLPI	VAMAS5
GZMB	VUR3
AKR1C2	WPWS
AKR1B10	YARS
	ZNF406
	AAKAD
	AC133
	ADMIO2
	ADSD
	ADTKD2
	ADTKD4
	ADTKD5
	AOS5
	APBD
	ARPKD
	BDPLT10
	BSF2
	CALLA
	CELIAC3
	CHN1
	CMH6
	CMT1F
	CMT2A1
	CMT2A2A
	CMT2EE
	CMT2F
	CMT2K
	CMT2R
	CMT2Y

CMT4D
CMTDIC
CMTDID
COLEC5
DBP2
DJ9
DSMA4
EMD2
ESA
ETM6
FIL1Z
FPC
GDA
GSD10
GVHDS
HDL4
HFE1
HHD
HOMGSMR2
HPALP1
HSCR1
IBD10
IBD29
IFI1
IFI75
IMD71
IT15
KIAA0473
KIAA1568
KTELC1
LCCS
MC4DN1
MDR1
MDR3
MNMN
MPDT
NISBD1
NPPS
OCIF
PARK1
PARK11
PARK13
PARK18
PDB3
PDB6
PDE11A2
PDJ
PKD5
PMX2B
POVD1
PSAPD
PSN
PTMP
PYL

QPD
RIFLE
RILDBC1
SAC3
SARA2
SLD
SLEB1
SLEB10
SMAD1
SPG79
STGD1
STGD2
STM2
STSL1
TDH3
TNXB1
TSLL1
USH3B
V28
VAMAS2
VMCKD
VSD1
ABBP2
ALPS5
ALS11
ANDD
AOVD1
BDPLT5
BTPS2
CMD1V
CMT2D
CMT2P
CMT2Q
CMT2W
CMTDIG
CMTRIA
CMTRIC
DSS
FFM
FPLD2
FTDALS3
FTDALS6
HMN2B
HMSN6A
HSF
IBD13
IBD19
ICP3
IL1H4
ILD2
IMD48
LCCS1
LOMARS
M6S1

MELIOS
MSUDMV
MVCD5
MVCD7
NBLST1
NBLST2
NDGOA
NECL1
NEP
OPDM3
PARK19
PARK24
PARK4
PDB5
PDE11A3
PKD4
PRP8
PRSS25
RMD2
RP41
RUMI
STGD3
TACHD
TGD
TNXBS
TYRRS
VODI
C3orf9
CAAHD
CCHS
CHN2
CLCs
CMD1A
CMT2A2B
CMT2T
CORD12
DESMD
HDLCQTL13
HGF
HMN5A
IL1RP1
ISQMR
MGCA8
NADGP
NMOAS
PCA1
PKD6
PPNAD2
RP19
SYNCAM3
TFQTL2
TNXB2
TOF
YTS

YVS
YRS
SMAJI
SCA43
SCA34
IBD31
HGPS
EDSCLL1
DMRV
DDD4
CORD3
CMT2FF
CD133
BTOP
ARHR2
VUR8
MCDR2
LGMDR21
IMNEPD2
COLED
ARMD2
STGD4
ABCA1
ABCA4
ABCB1
ABCB4
ABCG8
AD10
AD11
AD12
AD13
AD14
AD15
AD17
AD7
AD7CNTP
ADAM17
ADH1C
AGL
AIS2
AIS3
AIS4
AITD1
AITD2
AITD4
APC
ARPC1B
ATG16L1
ATP1A1
ATP2C1
BCKDHB
BMPR2
CADM3
CAV3

CD36
CELIAC10
CELIAC11
CELIAC12
CELIAC2
CELIAC6
CELIAC7
CELIAC8
CELIAC9
CHCHD2
CHDS2
CHDS5
CHDS8
CHDS9
CMT2H
COPA
COPD
CRB2
CTLA4
CX3CR1
DBT
DHTKD1
DHX16
DJ1
DNAJB11
DNAJC6
DZIP1L
ECE1
ECM1
EGFR
EGR2
EIF4G1
ELOVL4
ENPP1
FARSB
FCYT
FIG4
FOXD3
GARS1
GATA4
GBA
GBD2
GBD3
GBE1
GDAP1
GDNF
GIGYF2
GLE1
GNB4
GYG1
HARS1
HDL3
HEXB
HFE

HLA-DPB1
HLA-DQA1
HLA-DQB1
HNRPA2B1
HOXD10
HSCR5
HSCR6
HSCR9
HSPB1
HTRA2
HTT
IBD11
IBD12
IBD15
IBD16
IBD18
IBD23
IBD3
IBD5
IBD7
IBD9
IL10
IL23R
IL37
IL6
INAVA
IRF5
IRGM
IRS1
JPH1
KIF1B
LMNA
LPA
LRSAM1
MFN2
MME
MPV17
MPZ
MTHFR
MUC1
MYMY1
MYMY3
NBLST6
NCF1
NCF2
NDRG1
NEFL
NOS3
NOTCH1
NOTCH2NLC
PAOD1
PARK10
PARK16
PARK21

PARK3
PDB4
PDCD1
PDE11A
PDE8B
PGAM2
PHOX2B
PHYH
PINK1
PKD2
PLAU
PLEKHG5
PMP2
POGLUT1
PON1
PON2
PPM1K
PRKAG2
PRKN
PROM1
PSAP
PSEN2
RAB7
REN
RET
RHCE
RHD
RMD1
ROBO2
SAG
SAR1B
SEC61A1
SEC63
SFRP4
SFTPA2
SH3TC2
SLC17A5
SLC25A16
SLURP1
SNCA
SOX17
SP110
SQSTM1
SURF1
TBP
TG
TLR5
TNFRSF11B
TNNI3K
TNXB
TRIM2
UCHL1
VAMAS6
VCP

VUR
VUR4
YARS1
ZAP70
ZFAT1
ZNF687
