# Supplementary material for: Herbal Medicine Hewei Jiangni Decoction Is Noninferior to Oral Omeprazole for the Treatment of Nonerosive Gastroesophageal Reflux Disease: A Randomized, Double-Blind, and Double-Dummy Controlled Trail
Source: Evid Based Complement Alternat Med. 2022 Sep 22;2022:9647003. doi: 10.1155/2022/9647003 (PMC9522514; doi:10.1155/2022/9647003)
Supplement: Supplementary Materials — (1) The active compounds and potential targets of HWJND. Supplementary materials. (2) The information of GERD-related targets. (3) The information on GO and KEGG pathway enrichment analysis. (4) The information of molecular docking. [file 9647003.f1.zip › 9647003.f1/Supplementary materials 3 the information on GO and KEGG pathway enrichment analysis.pdf]

GO-BP

GO	Description	LogP	Enrichment	#GeneInGO	%InGO
GO:0032496	response to lipopoly:	-13.18135982	63.33542977	8	66.66666667
GO:0002237	response to molecule	-12.95785717	59.41199607	8	66.66666667
GO:0071216	cellular response to	-11.86649513	73.73675035	7	58.33333333
GO:0019221	cytokine-mediated si;	-10.68420685	50.06557765	7	58.33333333
GO:0009617	response to bacteriu	-10.31271604	27.74196511	8	66.66666667
GO:0071222	cellular response to	-10.1561797	75.5275	6	50
GO:0071219	cellular response to	-10.00335923	71.25235849	6	50
GO:0034097	response to cytokine	-9.982108108	25.20734251	8	66.66666667
GO:0050900	leukocyte migration	-9.767239987	65.10991379	6	50
GO:0071396	cellular response to	-9.624941848	35.31680027	7	58.33333333
GO:0006954	inflammatory respons	-9.594767836	34.96643519	7	58.33333333
GO:0071345	cellular response to	-8.59065866	25.06839734	7	58.33333333
GO:0008285	negative regulation (	-8.262984711	22.47842262	7	58.33333333
GO:0002685	regulation of leukoc	-7.847643809	57.742737	5	41.66666667
GO:1903039	positive regulation (	-7.594147879	51.3792517	5	41.66666667
GO:0022409	positive regulation (	-7.221560895	43.25744559	5	41.66666667
GO:1903037	regulation of leukoc	-6.860389534	36.59278101	5	41.66666667
GO:0097529	myeloid leukocyte mi;	-6.842794928	79.29396325	4	33.33333333
GO:0030155	regulation of cell a(	-6.695112462	19.87565789	6	50
GO:0030595	leukocyte chemotaxis	-6.611606539	69.45057471	4	33.33333333
GO:0002696	positive regulation (	-6.425958323	29.90003959	5	41.66666667
GO:0050867	positive regulation (	-6.350849359	28.8713685	5	41.66666667
GO:0045785	positive regulation (	-6.302229824	28.2240284	5	41.66666667
GO:0022407	regulation of cell-c(	-6.254712306	27.60508041	5	41.66666667
GO:0060326	cell chemotaxis	-5.993463677	48.6489533	4	33.33333333
GO:0050870	positive regulation (	-5.86469068	45.15844544	4	33.33333333
GO:0002694	regulation of leukoc	-5.568339206	20.01258612	5	41.66666667
GO:0030593	neutrophil chemotaxi:	-5.429196395	95.60443038	3	25
GO:0006959	humoral immune respo	-5.411788004	34.72528736	4	33.33333333
GO:0050865	regulation of cell a(	-5.399754209	18.48445913	5	41.66666667
GO:0071621	granulocyte chemotax:	-5.348723291	89.91369048	3	25
GO:1990266	neutrophil migration	-5.272988327	84.86235955	3	25
GO:1904035	regulation of epithe:	-5.201467392	80.34840426	3	25
GO:0097530	granulocyte migratio	-5.160390921	77.86340206	3	25
GO:0050863	regulation of T cell	-5.128712333	29.4454191	4	33.33333333
GO:0071347	cellular response to	-5.094721756	74.04656863	3	25
GO:0051251	positive regulation (	-4.989407924	27.14375562	4	33.33333333
GO:0002688	regulation of leukoc	-4.84009456	60.90927419	3	25
GO:0070555	response to interleu	-4.758770154	57.21780303	3	25
GO:0006935	chemotaxis	-4.460941624	19.90184453	4	33.33333333
GO:0042330	taxis	-4.447593979	19.74575163	4	33.33333333
GO:0051249	regulation of lympho(	-4.376060034	18.92919799	4	33.33333333
GO:0050920	regulation of chemot:	-4.068867499	33.56777778	3	25
GO:0043270	positive regulation (	-3.820573729	27.66575092	3	25
GO:0001934	positive regulation (	-12.30142618	31.38261773	9	75
GO:0045596	negative regulation (	-8.668701123	25.72712895	7	58.33333333
GO:0009410	response to xenobiot:	-6.53047203	31.39131338	5	41.66666667
GO:0030856	regulation of epithe:	-6.473192071	64.14225053	4	33.33333333
GO:0010638	positive regulation (	-4.529354109	20.72085048	4	33.33333333
GO:0097190	apoptotic signaling	-3.844260129	28.18190299	3	25
GO:0062197	cellular response to	-11.62044952	68.04279279	7	58.33333333

GO:0009411	response to UV	-10.98454768	103.4623288	6	50
GO:0006979	response to oxidativ	-10.57384702	48.28242009	7	58.33333333
GO:0009314	response to radiatio	-10.068884	40.88882444	7	58.33333333
GO:0034599	cellular response to	-9.930221061	69.2912844	6	50
GO:0010035	response to inorgani	-9.541213831	34.35298895	7	58.33333333
GO:0034614	cellular response to	-9.20803788	107.5890313	5	41.66666667
GO:0009416	response to light st	-9.061541977	49.68914474	6	50
GO:0000302	response to reactive	-8.376549175	73.61354776	5	41.66666667
GO:0010038	response to metal io	-6.816983951	35.86301045	5	41.66666667
GO:0071276	cellular response to	-6.4327577	204.1283784	3	25
GO:0090199	regulation of releas	-6.14278082	164.1902174	3	25
GO:0071248	cellular response to	-6.141900306	53.00175439	4	33.33333333
GO:0071241	cellular response to	-5.888103253	45.77424242	4	33.33333333
GO:0046686	response to cadmium	-5.813311401	128.0127119	3	25
GO:0001503	ossification	-5.528380592	37.1599016	4	33.33333333
GO:2001243	negative regulation	-5.094721756	74.04656863	3	25
GO:0010821	regulation of mitoch	-4.654784481	52.81643357	3	25
GO:0010634	positive regulation	-4.400518216	43.4066092	3	25
GO:0007169	transmembrane recept	-3.324264122	18.74131514	3	25
GO:0150077	regulation of neuroi	-11.55108361	307.0223577	5	41.66666667
GO:1903798	regulation of produc	-9.745600055	402.8133333	4	33.33333333
GO:0070920	regulation of produc	-9.673141449	387.3205128	4	33.33333333
GO:0002675	positive regulation	-9.603597583	372.9753086	4	33.33333333
GO:0010573	vascular endothelial	-8.776198424	1078.964286	3	25
GO:0002673	regulation of acute	-8.598406674	214.2624113	4	33.33333333
GO:0060964	regulation of gene s	-8.523818746	205.5170068	4	33.33333333
GO:0060147	regulation of posttr	-8.41764846	193.6602564	4	33.33333333
GO:0060966	regulation of gene s	-8.383660778	190.0062893	4	33.33333333
GO:1903800	positive regulation	-7.978329092	629.3958333	3	25
GO:1903799	negative regulation	-7.663031573	503.5166667	3	25
GO:0043065	positive regulation	-7.62566155	28.55482042	6	50
GO:0043068	positive regulation	-7.558326323	27.81860037	6	50
GO:0060252	positive regulation	-7.488728193	444.2794118	3	25
GO:0150078	positive regulation	-7.409644017	419.5972222	3	25
GO:0010942	positive regulation	-7.284261046	25.00910596	6	50
GO:0060965	negative regulation	-7.134201747	343.3068182	3	25
GO:0060149	negative regulation	-7.015706111	314.6979167	3	25
GO:0060967	negative regulation	-7.015706111	314.6979167	3	25
GO:0001816	cytokine production	-6.907137429	290.4903846	3	25
GO:0010575	positive regulation	-6.759633477	260.4396552	3	25
GO:0097421	liver regeneration	-6.759633477	260.4396552	3	25
GO:0060251	regulation of glial	-6.71397307	251.7583333	3	25
GO:2000637	positive regulation	-6.71397307	251.7583333	3	25
GO:0051384	response to glucocor	-6.685254483	72.44844125	4	33.33333333
GO:0050768	negative regulation	-6.672757613	71.93095238	4	33.33333333
GO:0060148	positive regulation	-6.669866492	243.6370968	3	25
GO:0050729	positive regulation	-6.611606539	69.45057471	4	33.33333333
GO:0051961	negative regulation	-6.611606539	69.45057471	4	33.33333333
GO:0050727	regulation of inflam	-6.568314228	31.94902707	5	41.66666667
GO:0051052	regulation of DNA me	-6.514459971	31.15820957	5	41.66666667
GO:0031960	response to corticos	-6.473192071	64.14225053	4	33.33333333
GO:0051960	regulation of nervou	-6.336144798	28.67406986	5	41.66666667
GO:0032103	positive regulation	-6.321542598	28.47944947	5	41.66666667
GO:0010721	negative regulation	-6.226060024	55.63720074	4	33.33333333

GO:1905952	regulation of lipid l	-6.206995241	55.0291439	4	33.33333333
GO:0008283	cell population prol:	-6.105143102	25.74216087	5	41.66666667
GO:0060284	regulation of cell de	-6.057573836	25.17583333	5	41.66666667
GO:0010574	regulation of vascula	-5.813311401	128.0127119	3	25
GO:0032757	positive regulation (	-5.747884841	121.8185484	3	25
GO:1905953	negative regulation (	-5.726792662	119.8849206	3	25
GO:0014015	positive regulation (	-5.706039645	118.0117188	3	25
GO:0031100	animal organ regenera	-5.6856151	116.1961538	3	25
GO:0050730	regulation of peptidy	-5.593142641	38.58365262	4	33.33333333
GO:0032722	positive regulation (	-5.588078567	107.8964286	3	25
GO:0051091	positive regulation (	-5.573451901	38.14520202	4	33.33333333
GO:0001775	cell activation	-5.544826132	19.7923218	5	41.66666667
GO:0048545	response to steroid l	-5.522038967	37.02328431	4	33.33333333
GO:0031347	regulation of defense	-5.50513094	19.42579733	5	41.66666667
GO:0002526	acute inflammatory re	-5.497404425	100.7033333	3	25
GO:0031349	positive regulation (	-5.417727453	34.84544406	4	33.33333333
GO:0050673	epithelial cell prol:	-5.25836222	83.91944444	3	25
GO:0009725	response to hormone	-5.218143654	16.96484726	5	41.66666667
GO:0032642	regulation of chemok:	-5.160390921	77.86340206	3	25
GO:0032755	positive regulation (	-5.160390921	77.86340206	3	25
GO:0002718	regulation of cytokir	-5.14698561	77.06887755	3	25
GO:0014013	regulation of glioger	-5.120587704	75.5275	3	25
GO:0045765	regulation of angioge	-5.113751496	29.18937198	4	33.33333333
GO:0022612	gland morphogenesis	-5.107589569	74.77970297	3	25
GO:0032677	regulation of interle	-5.094721756	74.04656863	3	25
GO:1901342	regulation of vascula	-5.084225129	28.69040836	4	33.33333333
GO:0002521	leukocyte differentia	-5.064826478	28.36713615	4	33.33333333
GO:0050767	regulation of neuroge	-5.036144088	27.8956602	4	33.33333333
GO:0002824	positive regulation (	-4.996177714	68.66136364	3	25
GO:0002821	positive regulation (	-4.938225397	65.67608696	3	25
GO:0002708	positive regulation (	-4.915759201	64.5534188	3	25
GO:0001889	liver development	-4.861260365	61.90778689	3	25
GO:0061008	hepaticobiliary syste	-4.829641567	60.422	3	25
GO:0031331	positive regulation (	-4.7022558	22.93925588	4	33.33333333
GO:0002705	positive regulation (	-4.701003378	54.73007246	3	25
GO:0002687	positive regulation (	-4.673071609	53.56560284	3	25
GO:0051090	regulation of DNA-bir	-4.656362351	22.32889874	4	33.33333333
GO:0050796	regulation of insulir	-4.618978631	51.3792517	3	25
GO:0031099	regeneration	-4.610182133	51.03209459	3	25
GO:0001819	positive regulation (	-4.568228225	21.20070175	4	33.33333333
GO:0051223	regulation of protein	-4.498216198	20.34410774	4	33.33333333
GO:0009896	positive regulation (	-4.47102395	20.02054341	4	33.33333333
GO:0002700	regulation of produc	-4.453652828	45.2260479	3	25
GO:0045321	leukocyte activation	-4.431060435	19.55404531	4	33.33333333
GO:0032675	regulation of interle	-4.415474049	43.91133721	3	25
GO:0070201	regulation of establ:	-4.414691647	19.36602564	4	33.33333333
GO:0002822	regulation of adaptiv	-4.40797411	43.65751445	3	25
GO:0002706	regulation of lymphoc	-4.393105859	43.15857143	3	25
GO:0034248	regulation of cellula	-4.392046134	19.1087919	4	33.33333333
GO:0090276	regulation of peptide	-4.378409769	42.67090395	3	25
GO:0002791	regulation of peptide	-4.35667996	41.95972222	3	25
GO:0090087	regulation of peptide	-4.335316204	41.27185792	3	25
GO:0002819	regulation of adaptiv	-4.30049114	40.17420213	3	25
GO:0050731	positive regulation (	-4.286824807	39.75131579	3	25

GO:0030097	hemopoiesis	-4.189812584	16.95342312	4	33.33333333
GO:0050769	positive regulation (	-4.086143073	34.0213964	3	25
GO:0048534	hematopoietic or lym]	-4.056339932	15.66148263	4	33.33333333
GO:0046883	regulation of hormon(	-4.035011421	32.69588745	3	25
GO:0002703	regulation of leukoc;	-4.007477545	32.00317797	3	25
GO:0002520	immune system develo]	-3.954989179	14.7442655	4	33.33333333
GO:0002699	positive regulation (	-3.943757353	30.4546371	3	25
GO:0050708	regulation of protei]	-3.923216133	29.97123016	3	25
GO:0051962	positive regulation (	-3.839486834	28.07713755	3	25
GO:0010720	positive regulation (	-3.712741175	25.43013468	3	25
GO:0002697	regulation of immune	-3.492441784	21.39589235	3	25
GO:0048871	multicellular organi:	-3.471005235	21.03830084	3	25
GO:0006417	regulation of transl:	-3.146147736	16.27747845	3	25
GO:0010817	regulation of hormon(	-3.062197279	15.22731855	3	25
GO:1903530	regulation of secret:	-2.934844769	13.75728597	3	25
GO:0050778	positive regulation (	-2.912260336	13.51118068	3	25
GO:0051046	regulation of secret:	-2.811487741	12.46328383	3	25
GO:0030335	positive regulation (	-11.21106684	35.96547619	8	66.66666667
GO:2000147	positive regulation (	-11.065615	34.48744292	8	66.66666667
GO:0051272	positive regulation (	-10.99514868	33.79306488	8	66.66666667
GO:0040017	positive regulation (	-10.97775712	33.62381747	8	66.66666667
GO:0033138	positive regulation (	-9.404772532	117.644081	5	41.66666667
GO:0033135	regulation of peptid;	-8.752300041	87.41608796	5	41.66666667
GO:0045598	regulation of fat ce]	-6.64803433	70.91784038	4	33.33333333
GO:0050890	cognition	-3.674592955	24.68218954	3	25
GO:0070141	response to UV-A	-10.84624395	719.3095238	4	33.33333333
GO:0007346	regulation of mitoti(	-9.76146523	36.94566737	7	58.33333333
GO:0050999	regulation of nitric-	-8.800309342	239.7698413	4	33.33333333
GO:0010564	regulation of cell c;	-8.655549901	25.61494671	7	58.33333333
GO:2000045	regulation of G1/S t]	-8.521782176	78.67447917	5	41.66666667
GO:0032768	regulation of monoox;	-8.254029286	176.6725146	4	33.33333333
GO:1902806	regulation of cell c;	-8.169836329	66.95700355	5	41.66666667
GO:0071902	positive regulation (	-7.949738319	60.51883013	5	41.66666667
GO:0090068	positive regulation (	-7.576516909	50.96322537	5	41.66666667
GO:0051341	regulation of oxidor(	-7.142544241	94.1152648	4	33.33333333
GO:1901990	regulation of mitoti(	-7.009625737	39.21469367	5	41.66666667
GO:0009895	negative regulation (	-7.002913615	39.0929089	5	41.66666667
GO:0045787	positive regulation (	-6.956515306	38.26114488	5	41.66666667
GO:0071900	regulation of protei]	-6.691876347	33.83848566	5	41.66666667
GO:0045737	positive regulation (	-6.669866492	243.6370968	3	25
GO:0050678	regulation of epithe]	-6.651769771	33.21350044	5	41.66666667
GO:0045860	positive regulation (	-6.573776223	32.03032231	5	41.66666667
GO:0048732	gland development	-6.53047203	31.39131338	5	41.66666667
GO:1904031	positive regulation (	-6.507074887	215.7928571	3	25
GO:1901987	regulation of cell c;	-6.4567705	30.33232932	5	41.66666667
GO:0048568	embryonic organ deve]	-6.302229824	28.2240284	5	41.66666667
GO:0071363	cellular response to	-6.217459371	27.12913075	5	41.66666667
GO:0033674	positive regulation (	-6.176327272	26.61293164	5	41.66666667
GO:0070848	response to growth f:	-6.087719559	25.53329953	5	41.66666667
GO:0050679	positive regulation (	-5.976822836	48.18341308	4	33.33333333
GO:1900087	positive regulation (	-5.955034193	142.504717	3	25
GO:0051347	positive regulation (	-5.819451327	22.51863447	5	41.66666667
GO:0051054	positive regulation (	-5.803792592	43.59451659	4	33.33333333
GO:1903078	positive regulation (	-5.791132123	125.8791667	3	25

GO:1902808	positive regulation o	-5.665508838	114.4356061	3	25
GO:1904377	positive regulation o	-5.645711134	112.7276119	3	25
GO:0045859	regulation of protein	-5.514983361	19.51614987	5	41.66666667
GO:0043549	regulation of kinase	-5.159170571	16.49792486	5	41.66666667
GO:0000079	regulation of cyclin-	-5.14698561	77.06887755	3	25
GO:1901992	positive regulation o	-5.120587704	75.5275	3	25
GO:1904029	regulation of cyclin-	-5.107589569	74.77970297	3	25
GO:1903076	regulation of protein	-5.081981696	73.3276699	3	25
GO:1905477	positive regulation o	-5.081981696	73.3276699	3	25
GO:0042176	regulation of protein	-4.882310057	25.49451477	4	33.33333333
GO:0032355	response to estradiol	-4.850633687	61.40447154	3	25
GO:1901989	positive regulation o	-4.850633687	61.40447154	3	25
GO:1904375	regulation of protein	-4.84009456	60.90927419	3	25
GO:0045931	positive regulation o	-4.798785716	59.00585938	3	25
GO:0048608	reproductive structur	-4.781687542	24.03420843	4	33.33333333
GO:0061458	reproductive system o	-4.769527597	23.86334913	4	33.33333333
GO:0001890	placenta development	-4.62783626	51.73116438	3	25
GO:1905475	regulation of protein	-4.415474049	43.91133721	3	25
GO:0007610	behavior	-4.280283895	17.88691533	4	33.33333333
GO:0051345	positive regulation o	-4.238803443	17.45291739	4	33.33333333
GO:0007167	enzyme linked recepto	-4.170060503	16.75596229	4	33.33333333
GO:0097305	response to alcohol	-4.018418881	32.2767094	3	25
GO:0016032	viral process	-3.948945624	30.57793522	3	25
GO:0006468	protein phosphorylati	-3.913771394	14.38619048	4	33.33333333
GO:0001701	in utero embryonic de	-3.395447217	19.82349081	3	25
GO:0001558	regulation of cell gr	-3.287111103	18.19939759	3	25
GO:0043009	chordate embryonic de	-2.765122419	12.00755167	3	25
GO:0009792	embryo development en	-2.724319802	11.61961538	3	25
GO:0040008	regulation of growth	-2.705375212	11.44356061	3	25
GO:0061024	membrane organization	-2.508644588	9.758074935	3	25
GO:0034612	response to tumor nec	-10.13007159	74.77970297	6	50
GO:0048661	positive regulation o	-9.667349671	132.504386	5	41.66666667
GO:0031663	lipopolysaccharide-me	-9.028955477	272.1711712	4	33.33333333
GO:0043491	protein kinase B sign	-8.980742879	265.0087719	4	33.33333333
GO:0045429	positive regulation o	-8.843682947	245.6178862	4	33.33333333
GO:0031622	positive regulation o	-8.776198424	1078.964286	3	25
GO:1904407	positive regulation o	-8.757997583	234.1937984	4	33.33333333
GO:2001233	regulation of apopto	-8.571765654	41.15940054	6	50
GO:0031620	regulation of fever g	-8.396181301	839.1944444	3	25
GO:0031652	positive regulation o	-8.396181301	839.1944444	3	25
GO:0048660	regulation of smooth	-8.389353002	74.04656863	5	41.66666667
GO:0071356	cellular response to	-8.289002852	70.71863296	5	41.66666667
GO:0045428	regulation of nitric	-8.133456001	165.0874317	4	33.33333333
GO:0031667	response to nutrient	-8.119232771	34.56636156	6	50
GO:0080164	regulation of nitric	-8.048251417	157.3489583	4	33.33333333
GO:0031650	regulation of heat ge	-7.978329092	629.3958333	3	25
GO:0009991	response to extracell	-7.93094741	32.1393617	6	50
GO:2001234	negative regulation o	-7.759754986	55.45337739	5	41.66666667
GO:0043154	negative regulation o	-7.721093961	130.7835498	4	33.33333333
GO:0046889	positive regulation o	-7.546838356	118.4745098	4	33.33333333
GO:2000117	negative regulation o	-7.546838356	118.4745098	4	33.33333333
GO:0043269	regulation of ion tra	-6.961145068	22.05182482	6	50
GO:0080135	regulation of cellula	-6.935081977	21.82875723	6	50
GO:0050995	negative regulation o	-6.907137429	290.4903846	3	25

GO:0062012	regulation of small r	-6.872956641	36.80677388	5	41.66666667
GO:0045861	negative regulation c	-6.823130426	35.96547619	5	41.66666667
GO:0030162	regulation of proteol	-6.752976862	20.33041723	6	50
GO:0034763	negative regulation c	-6.660351327	71.42080378	4	33.33333333
GO:0032770	positive regulation c	-6.627211597	236.0234375	3	25
GO:0043086	negative regulation c	-6.609134649	19.21819338	6	50
GO:0062013	positive regulation c	-6.599634295	68.97488584	4	33.33333333
GO:1901099	negative regulation c	-6.585915999	228.8712121	3	25
GO:2001240	negative regulation c	-6.585915999	228.8712121	3	25
GO:0045834	positive regulation c	-6.51809638	65.81917211	4	33.33333333
GO:1901652	response to peptide	-6.360709967	29.00441628	5	41.66666667
GO:0007565	female pregnancy	-6.334907245	59.2372549	4	33.33333333
GO:2001242	regulation of intrins	-6.324719917	58.89083821	4	33.33333333
GO:1903829	positive regulation c	-6.302229824	28.2240284	5	41.66666667
GO:0046890	regulation of lipid b	-6.284568348	57.5447619	4	33.33333333
GO:0051051	negative regulation c	-6.222079905	27.18772498	5	41.66666667
GO:2001239	regulation of extrins	-6.201839218	171.6534091	3	25
GO:0044706	multi-multicellular c	-6.070431879	50.86026936	4	33.33333333
GO:0009612	response to mechanica	-6.04438674	50.10116086	4	33.33333333
GO:0043281	regulation of cystein	-6.018734526	49.36437908	4	33.33333333
GO:0042063	gliogenesis	-5.935920237	47.0576324	4	33.33333333
GO:2000116	regulation of cystein	-5.818813285	43.97525473	4	33.33333333
GO:0051353	positive regulation c	-5.813311401	128.0127119	3	25
GO:0050994	regulation of lipid c	-5.7693274	123.8155738	3	25
GO:0071417	cellular response to	-5.766735876	21.96844095	5	41.66666667
GO:0034762	regulation of transmo	-5.740871934	21.7033046	5	41.66666667
GO:0010951	negative regulation c	-5.660486626	40.12084993	4	33.33333333
GO:0032890	regulation of organic	-5.588078567	107.8964286	3	25
GO:0051966	regulation of synapti	-5.588078567	107.8964286	3	25
GO:0010466	negative regulation c	-5.586553523	38.43638677	4	33.33333333
GO:0006970	response to osmotic s	-5.56942626	106.3767606	3	25
GO:0050766	positive regulation c	-5.56942626	106.3767606	3	25
GO:1901699	cellular response to	-5.55822937	19.91758966	5	41.66666667
GO:0019233	sensory perception of	-5.515036058	102.0641892	3	25
GO:0010827	regulation of glucose	-5.445912756	96.83012821	3	25
GO:0036293	response to decreased	-5.429669557	35.08826945	4	33.33333333
GO:0010632	regulation of epithel	-5.411788004	34.72528736	4	33.33333333
GO:0070482	response to oxygen le	-5.302747362	32.59007551	4	33.33333333
GO:1901214	regulation of neuron	-5.280665557	32.17358892	4	33.33333333
GO:0019217	regulation of fatty a	-5.229599984	82.0951087	3	25
GO:2001237	negative regulation c	-5.160390921	77.86340206	3	25
GO:0019216	regulation of lipid m	-5.143808045	29.70599803	4	33.33333333
GO:0050764	regulation of phagocy	-5.133718809	76.29040404	3	25
GO:0060341	regulation of cellula	-5.091115129	15.97451354	5	41.66666667
GO:0045833	negative regulation c	-5.056874937	71.93095238	3	25
GO:0021782	glial cell developmen	-5.020112909	69.93287037	3	25
GO:0051346	negative regulation c	-4.989407924	27.14375562	4	33.33333333
GO:0071675	regulation of mononuc	-4.926943031	65.10991379	3	25
GO:0010565	regulation of cellula	-4.758770154	57.21780303	3	25
GO:0052548	regulation of endopep	-4.753451694	23.63928013	4	33.33333333
GO:0050804	modulation of chemica	-4.74547178	23.5288162	4	33.33333333
GO:0099177	regulation of trans-s	-4.741496173	23.47397047	4	33.33333333
GO:0043467	regulation of genera	-4.739222761	56.36380597	3	25
GO:0036294	cellular response to	-4.710451492	55.12956204	3	25

GO:0045936	negative regulation (	-4.698382387	22.88712121	4	33.33333333
GO:0010563	negative regulation (	-4.69451801	22.83522298	4	33.33333333
GO:0050806	positive regulation (	-4.673071609	53.56560284	3	25
GO:0052547	regulation of peptida	-4.63760946	22.08406433	4	33.33333333
GO:0043535	regulation of blood v	-4.592769229	50.35166667	3	25
GO:0071453	cellular response to	-4.575591115	49.68914474	3	25
GO:2001236	regulation of extrins	-4.567088164	49.36437908	3	25
GO:0043271	negative regulation (	-4.550250724	48.72741935	3	25
GO:0051235	maintenance of locat:	-4.50103495	46.91149068	3	25
GO:0010001	glial cell different:	-4.453652828	45.2260479	3	25
GO:0001936	regulation of endothe	-4.371125063	42.43117978	3	25
GO:1902532	negative regulation (	-4.34142996	18.54573358	4	33.33333333
GO:0010594	regulation of endothe	-4.040591248	32.83804348	3	25
GO:0045732	positive regulation (	-4.023925449	32.41523605	3	25
GO:0051129	negative regulation (	-3.952535184	14.72270955	4	33.33333333
GO:0051348	negative regulation (	-3.83473168	27.97314815	3	25
GO:0001666	response to hypoxia	-3.815889811	27.56478102	3	25
GO:0051222	positive regulation (	-3.730078383	25.77730375	3	25
GO:1901653	cellular response to	-3.708444306	25.34479866	3	25
GO:1904951	positive regulation (	-3.662132404	24.44255663	3	25
GO:0030336	negative regulation (	-3.517930582	21.82875723	3	25
GO:2000146	negative regulation (	-3.463941669	20.92174515	3	25
GO:0045862	positive regulation (	-3.436085191	20.46815718	3	25
GO:0051271	negative regulation (	-3.436085191	20.46815718	3	25
GO:0040013	negative regulation (	-3.346468663	19.07260101	3	25
GO:0002683	negative regulation (	-3.256989554	17.77117647	3	25
GO:0034765	regulation of ion tra	-3.095603243	15.63716356	3	25
GO:0031400	negative regulation (	-3.005268353	14.55250482	3	25
GO:0060627	regulation of vesicle	-2.981332027	14.27741021	3	25
GO:0045786	negative regulation (	-6.726871104	34.39321494	5	41.66666667
GO:0045930	negative regulation (	-5.895979787	45.98325723	4	33.33333333
GO:2000134	negative regulation (	-5.31789215	87.82267442	3	25
GO:1902807	negative regulation (	-5.160390921	77.86340206	3	25
GO:0071346	cellular response to	-5.094721756	74.04656863	3	25
GO:0034341	response to interfer(	-4.84009456	60.90927419	3	25
GO:1901991	negative regulation (	-4.430608138	44.42794118	3	25
GO:0043523	regulation of neuron	-4.176260244	36.48671498	3	25
GO:0034976	response to endoplasm	-4.109551845	34.6456422	3	25
GO:1901988	negative regulation (	-4.040591248	32.83804348	3	25
GO:0010948	negative regulation (	-3.792729939	27.07078853	3	25
GO:0006974	cellular response to	-2.616723264	10.65267983	3	25
GO:0045087	innate immune respons	-2.524630705	9.885798429	3	25
GO:0007566	embryo implantation	-6.295809891	184.2134146	3	25
GO:0071214	cellular response to	-5.313898039	32.80238871	4	33.33333333
GO:0104004	cellular response to	-5.313898039	32.80238871	4	33.33333333
GO:0034644	cellular response to	-5.287782733	85.82670455	3	25
GO:0071482	cellular response to	-4.961092732	66.83849558	3	25
GO:0071478	cellular response to	-4.378409769	42.67090395	3	25
GO:0071496	cellular response to	-3.695641936	25.09219269	3	25
GO:0000165	MAPK cascade	-6.216500968	55.33150183	4	33.33333333
GO:0043410	positive regulation (	-6.100773572	25.68962585	5	41.66666667
GO:0071407	cellular response to	-6.040541171	24.97602513	5	41.66666667
GO:1901224	positive regulation (	-5.645711134	112.7276119	3	25
GO:0043408	regulation of MAPK ca	-5.384248452	18.34973275	5	41.66666667

GO:0070372	regulation of ERK1 ar	-5.291670183	32.38049303	4	33.33333333
GO:1901222	regulation of NIK/NF-	-4.996177714	68.66136364	3	25
GO:0043406	positive regulation o	-4.926943031	65.10991379	3	25
GO:0048565	digestive tract devel	-4.80898848	59.47047244	3	25
GO:0055123	digestive system deve	-4.691624691	54.33633094	3	25
GO:0046328	regulation of JNK cas	-4.682314429	53.94821429	3	25
GO:0019722	calcium-mediated sign	-4.636755866	52.08793103	3	25
GO:0043405	regulation of MAP kin	-4.335316204	41.27185792	3	25
GO:0032872	regulation of stress-	-4.240122012	38.33883249	3	25
GO:0070302	regulation of stress-	-4.220622472	37.76375	3	25
GO:0019932	second-messenger-medi	-4.133403424	35.2932243	3	25
GO:0070374	positive regulation o	-4.097793121	34.33068182	3	25
GO:0098609	cell-cell adhesion	-2.971889393	14.17026266	3	25
GO:0035239	tube morphogenesis	-5.462972987	19.04374685	5	41.66666667
GO:0001525	angiogenesis	-5.237354264	31.37175493	4	33.33333333
GO:0048514	blood vessel morphoge	-4.781687542	24.03420843	4	33.33333333
GO:0001568	blood vessel developm	-4.47102395	20.02054341	4	33.33333333
GO:0001944	vasculature developme	-4.395262136	19.14512041	4	33.33333333
GO:0010959	regulation of metal i	-3.324264122	18.74131514	3	25
GO:0097237	cellular response to	-4.961092732	66.83849558	3	25
GO:0009636	response to toxic sub	-4.046195921	32.98144105	3	25
GO:0007568	aging	-3.849051703	28.28745318	3	25
GO:0042742	defense response to b	-3.514256983	21.76585014	3	25
GO:0002252	immune effector proce	-3.248096021	17.64661215	3	25

Hits	Log(q-value)
AKT1|IL1B|IL6|CXCL8|MPC	-9.07106
AKT1|IL1B|IL6|CXCL8|MPC	-9.07106
AKT1|IL1B|IL6|CXCL8|CCL	-8.28072
AKT1|IL1B|IL6|CXCL8|CCL	-7.61032
AKT1|IL1B|IL6|CXCL8|MPC	-7.30098
AKT1|IL1B|IL6|CXCL8|CCL	-7.17269
AKT1|IL1B|IL6|CXCL8|CCL	-7.09531
AKT1|IL1B|IL6|CXCL8|PTG	-7.09531
IL1B|IL6|CXCL8|MMP9|CCL	-6.93798
AKT1|EGFR|IL1B|IL6|CXCL	-6.86934
AKT1|IL1B|IL6|CXCL8|PTG	-6.86934
AKT1|IL1B|IL6|CXCL8|CCL	-6.05604
IL1B|IL6|CXCL8|PTGS2|CC	-5.82334
AKT1|IL6|CXCL8|CCL2|TNF	-5.47936
AKT1|IL1B|IL6|CCL2|TNF	-5.25758
AKT1|IL1B|IL6|CCL2|TNF	-4.93136
AKT1|IL1B|IL6|CCL2|TNF	-4.63635
IL1B|IL6|CXCL8|CCL2	-4.62345
AKT1|IL1B|IL6|CXCL8|CCL	-4.51688
IL1B|IL6|CXCL8|CCL2	-4.482
AKT1|IL1B|IL6|CCL2|TNF	-4.35207
AKT1|IL1B|IL6|CCL2|TNF	-4.28359
AKT1|IL1B|IL6|CCL2|TNF	-4.25741
AKT1|IL1B|IL6|CCL2|TNF	-4.21917
IL1B|IL6|CXCL8|CCL2	-4.00975
AKT1|IL1B|IL6|CCL2	-3.89697
AKT1|IL1B|IL6|CCL2|TNF	-3.66607
IL1B|CXCL8|CCL2	-3.55312
IL6|CXCL8|CCL2|TNF	-3.54202
AKT1|IL1B|IL6|CCL2|TNF	-3.53207
IL1B|CXCL8|CCL2	-3.48517
IL1B|CXCL8|CCL2	-3.4256
IL6|CCL2|TNF	-3.36388
IL1B|CXCL8|CCL2	-3.33307
AKT1|IL1B|IL6|CCL2	-3.31195
IL1B|CXCL8|CCL2	-3.29428
AKT1|IL1B|IL6|CCL2	-3.20982
IL6|CXCL8|CCL2	-3.08202
IL1B|CXCL8|CCL2	-3.01342
IL1B|IL6|CXCL8|CCL2	-2.76166
IL1B|IL6|CXCL8|CCL2	-2.75252
AKT1|IL1B|IL6|CCL2	-2.69877
IL6|CXCL8|CCL2	-2.41633
AKT1|IL1B|CCL2	-2.19145
AKT1|CCND1|EGFR|IL1B|IL	-8.59072
CCND1|EGFR|IL1B|IL6|MMF	-6.10412
CCND1|IL1B|PTGS2|TNF|TF	-4.429
CCND1|IL1B|MMP9|TNF	-4.38916
IL1B|MMP9|TNF|TP53	-2.82297
IL1B|TNF|TP53	-2.21152
AKT1|EGFR|IL6|MMP9|MPO|	-8.1414

AKT1|CCND1|EGFR|MMP9|PT	-7.83132
AKT1|EGFR|IL6|MMP9|MPO|	-7.53214
AKT1|CCND1|EGFR|MMP9|PT	-7.13633
AKT1|EGFR|IL6|MMP9|MPO|	-7.06461
AKT1|CCND1|EGFR|IL6|MMF	-6.8305
AKT1|EGFR|IL6|MMP9|MPO	-6.52536
AKT1|CCND1|EGFR|MMP9|PT	-6.39223
AKT1|EGFR|IL6|MMP9|MPO	-5.92111
AKT1|CCND1|EGFR|MMP9|PT	-4.60688
AKT1|EGFR|MMP9	-4.35552
AKT1|MMP9|TP53	-4.13305
AKT1|EGFR|MMP9|PTGS2	-4.13305
AKT1|EGFR|MMP9|PTGS2	-3.91776
AKT1|EGFR|MMP9	-3.85848
AKT1|EGFR|MMP9|PTGS2	-3.63281
AKT1|MMP9|PTGS2	-3.29428
AKT1|MMP9|TP53	-2.93234
AKT1|MMP9|PTGS2	-2.71511
AKT1|EGFR|MMP9	-1.71736
IL1B|IL6|MMP9|PTGS2|TNF	-8.1414
EGFR|IL6|TNF|TP53	-6.93798
EGFR|IL6|TNF|TP53	-6.89449
IL1B|IL6|PTGS2|TNF	-6.86934
IL1B|IL6|TNF	-6.17943
IL1B|IL6|PTGS2|TNF	-6.05604
EGFR|IL6|TNF|TP53	-6.01519
EGFR|IL6|TNF|TP53	-5.92111
EGFR|IL6|TNF|TP53	-5.92111
EGFR|IL6|TP53	-5.58984
IL6|TNF|TP53	-5.31405
IL6|MMP9|PTGS2|CCL2|TNF	-5.28293
IL6|MMP9|PTGS2|CCL2|TNF	-5.23407
IL1B|IL6|TNF	-5.18171
IL1B|IL6|TNF	-5.1083
IL6|MMP9|PTGS2|CCL2|TNF	-4.98852
IL6|TNF|TP53	-4.85486
IL6|TNF|TP53	-4.74695
IL6|TNF|TP53	-4.74695
IL1B|IL6|TNF	-4.67355
IL1B|IL6|PTGS2	-4.55857
CCND1|IL6|TNF	-4.55857
IL1B|IL6|TNF	-4.53046
EGFR|IL6|TP53	-4.53046
CCND1|IL6|PTGS2|TNF	-4.51446
IL1B|IL6|TNF|TP53	-4.51142
EGFR|IL6|TP53	-4.51142
IL1B|IL6|PTGS2|TNF	-4.482
IL1B|IL6|TNF|TP53	-4.482
IL1B|IL6|MMP9|PTGS2|TNF	-4.45966
AKT1|EGFR|IL6|TNF|TP53	-4.42005
CCND1|IL6|PTGS2|TNF	-4.38916
AKT1|IL1B|IL6|TNF|TP53	-4.27418
IL1B|IL6|CXCL8|PTGS2|TN	-4.26725
IL1B|IL6|TNF|TP53	-4.19357

AKT1|IL1B|IL6|TNF	-4.18648
AKT1|CCND1|IL6|TNF|TP53	-4.09916
AKT1|IL1B|IL6|TNF|TP53	-4.06287
IL1B|IL6|PTGS2	-3.85848
IL1B|IL6|TNF	-3.80557
AKT1|IL6|TNF	-3.78938
IL1B|IL6|TNF	-3.77106
CCND1|IL6|TNF	-3.75306
EGFR|IL6|TNF|TP53	-3.67518
IL1B|IL6|TNF	-3.67518
AKT1|IL1B|IL6|TNF	-3.66607
IL1B|IL6|CXCL8|TNF|TP53	-3.64703
CCND1|IL6|PTGS2|TNF	-3.62867
IL1B|IL6|MMP9|PTGS2|TNF	-3.61833
IL1B|IL6|TNF	-3.61277
IL1B|IL6|PTGS2|TNF	-3.54376
CCND1|IL6|TNF	-3.41295
AKT1|CCND1|IL6|PTGS2|TN	-3.37862
IL1B|IL6|TNF	-3.33307
IL1B|IL6|TNF	-3.33307
IL1B|IL6|TNF	-3.32464
IL1B|IL6|TNF	-3.30751
IL1B|IL6|CXCL8|TNF	-3.3025
EGFR|IL6|TNF	-3.29997
IL1B|IL6|TNF	-3.29428
IL1B|IL6|CXCL8|TNF	-3.2886
IL6|MMP9|TNF|TP53	-3.2732
IL1B|IL6|TNF|TP53	-3.24799
IL1B|IL6|TNF	-3.21318
IL1B|IL6|TNF	-3.16369
IL1B|IL6|TNF	-3.14623
CCND1|IL6|TNF	-3.09503
CCND1|IL6|TNF	-3.07317
AKT1|IL1B|IL6|TNF	-2.96649
IL1B|IL6|TNF	-2.96649
IL6|CXCL8|TNF	-2.94764
AKT1|IL1B|IL6|TNF	-2.93242
IL1B|IL6|TNF	-2.90244
CCND1|IL6|TNF	-2.89511
IL1B|IL6|PTGS2|TNF	-2.85782
IL1B|IL6|PTGS2|TNF	-2.79469
AKT1|IL1B|IL6|TNF	-2.77033
IL1B|IL6|TNF	-2.75718
IL6|CXCL8|TNF|TP53	-2.73832
IL1B|IL6|TNF	-2.72655
IL1B|IL6|PTGS2|TNF	-2.72655
IL1B|IL6|TNF	-2.7212
IL1B|IL6|TNF	-2.71072
AKT1|IL6|TNF|TP53	-2.71072
IL1B|IL6|TNF	-2.69978
IL1B|IL6|TNF	-2.68207
IL1B|IL6|TNF	-2.66468
IL1B|IL6|TNF	-2.63117
IL6|TNF|TP53	-2.61882

IL6|MMP9|TNF|TP53	-2.52832
IL1B|IL6|TNF	-2.43234
IL6|MMP9|TNF|TP53	-2.40507
IL1B|IL6|TNF	-2.38876
IL1B|IL6|TNF	-2.36495
IL6|MMP9|TNF|TP53	-2.3137
IL1B|IL6|TNF	-2.30615
IL1B|IL6|TNF	-2.28684
IL1B|IL6|TNF	-2.20796
IL1B|IL6|TNF	-2.08839
IL1B|IL6|TNF	-1.87632
IL1B|IL6|PTGS2	-1.85605
AKT1|IL6|TNF	-1.54378
IL1B|IL6|TNF	-1.46208
IL1B|IL6|TNF	-1.33919
IL1B|IL6|TNF	-1.31772
IL1B|IL6|TNF	-1.21805
AKT1|EGFR|IL1B|IL6|CXCL	-7.86833
AKT1|EGFR|IL1B|IL6|CXCL	-7.83132
AKT1|EGFR|IL1B|IL6|CXCL	-7.83132
AKT1|EGFR|IL1B|IL6|CXCL	-7.83132
AKT1|EGFR|IL6|PTGS2|TNF	-6.7083
AKT1|EGFR|IL6|PTGS2|TNF	-6.17725
AKT1|IL6|PTGS2|TNF	-4.5016
EGFR|PTGS2|TNF	-2.05379
AKT1|CCND1|EGFR|MMP9	-7.73759
AKT1|CCND1|EGFR|IL1B|CC	-6.93798
AKT1|EGFR|IL1B|TNF	-6.18068
AKT1|CCND1|EGFR|IL1B|CC	-6.10119
AKT1|CCND1|EGFR|CCL2|TF	-6.01519
AKT1|EGFR|IL1B|TNF	-5.82207
AKT1|CCND1|EGFR|CCL2|TF	-5.74543
AKT1|CCND1|EGFR|IL1B|TN	-5.56809
AKT1|CCND1|EGFR|IL1B|TN	-5.24602
AKT1|EGFR|IL1B|TNF	-4.8578
AKT1|CCND1|EGFR|CCL2|TF	-4.74607
AKT1|EGFR|IL1B|TNF|TP53	-4.7445
AKT1|CCND1|EGFR|IL1B|TN	-4.7082
AKT1|CCND1|EGFR|IL1B|TN	-4.51688
AKT1|CCND1|EGFR	-4.51142
AKT1|CCND1|EGFR|CCL2|TN	-4.5016
AKT1|CCND1|EGFR|IL1B|TN	-4.46149
AKT1|CCND1|EGFR|IL6|TNF	-4.429
AKT1|CCND1|EGFR	-4.41615
AKT1|CCND1|EGFR|CCL2|TF	-4.37615
AKT1|EGFR|CXCL8|TNF|TP5	-4.25741
AKT1|EGFR|CXCL8|CCL2|TF	-4.19302
AKT1|CCND1|EGFR|IL1B|TN	-4.16168
AKT1|EGFR|CXCL8|CCL2|TF	-4.08741
AKT1|CCND1|EGFR|TNF	-3.99582
AKT1|CCND1|EGFR	-3.97672
AKT1|CCND1|EGFR|IL1B|TN	-3.85848
AKT1|EGFR|IL6|TNF	-3.85149
AKT1|EGFR|TNF	-3.84135

AKT1|CCND1|EGFR	-3.73536
AKT1|EGFR|TNF	-3.7227
AKT1|CCND1|EGFR|IL1B|TN	-3.62601
AKT1|CCND1|EGFR|IL1B|TN	-3.33307
AKT1|CCND1|EGFR	-3.32464
AKT1|CCND1|EGFR	-3.30751
AKT1|CCND1|EGFR	-3.29997
AKT1|EGFR|TNF	-3.2886
AKT1|EGFR|TNF	-3.2886
AKT1|EGFR|IL1B|TNF	-3.11443
CCND1|EGFR|PTGS2	-3.08768
AKT1|CCND1|EGFR	-3.08768
AKT1|EGFR|TNF	-3.08202
AKT1|CCND1|EGFR	-3.04552
AKT1|CCND1|EGFR|PTGS2	-3.03161
AKT1|CCND1|EGFR|PTGS2	-3.02103
AKT1|EGFR|PTGS2	-2.90983
AKT1|EGFR|TNF	-2.72655
AKT1|EGFR|PTGS2|TP53	-2.61359
AKT1|EGFR|CCL2|TNF	-2.57472
AKT1|EGFR|MMP9|TP53	-2.51115
AKT1|CCND1|TNF	-2.37466
EGFR|CCL2|TP53	-2.31012
AKT1|CCND1|EGFR|CCL2	-2.27861
AKT1|EGFR|TP53	-1.78511
AKT1|EGFR|TP53	-1.68134
AKT1|EGFR|TP53	-1.17279
AKT1|EGFR|TP53	-1.13309
AKT1|EGFR|TP53	-1.11524
AKT1|EGFR|TP53	-0.92179
AKT1|CXCL8|PTGS2|CCL2|T	-7.17269
AKT1|IL6|MMP9|PTGS2|TNF	-6.89449
AKT1|IL1B|CCL2|TNF	-6.3726
AKT1|IL1B|CCL2|TNF	-6.33698
AKT1|IL1B|PTGS2|TNF	-6.21215
IL1B|PTGS2|TNF	-6.17943
AKT1|IL1B|PTGS2|TNF	-6.17725
AKT1|IL1B|MMP9|PTGS2|TN	-6.04669
IL1B|PTGS2|TNF	-5.92111
IL1B|PTGS2|TNF	-5.92111
AKT1|IL6|MMP9|PTGS2|TNF	-5.92111
AKT1|CXCL8|CCL2|TNF|TP5	-5.84153
AKT1|IL1B|PTGS2|TNF	-5.71648
AKT1|CCND1|MPO|PTGS2|TN	-5.70955
AKT1|IL1B|PTGS2|TNF	-5.64575
IL1B|PTGS2|TNF	-5.58984
AKT1|CCND1|MPO|PTGS2|TN	-5.55603
AKT1|IL1B|MMP9|PTGS2|TN	-5.398
AKT1|MMP9|PTGS2|TNF	-5.36577
AKT1|IL1B|PTGS2|TNF	-5.23407
AKT1|MMP9|PTGS2|TNF	-5.23407
AKT1|IL1B|MMP9|PTGS2|CC	-4.7082
AKT1|EGFR|IL1B|PTGS2|TN	-4.69173
AKT1|IL1B|TNF	-4.67355

AKT1|IL1B|PTGS2|TNF|TP5	-4.64417
AKT1|MMP9|PTGS2|TNF|TP5	-4.60843
AKT1|IL1B|MMP9|PTGS2|TN	-4.55637
AKT1|IL1B|MMP9|TNF	-4.50594
AKT1|IL1B|TNF	-4.4847
AKT1|IL1B|MMP9|PTGS2|TN	-4.482
AKT1|IL1B|PTGS2|TNF	-4.47626
AKT1|IL1B|TNF	-4.46997
AKT1|IL1B|TNF	-4.46997
AKT1|IL1B|PTGS2|TNF	-4.42017
AKT1|MMP9|PTGS2|TNF|TP5	-4.29015
AKT1|IL1B|MMP9|PTGS2	-4.27418
AKT1|MMP9|PTGS2|TP53	-4.26725
AKT1|EGFR|IL1B|PTGS2|TN	-4.25741
AKT1|IL1B|PTGS2|TNF	-4.24596
AKT1|IL1B|MMP9|PTGS2|TN	-4.19302
AKT1|IL1B|TNF	-4.18427
AKT1|IL1B|MMP9|PTGS2	-4.07293
IL1B|MPO|PTGS2|TNF	-4.05246
AKT1|MMP9|PTGS2|TNF	-4.0323
AKT1|IL1B|CCL2|TNF	-3.96028
AKT1|MMP9|PTGS2|TNF	-3.85848
AKT1|IL1B|TNF	-3.85848
AKT1|IL1B|TNF	-3.82205
AKT1|EGFR|PTGS2|TNF|TP5	-3.82194
AKT1|IL1B|MMP9|CCL2|TNF	-3.80101
AKT1|MMP9|PTGS2|TNF	-3.73273
AKT1|IL1B|TNF	-3.67518
PTGS2|CCL2|TNF	-3.67518
AKT1|MMP9|PTGS2|TNF	-3.67518
PTGS2|TNF|TP53	-3.66607
IL1B|CCL2|TNF	-3.66607
AKT1|EGFR|PTGS2|TNF|TP5	-3.6582
PTGS2|CCL2|TNF	-3.62601
AKT1|IL1B|TNF	-3.56558
AKT1|PTGS2|TNF|TP53	-3.55312
AKT1|MMP9|PTGS2|TNF	-3.54202
AKT1|PTGS2|TNF|TP53	-3.44736
AKT1|CCL2|TNF|TP53	-3.43129
AKT1|IL1B|PTGS2	-3.38812
AKT1|IL1B|TNF	-3.33307
AKT1|IL1B|PTGS2|TNF	-3.32333
IL1B|CCL2|TNF	-3.3151
AKT1|EGFR|IL1B|PTGS2|TN	-3.29245
AKT1|IL1B|TNF	-3.26698
AKT1|IL1B|TNF	-3.23368
AKT1|MMP9|PTGS2|TNF	-3.20982
AKT1|CCL2|TNF	-3.15575
AKT1|IL1B|PTGS2	-3.01342
AKT1|MMP9|PTGS2|TNF	-3.00967
IL1B|PTGS2|CCL2|TNF	-3.00325
IL1B|PTGS2|CCL2|TNF	-3.00082
AKT1|TNF|TP53	-3.0001
AKT1|PTGS2|TP53	-2.97287

AKT1|IL1B|TNF|TP53	-2.9654
AKT1|IL1B|TNF|TP53	-2.96305
PTGS2|CCL2|TNF	-2.94764
AKT1|MMP9|PTGS2|TNF	-2.91727
AKT1|PTGS2|TNF	-2.87915
AKT1|PTGS2|TP53	-2.86343
AKT1|IL1B|TNF	-2.85782
MMP9|PTGS2|TNF	-2.84243
AKT1|IL1B|TNF	-2.79608
AKT1|IL1B|TNF	-2.75718
AKT1|CCL2|TNF	-2.69518
AKT1|IL1B|MMP9|PTGS2	-2.66815
AKT1|PTGS2|TNF	-2.39309
AKT1|IL1B|TNF	-2.37892
AKT1|IL1B|TNF|TP53	-2.31248
AKT1|IL1B|TP53	-2.20441
PTGS2|TNF|TP53	-2.18797
IL1B|PTGS2|TNF	-2.10454
AKT1|TNF|TP53	-2.08528
IL1B|PTGS2|TNF	-2.0425
AKT1|CCL2|TNF	-1.89947
AKT1|CCL2|TNF	-1.85014
AKT1|IL1B|TNF	-1.8246
AKT1|CCL2|TNF	-1.8246
AKT1|CCL2|TNF	-1.73728
AKT1|CCL2|TNF	-1.65236
MMP9|CCL2|TNF	-1.49436
AKT1|IL1B|TNF	-1.40627
IL1B|CCL2|TNF	-1.38345
CCND1|PTGS2|CCL2|TNF|TF	-4.53468
CCND1|CCL2|TNF|TP53	-3.92299
CCND1|CCL2|TP53	-3.45648
CCND1|CCL2|TP53	-3.33307
CCL2|TNF|TP53	-3.29428
CCL2|TNF|TP53	-3.08202
CCND1|CCL2|TP53	-2.73832
CCL2|TNF|TP53	-2.51606
CCND1|CXCL8|TP53	-2.4532
CCND1|CCL2|TP53	-2.39309
CCND1|CCL2|TP53	-2.166
CCND1|TNF|TP53	-1.02768
CCL2|TNF|TP53	-0.93668
IL1B|MMP9|PTGS2	-4.25411
IL1B|MMP9|PTGS2|TP53	-3.45648
IL1B|MMP9|PTGS2|TP53	-3.45648
MMP9|PTGS2|TP53	-3.43641
MMP9|PTGS2|TP53	-3.18488
MMP9|PTGS2|TP53	-2.69978
IL1B|PTGS2|TP53	-2.07366
EGFR|IL1B|CCL2|TNF	-4.19302
EGFR|IL1B|IL6|CCL2|TNF	-4.09763
EGFR|IL1B|PTGS2|CCL2|TN	-4.05137
EGFR|IL1B|TNF	-3.7227
EGFR|IL1B|IL6|CCL2|TNF	-3.51864

EGFR|IL1B|CCL2|TNF	-3.43829
EGFR|IL1B|TNF	-3.21318
EGFR|IL1B|TNF	-3.15575
EGFR|CXCL8|TNF	-3.05413
EGFR|CXCL8|TNF	-2.96168
EGFR|IL1B|TNF	-2.95388
EGFR|CXCL8|TNF	-2.91727
EGFR|IL1B|TNF	-2.66468
EGFR|IL1B|TNF	-2.57474
EGFR|IL1B|TNF	-2.55784
EGFR|CXCL8|TNF	-2.47577
EGFR|CCL2|TNF	-2.44272
EGFR|IL1B|TNF	-1.37512
AKT1|EGFR|CXCL8|PTGS2|C	-3.58049
AKT1|CXCL8|PTGS2|CCL2	-3.39392
AKT1|CXCL8|PTGS2|CCL2	-3.03161
AKT1|CXCL8|PTGS2|CCL2	-2.77033
AKT1|CXCL8|PTGS2|CCL2	-2.71122
AKT1|PTGS2|CCL2	-1.71736
MPO|PTGS2|TNF	-3.18488
MPO|PTGS2|TNF	-2.39618
MPO|PTGS2|TP53	-2.2151
IL6|MPO|TNF	-1.89697
IL6|MPO|TP53	-1.6446

GO-BP

GO	Description	LogP	Enrichment	#GeneInGC	%InGO
GO:0031965	nuclear membrane	-3.699894785	25.17583333	3	25
GO:0005635	nuclear envelope	-3.106070393	15.7677453	3	25
GO:0045121	membrane raft	-3.593789824	23.16794479	3	25
GO:0098857	membrane microdomain	-3.589884492	23.0970948	3	25

Hits	Log(q-value)
CCND1|EGFR|PTGS2	-0.780324778
CCND1|EGFR|PTGS2	-0.421449415
EGFR|PTGS2|TNF	-0.780324778
EGFR|PTGS2|TNF	-0.780324778

GO-BP

GO	Description	LogP	Enrichment	#GeneInG(	%InGO
GO:0005125	cytokine activity	-7.684559572	53.56560284	5	41.66666667
GO:0005126	cytokine receptor bi	-7.375604548	46.449877	5	41.66666667
GO:0048018	receptor ligand acti	-6.105143102	25.74216087	5	41.66666667
GO:0030546	signaling receptor a	-6.074746632	25.37886425	5	41.66666667
GO:0030545	signaling receptor r	-5.877457271	23.1395527	5	41.66666667
GO:0004672	protein kinase activ	-4.277285805	17.85520095	4	33.33333333
GO:0016773	phosphotransferase a	-3.984732187	15.00794834	4	33.33333333
GO:0016301	kinase activity	-3.845809037	13.81390032	4	33.33333333
GO:0019900	kinase binding	-3.79166398	13.37361664	4	33.33333333
GO:0019901	protein kinase bindi	-2.68488249	11.25596125	3	25
GO:0003682	chromatin binding	-2.857598959	12.9327911	3	25

Hits	Log(q-value)
IL1B|IL6|CXCL8|CCL2|TNF	-4.000342777
IL1B|IL6|CXCL8|CCL2|TNF	-3.992417749
IL1B|IL6|CXCL8|CCL2|TNF	-2.992589828
IL1B|IL6|CXCL8|CCL2|TNF	-2.992589828
IL1B|IL6|CXCL8|CCL2|TNF	-2.892210481
AKT1|CCND1|EGFR|CCL2	-1.37122026
AKT1|CCND1|EGFR|CCL2	-1.145613432
AKT1|CCND1|EGFR|CCL2	-1.064682229
AKT1|CCND1|EGFR|TP53	-1.061689695
AKT1|CCND1|TP53	-0.04205838
EGFR|MPO|TP53	-0.173382164

GO-BP

GO	Description	LogP	Enrichment	#GeneInGC	%InGO
hsa05163	Human cytomegaloviru:	-19.55338222	111.8925926	10	83.33333333
hsa04657	IL-17 signaling pathv	-14.7550531	187.4796099	7	58.33333333
hsa04933	AGE-RAGE signaling pa	-14.561231	176.2308333	7	58.33333333
hsa04668	TNF signaling pathway	-14.20727007	157.3489583	7	58.33333333
hsa05142	Chagas disease	-11.93565637	148.0931373	6	50
hsa05135	Yersinia infection	-11.15268306	110.2591241	6	50
hsa05144	Malaria	-11.10046997	251.7583333	5	41.66666667
hsa04936	Alcoholic liver disea	-11.05792774	106.3767606	6	50
hsa05164	Influenza A	-10.5678649	88.33625731	6	50
hsa05171	Coronavirus disease -	-9.767239987	65.10991379	6	50
hsa05323	Rheumatoid arthritis	-9.714400517	135.3539427	5	41.66666667
hsa04620	Toll-like receptor s	-9.467477718	121.0376603	5	41.66666667
hsa04625	C-type lectin recepto	-9.467477718	121.0376603	5	41.66666667
hsa04932	Non-alcoholic fatty l	-8.591195383	81.21236559	5	41.66666667
hsa05134	Legionellosis	-8.254029286	176.6725146	4	33.33333333
hsa04621	NOD-like receptor sig	-8.216709999	68.41259058	5	41.66666667
hsa05133	Pertussis	-7.744169678	132.504386	4	33.33333333
hsa05132	Salmonella infection	-7.559030671	50.5538822	5	41.66666667
hsa04061	Viral protein intera	-7.261178152	100.7033333	4	33.33333333
hsa05146	Amoebiasis	-7.226438798	98.72875817	4	33.33333333
hsa04064	NF-kappa B signaling	-7.192387817	96.83012821	4	33.33333333
hsa04060	Cytokine-cytokine re	-7.192049028	42.67090395	5	41.66666667
hsa01523	Antifolate resistance	-6.669866492	243.6370968	3	25
hsa05010	Alzheimer disease	-6.623582324	32.78103299	5	41.66666667
hsa05143	African trypanosomia:	-6.4327577	204.1283784	3	25
hsa05332	Graft-versus-host di:	-6.263722304	179.827381	3	25
hsa05152	Tuberculosis	-6.235673005	55.9462963	4	33.33333333
hsa05130	Pathogenic Escherich:	-6.079202982	51.11844332	4	33.33333333
hsa05321	Inflammatory bowel d:	-5.6856151	116.1961538	3	25
hsa05140	Leishmaniasis	-5.462848958	98.08766234	3	25
hsa04640	Hematopoietic cell l:	-5.133718809	76.29040404	3	25
hsa04931	Insulin resistance	-5.020112909	69.93287037	3	25
hsa04380	Osteoclast different:	-4.798785716	59.00585938	3	25
hsa05022	Pathways of neurodege	-4.564656108	21.15616246	4	33.33333333
hsa05020	Prion disease	-3.820573729	27.66575092	3	25
hsa05417	Lipid and atheroscle:	-14.55464869	93.67751938	8	66.66666667
hsa05418	Fluid shear stress a	-11.11436314	108.6726619	6	50
hsa05131	Shigellosis	-9.603370238	61.15587045	6	50
hsa05168	Herpes simplex virus	-7.797069352	30.51616162	6	50
hsa04010	MAPK signaling pathwa	-7.199388612	42.81604308	5	41.66666667
hsa04071	Sphingolipid signali	-4.893680427	63.46848739	3	25
hsa04210	Apoptosis	-4.719970058	55.53492647	3	25
hsa05219	Bladder cancer	-11.55108361	307.0223577	5	41.66666667
hsa05206	MicroRNAs in cancer	-7.084897895	40.6061828	5	41.66666667
hsa04921	Oxytocin signaling pa	-4.558641612	49.04383117	3	25
hsa05200	Pathways in cancer	-11.39550618	37.9296924	8	66.66666667
hsa05167	Kaposi sarcoma-assoc:	-10.23612996	77.86340206	6	50
hsa05205	Proteoglycans in canc	-10.09139899	73.68536585	6	50
hsa05215	Prostate cancer	-9.621304027	129.7723368	5	41.66666667

hsa01522	Endocrine resistance	-9.598644955	128.4481293	5	41.66666667
hsa05165	Human papillomavirus	-8.840059621	45.63595166	6	50
hsa05162	Measles	-8.829726235	90.56055156	5	41.66666667
hsa04218	Cellular senescence	-8.577131899	80.6917735	5	41.66666667
hsa05160	Hepatitis C	-8.563160003	80.17781316	5	41.66666667
hsa05213	Endometrial cancer	-8.223087168	173.6264368	4	33.33333333
hsa05169	Epstein-Barr virus i	-8.013421988	62.31641914	5	41.66666667
hsa05218	Melanoma	-7.83969256	139.8657407	4	33.33333333
hsa05223	Non-small cell lung	-7.83969256	139.8657407	4	33.33333333
hsa05166	Human T-cell leukemi	-7.808134647	56.70232733	5	41.66666667
hsa05214	Glioma	-7.767558627	134.2711111	4	33.33333333
hsa05212	Pancreatic cancer	-7.744169678	132.504386	4	33.33333333
hsa05210	Colorectal cancer	-7.526245922	117.0968992	4	33.33333333
hsa05222	Small cell lung canc	-7.407611962	109.4601449	4	33.33333333
hsa04151	PI3K-Akt signaling p	-6.798651106	35.55908663	5	41.66666667
hsa04068	FoxO signaling pathw	-6.788655521	76.87277354	4	33.33333333
hsa05224	Breast cancer	-6.587745267	68.50566893	4	33.33333333
hsa05226	Gastric cancer	-6.564212322	67.58612975	4	33.33333333
hsa04630	JAK-STAT signaling p	-6.418672202	62.16255144	4	33.33333333
hsa05225	Hepatocellular carci	-6.355466223	59.94246032	4	33.33333333
hsa05221	Acute myeloid leukem	-5.645711134	112.7276119	3	25
hsa05230	Central carbon metab	-5.588078567	107.8964286	3	25
hsa05220	Chronic myeloid leuk	-5.480010827	99.37828947	3	25
hsa01521	EGFR tyrosine kinase	-5.429196395	95.60443038	3	25
hsa04066	HIF-1 signaling path	-5.008089448	69.2912844	3	25
hsa04919	Thyroid hormone sign	-4.871976048	62.41942149	3	25
hsa04510	Focal adhesion	-4.214188879	37.57587065	3	25
hsa05207	Chemical carcinogene	-4.145500477	35.62617925	3	25
hsa05161	Hepatitis B	-10.71024746	93.24382716	6	50
hsa05202	Transcriptional misr	-8.123967362	65.56206597	5	41.66666667
hsa04926	Relaxin signaling pa	-4.788663745	58.54844961	3	25
hsa04915	Estrogen signaling p	-4.701003378	54.73007246	3	25
hsa04072	Phospholipase D sign	-4.610182133	51.03209459	3	25
hsa04062	Chemokine signaling	-4.273304391	39.33723958	3	25

Hits	Log(q-value)
AKT1|CCND1|EGFR|IL1B|IL	-17.01682378
IL1B|IL6|CXCL8|MMP9|PTG	-12.62015024
AKT1|CCND1|IL1B|IL6|CXC	-12.62015024
AKT1|IL1B|IL6|MMP9|PTGS	-12.36968163
AKT1|IL1B|IL6|CXCL8|CCL	-10.17724918
AKT1|IL1B|IL6|CXCL8|CCL	-9.605304217
IL1B|IL6|CXCL8|CCL2|TNF	-9.605304217
AKT1|CCND1|IL1B|IL6|CXC	-9.60055054
AKT1|IL1B|IL6|CXCL8|CCL	-9.177434496
EGFR|IL1B|IL6|CXCL8|CCL	-8.461130466
IL1B|IL6|CXCL8|CCL2|TNF	-8.43311458
AKT1|IL1B|IL6|CXCL8|TNF	-8.292647111
AKT1|IL1B|IL6|PTGS2|TNF	-8.292647111
AKT1|IL1B|IL6|CXCL8|TNF	-7.473759592
IL1B|IL6|CXCL8|TNF	-7.179868841
IL1B|IL6|CXCL8|CCL2|TNF	-7.17151325
IL1B|IL6|CXCL8|TNF	-6.809671226
AKT1|IL1B|IL6|CXCL8|TNF	-6.635256085
IL6|CXCL8|CCL2|TNF	-6.368072385
IL1B|IL6|CXCL8|TNF	-6.343092869
IL1B|CXCL8|PTGS2|TNF	-6.336731823
IL1B|IL6|CXCL8|CCL2|TNF	-6.336731823
IL1B|IL6|TNF	-5.849311393
AKT1|IL1B|IL6|PTGS2|TNF	-5.811299752
IL1B|IL6|TNF	-5.644387285
IL1B|IL6|TNF	-5.498015874
AKT1|IL1B|IL6|TNF	-5.477265813
IL1B|IL6|CXCL8|TNF	-5.327974374
IL1B|IL6|TNF	-4.941448347
IL1B|PTGS2|TNF	-4.745834451
IL1B|IL6|TNF	-4.429669279
AKT1|IL6|TNF	-4.322403558
AKT1|IL1B|TNF	-4.125550133
IL1B|IL6|PTGS2|TNF	-3.920192268
IL1B|IL6|TNF	-3.203093379
AKT1|IL1B|IL6|CXCL8|MMP	-12.62015024
AKT1|IL1B|MMP9|CCL2|TNF	-9.605304217
AKT1|EGFR|IL1B|CXCL8|TN	-8.384305807
AKT1|IL1B|IL6|CCL2|TNF|	-6.828712633
AKT1|EGFR|IL1B|TNF|TP53	-6.336731823
AKT1|TNF|TP53	-4.208380333
AKT1|TNF|TP53	-4.058472879
CCND1|EGFR|CXCL8|MMP9|T	-9.859623212
CCND1|EGFR|MMP9|PTGS2|T	-6.238535532
CCND1|EGFR|PTGS2	-3.919710261
AKT1|CCND1|EGFR|IL6|CXC	-9.762037723
AKT1|CCND1|IL6|CXCL8|PT	-8.875662779
AKT1|CCND1|EGFR|MMP9|TN	-8.758960527
AKT1|CCND1|EGFR|MMP9|TF	-8.384305807

AKT1|CCND1|EGFR|MMP9|TF	-8.384305807
AKT1|CCND1|EGFR|PTGS2|T	-7.691107801
AKT1|CCND1|IL1B|IL6|TP5	-7.691107801
AKT1|CCND1|IL6|CXCL8|TF	-7.473759592
AKT1|CCND1|EGFR|TNF|TP5	-7.473759592
AKT1|CCND1|EGFR|TP53	-7.17151325
AKT1|CCND1|IL6|TNF|TP53	-6.995377485
AKT1|CCND1|EGFR|TP53	-6.847202162
AKT1|CCND1|EGFR|TP53	-6.847202162
AKT1|CCND1|IL6|TNF|TP53	-6.828712633
AKT1|CCND1|EGFR|TP53	-6.810783781
AKT1|CCND1|EGFR|TP53	-6.809671226
AKT1|CCND1|EGFR|TP53	-6.612936769
AKT1|CCND1|PTGS2|TP53	-6.504521975
AKT1|CCND1|EGFR|IL6|TP5	-5.961062668
AKT1|CCND1|EGFR|IL6	-5.959667255
AKT1|CCND1|EGFR|TP53	-5.783580585
AKT1|CCND1|EGFR|TP53	-5.768016569
AKT1|CCND1|EGFR|IL6	-5.637988616
AKT1|CCND1|EGFR|TP53	-5.582335774
AKT1|CCND1|MPO	-4.908493241
AKT1|EGFR|TP53	-4.857700098
AKT1|CCND1|TP53	-4.756365742
AKT1|EGFR|IL6	-4.718712755
AKT1|EGFR|IL6	-4.316629046
AKT1|CCND1|TP53	-4.192750102
AKT1|CCND1|EGFR	-3.586115455
AKT1|CCND1|EGFR	-3.522755886
AKT1|IL6|CXCL8|MMP9|TNF	-9.287632373
IL6|CXCL8|MMP9|MPO|TP53	-7.092558898
AKT1|EGFR|MMP9	-4.121337022
AKT1|EGFR|MMP9	-4.045258528
AKT1|EGFR|CXCL8	-3.960114416
AKT1|CXCL8|CCL2	-3.639835935
