# Supplementary material for: Herbal Medicine Hewei Jiangni Decoction Is Noninferior to Oral Omeprazole for the Treatment of Nonerosive Gastroesophageal Reflux Disease: A Randomized, Double-Blind, and Double-Dummy Controlled Trail
Source: Evid Based Complement Alternat Med. 2022 Sep 22;2022:9647003. doi: 10.1155/2022/9647003 (PMC9522514; doi:10.1155/2022/9647003)
Supplement: Supplementary Materials — (1) The active compounds and potential targets of HWJND. Supplementary materials. (2) The information of GERD-related targets. (3) The information on GO and KEGG pathway enrichment analysis. (4) The information of molecular docking. [file 9647003.f1.zip › 9647003.f1/Supplementary materials 4 the detailed information of molecular docking.docx]

**Supplementary materials 4 the detailed information of molecular docking**

**IL6——quercetin**

mode | affinity | dist from best mode

| (kcal/mol) | rmsd l.b. | rmsd u.b.

-----+------------+----------+----------

1 -7.8 0.000 0.000

2 -7.4 28.531 31.943

3 -7.1 2.678 3.462

4 -7.1 21.183 22.983

5 -7.1 13.960 15.711

6 -7.0 23.290 24.498

7 -7.0 12.791 14.362

8 -6.9 2.981 5.465

9 -6.9 22.603 24.215

**Grid Box Center:**

center_x = -18.362

center_y = 8.444

center_z = 36.281

size_x = 82.15

size_y = 74.4

size_z = 97.65

**IL6——beta-sitosterol**

mode | affinity | dist from best mode

| (kcal/mol) | rmsd l.b. | rmsd u.b.

-----+------------+----------+----------

1 -6.8 0.000 0.000

2 -6.8 48.346 51.047

3 -6.7 66.942 69.445

4 -6.7 3.090 8.845

5 -6.6 57.105 60.201

6 -6.4 27.283 28.551

7 -6.4 54.572 57.057

8 -6.4 27.931 29.371

9 -6.3 54.546 56.845

**Grid Box Center:**

center_x = -17.499

center_y = 8.719

center_z = 36.611

size_x = 82.8333333333

size_y = 74.9444444444

size_z = 100.583333333

**IL6——naringenin**

mode | affinity | dist from best mode

| (kcal/mol) | rmsd l.b. | rmsd u.b.

-----+------------+----------+----------

1 -7.3 0.000 0.000

2 -7.1 1.437 2.664

3 -7.0 30.558 31.489

4 -7.0 30.447 31.105

5 -6.7 2.957 6.898

6 -6.6 27.445 29.080

7 -6.6 37.528 40.560

8 -6.6 20.211 22.193

9 -6.5 19.838 21.144

**Grid Box Center:**

center_x = -15.877

center_y = 9.362

center_z = 36.444

size_x = 82.4

size_y = 77.25

size_z = 97.85

**TNF——quercetin**

mode | affinity | dist from best mode

| (kcal/mol) | rmsd l.b. | rmsd u.b.

-----+------------+----------+----------

1 -10.4 0.000 0.000

2 -10.3 2.130 3.493

3 -10.2 1.315 6.277

4 -9.8 1.789 2.934

5 -9.3 5.411 8.690

6 -8.6 11.460 13.972

7 -8.1 11.502 13.257

8 -7.9 12.298 15.276

9 -7.7 13.186 15.852

**Grid Box Center:**

center_x = -3.782

center_y = -2.112

center_z = 17.355

size_x = 61.95

size_y = 59.0

size_z = 59.9833333333

**TNF——beta-sitosterol**

mode | affinity | dist from best mode

| (kcal/mol) | rmsd l.b. | rmsd u.b.

-----+------------+----------+----------

1 -12.5 0.000 0.000

2 -11.3 3.154 9.028

**Grid Box Center:**

center_x = -5.835

center_y = -3.064

center_z = 17.383

size_x = 62.65

size_y = 62.65

size_z = 62.65

**TNF——naringenin**

Mode | affinity | dist from best mode

| (kcal/mol) | rmsd l.b. | rmsd u.b.

-----+------------+----------+----------

1 -10.4 0.000 0.000

2 -9.8 1.739 6.455

3 -9.8 5.379 9.243

4 -9.5 1.176 2.899

5 -8.4 7.210 9.934

6 -8.2 11.473 13.512

7 -7.9 3.460 5.837

8 -7.9 4.830 6.924

9 -7.7 11.754 14.110

**Grid Box Center:**

center_x = -4.882

center_y = -2.112

center_z = 17.803

size_x = 60.3333333333

size_y = 59.3277777778

size_z = 58.3222222222

**IL1B——quercetin**

mode | affinity | dist from best mode

| (kcal/mol) | rmsd l.b. | rmsd u.b.

-----+------------+----------+----------

1 -7.0 0.000 0.000

2 -7.0 26.639 28.542

3 -7.0 27.167 28.323

4 -6.5 27.298 28.531

5 -6.4 27.174 28.983

6 -6.3 1.380 6.354

7 -6.2 27.416 29.013

8 -6.1 23.571 24.894

9 -6.1 15.590 17.791

**Grid Box Center:**

center_x = 12.43

center_y = 15.608

center_z = -9.245

size_x = 47.25

size_y = 42.75

size_z = 45.0

**IL1B——beta-sitosterol**

mode | affinity | dist from best mode

| (kcal/mol) | rmsd l.b. | rmsd u.b.

-----+------------+----------+----------

1 -7.2 0.000 0.000

2 -7.1 1.357 2.107

3 -6.8 3.083 9.507

4 -6.7 3.803 10.207

5 -6.6 25.283 28.933

6 -6.6 3.439 9.953

7 -6.5 1.928 4.295

8 -6.5 1.967 4.307

9 -6.4 22.306 25.887

**Grid Box Center:**

center_x = 12.708

center_y = 15.845

center_z = -8.547

size_x = 47.25

size_y = 44.25

size_z = 44.25

**IL1B——naringenin**

Mode | affinity | dist from best mode

| (kcal/mol) | rmsd l.b. | rmsd u.b.

-----+------------+----------+----------

1 -7.1 0.000 0.000

2 -6.4 26.731 28.758

3 -6.3 23.826 25.928

4 -6.2 25.762 26.646

5 -6.2 12.443 14.045

6 -6.1 16.782 18.821

7 -6.1 25.945 27.582

8 -6.1 25.639 28.215

9 -5.9 2.035 6.709

**Grid Box Center:**

center_x = 12.987

center_y = 15.667

center_z = -8.434

size_x = 47.25

size_y = 47.25

size_z = 45.0
